# Global impact of ten-valent and 13-valent pneumococcal conjugate vaccines on invasive pneumococcal disease in all ages (the PSERENADE project): a global surveillance analysis

**DOI:** 10.1016/S1473-3099(24)00665-0

**Published:** 2024-12-17

**Authors:** Julia C Bennett, Maria Deloria Knoll, Eunice W Kagucia, Maria Garcia Quesada, Scott L Zeger, Marissa K Hetrich, Yangyupei Yang, Carly Herbert, Anju Ogyu, Adam L Cohen, Inci Yildirim, Brita A Winje, Anne von Gottberg, Delphine Viriot, Mark van der Linden, Palle Valentiner-Branth, Shigeru Suga, Anneke Steens, Anna Skoczynska, Nadja Sinkovec Zorko, J Anthony Scott, Camelia Savulescu, Larisa Savrasova, Juan Carlos Sanz, Fiona Russell, Leah J Ricketson, Rodrigo Puentes, J Pekka Nuorti, Jolita Mereckiene, Kimberley McMahon, Allison McGeer, Lucia Mad’arová, Grant A Mackenzie, Laura MacDonald, Tiia Lepp, Shamez N Ladhani, Karl G Kristinsson, Jana Kozakova, Nicola P Klein, Sanjay Jayasinghe, Pak-Leung Ho, Markus Hilty, Robert S Heyderman, Md Hasanuzzaman, Laura L Hammitt, Marcela Guevara, Marta Grgic-Vitek, Ryan Gierke, Theano Georgakopoulou, Yvonne Galloway, Idrissa Diawara, Stefanie Desmet, Philippe De Wals, Ron Dagan, Edoardo Colzani, Cheryl Cohen, Pilar Ciruela, Urtnasan Chuluunbat, Guanhao Chan, Romina Camilli, Michael G Bruce, Maria-Cristina C Brandileone, Godfrey Bigogo, Krow Ampofo, Katherine L O’Brien, Daniel R Feikin, Kyla Hayford, the PSERENADE Team

**Affiliations:** Johns Hopkins Bloomberg School of Public Health, Baltimore, MD, USA; Johns Hopkins Bloomberg School of Public Health, Baltimore, MD, USA; Epidemiology and Demography Department, KEMRI-Wellcome Trust Research Programme, Centre for Geographic Medicine Coast, Kilifi, Kenya; Johns Hopkins Bloomberg School of Public Health, Baltimore, MD, USA; Johns Hopkins Bloomberg School of Public Health, Baltimore, MD, USA; Johns Hopkins Bloomberg School of Public Health, Baltimore, MD, USA; Johns Hopkins Bloomberg School of Public Health, Baltimore, MD, USA; UMass Chan Medical School, Worcester, MA, USA; Johns Hopkins Bloomberg School of Public Health, Baltimore, MD, USA; Department of Immunizations, Vaccines and Biologicals, WHO, Geneva, Switzerland; Division of Bacterial Diseases, National Center for Immunizations and Respiratory Diseases, Centers for Disease Control and Prevention, Atlanta, GA, USA; Department of Pediatrics, Yale New Haven Children’s Hospital, New Haven, CT, USA; Faculty of Health Sciences, Oslo Metropolitan University, Oslo, Norway; Centre for Respiratory Diseases and Meningitis, National Institute for Communicable Diseases of the National Health Laboratory Service, Johannesburg, South Africa; School of Pathology, Faculty of Health Sciences, University of the Witwatersrand, Johannesburg, South Africa; French Public Health Agency, Saint-Maurice, France; Reference Laboratory for Streptococci, Department of Medical Microbiology, University Hospital RWTH Aachen, Aachen, Germany; Infectious Disease Epidemiology and Prevention, Statens Serum Institut, Copenhagen, Denmark; Infectious Disease Center and Department of Clinical Research, National Hospital Organization Mie Hospital, Tsu, Japan; Centre for Infectious Disease Control, National Institute for Public Health and the Environment, Bilthoven, Netherlands; National Reference Centre for Bacterial Meningitis, National Medicines Institute, Warsaw, Poland; Communicable Diseases Centre, National Institute of Public Health, Ljubljana, Slovenia; Epidemiology and Demography Department, KEMRI-Wellcome Trust Research Programme, Centre for Geographic Medicine Coast, Kilifi, Kenya; Epidemiology Department, Epiconcept, Paris, France; Institute of Public Health, Riga Stradins University, Riga, Latvia; Regional Public Health Laboratory, General Directorate of Public Health, Madrid, Spain; Murdoch Children’s Research Institute, Parkville, VIC, Australia; Centre for International Child Health, WHO Collaborating Centre for Research and Training in Child and Neonatal Health, University of Melbourne, Parkville, VIC, Australia; Department of Pediatrics, University of Calgary, Calgary, AB, Canada; Instituto de Salud Pública de Chile, Santiago, Chile; Health Sciences Unit, Faculty of Social Sciences, Tampere University, Tampere, Finland; Department of Health Security, Finnish Institute for Health and Welfare, Helsinki, Finland; Health Protection Surveillance Centre, Dublin, Ireland; Centre for Disease Control, Department of Health and Community Services, Darwin, NT, Australia; Toronto Invasive Bacterial Diseases Network and Department of Laboratory Medicine and Pathobiology, University of Toronto, Toronto, ON, Canada; National Reference Centre for Pneumococcal and Haemophilus Diseases, Regional Authority of Public Health, Banská Bystrica, Slovakia; New Vaccines Group, Murdoch Children’s Research Institute, Parkville, VIC, Australia; Medical Research Council Unit The Gambia at London School of Hygiene & Tropical Medicine, Banjul, The Gambia; Faculty of Infectious and Tropical Diseases, London School of Hygiene & Tropical Medicine, London, UK; Department of Paediatrics, University of Melbourne, Parkville, VIC, Australia; Public Health Scotland, Glasgow, UK; Department of Communicable Disease and Control and Health Protection, Public Health Agency of Sweden, Solna, Sweden; Immunisation and Countermeasures Division, UK Health Security Agency, London, UK; Department of Clinical Microbiology, Landspitali—The National University Hospital, Reykjavik, Iceland; National Institute of Public Health, Prague, Czech Republic; Vaccine Study Center, Kaiser Permanente; National Centre for Immunisation Research and Surveillance and Discipline of Child and Adolescent Health, Children’s Hospital Westmead Clinical School, Faculty of Medicine and Health, University of Sydney, Westmead, NSW, Australia; Department of Microbiology and Carol Yu Centre for Infection, Queen Mary Hospital, The University of Hong Kong, Hong Kong Special Administrative Region, China; Swiss National Reference Centre for Invasive Pneumococci, Institute for Infectious Diseases, University of Bern, Bern, Switzerland; NIHR Global Health Research Unit on Mucosal Pathogens, Division of Infection and Immunity, University College London, London, UK; Malawi Liverpool Wellcome Programme, Blantyre, Malawi; Child Health Research Foundation, Dhaka, Bangladesh; Epidemiology and Demography Department, KEMRI-Wellcome Trust Research Programme, Centre for Geographic Medicine Coast, Kilifi, Kenya; Johns Hopkins Bloomberg School of Public Health, Baltimore, MD, USA; Public Health Institute of Navarre—IdiSNA, Pamplona, Spain; CIBER Epidemiology and Public Health, Madrid, Spain; Communicable Diseases Centre, National Institute of Public Health, Ljubljana, Slovenia; Division of Bacterial Diseases, National Center for Immunizations and Respiratory Diseases, Centers for Disease Control and Prevention, Atlanta, GA, USA; Department for Vaccine Preventable Diseases, National Public Health Organization, Athens, Greece; Epidemiology Team, Institute of Environmental Science and Research, Wellington, New Zealand; Infectious Diseases Research Unit, Mohammed VI Center for Research and Innovation (CM6RI), Rabat, Morocco; Mohammed VI University of Sciences and Health, Mohammed VI Higher Institute of Biosciences and Biotechnologies (UM6SS), Casablanca, Morocco; National Reference Centre for Streptococcus Pneumoniae, University Hospitals Leuven, Leuven, Belgium; Department of Microbiology, Immunology and Transplantation, KU Leuven, Leuven, Belgium; Department of Social and Preventive Medicine, Laval University, Quebec, QC, Canada; The Shraga Segal Department of Microbiology, Immunology and Genetics, Faculty of Health Sciences, Ben-Gurion University of the Negev, Beer-Sheva, Israel; European Centre for Disease Prevention and Control, Solna, Sweden; Centre for Respiratory Diseases and Meningitis, National Institute for Communicable Diseases of the National Health Laboratory Service, Johannesburg, South Africa; School of Public Health, Faculty of Health Sciences, University of the Witwatersrand, Johannesburg, South Africa; Surveillance and Public Health Emergency Response, Public Health Agency of Catalonia, Barcelona, Spain; CIBER Epidemiology and Public Health, Madrid, Spain; National Center of Communicable Diseases, Ministry of Health, Ulaanbaatar, Mongolia; Singapore Ministry of Health, Communicable Diseases Division, Singapore; Department of Infectious Diseases, Italian National Institute of Health, Rome, Italy; Arctic Investigations Program, Division of Infectious Disease Readiness and Innovation, National Center for Emerging and Zoonotic Infectious Diseases, Centers for Disease Control and Prevention, Anchorage, AK, USA; National Laboratory for Meningitis and Pneumococcal Infections, Center of Bacteriology, Institute Adolfo Lutz, São Paulo, Brazil; Centre for Global Health Research, Kenya Medical Research Institute, Kisumu, Kenya; Division of Pediatric Infectious Diseases, Department of Pediatrics, University of Utah Health Sciences Center, Salt Lake City, UT, USA; Johns Hopkins Bloomberg School of Public Health, Baltimore, MD, USA; Department of Immunizations, Vaccines and Biologicals, WHO, Geneva, Switzerland; Johns Hopkins Bloomberg School of Public Health, Baltimore, MD, USA

## Abstract

**Background:**

Pneumococcal conjugate vaccines (PCVs) that are ten-valent (PCV10) and 13-valent (PCV13) became available in 2010. We evaluated their global impact on invasive pneumococcal disease (IPD) incidence in all ages.

**Methods:**

Serotype-specific IPD cases and population denominators were obtained directly from surveillance sites using PCV10 or PCV13 in their national immunisation programmes and with a primary series uptake of at least 50%. Annual incidence rate ratios (IRRs) were estimated comparing the incidence before any PCV with each year post-PCV10 or post-PCV13 introduction using Bayesian multi-level, mixed-effects Poisson regressions, by site and age group. All site-weighted average IRRs were estimated using linear mixed-effects regression, stratified by product and previous seven-valent PCV (PCV7) effect (none, moderate, or substantial).

**Findings:**

Analyses included 32 PCV13 sites (488 758 cases) and 15 PCV10 sites (46 386 cases) in 30 countries, primarily high income (39 sites), using booster dose schedules (41 sites). By 6 years after PCV10 or PCV13 introduction, IPD due to PCV10-type serotypes and PCV10-related serotype 6A declined substantially for both products (age <5 years: 83–99% decline; ≥65 years: 54–96% decline). PCV7-related serotype 19A increases before PCV10 or PCV13 introduction were reversed at PCV13 sites (age <5 years: 61–79% decline relative to before any PCV; age ≥65 years: 7–26% decline) but increased at PCV10 sites (age <5 years: 1·6–2·3-fold; age ≥65 years: 3·6–4·9-fold). Serotype 3 IRRs had no consistent trends for either product or age group. Non-PCV13-type IPD increased similarly for both products (age <5 years: 2·3–3·3-fold; age ≥65 years: 1·7–2·3-fold). Despite different serotype 19A trends, all-serotype IPD declined similarly between products among children younger than 5 years (58–74%); among adults aged 65 years or older, declines were greater at PCV13 (25–29%) than PCV10 (4–14%) sites, but other differences between sites precluded attribution to product.

**Interpretation:**

Long-term use of PCV10 or PCV13 reduced IPD substantially in young children and more moderately in older ages. Non-vaccine-type serotypes increased approximately two-fold to three-fold by 6 years after introduction of PCV10 or PCV13. Continuing serotype 19A increases at PCV10 sites and declines at PCV13 sites suggest that PCV13 use would further reduce IPD at PCV10 sites.

**Funding:**

Bill & Melinda Gates Foundation as part of the WHO Pneumococcal Vaccines Technical Coordination Project.

## Introduction

*Streptococcus pneumoniae* causes substantial morbidity and mortality among children and adults globally.^1,2^ Currently, five pneumococcal conjugate vaccines (PCVs) are available for infant immunisation. The first PCV, licensed in 2000, covered seven serotypes. In 2007, WHO recommended that a seven-valent PCV (PCV7; Prevenar, Pfizer, New York, NY, USA) be included in national infant immunisation programmes for prevention of pneumococcal disease.^3^

Subsequently, in 2009–10, PCVs with additional serotypes were licensed, including a ten-valent vaccine (PCV10; Synflorix, GSK, Coleford, UK) covering PCV7 serotypes (4, 6B, 9V, 14, 18C, 19F, and 23F) plus serotypes 1, 5, and 7F, and a 13-valent vaccine (PCV13; Prevenar 13, Pfizer), which replaced PCV7 and included PCV10 serotypes plus serotypes 3, 6A, and 19A. About 10 years later, three additional PCVs became available: a second ten-valent vaccine (Pneumosil, Serum Institute of India, Pune, India) in 2020, which included the serotypes in Synflorix except with serotypes 4 and 18C replaced with 6A and 19A; a 15-valent PCV (PCV15; VAXNEUVANCE, Merck, Darmstadt, Germany) in 2022, which included the serotypes in PCV13 plus 22F and 33F; and a 20-valent PCV (PCV20; Prevenar20, Pfizer) in 2023, which included the serotypes in PCV15 plus 8, 10A, 11A, 12F, and 15BC. To date, PCVs have been introduced into 170 national infant immunisation programmes.^4^ Many countries also have an adult pneumococcal vaccine programme using pure polysaccharide or conjugate vaccines, although recommendations vary and uptake is generally low. ^5^

A 2013 global analysis of PCV7 impact in 16 countries for up to 7 years after PCV7 use found substantial declines in invasive pneumococcal disease (IPD) caused by PCV7 serotypes for both children and adults. Declines were partly offset by increases in incidence of non-vaccine-type IPD, including serotype 19A, to levels above those seen before PCV introduction and what would have been expected in the absence of pneumococcal conjugate vaccination, a phenomenon called serotype replacement.^6^ Subsequent wide use of PCV10 and PCV13 raised questions regarding their effect on the additional serotypes they include, cross-protection of PCV10 against serotype 6A, and non-vaccine-type replacement, especially for serotype 19A following PCV10 introduction. The degree of heterogeneity between countries is also of interest, including net indirect effects among adults, in whom some countries have seen a near return to pre-PCV incidence by 6–8 years following PCV10 or PCV13 introduction, whereas others have experienced sustained reductions in all-serotype IPD.^7,8^ Differences are possibly due to a combination of differential surveillance practices, pre-PCV serotype distributions, transmission dynamics, underlying population risk factors, PCV products, vaccine uptake, and immunisation schedule, among other factors.^9,10^ Multisite analyses using standardised methods might reduce some confounding and statistical heterogeneity by increasing sample size, another challenge for some countries. These analyses also allow examination of differences between settings and products. Previous multisite surveillance studies estimating the impact of PCV10 and PCV13 on IPD incidence aimed to address some of these issues but only included high-income countries in Europe, North America, and Australia and two or fewer PCV10 sites.^11–13^

A more globally representative evaluation of the long-term impact of PCV10 (Synflorix) and PCV13 in national infant immunisation programmes on IPD incidence in all ages is needed to address the remaining questions to inform vaccine policy and development of future vaccines. As part of the Pneumococcal Serotype Replacement and Distribution Estimation (PSERENADE) project,^5^ we sought all available published and unpublished population-based IPD surveillance data globally from 1991 to 2019 to estimate the change in vaccine-type, non-vaccine-type, and all-serotype IPD incidence by age group following introduction of PCV10 or PCV13 compared with the period preceding any PCV use.

## Methods

### Data collection and eligibility criteria

As described elsewhere,^5^ IPD surveillance sites in countries where PCV10 or PCV13 was universally recommended for all infants in the national infant immunisation schedule were identified by contacting known surveillance networks, a systematic literature review, and review of results from previous PCV reviews, conference abstracts, and citations. Invited sites had primary PCV series uptake of at least 50% at age 12 months in at least one year after PCV10 or PCV13 introduction and at least one complete year of data after PCV10 or PCV13 introduction, excluding the year of introduction.

In 2018–19, sites with annual serotype-specific IPD case data and population denominators in either aggregated or case-based format by age group were invited to contribute. IPD was defined as isolation of *S pneumoniae* from normally sterile fluids or detection using either *lytA*-based polymerase chain reaction or antigen testing in cerebrospinal fluid or pleural fluid. Sites meeting eligibility criteria in at least one age group with 2 years or more of data (to assess change), including sites without pre-PCV data, were included in analyses (appendix 2 pp 13–15, 18–21).

Investigators also described their site’s PCV programme and surveillance methods. This activity was reviewed by the Johns Hopkins Institutional Review Board and US Centers for Disease Control and Prevention, deemed research not involving human subjects and exempt from institutional review board oversite, and conducted consistent with applicable federal law and US Centers for Disease Control and Prevention policy.

Two PSERENADE coordinators evaluated each site for unanticipated patterns in IPD incidence rates to rule out influence from factors other than PCV introduction, including possible changes to the PCV programme or surveillance system and other concurrent events affecting risk of IPD.^5^ After discussion with site investigators, affected site–year–age group data that could not be accounted for were excluded (appendix 2 pp 13–15). For US sites, analyses were restricted to individuals hospitalised with IPD because of a large decline in outpatient surveillance after PCV7 introduction, consistent with another analysis,^14^ probably due to changes in outpatient blood culturing practices.^15–17^

The PCV7, PCV10, or PCV13 introduction year was the year in which the vaccine was recommended for all age-eligible children if introduced in January–September, or the following year if introduced in October–December. For data submitted in epidemiological years, the introduction year was defined accordingly. For sites with partial PCV introduction (eg, private market use before universal introduction), year of introduction was defined by the site. Year 0 is the year PCV10 or PCV13 was introduced. PCV10 refers to Synflorix (GSK) unless otherwise specified.

### Adjustments for missing data

Within site–year–age group strata, cases missing serotype information were excluded from analyses, other than for all-serotype IPD, if evidence suggested preferential selection for serotyping (eg, based on severity) or if fewer than 50% of individuals had reported serotypes (appendix 2 pp 13–15). To account for not-serotyped cases in vaccine-type, non-vaccine-type, and serotype-specific analyses, population denominators were adjusted by the proportion of cases serotyped, as opposed to reapportioning unknown serotypes, to weight sites in the model on the basis of the actual serotype data reported. Detailed methods to account for partly serotyped cases, including serogrouped only or undistinguished (eg, 6A or 6C), typed but serotype not identified, untypeable, two serotypes reported, and serotype pool, are described in appendix 2 (pp 7–8).

For individuals with unknown age, population denominators were adjusted by the proportion of individuals with known age for each year and age group. Minor changes were made to age group cutoffs when standardised age categories used for analyses were not available.

### Statistical analysis

Annual IPD incidence rate ratios (IRRs) comparing the period before universal introduction of any PCV (pre-PCV period) with each year after PCV10 or PCV13 introduction were estimated by age group (<5, 5–17, 18–49, 50–64, and ≥65 years) for all-serotype IPD and select vaccine-type and non-vaccine-type serotype groupings using a three-step process. In the first step, incidence rate curves were estimated over time by site using a Bayesian multi-level, mixed-effects Poisson regression using the MCMCglmm package in R version 4.4.0.^18^ The model included an offset for population denominator and random effects for all site-specific regression coefficients, allowing heterogeneity among sites’ incidence curves. The regression modelled pre-PCV incidence rates with a linear slope. A non-linear break was included 1 year before PCV7 (if used) and 1 year before PCV10 or PCV13 introduction for children younger than 5 years; for older individuals for whom indirect PCV effects are not immediate, non-linear breaks were included at the year of PCV introduction. For all ages, cubic spline knots were included for each site at years 1 and 3 after PCV7 (if used) and years 1 and 3 after PCV10 or PCV13 to allow flexibility in incidence rates over time. Site-specific modelled incidence rate curves were visually inspected for reasonableness of model fit and approved by site investigators with expertise in the site’s IPD surveillance.

In the second step, a counterfactual incidence rate (ie, expected incidence rate had PCV not been introduced) was estimated for sites with both pre-PCV and post-PCV data by extending the modelled pre-PCV incidence rate slope for 3 years after PCV introduction, then flattening to zero slope assuming any pre-PCV trends would stabilise thereafter. Site-specific modelled and counterfactual incidence rates were used to estimate site-specific annual IRRs in each post-PCV year, reported as the mean of the posterior distribution of rate ratios. Credibility intervals (Bayesian analogue of a 95% CI) were estimated using the 2·5th and 97·5th percentiles of the incidence rates’ posterior distribution (appendix 2 p 9).

In the third step, all site-weighted average IRRs were estimated for each year after PCV introduction for six strata defined by product (PCV10 and PCV13) and previous PCV7 impact (none, moderate, and substantial). PCV7 impact strata were defined by the modelled site-specific reduction in PCV7-type IPD among children younger than 5 years in the year before PCV10 or PCV13 introduction: no PCV7 impact (ie, PCV7 not used), substantial impact (PCV7-type IRR ≤0·05), or moderate impact (all others; appendix 2 pp 10–11). Modelled site-specific IRRs from the second step were used to estimate annual all site-weighted average IRRs for the six strata using linear mixed-effects regression, including a three-way interaction between year since PCV10 or PCV13 introduction, previous PCV7 impact, and product, with a random intercept for site. Year since PCV10 or PCV13 introduction was modelled with interior cubic spline knots at the observed 25th, 50th, and 75th percentiles to allow flexibility in the curve over time. The model was weighted by the inverse of posterior variances, which gives more weight to sites with smaller IRR standard errors.

To check parametric assumptions underlying the linear mixed-effects model fit simultaneously for all strata, we fit analogous models separately by stratum. For all site-weighted average IRRs estimated using data from a single stratum only, estimates for separate and simultaneous models were similar but CIs differed because uncertainty is assumed to be constant across strata in the simultaneous model but not in the separate models (appendix 2 pp 106–07). Note, however, that all site-weighted average IRRs were estimated in the simultaneous model including data from all PCV7 impact and product strata, instead of generating estimates for each stratum individually, in order to borrow evidence across strata and more robustly estimate uncertainty around all site-weighted average IRR estimates. All analyses were conducted in R version 4.4.0.

### Role of the funding source

The funder was provided with the opportunity to review and comment on the manuscript, but otherwise had no role in the study design, data collection, data analysis, data interpretation, writing of the manuscript, or the decision to submit for publication.

## Results

**Figure 1:**
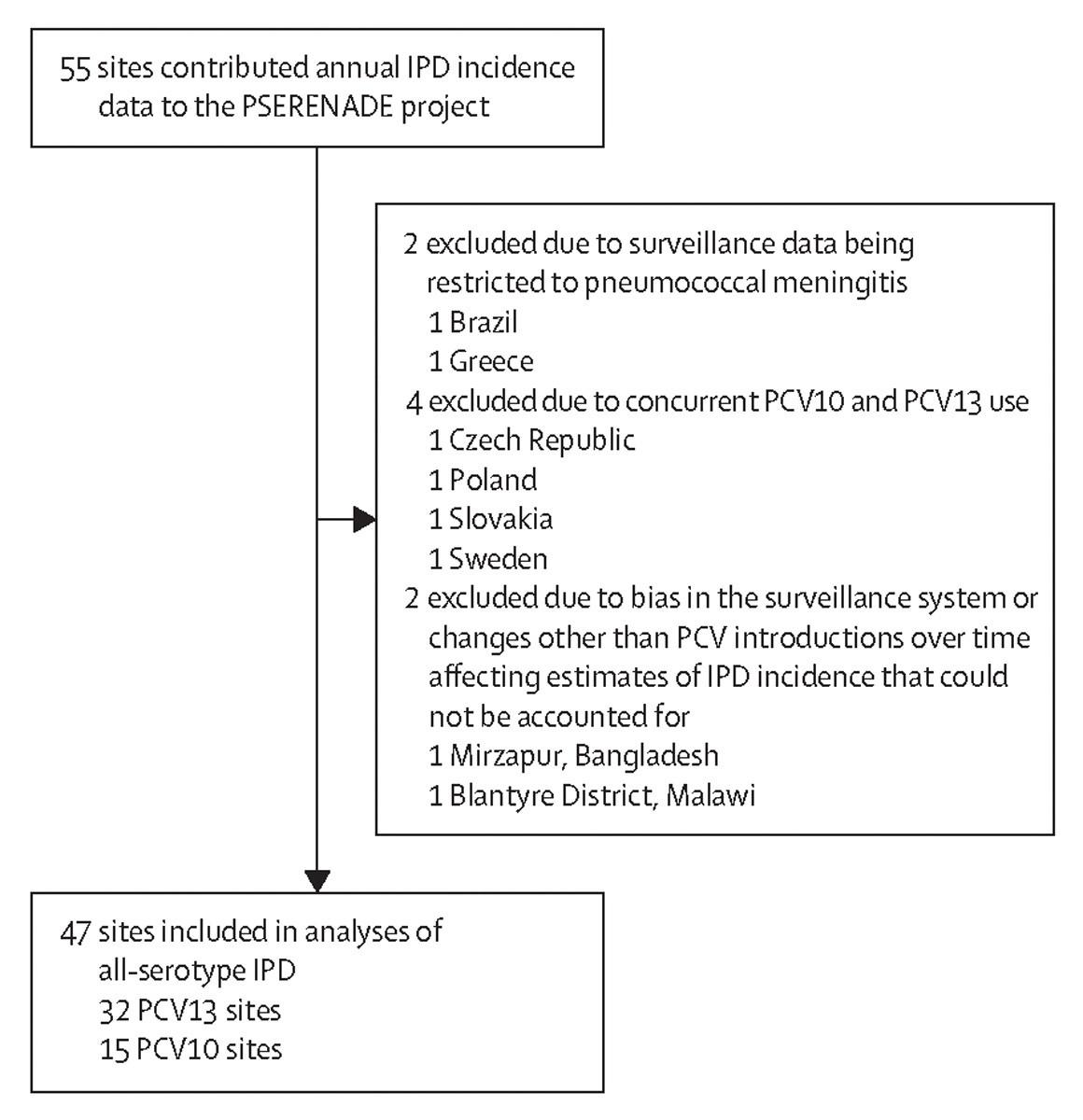
Sites that contributed IPD incidence data to the PSERENADE project and reasons for exclusion from present analysis. IPD=invasive pneumococcal disease. PCV=pneumococcal conjugate vaccine. PCV10=ten-valent PCV (Synflorix, GSK). PCV13=13-valent PCV (Prevenar 13, Pfizer).

30 countries contributed 527 692 IPD cases (median per site 2981, range 19–115 393) between 1991 and 2019 from 47 surveillance sites; 32 (68%) sites had both pre-PCV and post-PCV data (table, appendix 2 pp 18–21). Eight ineligible sites were excluded (figure 1, appendix 2 pp 13–15). Although all global regions were represented, Latin America and the Caribbean and Northern Africa and Western Asia had only two sites each (table). The European region (18 sites) contributed most cases (n=331 026, 63%), whereas Asia (four sites) contributed fewest (n=3185, <1%). Few sites were from low-income and middle-income countries (two upper-middle income, five lower-middle income, and one low income) or countries eligible for support from Gavi, the Vaccine Alliance (five sites).

PCV13 sites contributed more data (32 sites, 488 758 cases) than PCV10 sites (15 sites, 46 386 cases) and more had previously used PCV7 (26 [81%] *vs* six [40%]; table). Most sites’ PCV schedules included a booster dose (30 [94%] PCV13 sites and 11 [73%] PCV10 sites). 12 (38%) PCV13 and three (20%) PCV10 sites introduced PCV10 or PCV13 with a catch-up campaign. Median PCV10 or PCV13 uptake was high (91%, range across sites 55–99); three sites had median uptake of less than 70% (appendix 2 p 26). Although 38 (81%) sites recommended pure polysaccharide or conjugate vaccines for at-risk or all age-eligible adults, where data were available, vaccine uptake was low (data not shown). Before PCV introduction among children younger than 5 years, the median proportion of IPD due to PCV10 types at PCV10 sites was 77% (range 67–90) and the median proportion due to PCV13 types at PCV13 sites was 91% (81–100). Among 44 sites included in vaccine-type, non-vaccine-type, and serotype-specific analyses, the median proportion of IPD cases serotyped was 89% (68–99).

**Figure 2:**
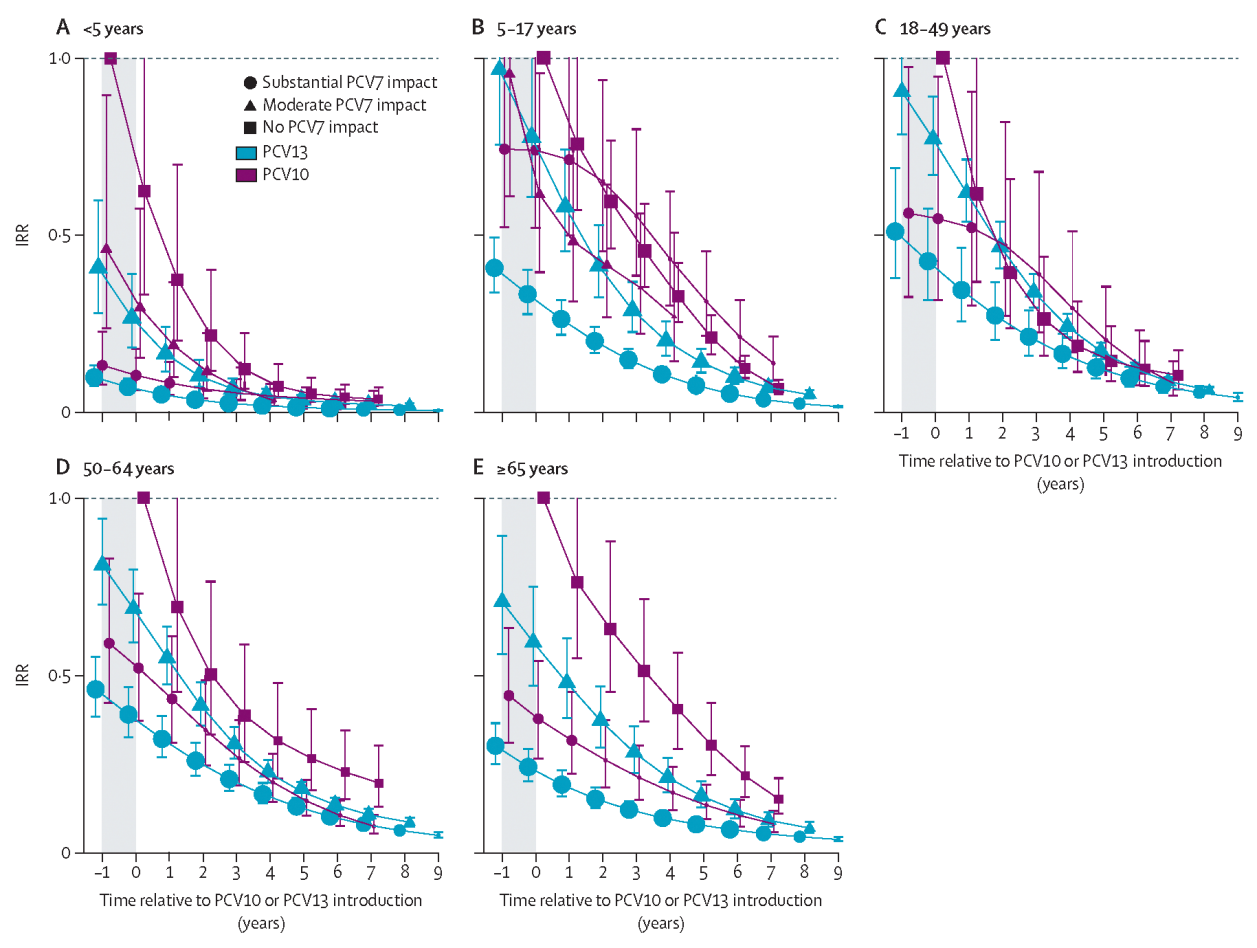
All-site weighted average IRRs for PCV10-type IPD by age group. Site-specific annual incidence rates of IPD following PCV10 or PCV13 introduction were compared to the site’s average incidence rate before PCV introduction to compute site-specific IRRs; all-site-weighted average IRRs are an average of the sites’ IRRs. PCV10 types include serotypes 1, 4, 5, 6B, 7F, 9V, 14, 18C, 19F, and 23F. Cross-protection from serotype 6A was not included. The shaded area indicates the year of PCV10 or PCV13 rollout. Estimates at year 0 indicate the change in incidence after the first year of PCV10 or PCV13 use. In individuals aged 5 years or older, the model that best fit the data was one that shifted the first year of estimated effect to the year after the first year of PCV introduction, not the year of PCV rollout as for children younger than 5 years. For sites with moderate or substantial PCV7 impact, the first year of estimated effect occurred at PCV7 introduction (not shown). For sites with no PCV7 impact, the first year of estimated effect can be seen for the first point estimate, indicating the pre-PCV period (ie, IRR=1), which occurs at year −1 for children younger than 5 years and at year 0 for all other age groups. IPD=invasive pneumococcal disease. IRR=incidence rate ratio. PCV=pneumococcal conjugate vaccine. PCV7=seven-valent PCV (Prevenar, Pfizer). PCV10=ten-valent PCV (Synflorix, GSK). PCV13=13-valent PCV (Prevenar 13, Pfizer).

**Figure 3:**
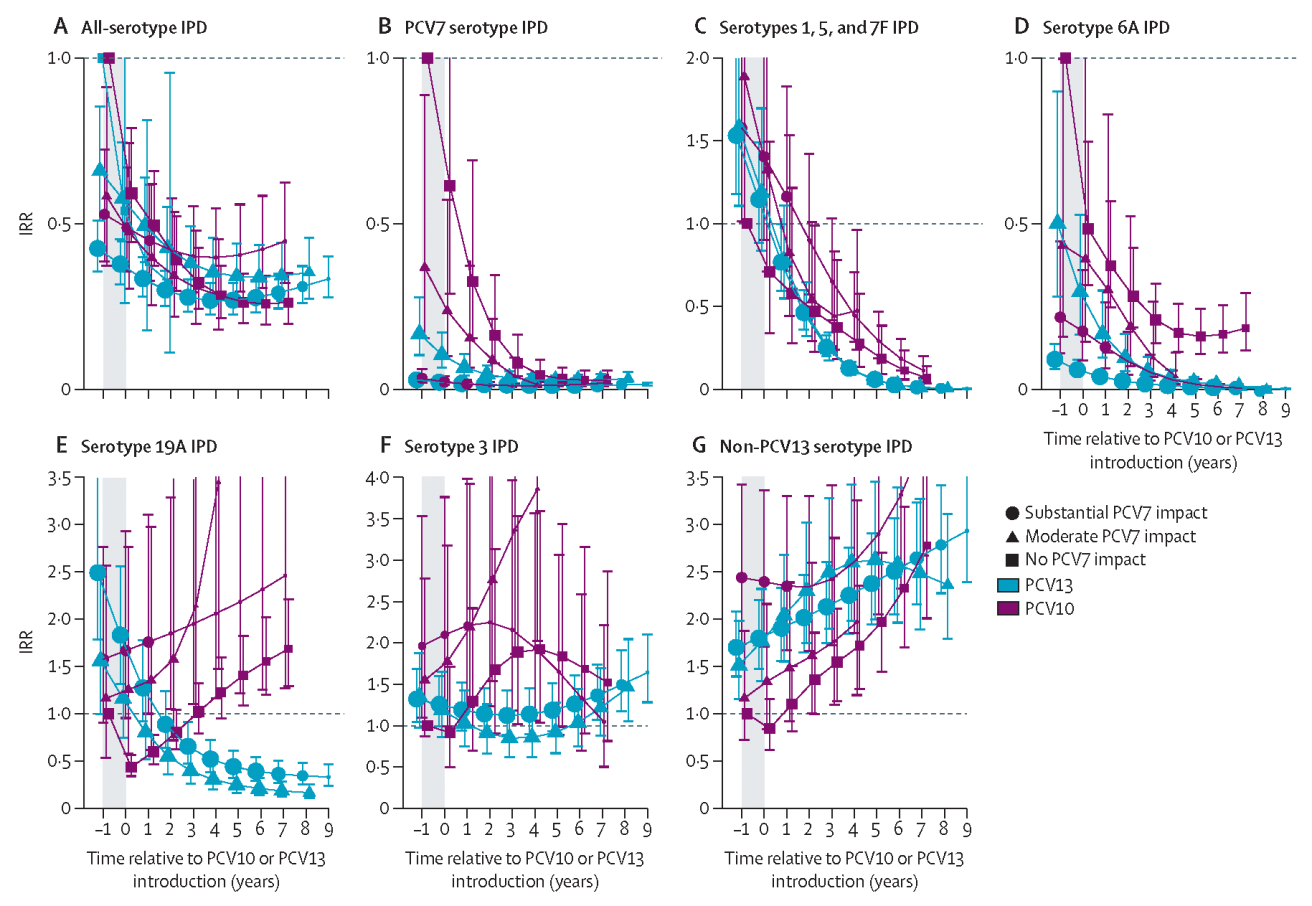
All-site weighted average IRRs for IPD due to any serotype or specific serotypes or serotype groups for children younger than 5 years. Site-specific annual incidence rates of IPD following PCV10 or PCV13 introduction were compared to the site’s average incidence rate before PCV introduction to compute site-specific IRRs; all-site weighted average IRRs are an average of the sites’ IRRs. The y-axis scale changes between figures. PCV13 types include serotypes 1, 3, 4, 5, 6A, 6B, 7F, 9V, 14, 18C, 19A, 19F, and 23F. The shaded area indicates the year of PCV10 or PCV13 rollout. Estimates at year 0 indicate the change in incidence after the first year of PCV10 or PCV13 use. In individuals aged 65 years or older, the model that best fit the data was one that shifted the first year of estimated effect to the year after the first year of PCV introduction, not the year of PCV rollout as for children younger than 5 years. For sites with moderate or substantial PCV7 impact, the first year of estimated effect occurred at PCV7 introduction (not shown). For sites with no PCV7 impact, the first year of estimated effect can be seen for the first point estimate, indicating the pre-PCV period (ie, IRR=1), which occurs at year −1 for children younger than 5 years and at year 0 for all other age groups. Similar figures for age groups 5–17 years, 18–49 years, and 50–64 years are in appendix 2 (pp 23–25). IPD=invasive pneumococcal disease. IRR=incidence rate ratio. PCV=pneumococcal conjugate vaccine. PCV7=seven-valent PCV (Prevenar, Pfizer). PCV10=ten-valent PCV (Synflorix, GSK). PCV13=13-valent PCV (Prevenar 13, Pfizer).

**Figure 4:**
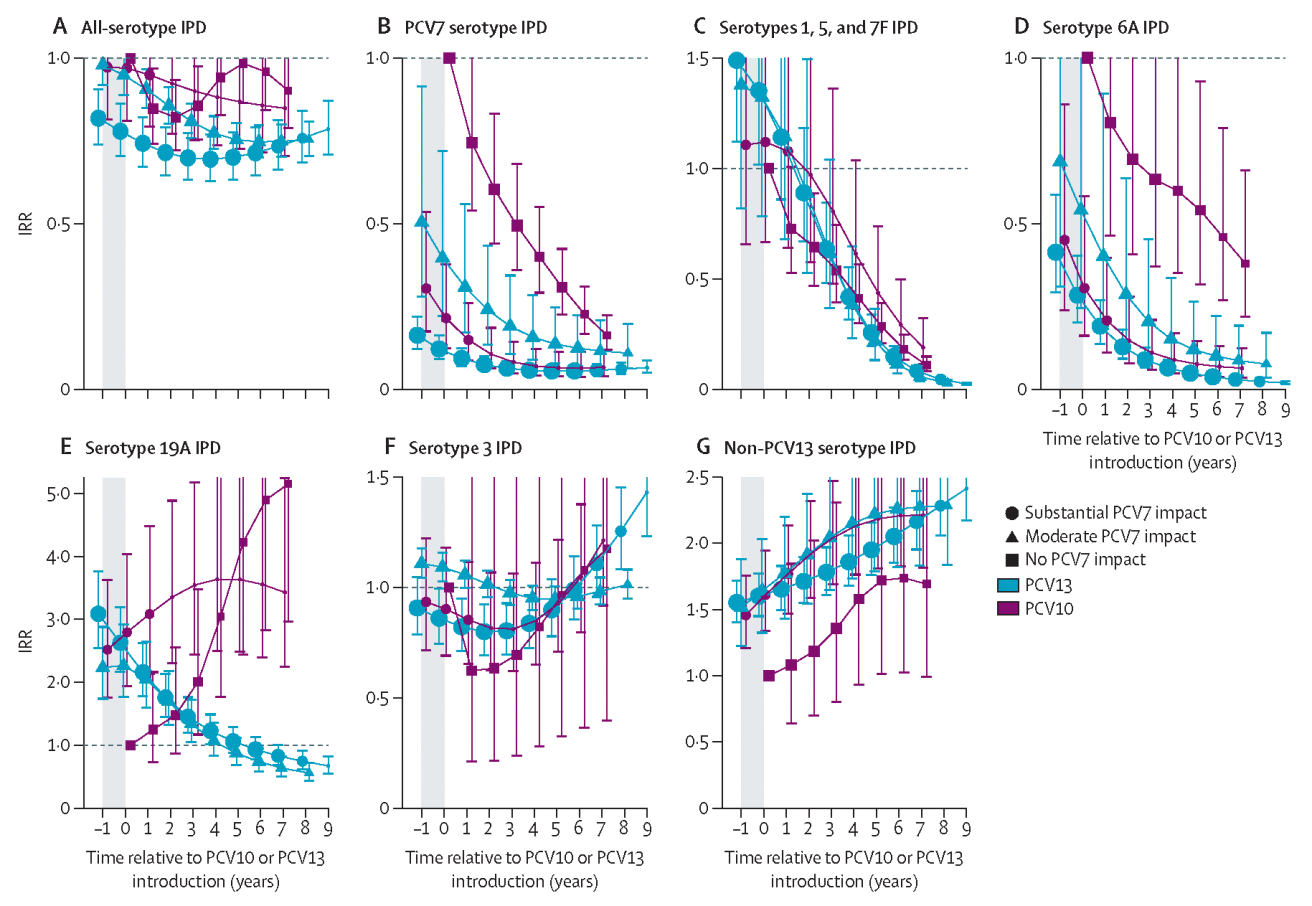
All-site weighted average IRRs for IPD due to any serotype or specific serotypes or serotype groups for adults aged 65 years or older. Site-specific annual incidence rates of IPD following PCV10 or PCV13 introduction were compared to the site’s average incidence rate before PCV introduction to compute site-specific IRRs; all-site weighted average IRRs are an average of the sites’ IRRs. The y-axis scale changes between figures. PCV13 types include serotypes 1, 3, 4, 5, 6A, 6B, 7F, 9V, 14, 18C, 19A, 19F, and 23F. The shaded area indicates the year of PCV10 or PCV13 rollout. Estimates at year 0 indicate the change in incidence after the first year of PCV10 or PCV13 use. In individuals aged 65 years or older, the model that best fit the data was one that shifted the first year of estimated effect to the year after the first year of PCV introduction, not the year of PCV rollout as for children younger than 5 years. For sites with moderate or substantial PCV7 impact, the first year of estimated effect occurred at PCV7 introduction (not shown). For sites with no PCV7 impact, the first year of estimated effect can be seen for the first point estimate, indicating the pre-PCV period (ie, IRR=1), which occurs at year −1 for children younger than 5 years and at year 0 for all other age groups. Similar figures for age groups 5–17 years, 18–49 years, and 50–64 years are in appendix 2 (pp 23–25). IPD=invasive pneumococcal disease. IRR=incidence rate ratio. PCV=pneumococcal conjugate vaccine. PCV7=seven-valent PCV (Prevenar, Pfizer). PCV10=ten-valent PCV (Synflorix, GSK). PCV13=13-valent PCV (Prevenar 13, Pfizer).

Declines in PCV7 and PCV10 types appeared to plateau by year 6 after introduction (ie, 7 years of PCV10 or PCV13 use) in children younger than 5 years (figures 2A, 3B), so impact is described at year 6 after introduction for all analyses unless otherwise specified. However, the declines and increases observed for other serotypes and age groups did not plateau by year 6 and continued their trends throughout the observation period, which extended to 7–9 years after introduction for most strata (figures 2–4).

Analyses for children younger than 5 years included 9011 cases (median per site 348 [range 22–2599]) from 14 PCV10 sites and 59 977 cases (median per site 622 [44–11 594]) from 31 PCV13 sites (table). Two PCV10 sites and ten PCV13 sites had post-PCV data only (appendix 2 p 16); of the remaining sites, four PCV10 sites and 12 PCV13 sites had substantial previous PCV7 impact, two PCV10 sites and six PCV13 sites had moderate PCV7 impact, and six PCV10 sites and two PCV13 sites had no PCV7 impact. The two PCV13 sites with no previous PCV7 use were only eligible for all-IPD analyses due to the low proportion of cases serotyped. Vaccine-type IPD in children younger than 5 years was nearly eliminated in all strata by 6 years after PCV10 or PCV13 introduction. Overall, at both PCV10 and PCV13 sites, IPD caused by PCV10 serotypes declined by 96–99% by year 6 after introduction (figure 2A), and serotype 6A IPD declined by 83–99% (figure 3D). IPD due to PCV13 serotypes declined more at PCV13 sites (94–95%) than at PCV10 sites (80–88%; appendix 2 p 31). Serotype 19A IPD declined at PCV13 sites (61–79%) and increased at least 1·5-fold at PCV10 sites (figure 3E). No consistent trends in serotype 3 IPD were observed for either product (IQR of site-specific modelled IRRs 0·76–2·1 at year 6; figure 3F, appendix 2 p 35). Serotype 6C IPD case counts were insufficient to robustly model pre-PCV incidence rates for most sites; however, serotype 6C incidence rates appeared to trend upward following PCV7 and PCV10 introduction and downward following PCV13 introduction (appendix 2 pp 27–28). Effect on all-serotype IPD ranged from 58% (95% CI 41–70) to 74% (66–81), with some heterogeneity across sites, but no clear difference by product (figure 3A, appendix 2 p 33). IPD due to non-vaccine serotypes increased following PCV7 introduction (figure 3C, E–G), which continued following introduction of PCV10 or PCV13, ranging from 2·3-fold (95% CI 1·7–3·2) to 3·3-fold (2·3–4·7) across both products (figure 3G). Some heterogeneity was observed across sites in duration of non-PCV13-type IPD increases, with IRRs continuing to increase at some sites but levelling off at others, and in their magnitude at year 6 (IQR 1·7–2·7; appendix 2 p 36). Results were similar after excluding sites without pre-PCV data (appendix 2 p 105).

Analyses of adults aged 65 years or older included 12 PCV10 sites (17 142 cases; median per site 503 [range ten to 5368]) and 26 PCV13 sites (192 442 cases; median per site 2641 [ten to 49 475]). Vaccine-type IPD IRRs for adults aged 65 years or older generally followed trends similar to those for children younger than 5 years, but with a slower rate of decline (figure 2). By 6 years after PCV10 or PCV13 introduction, declines were similar between PCV10 and PCV13 sites for PCV10-type IPD (78–93%; figure 2E) and IPD due to serotype 6A (54–96%; figure 4D). PCV13-type IPD declined more at PCV13 sites (71–77%) than at PCV10 sites (28–66%; appendix 2 p 31). Serotype 19A IPD increased following PCV7 and PCV10 introduction and declined after PCV13 introduction; however, the declines in serotype 19A IPD at PCV13 sites were much smaller for adults (7–26% by year 6) than for children younger than 5 years (61–79%), and increases in serotype 19A IPD at PCV10 sites were on average greater for adults (3·6–4·9-fold) than for children (1·6–2·3-fold) among strata with data for both age groups (figure 4E, appendix 2 p 34). No consistent trends were observed in serotype 3 IPD for either product, which increased, decreased, or had no meaningful change depending on the site (IQR of year 6 site-specific modelled IRRs 0·8–1·0; figure 4F, appendix 2 p 35). Similar to children, IPD due to non-PCV13 serotypes increased 1·7–2·3-fold across strata (figure 4G), ranging from 1·2-fold to 2·8-fold across sites (appendix 2 p 36); increases appeared to be continuing for some, although not all, sites. Net effect on all-serotype IPD was smaller for adults than for children. Declines at PCV13 sites were 25% (95% CI 21–36) to 29% (20–30); evidence was less clear for PCV10 sites but appeared lower (declines were 4–14% with 95% CIs overlapping no effect), but sample size and other site differences prevent attribution to product (figure 4A). Additionally, heterogeneity between sites within strata ranged from sustained declines to some nearly returning to pre-PCV rates (IQR of year 6 site-specific modelled IRRs 5–27% decline; appendix 2 p 33), which was not explained by adult pneumococcal vaccine recommen dations (appendix 2 p 43).

Analyses of other ages included 13 PCV10 sites (2511 cases) and 28 PCV13 sites (21 626 cases) for children aged 5–17 years, 14 PCV10 sites (8282 cases) and 27 PCV13 sites (116 510 cases) for adults aged 18–49 years, and 12 PCV10 sites (9440 cases) and 26 PCV13 sites (98 203 cases) for adults aged 50–64 years (table). Patterns for vaccine-type, non-vaccine-type, and serotype-specific IPD for these age groups were generally similar to those for adults aged 65 years or older (figure 2B–D, appendix 2 pp 23–25). However, declines in all-serotype IPD reduced with increasing age: 42–65% across strata for ages 5–17 years, 40–62% for ages 18–49 years, and 10–30% for ages 50–64 years (appendix 2 pp 23–25). Some serotype-specific analyses (eg, serotype 6A) with low annual numbers produced unusual patterns, probably due to model instability (appendix 2 pp 23–25).

## Discussion

The PSERENADE project compiled the largest and most globally diverse IPD surveillance database including data up to 2019 (ie, before the COVID-19 pandemic) from countries with high PCV10 and PCV13 uptake in childhood immunisation programmes. We estimated that 6 years after PCV10 or PCV13 introduction, all-IPD incidence in children younger than 5 years declined by 58–74% relative to the pre-PCV period. Vaccine-type IPD rapidly declined in children younger than 5 years, soon followed by declines in older children and adults due to indirect effects. Vaccine-type declines were partly offset by consistent increases in non-vaccine-type IPD in all ages, probably resulting from serotype replacement. The net effect on all-serotype IPD decreased with age, with declines of 4–29% in adults aged 65 years or older, probably due to the smaller proportion of IPD in older adults than in children caused by vaccine serotypes before PCV introduction.^21^ Despite this finding, similar or even greater absolute numbers of cases might have been prevented in adults compared with children due to the much larger adult population size.^22^ The only vaccine-type IPD not observed to decline was PCV13’s serotype 3, which also did not increase at PCV10 sites like other non-vaccine types. Cross-protection was observed against serotype 6A at PCV10 sites and possibly serotype 6C at PCV13 sites. Countries with extensive PCV7 use experienced further declines in all-serotype IPD after switching to PCV10 or PCV13, including reversing PCV7-driven replacement disease for the additional serotypes they covered. Declines were similar between PCV10 and PCV13 sites for their respective vaccine types, as were the approximately two-fold increases in non-vaccine types 6 years after introduction. Although declines in all-serotype IPD were similar between PCV10 and PCV13 sites in children, PCV10 sites could expect additional impact with PCV13 by reducing serotype 19A.

Other multicountry studies estimating PCV impact were restricted to high-income countries in Europe, North America, and Australia and included few PCV10 sites but were broadly consistent with our findings.^11–13^ Despite having broader representation, our analysis included few PCV10 countries (ten) and 3+0 schedules (Australia, Kenya, Fiji, and The Gambia), hampering the ability to assess the effect of a booster dose. However, trends in all-serotype IPD, vaccine-type IPD, and non-vaccine-type IPD were similar among sites with and without a booster dose. Previous PSERENADE analyses of serotype 1 showed delayed effect in some countries, but after 5–6 years, serotype 1 was nearly eliminated in all ages regardless of schedule.^23^ Data from two African meningitis belt countries (Ghana and Burkina Faso) using PCV13 with a 3+0 schedule could not be included in this project, but published data do not indicate a clear difference in impact compared with our findings.^24–26^ However, Australia observed breakthrough cases in the second year of life, suggesting a shorter duration of protection without a booster dose, leading them to switch from a 3+0 to a 3+1 schedule in 2018.^27^ High-burden settings could require higher uptake, a booster dose, or longer-term use to maximise the impact on IPD in all ages.

The absence of PCV13 or PCV10 effects on serotype 3 IPD incidence has been observed previously, including site analyses evaluating population-level impact.^7,8,28–33^ Post-licensure case–control studies using either the indirect cohort (Broome) method^34^ or healthy controls found PCV13 effectiveness against serotype 3 IPD to be approximately 50–65%.^35–37^ These observational methodologies might overestimate direct vaccine effectiveness due to so-called healthy vaccinee bias in studies that compare with healthy controls, and due to greater relative increases in non-vaccine-type IPD among vaccinated children in indirect cohort studies. For example, if non-vaccine-type replacement is 1·5–2 times higher in vaccinated compared with unvaccinated children and the true vaccine effectiveness against serotype 3 is 0%, then the indirect cohort method produces biased estimates of vaccine effectiveness of between 50% and 67%, similar to the estimates of indirect cohort studies.^35,38^ Discrepant findings between impact and effectiveness designs could also be explained by decreases in serotype 3 IPD among vaccinated children that are offset by large serotype 3 increases among unvaccinated children, but this is unlikely given that large serotype 3 replacement was not observed at PCV10 sites and vaccine uptake was high at sites in this study. Because serotype 3 IPD did not increase similarly to other non-vaccine types at PCV13 sites, it has been hypothesised that protection is low rather than none; however, serotype 3 at PCV10 sites also did not increase like other non-vaccine types, and the similar trends between PCV10 and PCV13 sites suggest that PCV13 does not protect against serotype 3 and that propensity for replacement disease differs between serotypes. A clinical trial of an investigational 11-valent PCV observed low non-statistically significant efficacy against acute otitis media caused by serotype 3 after the primary series and moderate non-statistically significant negative efficacy after the booster dose,^39,40^ suggesting possible hyporesponsiveness from priming doses, but this was not supported by immunogenicity data.^41–43^ Absence of indirect effects on serotype 3 is consistent with a PCV13 trial evaluating serotype 3 carriage in children, which found no effect in vaccinated children, indicating that PCV13 would not interrupt serotype 3 transmission.^44^ Additionally, PCVs containing serotype 3 have not shown sufficient antibody responses expected to provide protection against the serotype 3 capsule.^45^ The result is that serotype 3 is now the top IPD serotype at PCV13 sites and second most common after serotype 19A at PCV10 sites,^46^ indicating that new strategies to address serotype 3 IPD are needed.

Serotype 19A IPD substantially increased following PCV7 introduction, a change that reversed after PCV13 introduction, bringing 19A incidence back to baseline after about 3 years. Although declines continued throughout our observation period 8–9 years after PCV13 introduction, reductions were smaller than for other vaccine types (eg, in children younger than 5 years, serotype 19A declined by 61–79% *vs* >85% for serotype 1, serotype 5, and serotype 7F). This might be because serotype 19A had greater replacement with PCV7 than other non-vaccine types (eg, two-fold increases versus 1·5-fold). At PCV10 sites, the magnitude of replacement was generally similar between serotype 19A and other non-vaccine types among children, approximately two-fold at 6 years after introduction, but in adults serotype 19A increased more than other non-vaccine types (approximately four-fold *vs* two-fold).

Serotype 6A declined substantially at PCV10 sites, including in adults, suggesting that cross-protection from serotype 6B in PCV10 reduced transmission. Although clinical trials found similar declines in serotype 6A carriage for PCV10 and PCV13,^47^ a longitudinal surveillance study in Kilifi, Kenya, did not observe reductions in serotype 6A carriage among children younger than 5 years after 5 years of PCV10 use.^48^ Our study had few serotype 6C IPD cases, but PCV13 site data suggested cross-protection from serotype 6A, and the PSERENADE analysis estimating the serotype distribution at least 5 years after PCV10 or PCV13 introduction estimated more serotype 6C at PCV10 sites than at PCV13 sites;^46^ monitoring should continue.

Our estimated doubling of IPD due to non-vaccine types by year 6 for both PCV10 and PCV13 sites is broadly consistent with other multisite studies.^11–13^ Annual increases in incidence rates of IPD due to non-vaccine types appeared roughly constant over the 8-year period after PCV10 or PCV13 introduction, but it is unclear how long increases will continue. IPD is a function of both serotype invasiveness and host vulnerability. Because non-vaccine types have generally been less invasive than vaccine types, and non-vaccine types accounted for a smaller proportion of IPD in children before PCV introduction, the net impact of PCV10 or PCV13 is likely to remain large for children.^49,50^ However, because vulnerability among adults increases with age, older adults might eventually become susceptible to less invasive non-vaccine types, leading to no net impact on adult IPD from infant PCV programmes, as has been modelled for at least one country.^51^ Whether IPD would return to pre-PCV levels—and if so, how long it would take—was not estimated in this analysis, but would depend on several factors, including the pre-PCV serotype distribution, the age distribution of the population, and the underlying susceptibility of the population, which change over time and differ between countries and age groups.^11^

Although sites using the same product had similar trends, magnitudes in IPD changes following PCV10 or PCV13 introduction differed between sites, as illustrated by the three PCV7-impact strata for a given PCV product: because effects of previous PCV7 use should eventually wash out, these strata should converge but often did not, suggesting residual differences between sites beyond PCV product and age. This finding is despite excluding data with known biases and accounting for trends occurring during the pre-PCV period. Sites might differ in surveillance and clinical practices; PCV schedule, catch-up, and uptake; adult immunisation recommendations and uptake; transmission dynamics; prevalence and degree of changes in population risk factors (including underlying comorbidities); pathogen evolution; and pre-PCV IPD and carriage serotype distributions.^9–11,14,52^

This study highlights the value of having many countries conducting high-quality, population-based IPD surveillance and of collaborating to increase power and enable comparisons not possible in settings using a single vaccination programme. Sharing well characterised datasets enabled the evaluation of data quality and potential biases, creation of standardised comparisons, and performance of detailed stratified analyses that would not have been possible using data abstracted from published literature or from single surveillance systems because few are large enough to estimate robust serotype-specific effects. Although we estimated relative instead of absolute changes in incidence—because absolute changes would have varied depending on pre-PCV IPD incidence, which differed between sites due to differences in disease burden, surveillance systems, detection methods, etc—our analytical approach improved upon previous methods by accounting for uncertainty both within and between sites^6,12^ and attaining greater representativeness by incorporating data from sites without pre-PCV data and having no minimum sample size requirements.^6,12,13^

Nonetheless, this analysis has limitations. Although all regions were represented, there was data paucity from Asia, Latin America, sub-Saharan Africa, high-burden countries, and countries using a 3+0 schedule, which restricts the generalisability of our results. This prevented a robust assessment of regional variation and impact by schedule, particularly of a booster dose. Most long-term data (ie, ≥7 years after PCV10 or PCV13 introduction) came from high-income countries that introduced PCVs earliest, but early trends of all sites influence the trajectory and thus affect estimates of later years. Most sites had few years of pre-PCV surveillance to accurately establish trends, and we assumed any such trends were limited to 3 years after PCV use, which might have been too long or too short for some sites. We did not have sufficient evidence to assess changes in blood culturing practices among outpatients for non-US sites, but the proportion of IPD from outpatients was low in non-US countries so this is unlikely to have meaningfully influenced the results. As such changes were known for the USA, for those sites, outpatients were excluded from analyses. We were otherwise unable to control for several potential confounding factors that are likely to have contributed to observed heterogeneity between sites, including pre-PCV serotype distribution, prevalence of comorbidities including HIV, PCV schedule, catch-up programmes, and adult immunisation. Although the majority of sites had adult pneumococcal vaccine programmes that could influence the results, we found no differences in effect between sites with adult pneumococcal vaccine recommendations versus those without, probably in part due to low uptake. Furthermore, comparison of impact among adults aged 65 years or older between PCV10 and PCV13 sites was difficult because estimates for PCV13 sites were for strata that used PCV7 first, whereas few PCV10 sites with adult data had used PCV7. Although we were unable to test the assumption that the serotype distribution of cases not serotyped was the same as those serotyped within covariate strata, the proportion of redistributed cases was small. Finally, due to unknown sensitivity of case detection, we did not estimate absolute incidence rates.

This study, evaluating the impact of long-term use of PCV10 or PCV13, highlights the degree to which infant PCV immunisation programmes have reduced IPD in all ages and the power of multisite collaboration to elucidate evidence for individual serotypes and compare products. The study showed that the degree and speed with which PCVs reduce vaccine-type IPD vary by serotype, ranging from rapid (PCV7 serotypes) to taking many years for some (serotype 19A) and no effect (serotype 3). This study included IPD up to 2019, just before the COVID-19 pandemic, which disrupted IPD surveillance systems, vaccination programmes, and disease trends for several years.^51^ Additionally, some countries have since introduced new higher-valency PCV products and the UK switched to a 1+1 schedule. Continued surveillance and multicountry evaluations will be essential for assessing the effect of alternative schedules and the new higher-valency PCVs that target many of the remaining serotypes that historically were much less common than PCV10 or PCV13 serotypes but are now on the rise.

## Supplementary Material

Supplement 1

Supplement 2

## Figures and Tables

**Table:** Characteristics of surveillance sites and surveillance data included in analyses

	Age <5 years		Age 5–17 years		Age 18–49 years		Age 50–64 years		Age ≥65 years		All ages*		
	PCV10	PCV13	PCV10	PCV13	PCV10	PCV13	PCV10	PCV13	PCV10	PCV13	PCV10	PCV13	Total

Total sites	14	31	3	28	4	27	12	26	2	26	15	32	47
Sites eligible for serotype analysis	12	30	1	28	2	26	11	25	1	25	12	30	44
Region†													
Asia	0	3 (10%)	0	2 (7%)	0	3 (11%)	0	3 (12%)	0	3 (12%)	0	4 (13%)	4 (9%)
Europe	4 (29%)	13 (42%)	4 (31%)	13 (45%)	5 (35%)	12 (44%)	5 (42%)	12 (45%)	5 (42%)	12 (45%)	5 (33%)	13 (41%)	18 (38%)
Latin America and the Caribbean	2 (14%)	0	2 (15%)	0	2 (14%)	0	2 (17%)	0	2 (17%)	0	2 (13%)	0	2 (4%)
North America	2 (14%)	10 (32%)	2 (15%)	8 (29%)	2 (14%)	8 (30%)	2 (17%)	8 (31%)	2 (17%)	8 (31%)	2 (13%)	10 (31%)	12 (25%)
Northern Africa and Western Asia	0	2 (7%)	0	2 (7%)	0	2 (7%)	0	1 (4%)	0	1 (4%)	0	2 (5%)	2 (4%)
Oceania	3 (21%)	1 (3%)	2 (15%)	1 (4%)	2 (14%)	1 (4%)	2 (17%)	1 (4%)	2 (17%)	1 (4%)	3 (20%)	1 (3%)	4 (9%)
Sub-Saharan Africa	3 (21%)	2 (7%)	3 (23%)	2 (7%)	3 (21%)	1 (4%)	1 (8%)	1 (4%)	1 (8%)	1 (3%)	3 (20%)	2 (5%)	5 (11%)
World Bank income level‡													
High	10 (71%)	27 (87%)	0 (77%)	25 (89%)	1 (79%)	25 (93%)	11 (92%)	25 (95%)	1 (92%)	25 (95%)	11 (73%)	28 (88%)	39 (83%)
Upper middle	1 (7%)	1 (3%)	0	1 (4%)	0	1 (4%)	0	1 (4%)	0	1 (4%)	1 (7%)	1 (3%)	2 (4%)
Lower middle	3 (21%)	2 (7%)	3 (23%)	1 (4%)	3 (21%)	1 (4%)	1 (8%)	0	1 (8%)	0	3 (20%)	2 (5%)	5 (11%)
Low	0	1 (3%)	0	1 (4%)	0	0	0	0	0	0	0	1 (3%)	1 (2%)
Gavi status§	3 (21%)	2 (7%)	3 (23%)	1 (4%)	3 (21%)	0	1 (8%)	0	1 (8%)	0	3 (20%)	2 (5%)	5 (11%)
Pre-PCV *Streptococcus pneumoniae* disease burden in children <5 years (2000)¶													
Low	8 (57%)	27 (87%)	8 (52%)	25 (89%)	8 (57%)	25 (93%)	8 (57%)	25 (95%)	8 (57%)	25 (95%)	8 (53%)	28 (88%)	35 (77%)
Medium	2 (14%)	0 (0%)	2 (15%)	0 (0%)	3 (21%)	0 (0%)	3 (25%)	0 (0%)	3 (25%)	0 (0%)	3 (20%)	0 (0%)	3 (5%)
High	4 (29%)	4 (13%)	3 (23%)	3 (11%)	3 (21%)	2 (7%)	1 (8%)	1 (4%)	1 (8%)	1 (4%)	4 (27%)	4 (13%)	8 (17%)
Schedule													
3+0	4 (29%)	2 (7%)	3 (23%)	2 (7%)	3 (21%)	1 (4%)	1 (8%)	1 (4%)	1 (8%)	1 (4%)	4 (27%)	2 (5%)	5 (13%)
2+1	6 (43%)	14 (45%)	5 (45%)	13 (45%)	5 (43%)	12 (44%)	5 (50%)	11 (42%)	5 (50%)	11 (42%)	5 (40%)	14 (44%)	20 (43%)
3+1	3 (21%)	8 (25%)	3 (23%)	5 (21%)	3 (21%)	7 (25%)	3 (25%)	7 (27%)	3 (25%)	7 (27%)	3 (20%)	9 (28%)	12 (25%)
2+1 and 3+1	1 (7%)	7 (23%)	1 (8%)	7 (25%)	2 (14%)	7 (25%)	2 (17%)	7 (27%)	2 (17%)	7 (27%)	2 (13%)	7 (22%)	9 (19%)
PCV10 or PCV13 catch-up	3 (21%)	12 (39%)	3 (23%)	9 (32%)	3 (21%)	9 (33%)	2 (17%)	9 (35%)	2 (17%)	9 (35%)	3 (20%)	12 (38%)	15 (32%)
Previous PCV7 use	6 (43%)	25 (84%)	5 (45%)	25 (89%)	5 (35%)	22 (82%)	5 (42%)	22 (85%)	5 (42%)	22 (85%)	5 (40%)	25 (81%)	32 (58%)
Setting with adult vaccine recommendation‖													
PPV23	10 (71%)	27 (87%)	0 (77%)	25 (89%)	0 (71%)	25 (93%)	10 (83%)	25 (95%)	0 (83%)	25 (95%)	10 (57%)	28 (88%)	38 (81%)
PCV13	8 (57%)	27 (87%)	8 (52%)	25 (89%)	8 (57%)	24 (89%)	8 (57%)	24 (92%)	8 (57%)	24 (92%)	8 (53%)	27 (84%)	35 (75%)
Number of surveillance years per site													
Total	12 (3–24)	14 (5–27)	2 (5–24)	14 (5–27)	0 (4–24)	14 (2–27)	11 (4–24)	14 (2–27)	1 (4–24)	14 (2–27)	11 (3–24)	14 (2–27)	12 (2–27)
Pre-PCV period	5 (1–15)	4 (1–10)	5 (1–15)	3 (1–10)	5 (1–15)	4 (1–10)	5 (2–15)	3 (1–10)	5 (2–15)	3 (1–10)	5 (1–15)	4 (1–10)	5 (1–15)
PCV7 period	5 (2–9)	5 (1–10)	5 (2–9)	4 (1–10)	5 (2–9)	5 (1–10)	5 (2–9)	5 (1–10)	5 (2–9)	5 (1–10)	5 (2–9)	5 (1–10)	5 (1–10)
PCV10 or PCV13 period	5 (2–8)	8 (1–10)	5 (2–8)	8 (1–10)	5 (2–8)	8 (1–10)	4 (2–8)	8 (1–10)	5 (2–8)	8 (1–10)	5 (2–8)	8 (1–10)	8 (1–10)
Number of IPD cases													
All years, total	9011	59 977	2511	21626	8282	116510	9440	98203	17142	192 442	46 386	488758	527692
All years, median per site	348 (22–2599)	622 (44–11594)	151 (17–634)	310 (15–4672)	306 (24–2352)	1384 (20–27576)	394 (11–2875)	1461 (11–23 510)	502 (10–5368)	2640 (10–49475)	1117 (22–11030)	4816 (77–115393)	2981 (19–115393)
Pre-PCV period, total	5602	21803	1178	6339	2744	30 968	2753	18648	4554	37466	16 831	115 224	127759
Pre-PCV period, median per site	238 (9–1656)	235 (10–5806)	54 (4–399)	106 (4–2045)	67 (9–1130)	467 (9–11930)	196 (2–1236)	505 (2–8560)	455 (2–1650)	1022 (2–17672)	933 (29–4788)	2206 (29–46 009)	1245 (29–46009)
PCV7 period, total	1353	18167	494	7340	750	37502	1664	29 867	3385	55 831	8646	148707	154197
PCV7 period, median per site	204 (23–429)	250 (23–3289)	72 (9–150)	100 (9–1452)	286 (9–687)	617 (9–10704)	205 (6–721)	544 (6–8973)	460 (5–1726)	818 (5–16891)	950 (52–3128)	1966 (52–36188)	1641 (52–36188)
PCV10 or PCV13 period, total	2056	20007	839	7947	3788	48040	5023	49688	9203	99145	20 909	224827	245736
PCV10 or PCV13 period, median per site	80 (7–565)	238 (11–3599)	42 (2–152)	125 (0–1780)	37 (6–1222)	638 (2–11685)	265 (3–1639)	880 (3–10075)	491 (7–2976)	1673 (3–22077)	442 (22–6242)	2084 (13–45585)	1283 (13–45585)
Proportion of children vaccinated with PCV10 or PCV13 across sites, %**††	91% (55–97)	91% (64–99)	92% (55–97)	88% (64–99)	92% (55–97)	88% (64–99)	93% (55–97)	88% (64–99)	93% (55–7)	88% (64–99)	92% (55–97)	91% (64–99)	91% (55–99)
Proportion of vaccine-type IPD pre-PCV across sites, %‡‡	77% (67–90)	91% (81–100)	79% (44–84)	84% (65–100)	69% (52–77)	72% (48–84)	60% (46–69)	71% (59–79)	59% (33–64)	74% (56–83)	71% (58–90)	78% (63–100)	76% (58–100)
Proportion of IPD cases fully serotyped across sites, %††	95% (87–99)	85% (68–99)	95% (87–99)	86% (68–99)	95% (85–99)	85% (68–99)	95% (85–99)	86% (68–99)	95% (85–99)	86% (68–99)	95% (85–99)	85% (68–99)	89% (68–99)

Data are n, n (%), or median (range). IPD=invasive pneumococcal disease. PCV=pneumococcal conjugate vaccine. PCV7=seven-valent PCV (Prevenar, Pfizer). PCV10=ten-valent PCV (Synflorix, GSK). PCV13=13-valent PCV (Prevenar 13, Pfizer). PPV23=23-valent pneumococcal polysaccharide vaccine (Pneumovax23, Merck).

* Quebec used both products but is counted in the total only once.

† UN regions are adapted from the UN Statistics Division.^19^

‡ World Bank income levels are as of November, 2020.^20^

§ Gavi countries are those that are eligible for or have graduated from Gavi, the Vaccine Alliance.

¶ Strata were defined as fewer than 300 cases per 100 000 children (low burden), 300 to fewer than 2000 cases per 100 000 children (medium burden), or 2000 or more cases per 100 000 children (high burden). The year 2000 was the first year of PCV introduction into infant immunisation programmes in any country.^1^

‖ Any recommendation (individuals at high risk only or universal) for any adult age group; where data were available, adult PPV23 and PCV13 uptake was generally low.

** Annual PCV uptake estimates provided by the surveillance site for PCV10 or PCV13 years of data included in analyses. Uptake is for the primary series of PCV by age 12 months (if available, for some sites, up to age 15 months), excluding the year of vaccine roll-out. If unavailable, annual PCV uptake estimates provided by the surveillance site for the primary series plus the booster dose by age 23 months, excluding the year of vaccine roll-out, were used. If PCV uptake data from the surveillance site were unavailable, WHO and UNICEF Estimates of National Immunization Coverage PCV uptake, excluding the year of vaccine roll-out, were used (appendix 2 pp 18–21). Medians were generated using the median uptake for each site across years of PCV10 and PCV13 data included in the analyses, excluding the year of PCV10 or PCV13 introduction.

†† The number of fully serotyped IPD cases (excluding not serotyped, untypeable, pool, serogroup-only, and undistinguished cases) divided by the total number of cases reported.

‡‡ PCV10 serotypes for PCV10 sites and PCV13 serotypes for PCV13 serotypes in the pre-any PCV period (ie, before PCV7, if used).

## Data Availability

Restrictions apply to the availability of these data. Data were obtained under data-sharing agreements from contributing surveillance sites and can only be shared by contributing organisations with their permission.

## Research in context

### Evidence before this study

Many countries have published country-specific impact studies evaluating the effect of introducing ten-valent (PCV10) or 13-valent (PCV13) pneumococcal conjugate vaccines (PCVs) into their national infant immunisation programmes on invasive pneumococcal disease (IPD) incidence. Although all of these studies showed declines in vaccine-type IPD, the magnitude of those declines, increases in non-vaccine serotypes, and net effect on overall IPD varied between sites, and serotype-specific analyses were restricted by sample size. Differences in study context and design, including in PCV use (eg, number of years PCVs were in use, immunisation coverage, dosing schedules, and previous seven-valent PCV use before switching to PCV10 or PCV13), case definitions, and surveillance or analytical methods, contributed to variability. Additionally, multicountry comparisons are needed to assess differences between vaccine formulations and schedules. We searched PubMed without language restrictions for studies published between Jan 1, 2010, and March 3, 2024, reporting global analyses of changes in IPD incidence after PCV10 or PCV13 introduction relative to before PCV using the search terms “PCV”, “IPD”, “impact OR effect*”, AND “NOT cost”. 15 previous multicountry impact assessments were published between 2016 and 2024; analyses were restricted to specific regions, high-income countries, certain serotypes (including antimicrobial-resistant), or certain populations, or did not evaluate long-term effects in settings of mature PCV10 or PCV13 use (ie, >5 years of PCV10 or PCV13 use with high uptake) relative to the pre-PCV period. Most analyses also used only data available in the published literature, which restricts analyses in both scope and ability to reduce bias by standardising methodology across sites.

### Added value of this study

We aimed to describe the full direct and indirect impact of PCV10 or PCV13 after long-term use on incidence of IPD due to any serotypes or vaccine or non-vaccine serotypes only, and to assess whether the effects differed by product, schedule, or serotype, for all ages globally. Sites collaborated to share their data using a common data collection template, which facilitated evaluation of surveillance data for potential biases, standardisation in methods and definitions across sites, robust analytical methods, and a broad range of detailed analyses. This analysis also described impact in all ages, specifically for age groups younger than 5 years, 5–17 years, 18–49 years, 50–64 years, and 65 years or older.

### Implications of all the available evidence

The totality of evidence shows that long-term use of PCV10 and PCV13 reduced vaccine-serotype IPD (responsible for most IPD in children) and, despite increases in non-vaccine serotypes, resulted in reduction of overall IPD in all ages. Serotype 19A increased with PCV10 use and decreased with PCV13 use, suggesting that PCV10 sites could expect a greater effect by using a formulation that includes serotype 19A; otherwise, the effect was similar between PCV10 and PCV13 and between different schedules. Further reductions are anticipated with higher-valency PCVs for the serotypes they cover—with the exception of serotype 3, which does not appear to be affected by PCV use—and cross-protection for some related serotypes might be expected. Immunising infants reduces IPD in all ages, but because indirect effects can take many years to maximise, immunising older, at-risk individuals in the early years after introduction could accelerate reductions in IPD. Although differences in overall effect between PCV10 and PCV13 and dosing schedules appeared minimal, local serotype distribution should be considered for product and schedule choice.
