# Supplementary material for: Global impact of ten-valent and 13-valent pneumococcal conjugate vaccines on invasive pneumococcal disease in all ages (the PSERENADE project): a global surveillance analysis
Source: Lancet Infect Dis. Author manuscript; Available in PMC 2025 Apr 8. (PMC11947069; doi:10.1016/S1473-3099(24)00665-0)
Supplement: Supplement 1 [file NIHMS2058689-supplement-Supplement_1.pdf]

THE LANCET
Infectious Diseases

Supplementary appendix 1

This appendix formed part of the original submission and has been peer reviewed. We post it as supplied by the authors.

This online publication has been corrected. The corrected version first appeared at thelancet.com/infection on Jan 17, 2025

Supplement to: Bennett JC, Deloria Knoll M, Kagucia EW, et al. Global impact of ten-valent and 13-valent pneumococcal conjugate vaccines on invasive pneumococcal disease in all ages (the PSERENADE project): a global surveillance analysis. *Lancet Infect Dis* 2024; published online Dec 17. https://doi.org/10.1016/S1473-3099(24)00665-0.

Appendix 1

1. Appendix Table A1. PSERENADE Team Group Authors

Name	Affiliation
Khalid Zerouali, PhD	Department of Microbiology, Faculty of Medicine and Pharmacy, Hassan II University of Casablanca, Casablanca 20000, Morocco; Bacteriology-Virology and Hospital Hygiene Laboratory, Ibn Rochd University Hospital Centre, Casablanca 20250, Morocco
Didrik F. Vestrheim, MD	Department of Public Health, Ministry of Health and Care Services, Oslo, Norway
Jennifer R. Verani, MD	Division of Bacterial Diseases, National Center for Immunizations and Respiratory Diseases, Centers for Disease Control and Prevention, Atlanta, GA 30329, USA; Division of Global Health Protection, Center for Global Health, Centers for Disease Control and Prevention (CDC), Nairobi, Kenya
Emmanuelle Varon, MD	National Reference Centre for Pneumococci, Data Research Department (DRIIM), Centre Hospitalier Intercommunal de Créteil, 94000 Créteil, France
Nina M. van Sorge, PhD	Medical Microbiology and Infection Prevention, Netherlands Reference Laboratory for Bacterial Meningitis, Amsterdam UMC, location AMC, University of Amsterdam, 1105 AZ Amsterdam, The Netherlands
Maria Teresa Valenzuela, MD	Department of Public Health and Epidemiology, Faculty of Medicine, Universidad de los Andes, Santiago, Las Condes, Metropolitan Region, Chile
Georgina Tzanakaki, PhD	National Meningitis Reference Laboratory, Dept of Public Health Policy, School of Public Health, University of West Attica, Athens, Greece
Maija Toropainen, PhD	Department of Health Security, Finnish Institute for Health and Welfare, 00271 Helsinki, Finland
Koh Cheng Thoon, MRCPCH	KK Women's and Children's Hospital, 229899, Singapore
Todd D. Swarthout, PhD	Julius Center for Health Sciences and Primary Care, University Medical Center Utrecht, Utrecht University, Utrecht, the Netherlands; Malawi Liverpool Wellcome Programme, P.O. Box 30096, Chichiri, Blantyre 3, Malawi; NIHR Mucosal Pathogens Research Unit, Research Department of Infection, Division of Infection and Immunity, University College London, London, UK
Catherine G. Sutcliffe, PhD	Johns Hopkins Bloomberg School of Public Health, Baltimore, MD 21205, United States
Andrew Smith, PhD	Bacterial Respiratory Infection Service, Scottish Microbiology Reference Laboratory, NHS GG&C, G4 0SF Glasgow, United Kingdom; Glasgow Dental School, University of Glasgow, United Kingdom
Aalisha Sahu Khan, MD	Ministry of Health and Medical Services, Suva, Fiji
Flavia Riccardo, PhD	Department of Infectious Diseases, Italian National Institute of Health (Istituto Superiore di Sanità, ISS), 00161 Rome, Italy
Alisa Reasonover, BS	Arctic Investigations Program, Division of Preparedness and Emerging Infections, National Center for Emerging and Zoonotic Infectious Diseases, Centers for Disease Control and Prevention, Anchorage, AK 99508, United States
Hafizur Rahman, DMT	Child Health Research Foundation, Dhaka 1207, Bangladesh

Marie-Cecile Ploy, PhD	University Hospital Centre Limoges, Regional Observatories for Pneumococci, 87000 Limoges, France
Tamara Pilishvili, PhD	Division of Bacterial Diseases, National Center for Immunizations and Respiratory Diseases, Centers for Disease Control and Prevention, Atlanta, GA 30329, USA
Kate Pennington, MPH	Communicable Disease Epidemiology and Surveillance Section, Office of Health Protection, Australian Government Department of Health, 2606 Canberra ACT, Australia
Stephen I. Pelton, MD	Boston University Schools of Medicine and Public Health, Boston, MA 02118, United States
Kazunori Oishi, PhD	Toyama Institute of Health, Imizu, 939-0363 Toyama, Japan
Carmen Muñoz-Almagro, MD, PhD	Molecular Microbiology Department, Hospital Sant Joan de Déu Research Institute, 08950 Esplugues de Llobregat, Barcelona, Spain; Medicine Department, Universitat Internacional de Catalunya, 08017 Barcelona, Spain; CIBER Epidemiología y Salud Pública, (CIBERESP), 28029 Madrid, Spain
Tuya Mungun, MD	National Center of Communicable Diseases (NCCD), Ministry of Health, Bayanzurkh district, 13336 Ulaanbaatar, Mongolia
Eva Morfeldt, PhD	Department of Microbiology Public Health Agency of Sweden, 171 82 Solna, Sweden
Ioanna Magaziotou, PhD	National Public Health Organisation, 15123 Athens, Greece
Brigitte Lefebvre, PhD	Laboratoire de Santé Publique du Québec, Sainte-Anne-de-Bellevue, Quebec H9X 3R5, Canada
Alicja Kuch, PhD	National Reference Centre for Bacterial Meningitis, National Medicines Institute, 00-725 Warsaw, Poland
Pavla Krizova, MD	National Institute of Public Health (NIPH), 100 00, Praha 10, Czech Republic
Vicki Krause, MD	Centre for Disease Control, Department of Health and Community Services, Darwin City NT 8000, Australia
Miwako Kobayashi, MD	Division of Bacterial Diseases, National Center for Immunizations and Respiratory Diseases, Centers for Disease Control and Prevention, Atlanta, GA 30329, USA
Jackie Kleynhans, MSc	Centre for Respiratory Diseases and Meningitis, National Institute for Communicable Diseases of the National Health Laboratory Service, Sandringham, 2192 Johannesburg, South Africa; School of Public Health, Faculty of Health Sciences, University of the Witwatersrand, Johannesburg, South Africa
James D. Kellner, MD	Department of Pediatrics, University of Calgary, and Alberta Health Services, Calgary Alberta T3B 6A8, Canada
Ilias Hossain, MPH	Medical Research Council Unit The Gambia at London School of Hygiene & Tropical Medicine, PO Box 273, Banjul, The Gambia
Juan Carlos Hormazabal, MD	Instituto de Salud Pública de Chile, 7780050 Santiago, Santiago Metropolitan, Chile
Germaine Hanquet, PhD	Epidemiology Department, Epiconcept, 75012 Paris, France
Charlotte Gilkison, MPH	Epidemiology Team, Institute of Environmental Science and Research, Porirua, 5022 Wellington, New Zealand
Helga Erlendsdottir, MS	Department of Clinical Microbiology, Landspitali - The National University Hospital, Hringbraut, 101 Reykjavik, Iceland

Elina Dimina, MD	Centre for disease prevention and control of Latvia, Riga, 1005, Latvia
Geneviève Deceuninck, MD	Quebec University Hospital Research Centre, Québec, QC G1V 4G2, Canada
Sara de Miguel, MD	Epidemiology Department, Dirección General de Salud Pública, 28009 Madrid, Spain
Linda de Gouveia, Btech	Centre for Respiratory Diseases and Meningitis, National Institute for Communicable Diseases of the National Health Laboratory Service, Sandringham, 2192 Johannesburg, South Africa
Tine Dalby, PhD	Infectious Disease Epidemiology and Prevention, Statens Serum Institut, DK-2300 Copenhagen S, Denmark; Department of Bacteria, Parasites and Fungi, Statens Serum Institut, DK-2300 Copenhagen S, Denmark
Mary Corcoran, PhD	Irish Meningitis and Sepsis Reference Laboratory, Children's Health Ireland at Temple Street, Temple Street, Dublin 1, D01 YC76, Ireland; Royal College of Surgeons in Ireland, Dublin, Ireland
Kin-Hung Chow, MSc	Department of Microbiology and Carol Yu Centre for Infection, Queen Mary Hospital, The University of Hong Kong, Hong Kong SAR, China
Lucia Pastore Celentano, MD	European Centre for Disease Prevention and Control, 169 73 Solna, Sweden
Jesús Castilla, MD	Instituto de Salud Pública de Navarra - IdiSNA, 31003 Pamplona, Navarra, Spain; CIBER Epidemiología y Salud Pública, (CIBERESP), 28029 Madrid, Spain
Carrie L. Byington, MD	University of Utah Department of Pediatrics (emeritus), Salt Lake City, UT 84108, United States; University of California Health System, Oakland, CA 94607, United States
Mária Avdicová, MD	National Reference Centre for Pneumococcal and Haemophilus Diseases, Regional Authority of Public Health, 975 56 Banská Bystrica, Slovak Republic
Laurie Aukes, RN	Vaccine Study Center, Kaiser Permanente, Oakland, CA 94612, United States
Michelle Ang, PhD	National Public Health Laboratory, National Centre for Infectious Diseases, Singapore 308442, Singapore
Zahin Amin-Chowdhury, MSc	Immunisation and Countermeasures Division, Public Health England, NW9 5EQ, London, United Kingdom
Samanta C. G. Almeida, PhD	National Laboratory for Meningitis and Pneumococcal Infections, Center of Bacteriology, Institute Adolfo Lutz (IAL), São Paulo, 01246-902, Brazil
Toronto Invasive Bacterial Diseases Network	

2. Appendix Table A2. Acknowledgment List

PSERENADE Technical Advisory Group
Thomas Cherian
William P. Hausdorff
Marc Lipsitch
Shabir A. Madhi
Elizabeth Miller
Catherine Satzke
Cynthia G. Whitney
Ron Dagan

**World Health Organization**	
	Jenny A. Walldorf
	Heidi M. Soeters
**Johns Hopkins University**	
	Meagan E. Peterson
	Jenna Sinkevitch
	Yunfeng Cao
	Peggy Gross
	Donna Hesson
	Ananya Kumar
	Kate Perepezko
	Francesca Schiaffino Salazar
	Daniel Stephens
	Melody Xiao
**Dirección General de Salud Pública, Comunidad de Madrid, Spain**	
	Luis García Comas
	Maria Ordobás Gavín
**Department of Infectious Diseases, Italian National Institute of Health (Istituto Superiore di Sanità, ISS), Rome, Italy**	
	Martina Del Manso
**Department of Microbiology, Faculty of Medicine and Pharmacy, Hassan II University of Casablanca, Casablanca, Morocco; Bacteriology-Virology and Hospital Hygiene Laboratory, Ibn Rochd University Hospital Centre, Casablanca, Morocco**	
	Néhémie Nzoyikorera
**Surveillance and Public Health Emergency Response, Public Health Agency of Catalonia, Barcelona, Spain**	
	Sonia Broner
	Conchita Izquierdo
**Australian National Notifiable Diseases Surveillance data were provided by the Office of Health Protection, Australian Government Department of Health, on behalf of the Communicable Diseases Network Australia and the Enhanced Invasive Pneumococcal Disease Surveillance Working Group.**	
