# Supplementary material for: Global impact of ten-valent and 13-valent pneumococcal conjugate vaccines on invasive pneumococcal disease in all ages (the PSERENADE project): a global surveillance analysis
Source: Lancet Infect Dis. Author manuscript; Available in PMC 2025 Apr 8. (PMC11947069; doi:10.1016/S1473-3099(24)00665-0)
Supplement: Supplement 2 [file NIHMS2058689-supplement-Supplement_2.pdf]

THE LANCET
Infectious Diseases

Supplementary appendix 2

This appendix formed part of the original submission and has been peer reviewed. We post it as supplied by the authors.

Supplement to: Bennett JC, Deloria Knoll M, Kagucia EW, et al. Global impact of ten-valent and 13-valent pneumococcal conjugate vaccines on invasive pneumococcal disease in all ages (the PSERENADE project): a global surveillance analysis. *Lancet Infect Dis* 2024; published online Dec 17. https://doi.org/10.1016/S1473-3099(24)00665-0.

Supplementary Materials

Table of Contents

Acronyms 6

1. Supplementary Methods 7

Supplementary Methods 1.1 Accounting for cases with missing ST information in analyses of VT, NVT, and ST-specific IPD 7

Supplementary Table 1.1 Methods to account for cases with missing ST information in VT, NVT, and ST-specific analyses. Adjustments assumed serotype information was missing completely at random; i.e., among subsets of cases with the same values of observed covariates, the serotype distribution was assumed to be similar to the unknown serotype distribution of cases that were not serotyped or not fully serotyped. 8

Supplementary Figure 1.1 Method for estimating annual IPD IRRs comparing the pre-PCV period to each post-PCV10/13 year for each included site with pre- and post-PCV10/13 data: example for all ST IPD for children aged <5 years from one site 9

Supplementary Methods 1.2 Defining PCV7 impact strata 10

Supplementary Figure 1.2 Site-specific modeled PCV7-type IPD IRRs for children aged <5 years in the last year of PCV7-use prior to PCV10/13 introduction compared to the pre-PCV period. Data shown for sites that used PCV7, were included in VT/NVT analyses, and had both pre- and post-PCV data available. 11

Supplementary Methods 1.3 Estimating annual IPD IRRs comparing the pre-PCV period to each post-PCV10/13 year using a Bayesian multi-level, mixed-effects Poisson regression 12

2. Description of Data 13

Supplementary Table 2.1 Sites included in PSERENADE evaluated for the IPD incidence analyses by age group 13

Supplementary Table 2.2 Description of surveillance data included in analyses by PCV product and PCV7-impact strata 16

Supplementary Table 2.3 Description of surveillance data included in analysis by site 18

Supplementary Figure 2.1 Number of IPD cases per site included in the analysis by region and age group 22

Supplementary Figure 2.2 All-site weighted average incidence rate ratios for all serotype IPD and vaccine/serotype specific IPD, comparing annual post-PCV10/13 incidence rate to the average pre-PCV incidence rate by age group for ages 5-64 years 23

2.2.1. 5-17 years 23

2.2.2. 18-49 years 24

2.2.3. 50-64 years 25

Supplementary Figure 2.3 Annual site-specific PCV uptake across years of data included in analyses 26

Supplementary Data 2.1 ST6C Trends 27

Supplementary Figure 2.4 Observed ST6C IPD IRs for (A) children aged <5 years at PCV10 sites, (B) children aged <5 years at PCV13 sites, (C) adults aged ≥65 years at PCV10 sites and (D) adults aged ≥65 years at PCV13 sites. 28

Supplementary Figure 3.1 All-site weighted average IRRs for PCV13 ST IPD comparing the annual post-PCV10/13 IR to the average pre-PCV IR by age group..... 31

Supplementary Figure 3.2 All-site weighted average IRRs for PCV13 ST-minus-ST3 IPD comparing the annual post-PCV10/13 IR to the average pre-PCV IR by age group..... 32

Supplementary Figure 3.3 Site-specific modeled all ST IPD IRRs for children aged <5 years 33

Supplementary Figure 3.4 Site-specific modeled all ST IPD IRRs for adults aged ≥65 years 33

Supplementary Figure 3.5 Site-specific modeled ST19A IPD IRRs for children aged <5 years 34

Supplementary Figure 3.6 Site-specific modeled ST19A IPD IRRs for adults aged ≥65 years..... 34

Supplementary Figure 3.7 Site-specific modeled ST3 IPD IRRs for children aged <5 years..... 35

Supplementary Figure 3.8 Site-specific modeled ST3 IPD IRRs for adults aged ≥65 years..... 35

Supplementary Figure 3.9 Site-specific modeled all non-PCV13 ST IPD IRRs for children aged <5 years 36

Supplementary Figure 3.10 Site-specific modeled all non-PCV13 ST IPD IRRs for adults aged ≥65 years 36

Supplementary Figure 3.11 Site-specific modeled **all ST** IPD IRRs **by infant PCV10/13 schedule** for children aged <5 years and adults aged ≥65 years 37

Supplementary Figure 3.12 Site-specific modeled **all PCV10 ST** IPD IRRs **by infant PCV10/13 schedule** for children aged <5 years and adults aged ≥65 years 38

Supplementary Figure 3.13 Site-specific modeled **ST6A** IPD IRRs **by infant PCV10/13 schedule** for children aged <5 years and adults aged ≥65 years 39

Supplementary Figure 3.14 Site-specific modeled **all non-PCV13 ST** IPD IRRs **by infant PCV10/13 schedule** for children aged <5 years and adults aged ≥65 years..... 40

Supplementary Figure 3.15 Site-specific modeled **all ST** IPD IRRs **by PCV10/13 catch-up program** for children aged <5 years and adults aged ≥65 years 41

Supplementary Figure 3.16 Site-specific modeled **all ST** IPD IRRs **by region** for children <5 aged years and adults aged ≥65 years 42

Supplementary Figure 3.17 Site-specific modeled all ST IPD IRRs for adults aged ≥65 years by adult pneumococcal vaccine recommendation. 43

Supplementary Figure 3.18 Site-specific modeled all ST IPD IRRs for adults ≥65 years by adult pneumococcal vaccine recommendation. 44

Supplementary Figure 3.19 All ST IPD all-site weighted average IRRs comparing the annual post-PCV10/13 IR to the average pre-PCV IR for **children aged <5 years** 45

Supplementary Figure 3.20 All ST IPD all-site weighted average IRRs comparing the annual post-PCV10/13 IR to the average pre-PCV IR for **children aged 5–17 years** 46

Supplementary Figure 3.21 All ST IPD all-site weighted average IRRs comparing the annual post-PCV10/13 IR to the average pre-PCV IR for **adults aged 18–49 years** ... 47

Supplementary Figure 3.22 All ST IPD all-site weighted average IRRs comparing the annual post-PCV10/13 IR to the average pre-PCV IR for **adults aged 50–64 years** ... 48

Supplementary Figure 3.23 All ST IPD all-site weighted average IRRs comparing the annual post-PCV10/13 IR to the average pre-PCV IR for **adults aged ≥65 years** ... 49

Supplementary Figure 3.24 PCV7 ST IPD all-site weighted average IRRs comparing the annual post-PCV10/13 IR to the average pre-PCV IR for **children aged <5 years** ... 50

Supplementary Figure 3.25 PCV7 ST IPD all-site weighted average IRRs comparing the annual post-PCV10/13 IR to the average pre-PCV IR for **children aged 5–17 years** ... 51

Supplementary Figure 3.26 PCV7 ST IPD all-site weighted average IRRs comparing the annual post-PCV10/13 IR to the average pre-PCV IR for **adults aged 18–49 years** ... 52

Supplementary Figure 3.27 PCV7 ST IPD all-site weighted average IRRs comparing the annual post-PCV10/13 IR to the average pre-PCV IR for adults aged 50–64 years ... 53

Supplementary Figure 3.28 STs 1, 5, & 7F (PCV10X-type) IPD all-site weighted average IRRs comparing the annual post-PCV10/13 IR to the average pre-PCV IR for **children aged <5 years** ... 55

Supplementary Figure 3.29 STs 1, 5, & 7F (PCV10X-type) IPD all-site weighted average IRRs comparing the annual post-PCV10/13 IR to the average pre-PCV IR for **children aged 5–17 years** ... 56

Supplementary Figure 3.30 STs 1, 5, & 7F (PCV10X-type) IPD all-site weighted average IRRs comparing the annual post-PCV10/13 IR to the average pre-PCV IR for **adults aged 18–49 years** ... 57

Supplementary Figure 3.31 STs 1, 5, & 7F (PCV10X-type) IPD all-site weighted average IRRs comparing the annual post-PCV10/13 IR to the average pre-PCV IR for **adults aged 50–64 years** ... 58

Supplementary Figure 3.32 STs 1, 5, & 7F (PCV10X-type) IPD all-site weighted average IRRs comparing the annual post-PCV10/13 IR to the average pre-PCV IR for **adults aged ≥65 years** ... 59

Supplementary Figure 3.33 All PCV10 ST IPD all-site weighted average IRRs comparing the annual post-PCV10/13 IR to the average pre-PCV IR for children aged <5 years ... 60

Supplementary Figure 3.34 All PCV10 ST IPD all-site weighted average IRRs comparing the annual post-PCV10/13 IR to the average pre-PCV IR for **children aged 5–17 years** ... 61

Supplementary Figure 3.35 All PCV10 ST IPD all-site weighted average IRRs comparing the annual post-PCV10/13 IR to the average pre-PCV IR for **adults aged 18–49 years** ... 62

Supplementary Figure 3.36 All PCV10 ST IPD all-site weighted average IRRs comparing the annual post-PCV10/13 IR to the average pre-PCV IR for **adults aged 50–64 years** ... 63

Supplementary Figure 3.37 All PCV10 ST IPD all-site weighted average IRRs comparing the annual post-PCV10/13 IR to the average pre-PCV IR for **adults aged ≥65 years** ... 64

Supplementary Figure 3.38 ST6A IPD all-site weighted average IRRs comparing the annual post-PCV10/13 IR to the average pre-PCV IR for **children aged <5 years** ... 65

Supplementary Figure 3.39 ST6A IPD all-site weighted average IRRs comparing the annual post-PCV10/13 IR to the average pre-PCV IR for children aged 5–17 years ... 66

Supplementary Figure 3.40 ST6A IPD all-site weighted average IRRs comparing the annual post-PCV10/13 IR to the average pre-PCV IR for **adults aged 18–49 years** 67

Supplementary Figure 3.41 ST6A IPD all-site weighted average IRRs comparing the annual post-PCV10/13 IR to the average pre-PCV IR for **adults aged 50–64 years** 68

Supplementary Figure 3.42 ST6A IPD all-site weighted average IRRs comparing the annual post-PCV10/13 IR to the average pre-PCV IR for adults aged ≥65 years 69

Supplementary Figure 3.43 ST19A IPD all-site weighted average IRRs comparing the annual post-PCV10/13 IR to the average pre-PCV IR for **children aged <5 years** 70

Supplementary Figure 3.44 ST19A IPD all-site weighted average IRRs comparing the annual post-PCV10/13 IR to the average pre-PCV IR for children aged 5–17 years 71

Supplementary Figure 3.45 ST19A IPD all-site weighted average IRRs comparing the annual post-PCV10/13 IR to the average pre-PCV IR for adults aged 18–49 years 72

Supplementary Figure 3.46 ST19A IPD all-site weighted average IRRs comparing the annual post-PCV10/13 IR to the average pre-PCV IR for adults aged 50–64 years 73

Supplementary Figure 3.47 ST19A IPD all-site weighted average IRRs comparing the annual post-PCV10/13 IR to the average pre-PCV IR for **adults aged ≥65 years** 74

Supplementary Figure 3.48 ST3 IPD all-site weighted average IRRs comparing the annual post-PCV10/13 IR to the average pre-PCV IR for children aged <5 years 75

Supplementary Figure 3.49 ST3 IPD all-site weighted average IRRs comparing the annual post-PCV10/13 IR to the average pre-PCV IR for children aged 5–17 years 76

Supplementary Figure 3.50 ST3 IPD all-site weighted average IRRs comparing the annual post-PCV10/13 IR to the average pre-PCV IR for adults aged 18–49 years 77

Supplementary Figure 3.51 ST3 IPD all-site weighted average IRRs comparing the annual post-PCV10/13 IR to the average pre-PCV IR for adults aged 50–64 years 78

Supplementary Figure 3.52 ST3 IPD all-site weighted average IRRs comparing the annual post-PCV10/13 IR to the average pre-PCV IR for adults aged ≥65 years 79

Supplementary Figure 3.53 All PCV13 ST IPD all-site weighted average IRRs comparing the annual post-PCV10/13 IR to the average pre-PCV IR for children aged <5 years........ 80

Supplementary Figure 3.54 All PCV13 ST IPD all-site weighted average IRRs comparing the annual post-PCV10/13 IR to the average pre-PCV IR for children aged 5–17 years........ 81

Supplementary Figure 3.55 All PCV13 ST IPD all-site weighted average IRRs comparing the annual post-PCV10/13 IR to the average pre-PCV IR for adults aged 18–49 years 82

Supplementary Figure 3.56 All PCV13 ST IPD all-site weighted average IRRs comparing the annual post-PCV10/13 IR to the average pre-PCV IR for adults aged 50–64 years 83

Supplementary Figure 3.57 All PCV13 ST IPD all-site weighted average IRRs comparing the annual post-PCV10/13 IR to the average pre-PCV IR for adults aged ≥65 years 84

Supplementary Figure 3.58 All PCV13 ST minus ST3 IPD all-site weighted average IRRs comparing the annual post-PCV10/13 IR to the average pre-PCV IR for children aged <5 years 85

Supplementary Figure 3.59 All PCV13 ST minus ST3 IPD all-site weighted average IRRs comparing the annual post-PCV10/13 IR to the average pre-PCV IR for **children aged 5–17 years** 86

Supplementary Figure 3.60 All PCV13 ST minus ST3 IPD all-site weighted average IRRs comparing the annual post-PCV10/13 IR to the average pre-PCV IR for adults aged 18–49 years 87

Supplementary Figure 3.61 All PCV13 ST minus ST3 IPD all-site weighted average IRRs comparing the annual post-PCV10/13 IR to the average pre-PCV IR for adults aged 50–64 years 88

Supplementary Figure 3.62 All PCV13 ST minus ST3 IPD all-site weighted average IRRs comparing the annual post-PCV10/13 IR to the average pre-PCV IR for adults aged ≥65 years 89

Supplementary Figure 3.63 All non-PCV10 ST IPD all-site weighted average IRRs comparing the annual post-PCV10/13 IR to the average pre-PCV IR for children aged <5 years... 90

Supplementary Figure 3.64 All non-PCV10 ST IPD all-site weighted average IRRs comparing the annual post-PCV10/13 IR to the average pre-PCV IR for children aged 5–17 years.. 91

Supplementary Figure 3.65 All non-PCV10 ST IPD all-site weighted average IRRs comparing the annual post-PCV10/13 IR to the average pre-PCV IR for adults aged 18–49 years 92

Supplementary Figure 3.66 All non-PCV10 ST IPD all-site weighted average IRRs comparing the annual post-PCV10/13 IR to the average pre-PCV IR for adults aged 50–64 years 93

Supplementary Figure 3.67 All non-PCV10 ST IPD all-site weighted average IRRs comparing the annual post-PCV10/13 IR to the average pre-PCV IR for adults aged ≥65 years 94

Supplementary Figure 3.68 Non-PCV10 ST minus ST6A IPD all-site weighted average IRRs comparing the annual post-PCV10/13 IR to the average pre-PCV IR for children aged <5 years 95

Supplementary Figure 3.69 Non-PCV10 ST minus ST6A IPD all-site weighted average IRRs comparing the annual post-PCV10/13 IR to the average pre-PCV IR for children aged 5–17 years 96

Supplementary Figure 3.70 Non-PCV10 ST minus ST6A IPD all-site weighted average IRRs comparing the annual post-PCV10/13 IR to the average pre-PCV IR for adults aged 18–49 years......................... 97

Supplementary Figure 3.71 Non-PCV10 ST minus ST6A IPD all-site weighted average IRRs comparing the annual post-PCV10/13 IR to the average pre-PCV IR for adults aged 50–64 years......................... 98

Supplementary Figure 3.72 Non-PCV10 ST minus ST6A IPD all-site weighted average IRRs comparing the annual post-PCV10/13 IR to the average pre-PCV IR for adults aged ≥65 years............................. 99

Supplementary Figure 3.73 All non-PCV13 ST IPD all-site weighted average IRRs comparing the annual post-PCV10/13 IR to the average pre-PCV IR for children aged <5 years... 100

Supplementary Figure 3.74 All non-PCV13 ST IPD all-site weighted average IRRs comparing the annual post-PCV10/13 IR to the average pre-PCV IR for children aged 5–17 years....................................... 101

Supplementary Figure 3.75 All non-PCV13 ST IPD all-site weighted average IRRs comparing the annual post-PCV10/13 IR to the average pre-PCV IR for adults aged 18–49 years 102

Supplementary Figure 3.76 All non-PCV13 ST IPD all-site weighted average IRRs comparing the annual post-PCV10/13 IR to the average pre-PCV IR for adults aged 50–64 years 103

Supplementary Figure 3.77 All non-PCV13 ST IPD all-site weighted average IRRs comparing the annual post-PCV10/13 IR to the average pre-PCV IR for adults aged ≥65 years 104

4. Sensitivity Analyses 105

Supplementary Figure 4.1 Sensitivity analysis excluding sites without pre-PCV data compared to results from the primary analysis: All-site weighted average IRRs for all serotype IPD for children aged <5 years, comparing the annual post-PCV10/13 IR to the average pre-PCV IR........ 105

Supplementary Figure 4.2 Sensitivity analysis comparing all-site weighted average IRRs when estimated using (1) a single linear mixed effects regression including data from all sites with a three-way interaction between year since PCV10/13 introduction, prior PCV7 impact, and product........ 106

Acronyms

Incidence Rate (IR)

Incidence Rate Ratio (IRR)

Invasive Pneumococcal Disease (IPD)

Non-Vaccine Serotype (NVT)

Pneumococcal Conjugate Vaccine (PCV)

Pneumococcal Polysaccharide Vaccine (PPV23)

Pneumococcal Serotype Replacement and Distribution Estimation Project (PSERENADE)

Serotype (ST)

Vaccine Serotype (VT)

1. Supplementary Methods

Supplementary Methods 1.1 Accounting for cases with missing ST information in analyses of VT, NVT, and ST-specific IPD

For adjustments for missing ST data, it was assumed that missing ST data were missing completely at random; that is, the ST distribution of serotyped cases was assumed not biased or different from the unknown ST distribution of cases that were not serotyped or were not fully serotyped. Site-year-age group strata where evidence were insufficient to support this assumption were excluded from VT, NVT, and ST-specific IPD analyses. For example, site-year-age group strata were excluded from VT, NVT, and ST-specific analyses when: cases were not serotyped with a comprehensive serotyping method and thus the ST distribution reflected only a subset of STs tested for, all cases were not attempted to be serotyped and selection of cases for serotyping was not random (e.g., more severe cases were preferentially selected for serotyping), or STs were reported for less than 50% of cases. Methods to account for missing ST data in VT, NVT, and ST-specific analyses are described in **Supplementary Table 1.1**

Supplementary Table 1.1 Methods to account for cases with missing ST information in VT, NVT, and ST-specific analyses. Adjustments assumed serotype information was missing completely at random; i.e., among subsets of cases with the same values of observed covariates, the serotype distribution was assumed to be similar to the unknown serotype distribution of cases that were not serotyped or not fully serotyped.

Categorization	Definition	Method to Account for Missing ST Data
Not serotyped	Serotyping was not attempted for any reason	Population denominators were adjusted by the proportion of cases that were serotyped (i.e., annual denominator *X* percent of cases that were serotyped in that year) for each site by year and age group. Because the proportion of cases serotyped varies across sites and over time, population denominators were adjusted rather than reapportioning STs to unknown ST cases in order to give appropriate weight to sites in the model based on ST data reported.
Serogrouped-only or undistinguished	Serogrouped-only (e.g., 6A/6B/6C/6D) and undistinguished (e.g., 6A/6C)	The case was included in VT or NVT analyses if the serogroup was exclusively VT or NVT. For serogroups that included both VT and NVT (i.e., 6, 7, 8, 18, 19, and 23), cases were redistributed for VT, NVT, and ST-specific analyses according to the distribution of fully serotyped cases within the serogroup by site, age group, and year. Where data were insufficient (i.e., >50% of cases undistinguished or fewer than 10 distinguished cases in the site, age group, year), cases were distributed using the ST distribution of the United Nations region and PCV-use time period. If data were still insufficient (i.e., >50% of cases undistinguished or fewer than 10 distinguished cases in the age group, region, PCV-use time period), the global ST distribution and PCV-use period were used.
Typed, but ST not identified	Serotyping was performed with a method that does not assess all STs and a specific ST was not identified	The case was counted as a NVT if the serotyping method tested for all VTs. For ST-specific analyses or if the serotyping method did not include all VTs, the case was treated as a not serotyped case as described above.
Untypeable	Comprehensive serotyping methodology was performed but a ST was not identified (e.g., non-encapsulated strain or an isolate that produced less capsule under lab conditions)	The case was counted as NVTs and excluded from ST-specific analyses.
Two STs reported	Two specimens taken from one case or two STs identified from one specimen	For VT and NVT analyses, the case was counted as VT if both STs were VT, as NVT if both STs were NVT, and as a not serotyped case, as described above, if one cases was VT and one case was NVT. For ST-specific analyses, the case was included in both ST-specific analyses as applicable.
ST pool	Only ST pool reported	For VT and NVT analyses, the case was counted as VT if all STs in pool were VT, as NVT if all STs in the pool were NVT, and as a not serotyped case, as described above, otherwise. For ST-specific analyses, the case was excluded.

Supplementary Figure 1.1 Method for estimating annual IPD IRRs comparing the pre-PCV period to each post-PCV10/13 year for each included site with pre- and post-PCV10/13 data: example for all ST IPD for children aged <5 years from one site

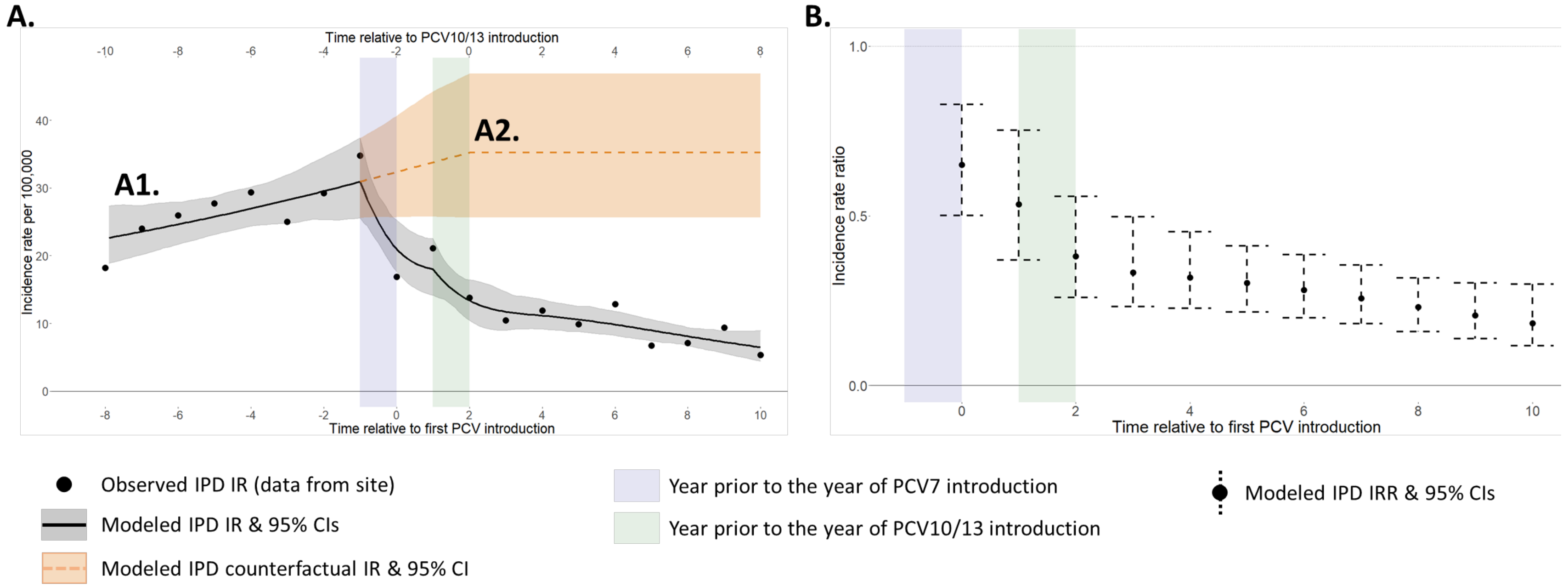

A. Annual IPD IRs.

A1. Plot A points show observed annual IPD IR (data received from site). Plot A black line and gray surrounding CIs show the modeled site-specific IR estimated for all years of available data using a Bayesian multi-level, mixed-effects Poisson regression separately for PCV10 and PCV13 sites. Model includes data from all other PCV10 or PCV13 sites for children aged <5 years with an offset for the population denominator and random effects for the intercept and slope. A non-linear break is included one year prior to PCV7 and PCV10/13 introduction to capture the change from the year prior to introduction to the year of PCV introduction and cubic spline knots are included at year +1 and +3 post-each PCV introduction to allow for flexibility in the IR of IPD following PCV introduction.

A2. Plot A orange dashed line and surrounding CIs show the modeled counterfactual IPD IR in the absence of PCV10/13 introduction. The counterfactual is estimated by extending the pre-PCV IR slope for three years post-PCV10/13 introduction before flattening to a slope of zero at the estimated rate onward.

B. Annual modeled IPD IRRs in each post-PCV10/13 year. IRRs are estimated by dividing the modeled IPD IR in each post-PCV year by the counterfactual IR.

Supplementary Methods 1.2 Defining PCV7 impact strata

Among sites that used PCV7 prior to PCV10/13, reductions in VT IPD and increases in NVT IPD had occurred during the PCV7 use period, thus impacting the IRR in year of PCV10/13 introduction relative to the pre-PCV period. To account for these differences due to variable PCV7 use, we stratified all-site weighted average IRRs by PCV7 impact prior to PCV10/13 introduction. Sites were categorized as no PCV7 impact (i.e., PCV7 not used), moderate PCV7 impact, and substantial PCV7 impact. Among the sites that used PCV7, PCV7 impact strata (moderate versus substantial) were defined by the modeled site-specific reduction in PCV7-type IPD among children aged <5 years in the year prior to PCV10/13 introduction. Sites with PCV7-type IRRs ≤0.05 (i.e., at least a 95% reduction in PCV7-type IPD) by the year prior to PCV10/13 introduction were defined as having substantial PCV7 impact and sites with PCV7-type IRRs >0.05 in the year prior to PCV10/13 introduction were defined as having moderate PCV7 impact. The ≤0.05 cut-off was chosen in consultation with the PSERENADE Technical Advisory Group as an arbitrary point to dichotomize sites with near elimination of PCV7-type IPD versus sites with reductions in but some amount of remaining PCV7-type IPD. Review of the data (shown in **Supplementary Figure 1.2**) showed a small gap between the substantial and moderate PCV7 impact sites and consensus was reached that ≤0.05 point was a reasonable cut-off. We used three PCV7 impact groups to reflect these three scenarios (no use, some impact but remaining disease, and near elimination) and did not combine the substantial and moderate PCV7 impact groups to capture the differences in IRR curves between these sites during the early years of PCV10/13 introduction. Although there is heterogeneity in the reduction observed in PCV7-type IPD among the moderate PCV7 impact sites, we did not further stratify these sites in order to have a reasonable number of strata for displaying results and to generally have enough sites in each strata to produce results.

Supplementary Figure 1.2 Site-specific modeled PCV7-type IPD IRRs for children aged <5 years in the last year of PCV7-use prior to PCV10/13 introduction compared to the pre-PCV period. Data shown for sites that used PCV7, were included in VT/NVT analyses, and had both pre- and post-PCV data available.

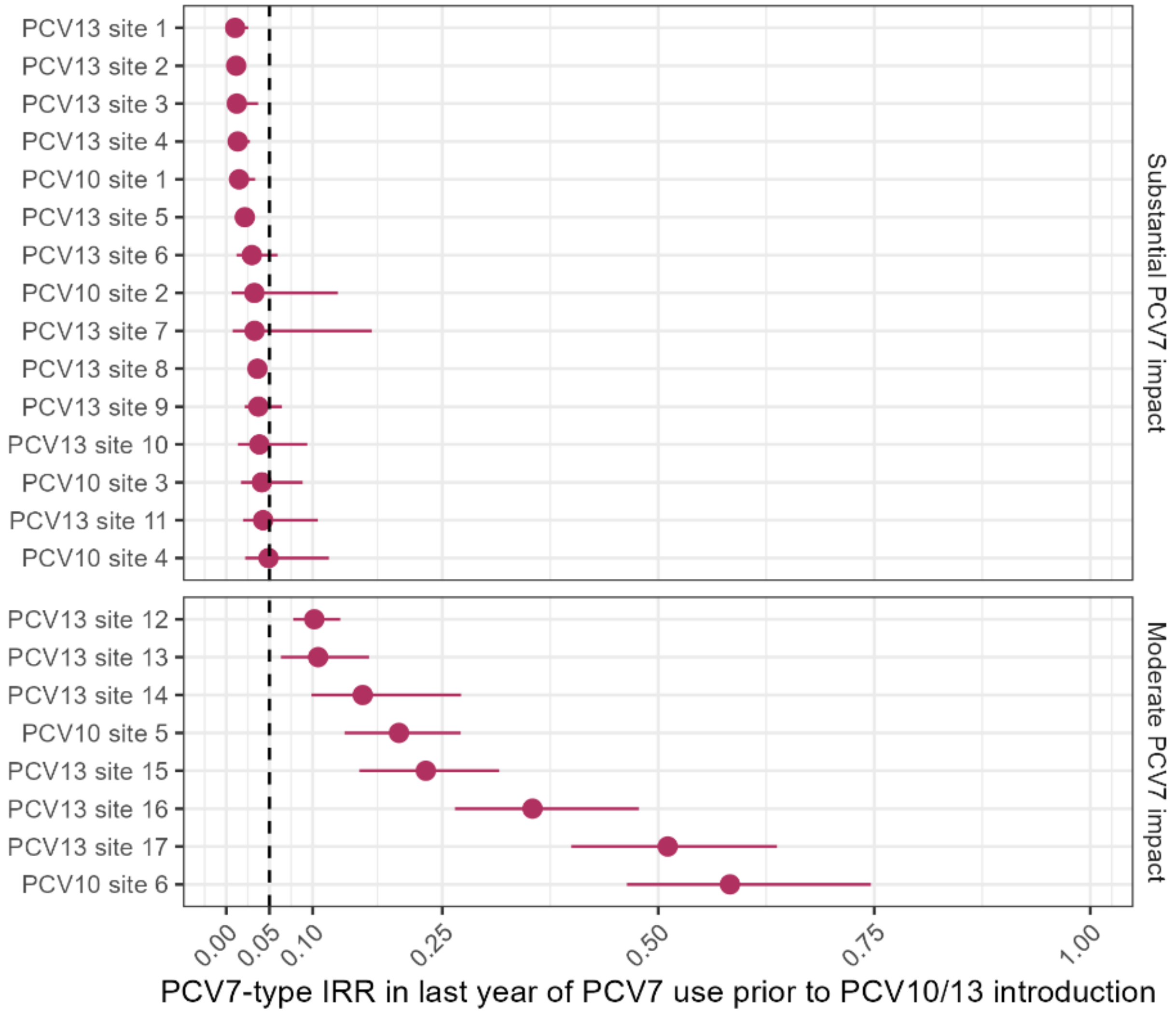

Supplementary Methods 1.3 Estimating annual IPD IRRs comparing the pre-PCV period to each post-PCV10/13 year using a Bayesian multi-level, mixed-effects Poisson regression

A Bayesian multi-level, mixed-effects Poisson regression was used to estimate annual IPD IRs across all years of available data for each site using the MCMCglmm package in R [1]. The model included an offset for population denominator and random effects for all of the site-specific regression coefficients, which allowed for heterogeneity among sites in the shapes of their IR curves. The regression identified commonalities within and across sites in the direction of change over time and smoothed out observed annual variability associated with rare outcomes. Data points from the same site were treated as repeated measures over time. Sites with small case counts or few years of data had less influence on the estimated overall average curve than sites with larger case counts and/or many years of data. We ran the model with 20,000 MCMC iterations, a burn-in of 10,000, and a thinning interval of 40, resulting in 250 samples. We used default conjugate priors for the regression coefficients and for the elements in the variance matrix for the random effects.

To allow an abrupt hinge in the curve to capture the change in the IR for each site's introduction of each PCV, a site-specific non-linear break (a pre-/post-PCV interaction term with the linear function of time) was included in the model. For children aged <5 years, the non-linear break occurred one year prior to each PCV introduction (i.e., captured the change from the year prior to introduction to the year of PCV introduction). For individuals aged ≥5 years for which indirect PCV impacts are not immediate, the non-linear break occurred on the year of each PCV introduction (i.e., captured the change from the year of PCV introduction to the following year).

Cubic splines knots that allow a smooth change in the slope were included for each site at years +1 and +3 following each PCV introduction (i.e., second and fourth year of PCV use) to allow for flexibility in the IR of IPD over time for each site following each PCV introduction. The +1 and +3 year timing of the cubic spline knots were chosen in the early post-PCV period where IR changes were expected to be the most dramatic and heterogenous across sites. The number of knots was limited by the number of degrees of freedom that the model could accommodate. During model development, versions of the model with cubic spline terms placed at other years were explored and with input from site investigators and the Technical Advisory Group it was determined that years +1 and +3 made sense epidemiologically and generally fit the data best.

For sites with both pre- and post-PCV data, we modeled the pre-PCV IRs as a linear slope. Assuming that any changes in pre-PCV trends would stabilize after three years absent PCV, a counterfactual IR was estimated by extending the pre-PCV IR slope linearly for three years post-PCV introduction, then flattening the slope to zero after 3 years. The choice to extend the pre-PCV slope for three years was agreed upon with the site investigators and Technical Advisory Group to be a reasonable assumption even if changes in the IR hypothetically would have continued longer or flattened out earlier for some individual sites. The site-specific modeled IR and counterfactual IR were used to estimate site-specific annual IRRs in each post-PCV year with the mean of the posterior distribution of rate ratios (the modeled IR in each post-PCV year relative to the counterfactual IR in each post-PCV year) for each site. CIs were estimated using the 2.5 and 97.5 percentiles of the posterior distribution of the IRs.

1. Hadfield, J.D. MCMC Methods for Multi-Response Generalized Linear Mixed Models: The MCMCglmm R Package. *J. Stat. Softw.* **2010**, *33*, 1–22, doi:10.18637/jss.v033.i02.

2. Description of Data

Supplementary Table 2.1 Sites included in PSERENADE evaluated for the IPD incidence analyses by age group (Y= Yes, included; N= No, excluded).

Eight sites were excluded from all analyses due to concurrent PCV10/13 use (n=4), surveillance data being restricted to pneumococcal meningitis (n=2), or biases in the surveillance system or changes other than PCV introductions over time affecting estimates of IPD incidence that could not be accounted for (n=2). Additional age groups from sites included in all-IPD analyses were excluded from all VT and NVT analyses due to <50% of cases being serotyped and additional age groups from included sites did not meet all inclusion criteria and were excluded from specific analyses.

Site	<5 years		5-17 years		18-49 years		50-64 years		≥65 years	
	All-ST	VT/NVT	All-ST	VT/NVT	All-ST	VT/NVT	All-ST	VT/NVT	All-ST	VT/NVT
Australia (Non-Indigenous)	Y	Y	Y	Y	Y	Y	Y	Y	Y	Y
Northern Territory, Australia	Y	Y[1]	Y	Y[2]	Y	Y	Y	Y	Y	Y
Mirzapur, Bangladesh	N[3]	N[3]	N[3]	N[3]	--	--	--	--	--	--
Belgium	Y	Y	Y	Y	--	--	--	--	--	--
Brazil	N[4]	N[4]	N[4]	N[4]	N[4]	N[4]	N[4]	N[4]	N[4]	N[4]
Alberta, Canada	Y	Y	Y	Y	Y	Y	Y	Y	Y	Y
Ontario, Canada	Y	Y	Y	Y	Y	Y	Y	Y	Y	Y
Quebec (excluding Nunavik), Canada (PCV13)	Y	Y[2]	Y	Y	Y	Y	Y	Y	Y	Y
Quebec (excluding Nunavik), Canada (PCV10)	Y	Y	Y	Y	Y	Y	Y	Y	Y	Y
Quebec-Nunavik, Canada (PCV13)	Y	Y	Y	N[5]	Y	N[5]	Y	N[5]	Y	N[5]
Quebec-Nunavik, Canada (PCV10)	Y	Y	Y	N[5]	Y	N[5]	Y	N[5]	Y	N[5]
Metropolitan Region, Chile	Y	Y	Y	Y	Y	Y	Y	Y	Y	Y
Non-Metropolitan Regions, Chile	Y	Y	Y	Y	Y	Y	Y	Y	Y	Y
Czech Republic	N[6]	N[6]	N[6]	N[6]	N[6]	N[6]	N[6]	N[6]	N[6]	N[6]
Denmark	Y	Y	Y	Y	Y	Y	Y	Y	Y	Y
Fiji	Y	N[5]	N[3]	N[3]	N[3]	N[3]	N[3]	N[3]	N[3]	N[3]
Finland	Y	Y	Y	Y	Y	Y	Y	Y	Y	Y
France	Y	Y	Y	Y	Y	Y	Y	Y	Y	Y
Basse, The Gambia	Y	Y	Y	Y[1, 7]	N[3]	N[3]	N[3]	N[3]	N[3]	N[3]
Germany	Y	Y	Y	Y	Y	Y	Y	Y	Y	Y
Greece	N[4]	N[4]	N[4]	N[4]	N[4]	N[4]	N[4]	N[4]	N[4]	N[4]
Hong Kong	Y	Y[2]	Y	Y[2, 8]	Y	Y	Y	Y[2]	Y	Y
Iceland	Y	Y	Y	Y[2]	Y	Y	Y	Y	Y	Y
Ireland	Y	Y	Y	Y	Y	Y	Y	Y	Y	Y
Israel	Y	Y	Y	Y	Y	Y	Y	Y	Y	Y

Italy	Y	Y	Y	Y	Y	Y	Y	Y	Y	Y
Japan	N[3]	N[3]	N[3]	N[3]	Y	Y[2]	Y	Y	Y	Y
Asembo, Kenya	Y	Y[7]	Y	Y[1, 7]	Y	Y[2]	N[3]	N[3]	N[3]	N[3]
Kibera, Kenya	Y	N[5]	Y	N[5]	Y	N[5]	N[3]	N[3]	N[3]	N[3]
Kilifi, Kenya	Y	Y	Y	Y	Y	Y[2]	Y	Y	Y	Y[1, 7, 8]
Latvia	N[3]	N[3]	N[3]	N[3]	Y	Y	Y	Y[2]	Y	Y[2]
Blantyre District, Malawi	N[3]	N[3]	N[3]	N[3]	N[3]	N[3]	N[3]	N[3]	N[3]	N[3]
Mongolia	Y	N[5]	--	--	--	--	--	--	--	--
Grand Casablanca, Morocco	Y	Y	Y	Y[1, 7]	Y	Y	--	--	N[3]	N[3]
Netherlands	Y	Y	Y	Y[2]	Y	Y	Y	Y	Y	Y
New Zealand	Y	Y	Y	Y	Y	Y	Y	Y	Y	Y
Norway	Y	Y	Y	Y	Y	Y	Y	Y	Y	Y
Poland	N[6]	N[6]	N[6]	N[6]	N[6]	N[6]	N[6]	N[6]	N[6]	N[6]
Singapore	Y	Y[8]	Y	Y[2,8]	Y	Y	Y	Y	Y	Y
Slovakia	N[6]	N[6]	N[6]	N[6]	N[6]	N[6]	N[6]	N[6]	N[6]	N[6]
Slovenia	Y	Y	Y	Y[1]	Y	Y	Y	Y	Y	Y
South Africa	Y	Y	Y	Y	Y	Y	Y	Y	Y	Y
Catalonia, Spain	Y	Y	Y	Y	Y	Y	Y	Y	Y	Y
Madrid, Spain	Y	Y	Y	Y	Y	Y	Y	Y	Y	Y
Navarra, Spain	Y	Y	Y	Y[2]	Y	Y	Y	Y	Y	Y
Sweden	N[6]	N[6]	N[6]	N[6]	N[6]	N[6]	N[6]	N[6]	N[6]	N[6]
Switzerland	Y	Y	Y	Y	Y	Y	Y	Y	Y	Y
England & Wales, UK	Y	Y	Y	Y	Y	Y	Y	Y	Y	Y
Scotland, UK	Y	Y	Y	Y	Y	Y	Y	Y	Y	Y
Active Bacterial Core surveillance (ABCs), USA[9]	Y	Y	Y	Y	Y	Y	Y	Y	Y	Y
Alaska, USA[9]	Y	Y	Y	Y	Y	Y	Y	Y	Y	Y
California, USA[9]	Y	Y	Y	Y[2]	Y	Y	Y	Y	Y	Y
Massachusetts, USA	Y	Y	Y	Y	--	--	--	--	--	--
Southwest, USA (Indigenous)[9]	Y	Y	Y	Y	Y	Y	Y	Y	Y	Y
Utah, USA[9]	Y	Y[8]	--	--	--	--	--	--	--	--

[1] Zero ST3 cases in all years

[2] Zero ST6A cases in all years

[3] Biases in surveillance system over time or other non-PCV changes that could not be accounted for in analyses

[4] Population-based surveillance data only available for pneumococcal meningitis cases

[5] Low proportion of cases serotyped or very few absolute cases (i.e., ≤3) and not all cases serotyped

[6] Concurrent PCV10/13 use.

[7] Zero ST19A cases in all years

[8] Zero ST1, 5, & 7F cases in all years

[9] For all U.S. sites where data were available, analyses were restricted to only hospitalized IPD cases (excluding outpatient cases).
-- Data not provided or not available

Supplementary Table 2.2 Description of surveillance data included in analyses by PCV product and PCV7-impact strata

	Total Number (Median, Range)				
	Pre- and post-PCV data[1]			Post-PCV data only[2]	Total
	No PCV7 Impact	Moderate PCV7 Impact	Substantial PCV7 Impact[3]		
**PCV10 Sites**					
<5 years					
Surveillance Sites	6	2	4	2	14
Countries	4	2	3	2	10
Cases	2128 (300.0, 30-995)	3971 (1985.5, 1372-2599)	2541 (322.5, 40-1856)	371 (185.5, 22-349)	9011 (347.5, 22-2599)
Surveillance Years	84 (12, 9-24)	26 (13, 12-14)	55 (13, 11-18)	9 (4, 3-6)	174 (12, 3-24)
5-17 years					
Surveillance Sites	6	2	4	1	13
Countries	4	2	3	1	9
Cases	608 (67.5, 31-260)	1027 (513.5, 393-634)	725 (155.0, 17-398)	151 (151.0, 151-151)	2511 (151.0, 17-634)
Surveillance Years	84 (12, 9-24)	26 (13, 12-14)	55 (13, 11-18)	6 (6, 6-6)	171 (12, 6-24)
18-49 years					
Surveillance Sites	6	-	4	4	14
Countries	4	-	3	3	10
Cases	3004 (123.5, 36-2352)	-	3840 (925.5, 24-1965)	1438 (361.0, 132-584)	8282 (306.5, 24-2352)
Surveillance Years	77 (10, 9-24)	-	55 (13, 11-18)	22 (6, 4-7)	154 (10, 4-24)
50-64 years					
Surveillance Sites	4	-	4	4	12
Countries	4	-	3	3	10
Cases	3684 (396.5, 16-2875)	-	4278 (1008.5, 11-2250)	1478 (393.5, 148-543)	9440 (393.5, 11-2875)
Surveillance Years	54 (12, 6-24)	-	55 (13, 11-18)	22 (6, 4-7)	131 (10, 4-24)
≥65 years					
Surveillance Sites	4	-	4	4	12
Countries	4	-	3	3	10
Cases	6210 (826.0, 10-4548)	-	8708 (1663.0, 14-5368)	2224 (491.0, 162-1080)	17142 (502.5, 10-5368)
Surveillance Years	56 (12, 8-24)	-	55 (13, 11-18)	22 (6, 4-7)	133 (10, 4-24)
**PCV13 Sites**					
<5 years					
Surveillance Sites	2	6	12	10	31
Countries	2	6	6	8	20
Cases	180 (90.0,	26707 (3192.5,	25108 (529.0,	7860 (403.0,	59977 (622.0,

	77-103)	758-11594)	44-10709)	78-2536)	44-11594)
Surveillance Years	14 (7, 5-9)	96 (16, 14-19)	223 (18, 12-27)	101 (10, 5-16)	445 (14, 5-27)
5-17 years					
Surveillance Sites	1	6	11	9	28
Countries	1	6	6	8	19
Cases	28 (28.0, 28-28)	9551 (801.0, 342-4672)	9492 (230.0, 15-4123)	2486 (183.0, 44-652)	21626 (309.5, 15-4672)
Surveillance Years	5 (5, 5-5)	96 (16, 14-19)	196 (18, 10-27)	93 (10, 5-16)	401 (14, 5-27)
18-49 years					
Surveillance Sites	1	4	12	9	27
Countries	1	4	6	8	18
Cases	88 (88.0, 88-88)	44418 (8696.0, 2284-24742)	61531 (1511.5, 20-27576)	10258 (855.0, 92-2894)	116510 (1384.0, 20-27576)
Surveillance Years	5 (5, 5-5)	66 (16, 14-19)	219 (18, 10-27)	78 (9, 2-12)	379 (14, 2-27)
50-64 years					
Surveillance Sites	-	4	12	9	26
Countries	-	4	6	8	17
Cases	-	27710 (4644.0, 2696-15726)	57518 (1638.5, 11-23510)	12575 (939.0, 164-4924)	98203 (1461.0, 11-23510)
Surveillance Years	-	66 (16, 14-19)	219 (18, 10-27)	78 (9, 2-12)	374 (14, 2-27)
≥65 years					
Surveillance Sites	-	4	12	9	26
Countries	-	4	6	8	17
Cases	-	59393 (8817.5, 2250-39508)	101875 (2818.0, 10-49475)	30566 (2072.0, 371-12131)	192442 (2640.5, 10-49475)
Surveillance Years	-	66 (16, 14-19)	219 (18, 10-27)	78 (9, 2-12)	374 (14, 2-27)

[1] Data included in analysis steps 1-3 (site-specific modeling of IR and IRR curves and estimation of all-site weighted average IRRs)
[2] Data included only in analysis step 1 (site-specific modeling of post-PCV IR curves)
[3] Pre-PCV and PCV7 years of data from Quebec sites included in both PCV10 and PCV13 models

Supplementary Table 2.3 Description of surveillance data included in analysis by site

Site	PCV Product[1]	PCV7-Impact Strata[2]	PCV10/13 Schedule[1]	PCV 10/13 Catch-up	Mean PCV 10/13 Uptake[3]	Proportion VT in Pre-Period[4] (%)	Proportion of Cases Serotyped[5] (%)	No. of surveillance years (Pre, PCV7, PCV10/13) by age (years)					No. of cases by age (years)				
								<5	5-17	18-49	50-64	≥65	<5	5-17	18-49	50-64	≥65
Asembo, Kenya	PCV10	No impact	3+0	Y	86	66.7	91.3	1, - 8	1, - 8	1, 0 8	- - -	- - -	30	42	58	-	-
Kibera, Kenya	PCV10	No impact	3+0	N	87	--	--	2, - 8	2, - 8	2, 0 8	- - -	- - -	44	40	36	-	-
Kilifi, Kenya	PCV10	No impact	3+0	Y	83	75.1	99.8	11, - 6	11, - 6	4, 0 6	3, - 3	3, - 5	427	142	40	16	10
Finland	PCV10	No impact	2+1	N	95	79.0	96	6, - 8	6, - 8	6, 0 8	6, - 8	6, - 8	995	260	2352	2875	4548
Iceland	PCV10	No impact	2+1	N	89	83.1	86.8	16, - 8	16, - 8	16, 0 8	16, - 8	16, - 8	173	31	189	179	399
Slovenia	PCV10	No impact	2+1	N	55	74.4	98.4	6, - 4	6, - 4	6, 0 4	6, - 4	6, - 4	459	93	329	614	1253
Metropolitan Region, Chile	PCV10	Moderate	2+1	N	97	75.7	94.5	7, 2, 5	7, 2, 5	- - 4	- - 4	- - 4	2599	634	284	270	376
New Zealand	PCV10	Moderate	3+1	N	93	84.4	88.2	6, 3, 3	6, 3, 3	- 2, 3	- 2, 3	- 2, 3	1372	393	584	543	1080
Quebec (excluding Nunavik), Canada	PCV10	Substantial	2+1	N	97	84.3	98.7	5, 4, 2	5, 4, 2	5, 4, 2	5, 4, 2	5, 4, 2	1856	398	1965	1920	3284
Northern Territory, Australia	PCV10	Substantial	3+1	Y	88	71.8	91.6	7, 9, 2	7, 9, 2	7, 9, 2	7, 9, 2	7, 9, 2	299	156	523	97	42
Quebec-Nunavik, Canada	PCV10	Substantial	3+1	N	97	90.0	95.0	2, 7, 2	2, 7, 2	2, 7, 2	2, 7, 2	2, 7, 2	40	17	24	11	14
Netherlands	PCV10	Substantial	2+1/3+1	N	95	76.9	99.9	2, 5, 8	2, 5, 8	2, 5, 8	2, 5, 8	2, 5, 8	346	154	1328	2250	5368
Fiji	PCV10	NA	3+0	N	88	NA	--	- - 3	- - -	- - -	- - -	- - -	22	-	-	-	-
Non-Metropolitan Regions, Chile	PCV10	NA	2+1	N	97	NA	95.4	- - 6	- - 6	- - 6	- - 6	- - 6	349	151	438	517	606
Latvia	PCV10	NA	2+1/3+1	N	91	NA	84.6	- - -	- - -	- - 7	- - 7	- - 7	-	-	132	148	162
Grand Casablanca, Morocco	PCV13	No impact	2+1	N	NA[3]	85.7	80.4	4, - 1	4, - 1	4, - 1	- - -	- - -	103	28	88	-	-

Site	PCV Product[1]	PCV7-Impact Strata[2]	PCV10/13 Schedule[1]	PCV 10/13 Catch-up	Mean PCV 10/13 Uptake[3]	Proportion VT in Pre-Period[4] (%)	Proportion of Cases Serotyped[5] (%)	No. of surveillance years (Pre, PCV7, PCV10/13) by age (years)					No. of cases by age (years)				
								<5	5-17	18-49	50-64	≥65	<5	5-17	18-49	50-64	≥65
Mongolia	PCV13	No impact	2+1	Y	93	--	--	6, - 3	- - -	- - -	- - -	- - -	77	-	-	-	-
Denmark	PCV13	Moderate	2+1	N	91	90.5	96.4	8, 2, 9	8, 2, 9	8, 2, 9	8, 2, 9	8, 2, 9	1105	342	2640	4223	9938
Israel	PCV13	Moderate	2+1	N	95	89.3	85.4	7, 1, 8	7, 1, 8	- 1, 8	- 1, 8	- 1, 8	4034	860	855	939	2072
South Africa	PCV13	Moderate	2+1	Y	77	85.2	67.8	4, 2, 8	4, 2, 8	4, 2, 8	4, 2, 8	4, 2, 8	11594	4672	24742	5065	2250
Switzerland	PCV13	Moderate	2+1	Y	79	91.4	82.2	3, 5, 7	3, 5, 7	3, 5, 7	3, 5, 7	3, 5, 7	758	442	2284	2696	7697
France	PCV13	Moderate	2+1/3+1	N	93	90.5	NA[6]	2, 7, 9	2, 7, 9	2, 7, 9	2, 7, 9	2, 7, 9	6865	2493	14752	15726	39508
Germany	PCV13	Moderate	2+1/3+1	N	84	85.8	99.8	2, 3, 9	2, 3, 9	- - 8	- - 8	- - 8	2351	742	2894	4924	12131
Australia (Non-Indigenous)	PCV13	Substantial	3+0	Y	92	90.9	91	3, 6, 7	3, 6, 7	3, 6, 7	3, 6, 7	3, 6, 7	4866	1478	5465	5039	8931
England, UK	PCV13	Substantial	2+1	N	94	91.7	79.8	6, 4, 10	6, 4, 10	6, 4, 10	6, 4, 10	6, 4, 10	10709	4123	27576	23510	49475
Norway	PCV13	Substantial	2+1	N	93	95.7	93.4	2, 5, 7	2, 5, 7	2, 5, 7	2, 5, 7	2, 5, 7	622	230	1801	2607	5539
Quebec (excluding Nunavik), Canada	PCV13	Substantial	2+1	N	97	93.2	98.6	5, 4, 3	5, 4, 1	5, 4, 1	5, 4, 1	5, 4, 1	1816	358	1639	1634	2924
Scotland, UK	PCV13	Substantial	2+1	N	97	90.5	79.7	6, 4, 9	6, 4, 9	6, 4, 9	6, 4, 9	6, 4, 9	975	373	2381	2627	5339
ABCs, USA	PCV13	Substantial	3+1	Y	88	92.8	88.7	2, 10, 8	2, 10, 8	2, 10, 8	2, 10, 8	2, 10, 8	4629	2429	18571	18296	24519
Alaska, USA	PCV13	Substantial	3+1	Y	83	90.7	89.3	10, 9, 8	10, 9, 8	10, 9, 8	10, 9, 8	10, 9, 8	436	181	947	755	662
California, USA	PCV13	Substantial	3+1	Y	96	NA	80.7	5, 10, 8	- - -	5, 10, 8	5, 10, 8	5, 10, 8	277	-	1384	1643	2712
Quebec-Nunavik, Canada	PCV13	Substantial	3+1	N	97	100.0	90.9	2, 7, 3	2, 7, 1	2, 7, 1	2, 7, 1	2, 7, 1	44	15	20	11	10
Southwest, USA (Indigenous)	PCV13	Substantial	3+1	Y	82	81.0	84.4	5, 10, 9	5, 10, 9	5, 10, 9	5, 10, 9	5, 10, 9	287	153	760	566	619

Site	PCV Product[1]	PCV7-Impact Strata[2]	PCV10/13 Schedule[1]	PCV 10/13 Catch-up	Mean PCV 10/13 Uptake[3]	Proportion VT in Pre-Period[4] (%)	Proportion of Cases Serotyped[5] (%)	No. of surveillance years (Pre, PCV7, PCV10/13) by age (years)					No. of cases by age (years)				
								<5	5-17	18-49	50-64	≥65	<5	5-17	18-49	50-64	≥65
Alberta, Canada	PCV13	Substantial	2+1/3+1	N	88	87.7	98.5	2, 8, 8	2, 8, 8	2, 8, 8	2, 8, 8	2, 8, 8	239	87	728	580	547
Navarra, Spain	PCV13	Substantial	2+1/3+1	N	71	90.3	85.8	3, 6, 9	3, 6, 9	3, 6, 9	3, 6, 9	3, 6, 9	258	65	259	250	598
Basse, The Gambia	PCV13	NA	3+0	N	77	NA	95.4	- 2, 7	- 2, 7	- - -	- - -	- - -	285	44	-	-	-
Belgium	PCV13	NA	2+1	N	93	NA	98.1	- 5, 5	- 5, 4	- - -	- - -	- - -	2536	652	-	-	-
Ireland	PCV13	NA	2+1	N	91	NA	82.4	- 2, 8	- 2, 8	- 2, 8	- 2, 8	- 2, 8	392	144	631	696	1804
Italy	PCV13	NA	2+1	N	86	NA	68.9	- - 9	- - 9	- - 9	- - 9	- - 9	414	183	1111	1288	4471
Singapore	PCV13	NA	2+1	Y	84	NA	77.7	1, 2, 8	1, 2, 8	1, 2, 8	1, 2, 8	1, 2, 8	122	69	215	400	608
Hong Kong	PCV13	NA	3+1	N	98	NA	97.5	- - 5	- - 5	- - 5	- - 5	- - 5	127	86	119	192	371
Japan	PCV13	NA	3+1	N	98	NA	96.6	- - -	- - -	- - 2	- - 2	- - 2	-	-	92	164	543
Massachusetts, USA	PCV13	NA	3+1	Y	94	NA	79.5	- 8, 8	- 8, 8	- - -	- - -	- - -	894	313	-	-	-
Utah, USA	PCV13	NA	3+1	Y	88	NA	77.3	- - 7	- - -	- - -	- - -	- - -	78	-	-	-	-
Catalonia, Spain	PCV13	NA	2+1/3+1	N	70	NA	76.8	- 4, 8	- 4, 8	- 4, 8	- 4, 8	- 4, 8	1779	582	2442	2353	5247
Madrid, Spain	PCV13	NA	2+1/3+1	N	98	NA	85.2	- 3, 9	- 3, 9	- 3, 9	- 3, 9	- 3, 9	1062	306	1522	1255	2569
Ontario, Canada	PCV13	NA	2+1/3+1	Y	72	NA	91.6	- 3, 8	- 3, 8	- 3, 8	- 3, 8	- 3, 8	293	176	592	764	1358

NA: not applicable

1 PCV product and schedule used during years of data included in analyses. For sites that used PCV10/PCV13 sequentially, the first PCV10/13 product introduced was included in the analysis. Quebec sites are included in PCV10 models for first two years of PCV10 use and PCV13 models for years of data ≥5-7 years after switch from PCV10 to PCV13.

2 Prior PCV7 impact strata defined by modeled site-specific reduction in PCV7-ST IPD among children aged <5 years in the last year of PCV7 use (i.e., the year prior to PCV10/13 introduction) for sites with pre-PCV VT data: no PCV7 impact (i.e., PCV7 not used), substantial (PCV7-type IRR ≤0.05), or moderate (all others with pre-PCV VT data). “NA" indicates site had no pre-PCV IR data or inadequate serotyping (<50%) in the pre-PCV period for assessing the impact of PCV7 for categorizing PCV7-impact strata.

3 Annual PCV uptake estimates provided by the surveillance site for PCV10/13 years of data included in analyses. Uptake is for the primary series of PCV by 12 months of age (if available, for some sites up to 15 months of age), excluding the year of vaccine rollout. If unavailable, annual PCV uptake estimates provided by the surveillance site for the primary series plus the booster dose by 23 months of age, excluding the year of vaccine rollout used (N=7 sites: Alberta, Belgium, Denmark, Italy, South Africa, Switzerland, and Ontario). If PCV uptake data from the surveillance site unavailable, WHO and UNICEF Estimates of National Immunization Coverage (WUENIC) PCV uptake, excluding the year of vaccine rollout used (N=3 sites: Germany, Iceland, and Japan). Uptake not reported for Morocco, for which only the year of vaccine rollout was included in analyses (PCV13 primary series uptake was 23% in the year of introduction for Morocco).

4 Proportion of serotyped cases due to VT IPD (PCV10 STs for PCV10 sites and PCV13 STs for PCV13 sites) among children aged <5 years in the pre-PCV period. “NA" indicates site had no pre-PCV data and ‘--’ indicates pre-PCV data for site was included in all IPD analyses only (i.e., pre-PCV years of use were not included in any VT/NVT analyses).

5 Proportion of cases fully serotyped (single ST identified) across all age groups. “--" indicates site only included in all IPD analyses (i.e., excluded from all VT/NVT analyses).

6 For France, serotype distribution data from the National Reference Laboratory-Pneumococcus Regional Observatory (ORP-CRNP; sentinel surveillance) was applied to annual case estimates from Epibac (hospital laboratory network without serotype data). ORP-CRNP serotypes all IPD isolates from children 0–15 years of age and a systematic sample (1/6) of IPD cases in individuals >15 years of age.

Supplementary Figure 2.1 Number of IPD cases per site included in the analysis by region and age group

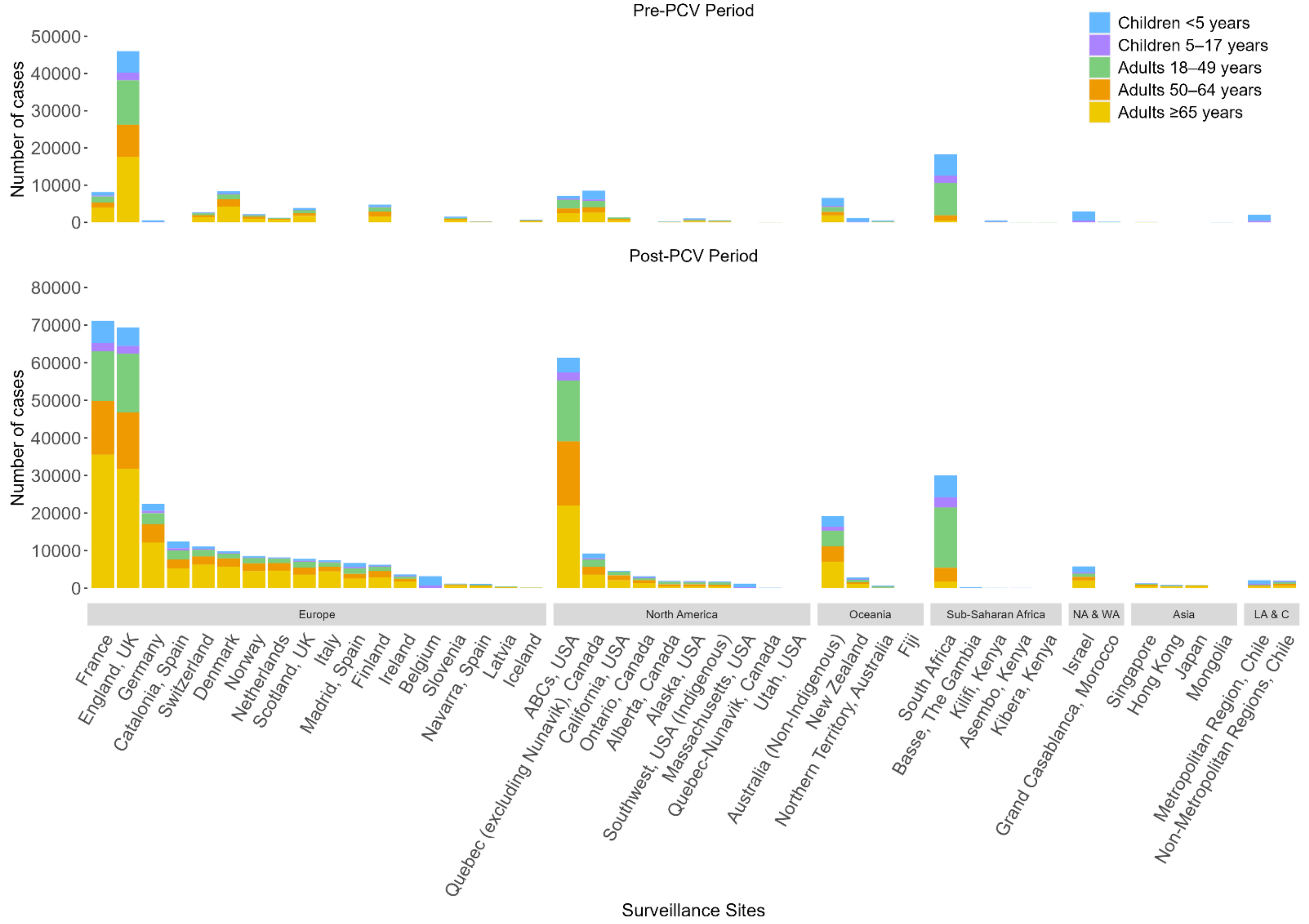

NA & WA – Northern Africa and Western Asia; LA & C – Latin America and the Caribbean
Not all age groups were included for all sites (Supplementary Table 2.1)
Analyses were done with minor changes to age groups for certain sites to align with availability of population denominators and age groups provided by sites in aggregate: the <5 years age group includes 0-5 years from Morocco; the 5-17 years age group included 5-14 years from Kilifi, Kenya, 5-15 years from Germany, 6-14 years from Morocco, and 5-19 years from Australia; and the 18-49 years age group includes 15-49 years from Japan and Kilifi, Kenya, 15-59 years from Morocco, 16-49 years from Germany, and 20-49 years from Australia

Supplementary Figure 2.2 All-site weighted average incidence rate ratios for all serotype IPD and vaccine/serotype specific IPD, comparing annual post-PCV10/13 incidence rate to the average pre-PCV incidence rate by age group for ages 5-64 years

2.2.1. 5-17 years

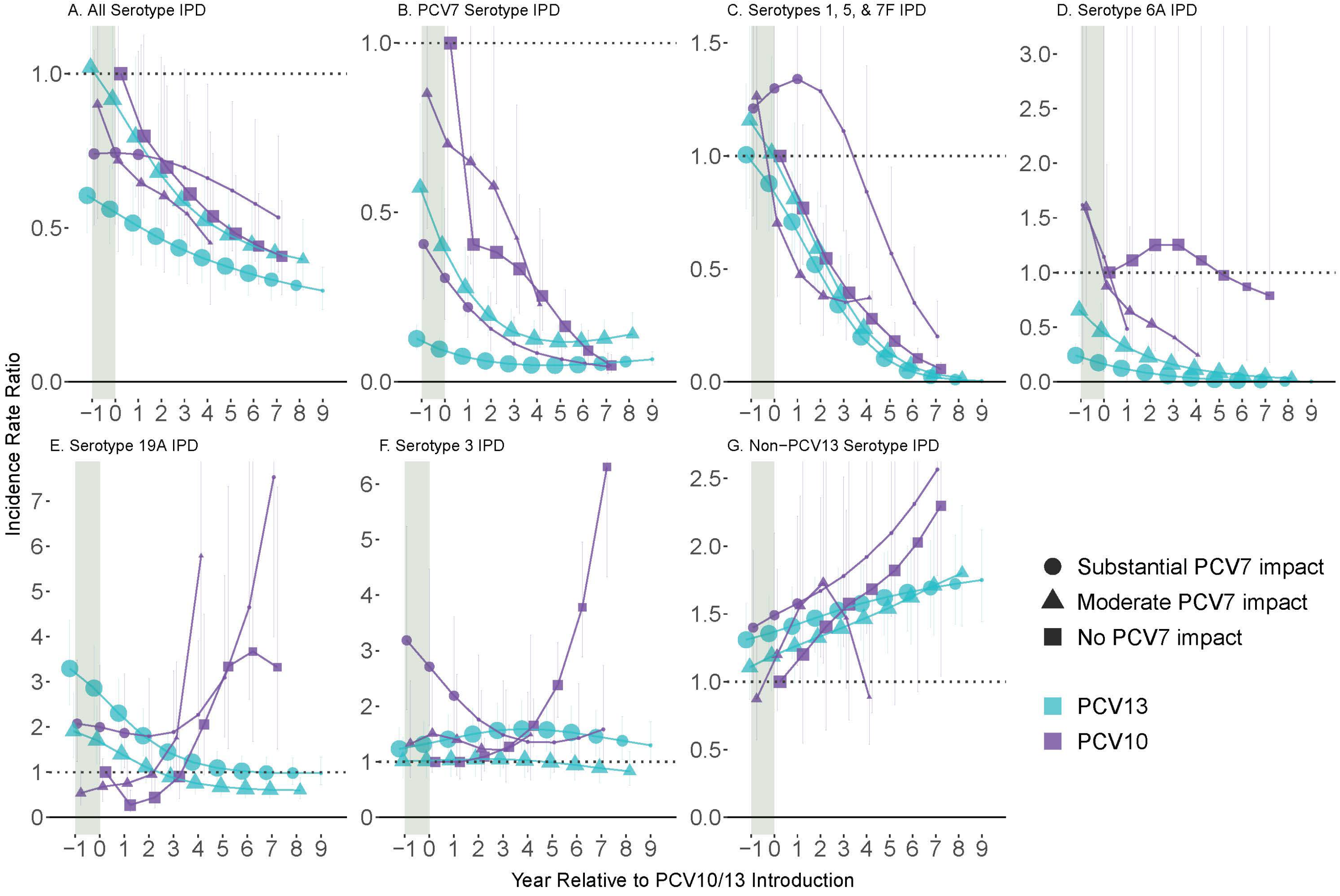

Children aged 5–17 years includes the cohort of children directly immunized with PCV7 or PCV10/13 after 5 years of PCV use.

2.2.2. 18-49 years

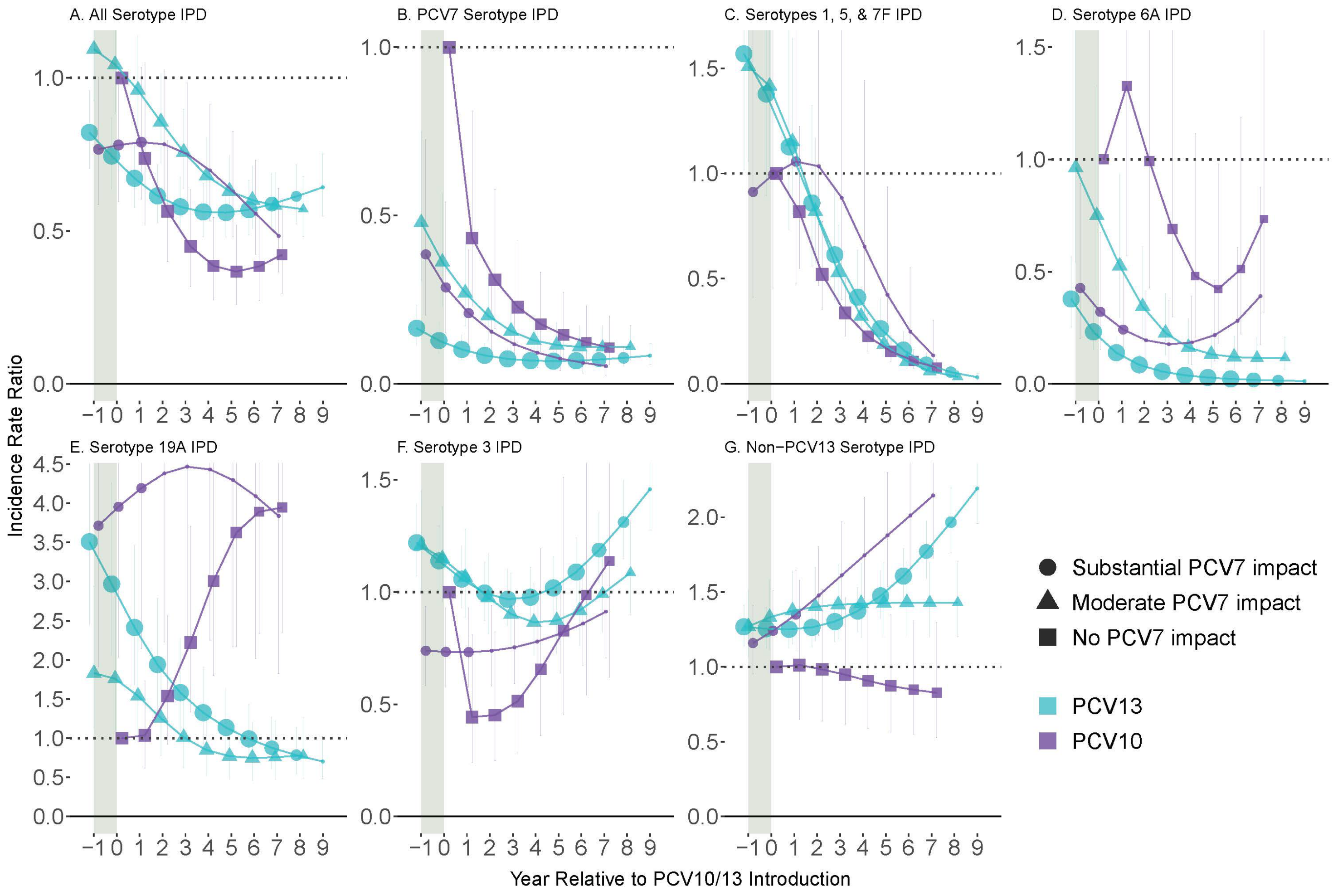

2.2.3. 50-64 years

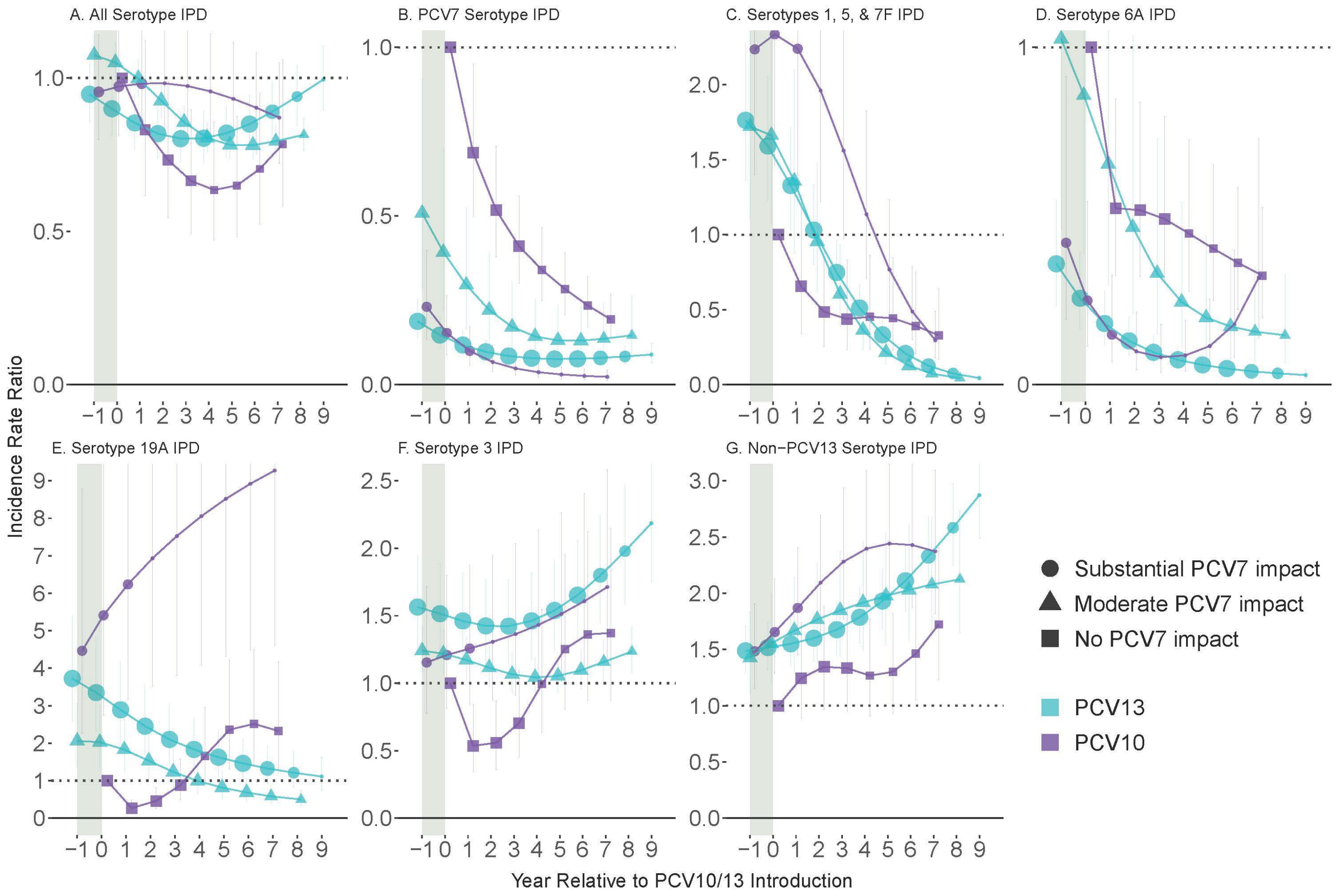

Supplementary Figure 2.3 Annual site-specific PCV uptake across years of data included in analyses

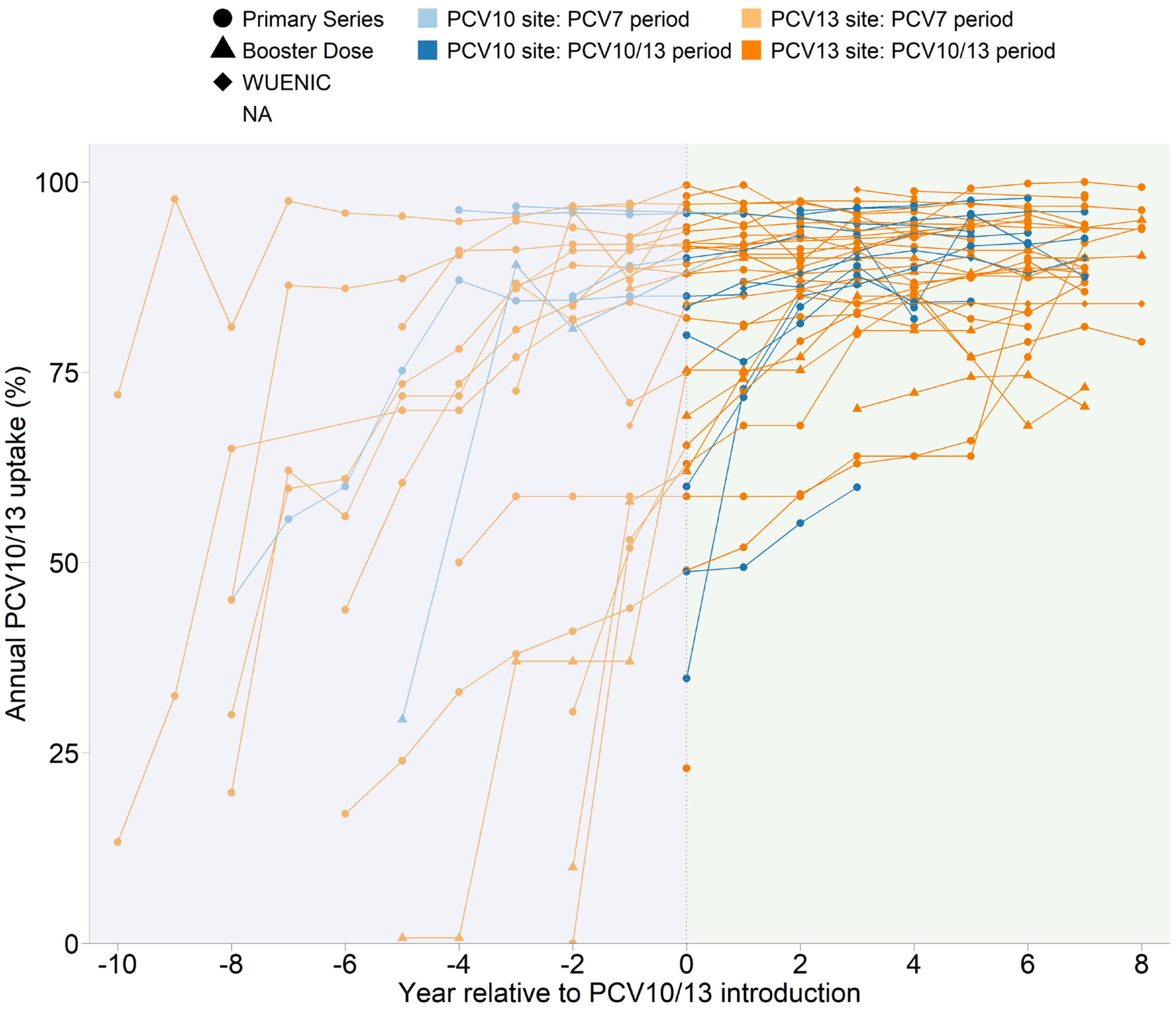

Annual PCV uptake estimates provided by the surveillance site for years of data included in analyses. Sites without PCV7 use start at year 0 (year of PCV10/13 introduction). Uptake is for the primary series of PCV by 12 months of age (if available, for some sites up to 15 months of age). If unavailable, annual PCV uptake estimates provided by the surveillance site for the primary series plus the booster dose by 23 months of age (N=7 sites: Alberta, Belgium, Denmark, Italy, South Africa, Switzerland, and Ontario). If PCV uptake data from the surveillance site unavailable, WHO and UNICEF Estimates of National Immunization Coverage (WUENIC) PCV uptake (N=3 sites: Germany, Iceland, and Japan).

Supplementary Data 2.1 ST6C Trends

Due primarily to small sample sizes pre-PCV, ST6C data were not robust enough to model IRRs for either children or adults for PCV10 or PCV13 sites and so only actual observed case data are shown (Supplementary Figure 2.3). In addition, 8.5% and 2.9% of ST6C cases in the pre-PCV10/13 period for children aged <5 years and adults aged ≥65 years, respectively, were estimated based on redistribution of serogroup 6 cases that were not fully serotyped as described in Supplementary Methods 1.1.

In children aged <5 years at PCV13 sites, after redistribution there were a total of 24 ST6C cases in the pre-PCV period, 151 in the PCV7 period, and 162 in the post-PCV13 period. ST6C generally increased following PCV7 introduction and stopped increasing or declined but was not eliminated after PCV13 introduction (Supplementary Figure 2.3, Panel B). At PCV10 sites, after redistribution there were 0 ST6C cases in the pre-PCV period, 16 in the PCV7 period, and 37 in the post-PCV10 period in children aged <5 years. ST6C IRs also increased following PCV7 introduction at PCV10 sites, but declines were not seen after PCV10 introduction (Supplementary Figure 2.3, Panel A).

In adults aged ≥65 years, general trends were similar to children. At PCV10 sites, after redistribution there were 36 ST6C cases in the pre-PCV period, 22 in the PCV7 period, and 334 in the post-PCV10 period; ST6C IRs tended to increase following PCV10 introduction (Supplementary Figure 2.3, Panel C). At PCV13 sites, there were 83 ST6C cases in the pre-PCV period, 965 in the PCV7 period, and 2933 in the post-PCV13 period in adults aged ≥65 years; ST6C IRs in general declined following PCV13 introduction (Supplementary Figure 2.3 Panel D).

Supplementary Figure 2.4 Observed ST6C IPD IRs for (A) children aged <5 years at PCV10 sites, (B) children aged <5 years at PCV13 sites, (C) adults aged ≥65 years at PCV10 sites and (D) adults aged ≥65 years at PCV13 sites. Plots on the right show area of left plot circled by black box zoomed in. Green vertical bar shows the year prior to PCV10/13 introduction to the year of PCV10/13 introduction; negatively numbered years left of the green vertical bar are a combination of pre-PCV and PCV7 period data. Each line is a different site.

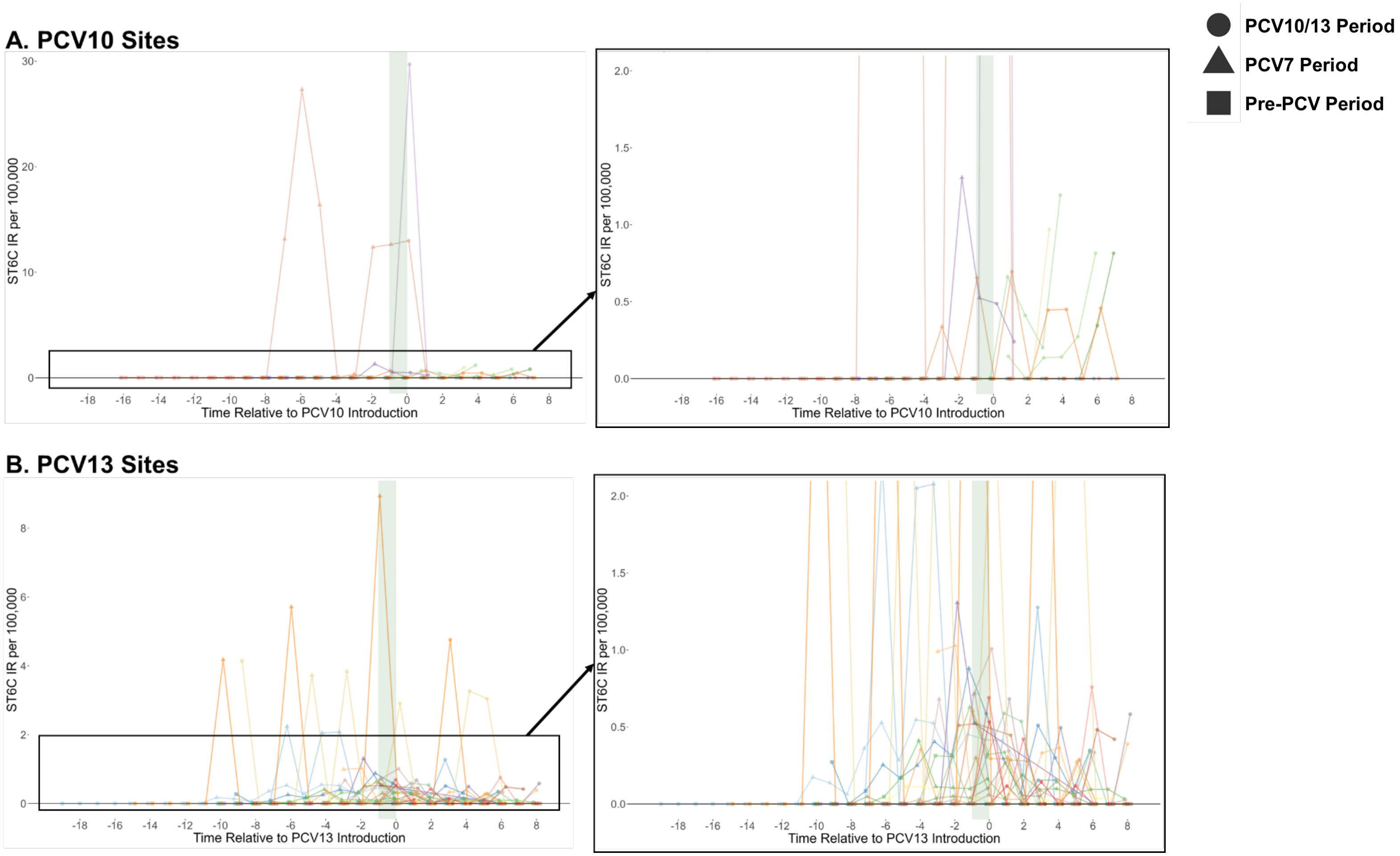

C. PCV10 Sites

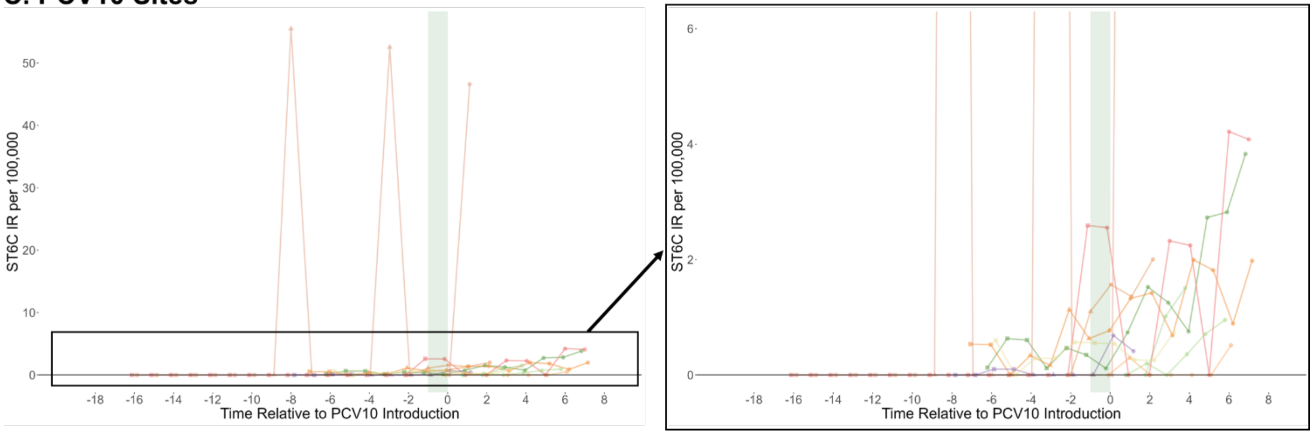

D. PCV13 Sites

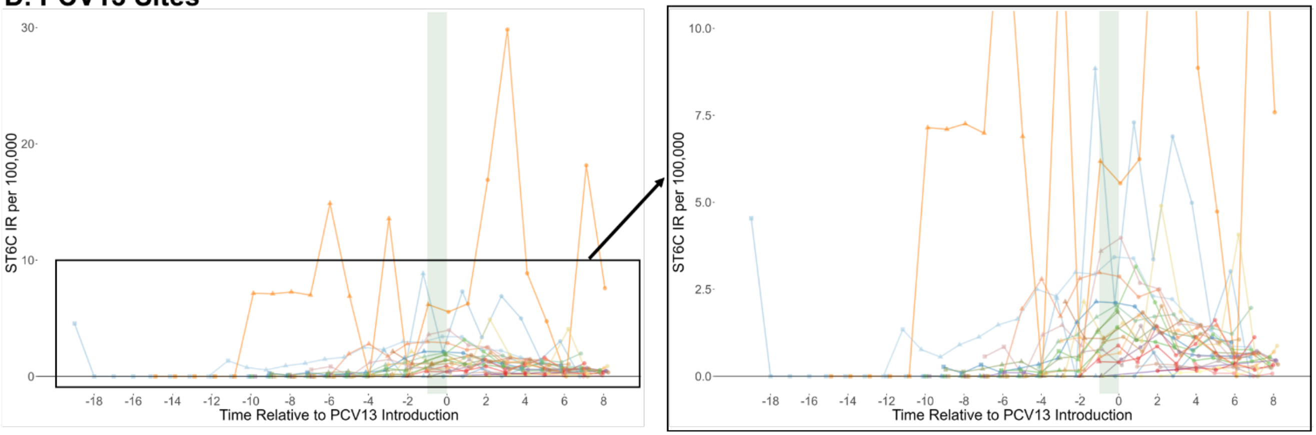

3. Modeled Results

Supplementary Figure 3.1 All-site weighted average IRRs for PCV13 ST IPD comparing the annual post-PCV10/13 IR to the average pre-PCV IR by age group

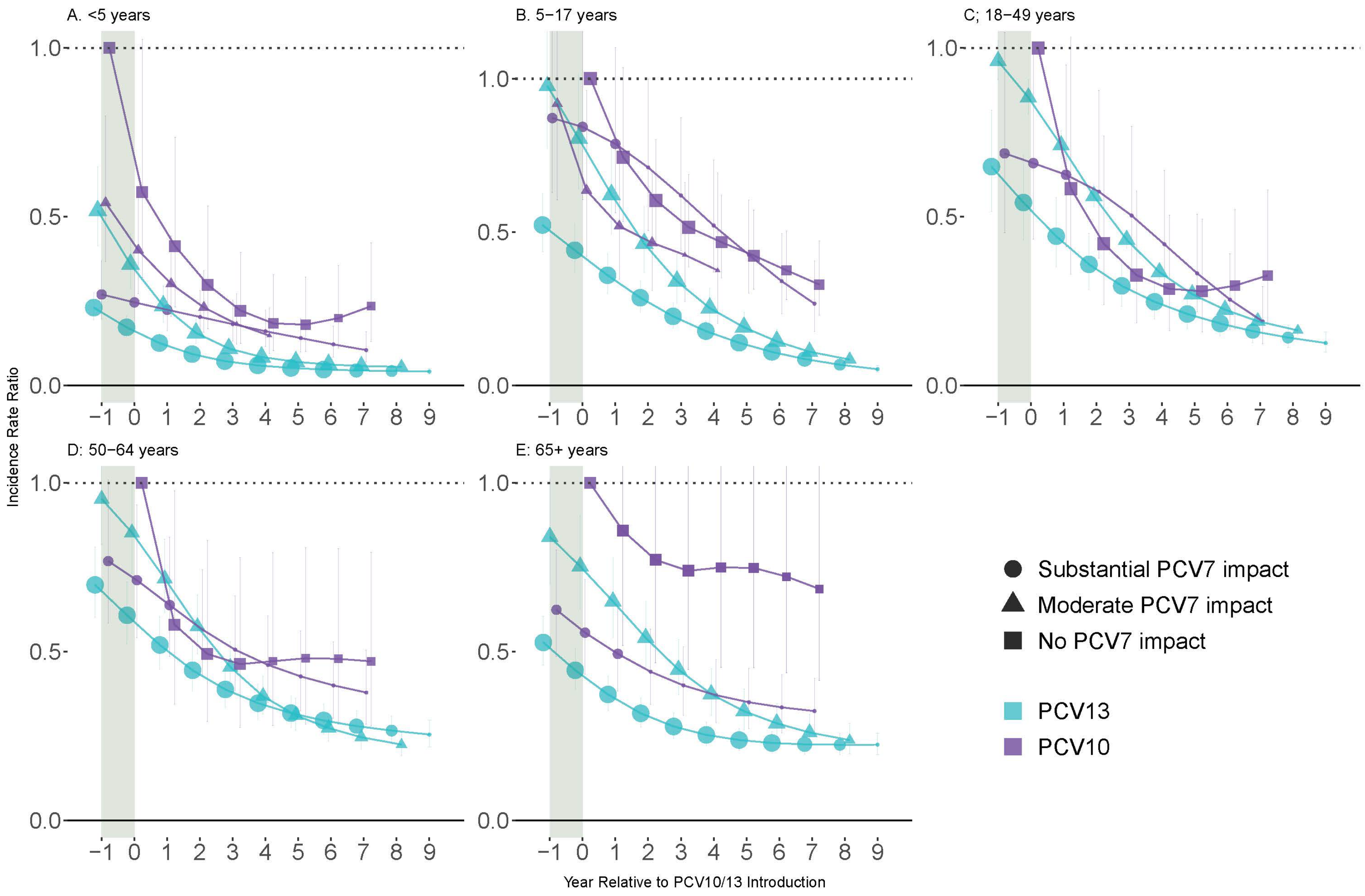

Supplementary Figure 3.2 All-site weighted average IRRs for PCV13 ST-minus-ST3 IPD comparing the annual post-PCV10/13 IR to the average pre-PCV IR by age group

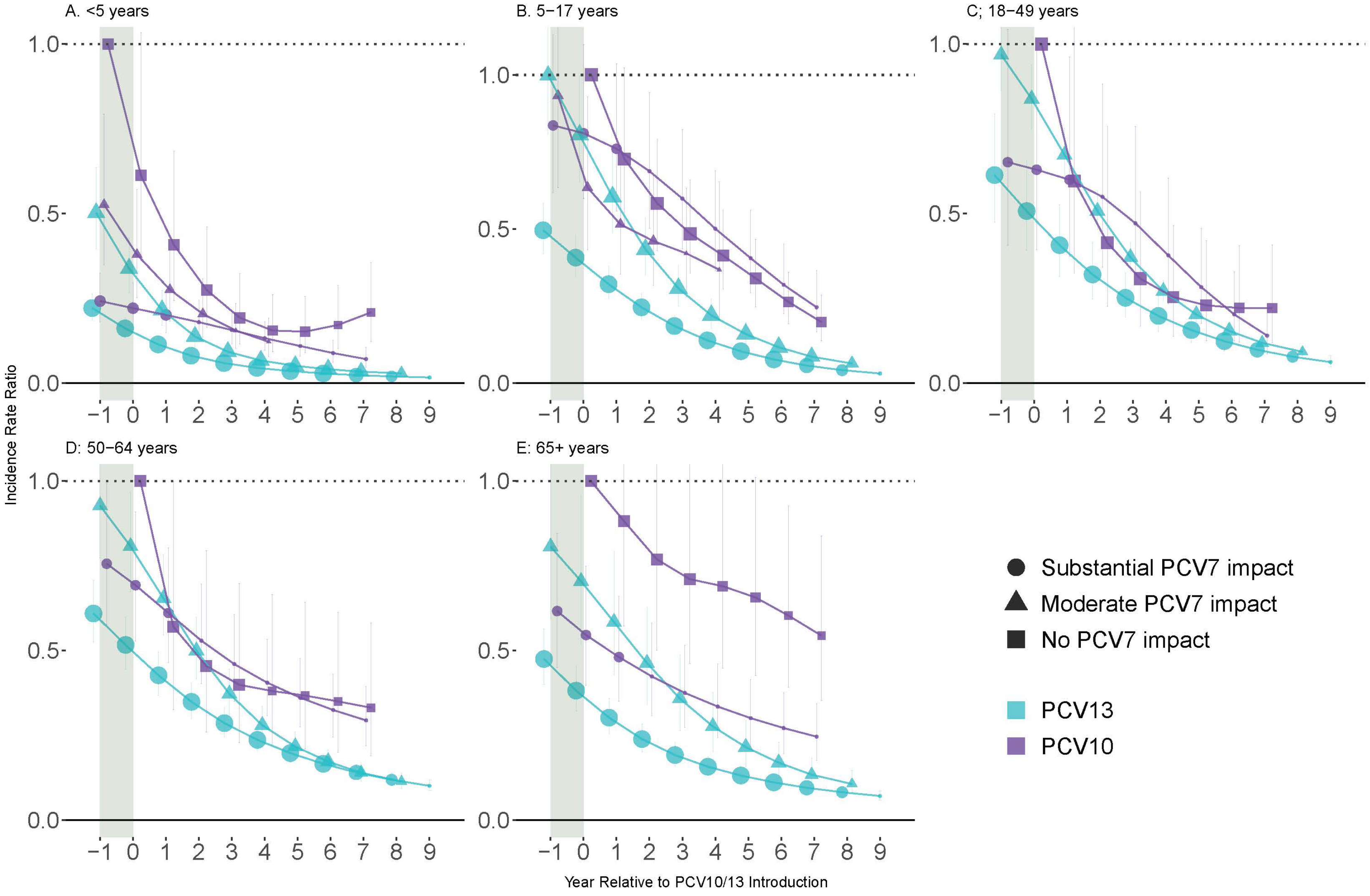

Supplementary Figure 3.3 Site-specific modeled all ST IPD IRRs for children aged <5 years

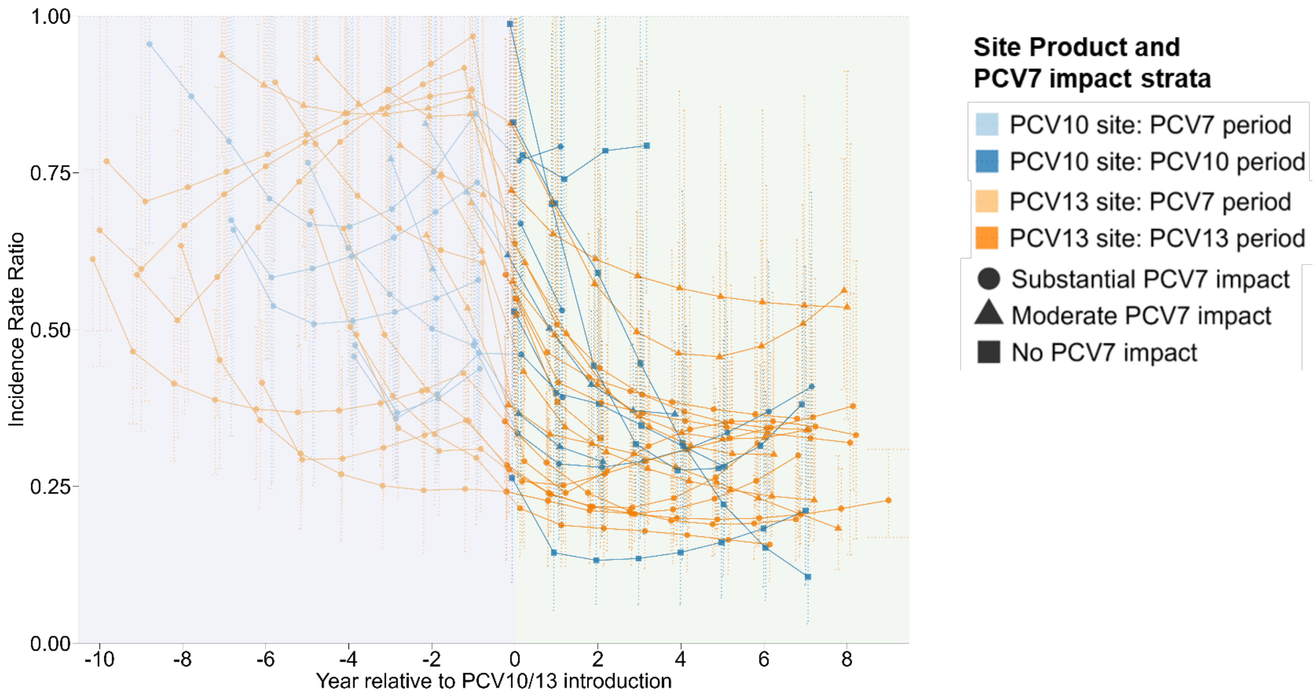

Supplementary Figure 3.4 Site-specific modeled all ST IPD IRRs for adults aged ≥65 years

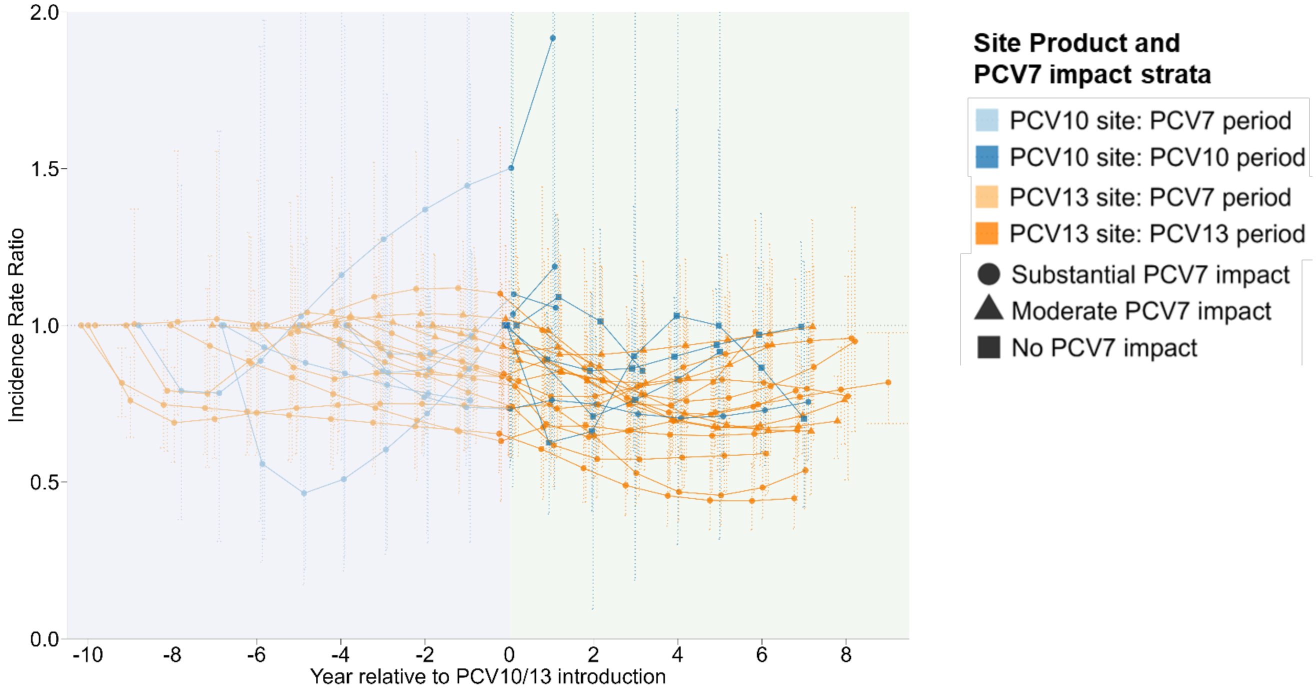

Negative years on x-axis (purple shaded area) show IRRs comparing PCV7 years of use (if any) relative to the pre-PCV IR and positive years (green shaded area) on x-axis show IRRs comparing PCV10/13 years of use relative to the pre-PCV IR

Supplementary Figure 3.5 Site-specific modeled ST19A IPD IRRs for children aged <5 years

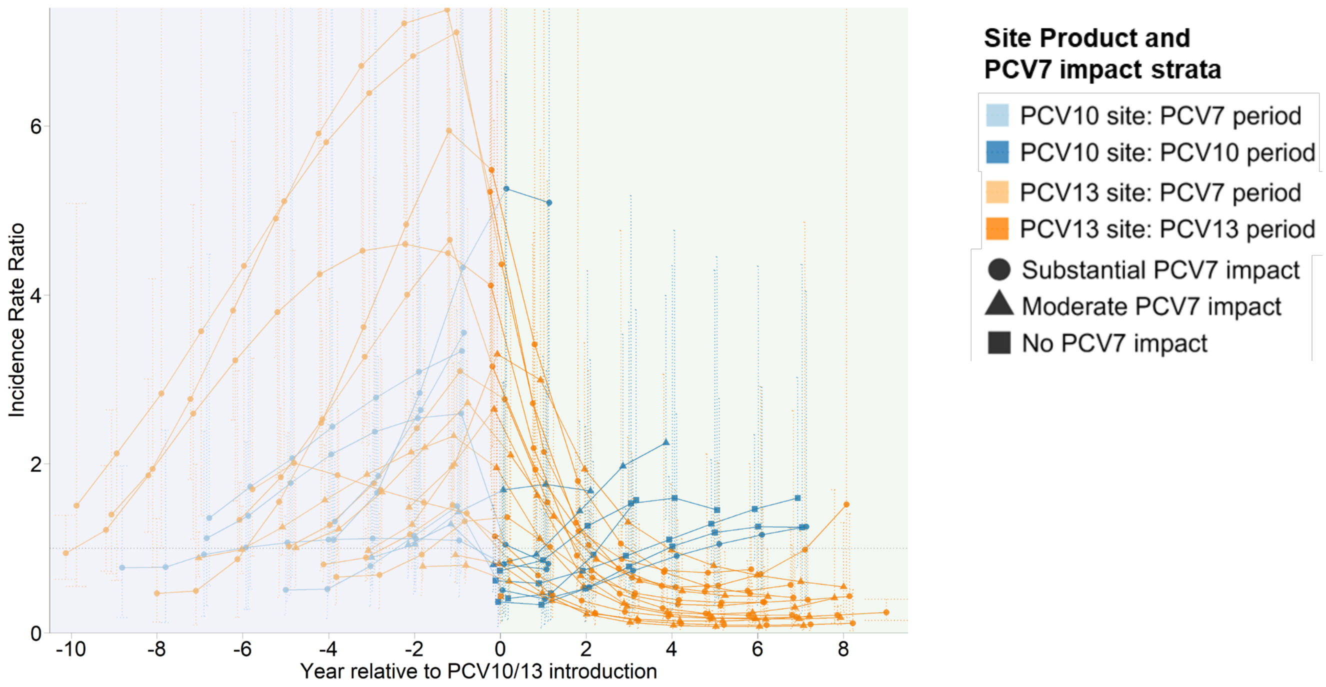

Supplementary Figure 3.6 Site-specific modeled ST19A IPD IRRs for adults aged ≥65 years

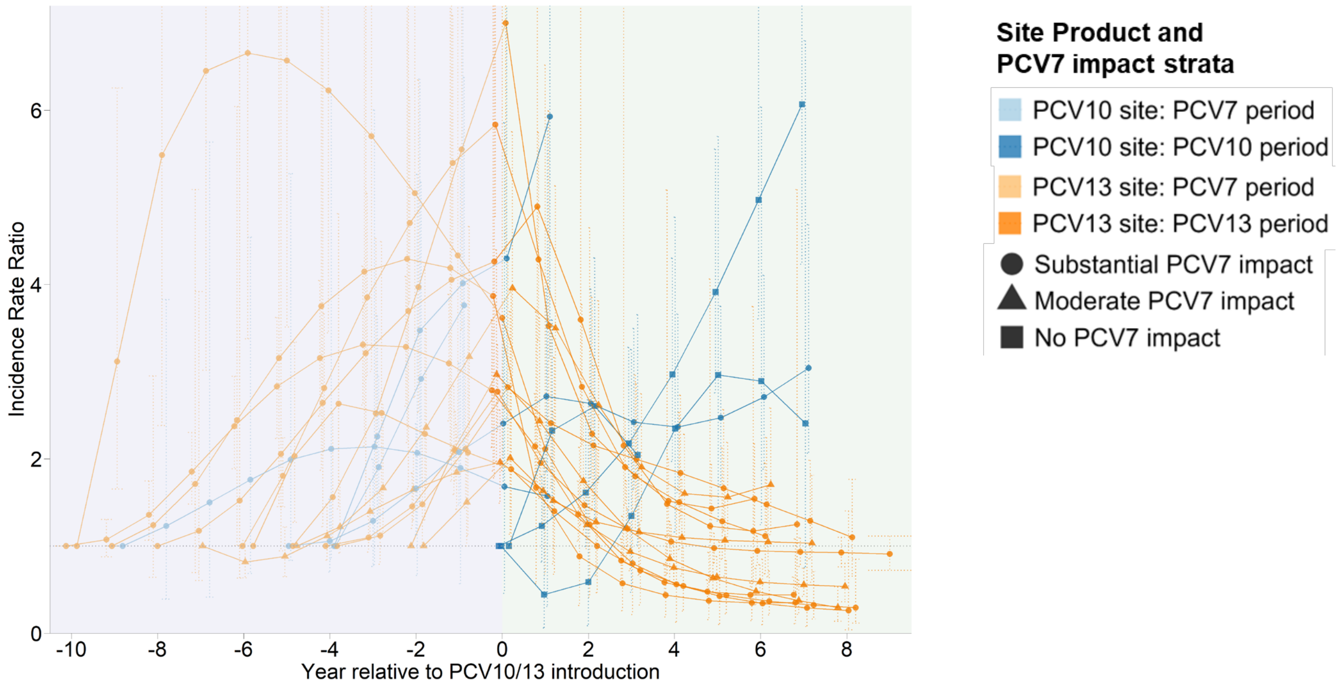

Negative years on x-axis (purple shaded area) show IRRs comparing PCV7 years of use (if any) relative to the pre-PCV IR and positive years (green shaded area) on x-axis show IRRs comparing PCV10/13 years of use relative to the pre-PCV IR

Supplementary Figure 3.7 Site-specific modeled ST3 IPD IRRs for children aged <5 years

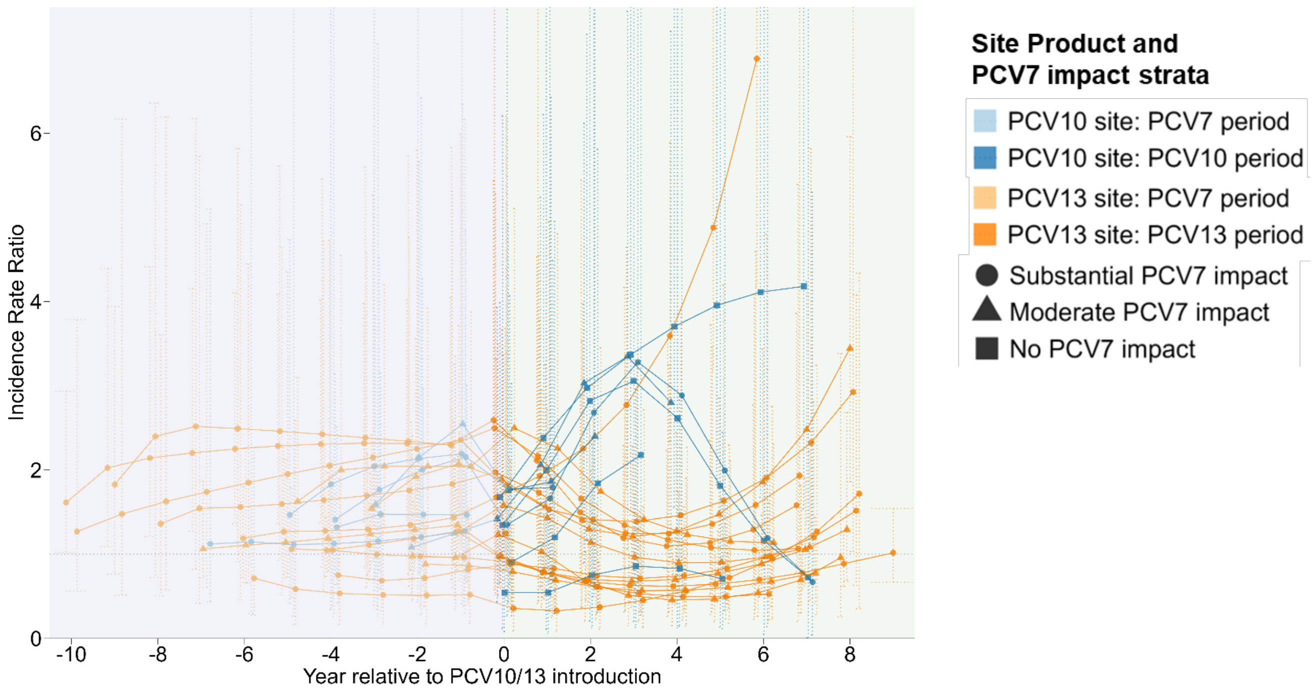

Supplementary Figure 3.8 Site-specific modeled ST3 IPD IRRs for adults aged ≥65 years

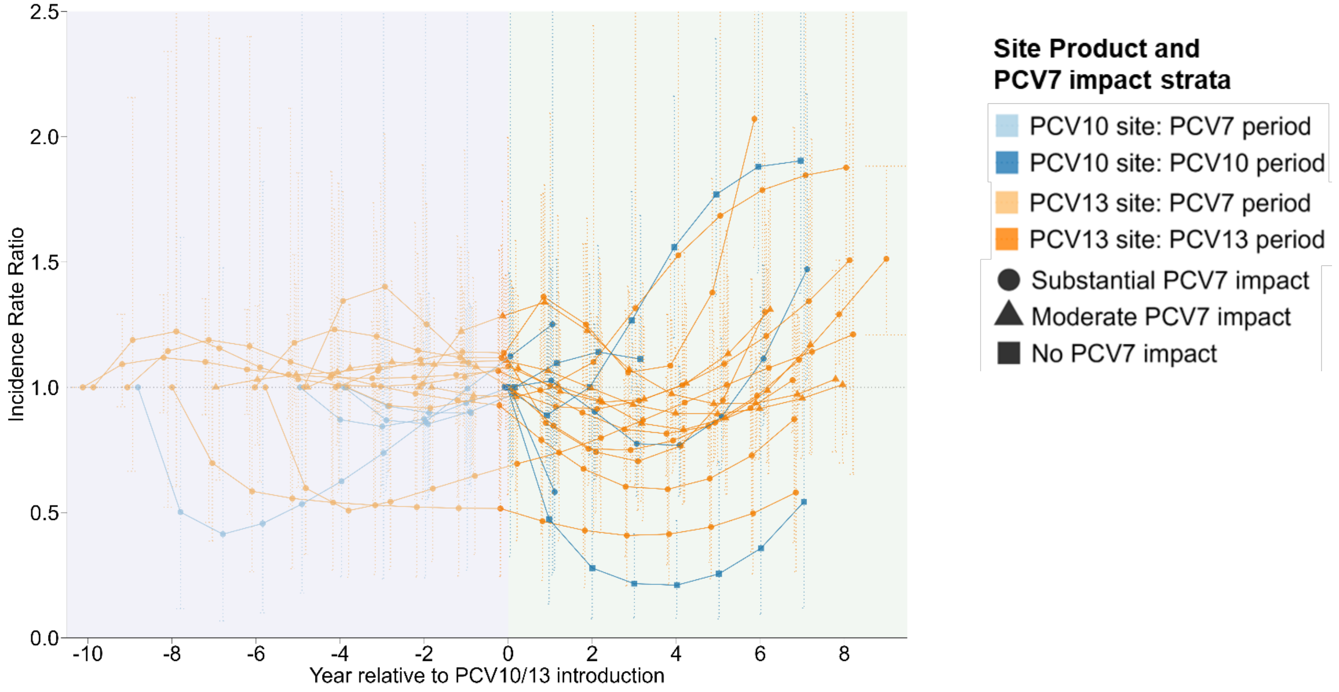

Negative years on x-axis (purple shaded area) show IRRs comparing PCV7 years of use (if any) relative to the pre-PCV IR and positive years (green shaded area) on x-axis show IRRs comparing PCV10/13 years of use relative to the pre-PCV IR

Supplementary Figure 3.9 Site-specific modeled all non-PCV13 ST IPD IRRs for children aged <5 years

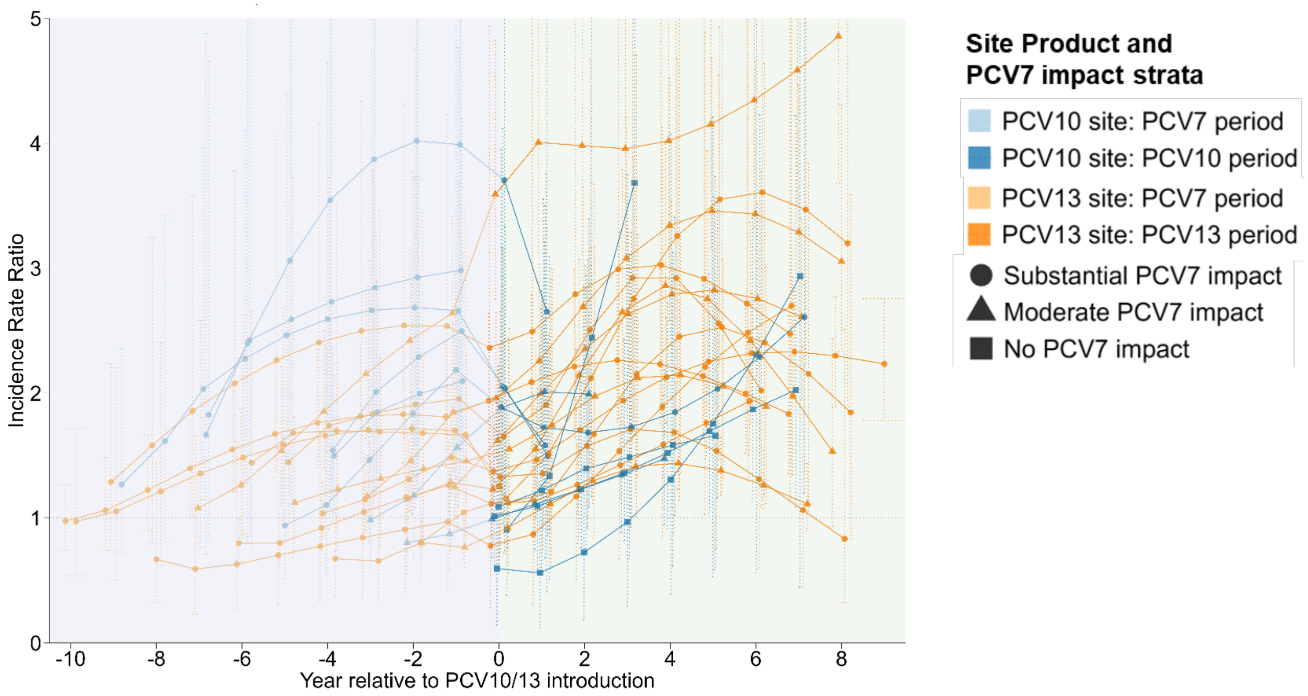

Supplementary Figure 3.10 Site-specific modeled all non-PCV13 ST IPD IRRs for adults aged ≥65 years

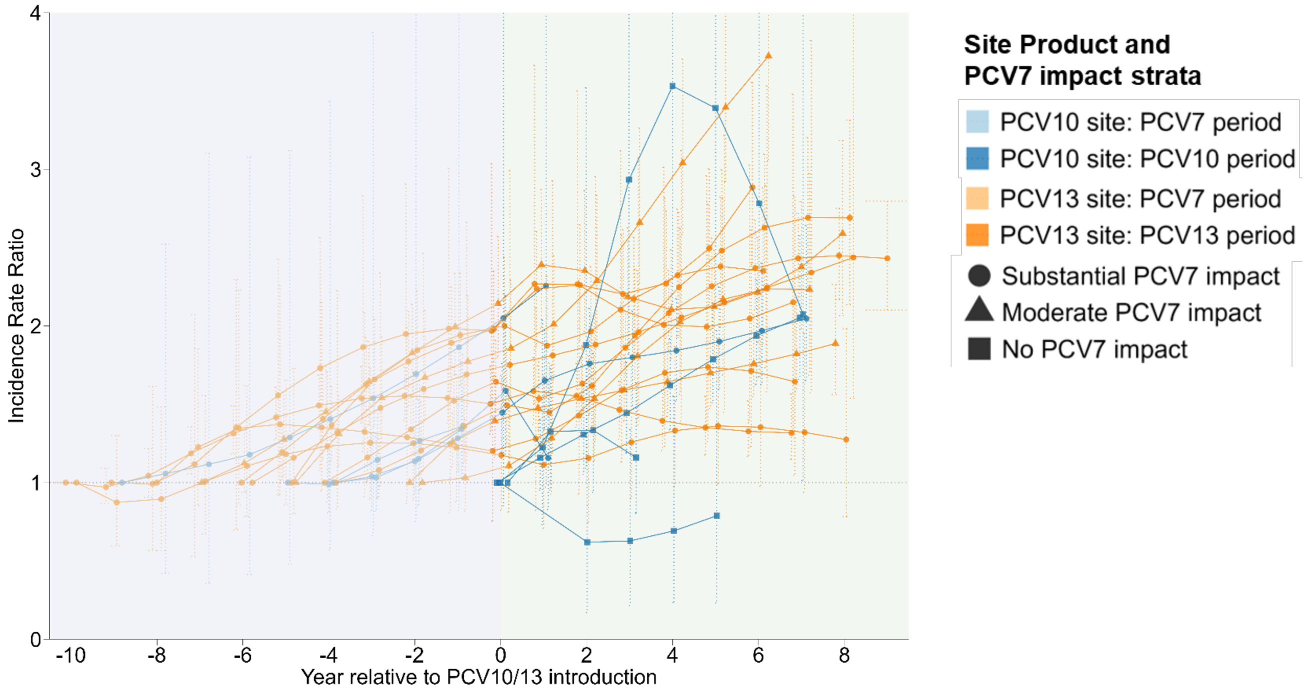

Negative years on x-axis (purple shaded area) show IRRs comparing PCV7 years of use (if any) relative to the pre-PCV IR and positive years (green shaded area) on x-axis show IRRs comparing PCV10/13 years of use relative to the pre-PCV IR

Supplementary Figure 3.11 Site-specific modeled **all ST** IPD IRRs **by infant PCV10/13 schedule** for children aged <5 years and adults aged ≥65 years

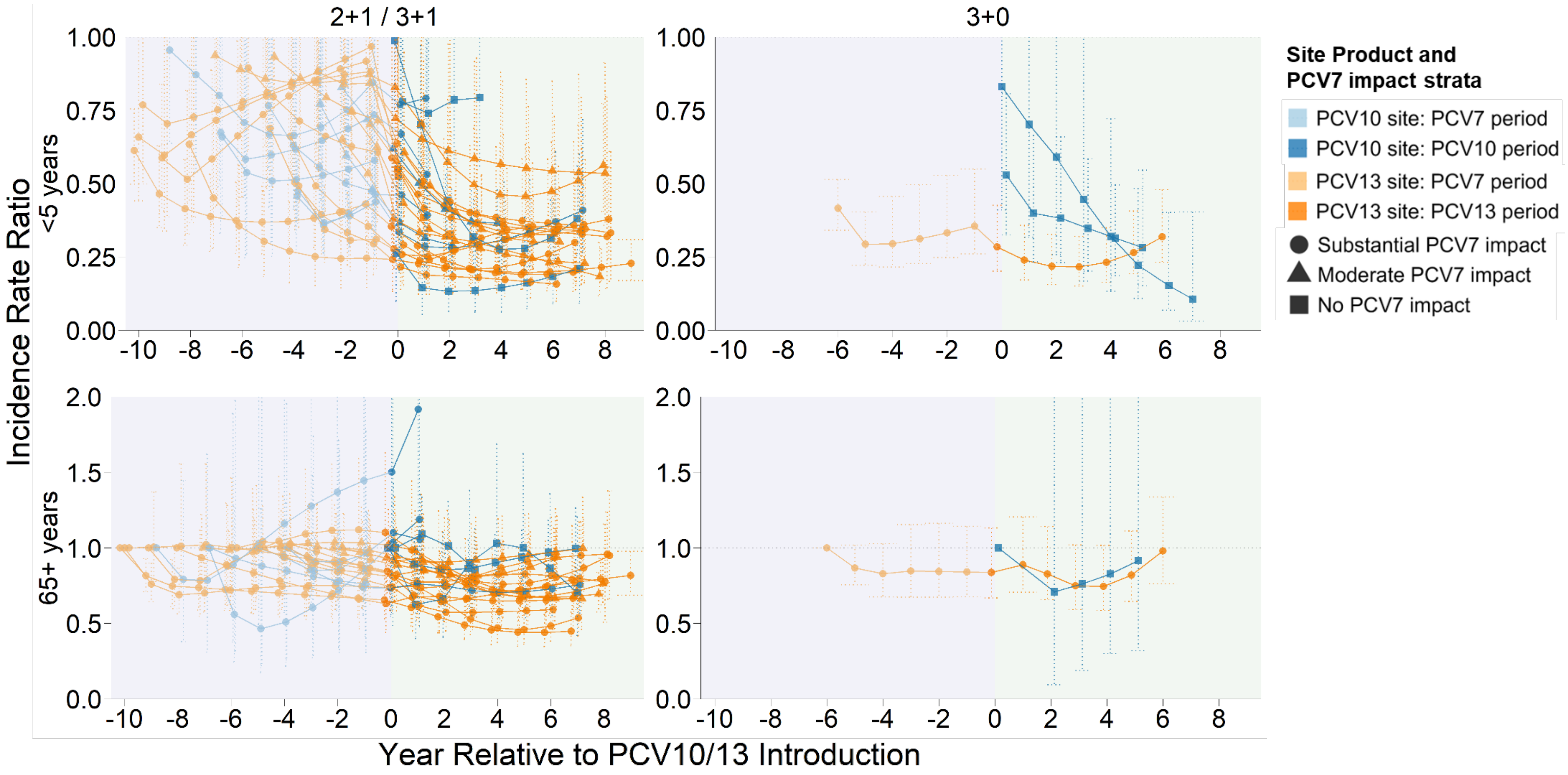

Negative years on x-axis (purple shaded area) show IRRs comparing PCV7 years of use (if any) relative to the pre-PCV IR and positive years (green shaded area) on x-axis show IRRs comparing PCV10/13 years of use relative to the pre-PCV IR

Supplementary Figure 3.12 Site-specific modeled **all PCV10 ST** IPD IRRs **by infant PCV10/13 schedule** for children aged <5 years and adults aged ≥65 years

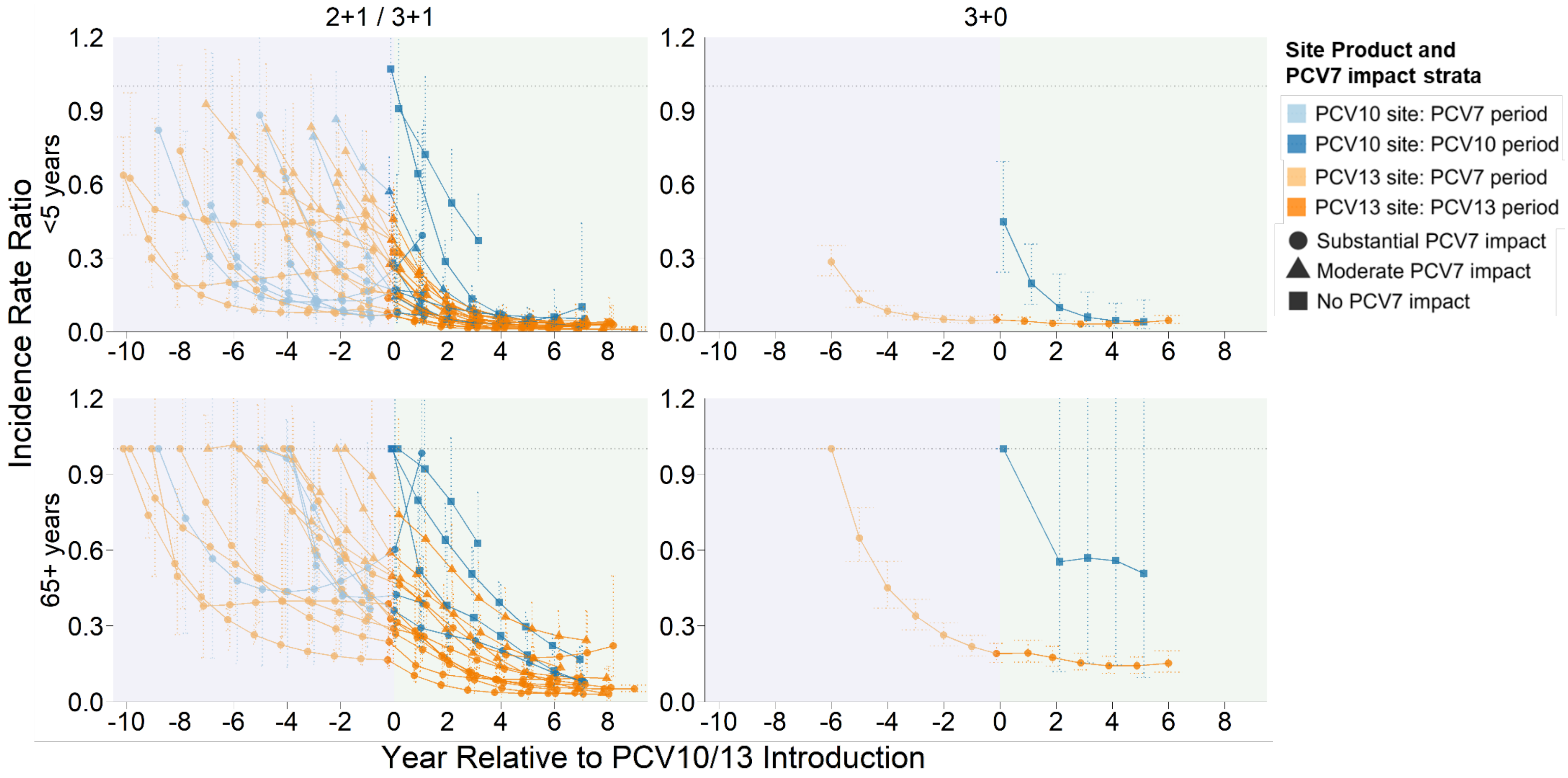

Negative years on x-axis (purple shaded area) show IRRs comparing PCV7 years of use (if any) relative to the pre-PCV IR and positive years (green shaded area) on x-axis show IRRs comparing PCV10/13 years of use relative to the pre-PCV IR

Supplementary Figure 3.13 Site-specific modeled **ST6A** IPD IRRs **by infant PCV10/13 schedule** for children aged <5 years and adults aged ≥65 years

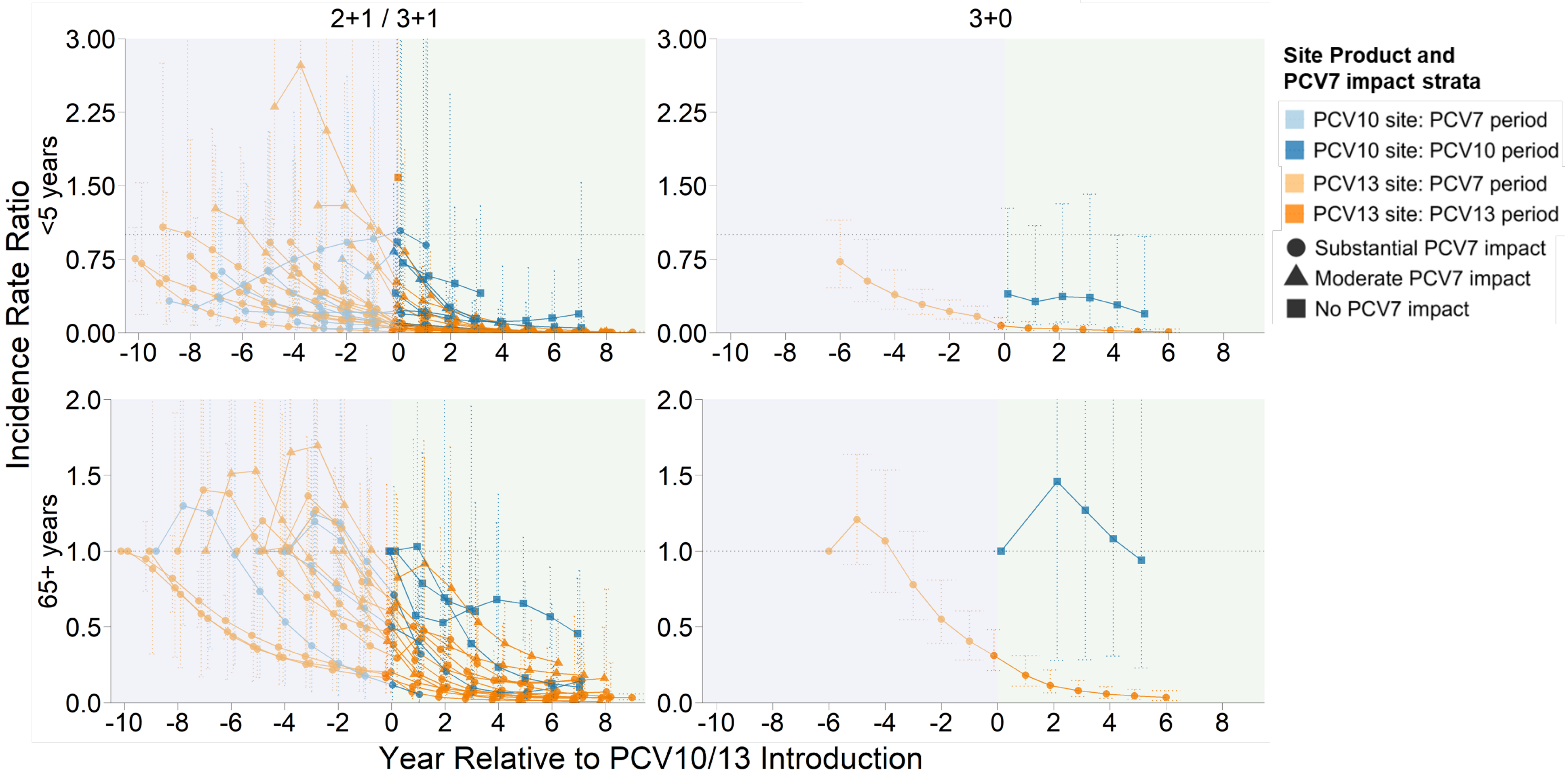

Supplementary Figure 3.14 Site-specific modeled **all non-PCV13 ST** IPD IRRs **by infant PCV10/13 schedule** for children aged <5 years and adults aged ≥65 years

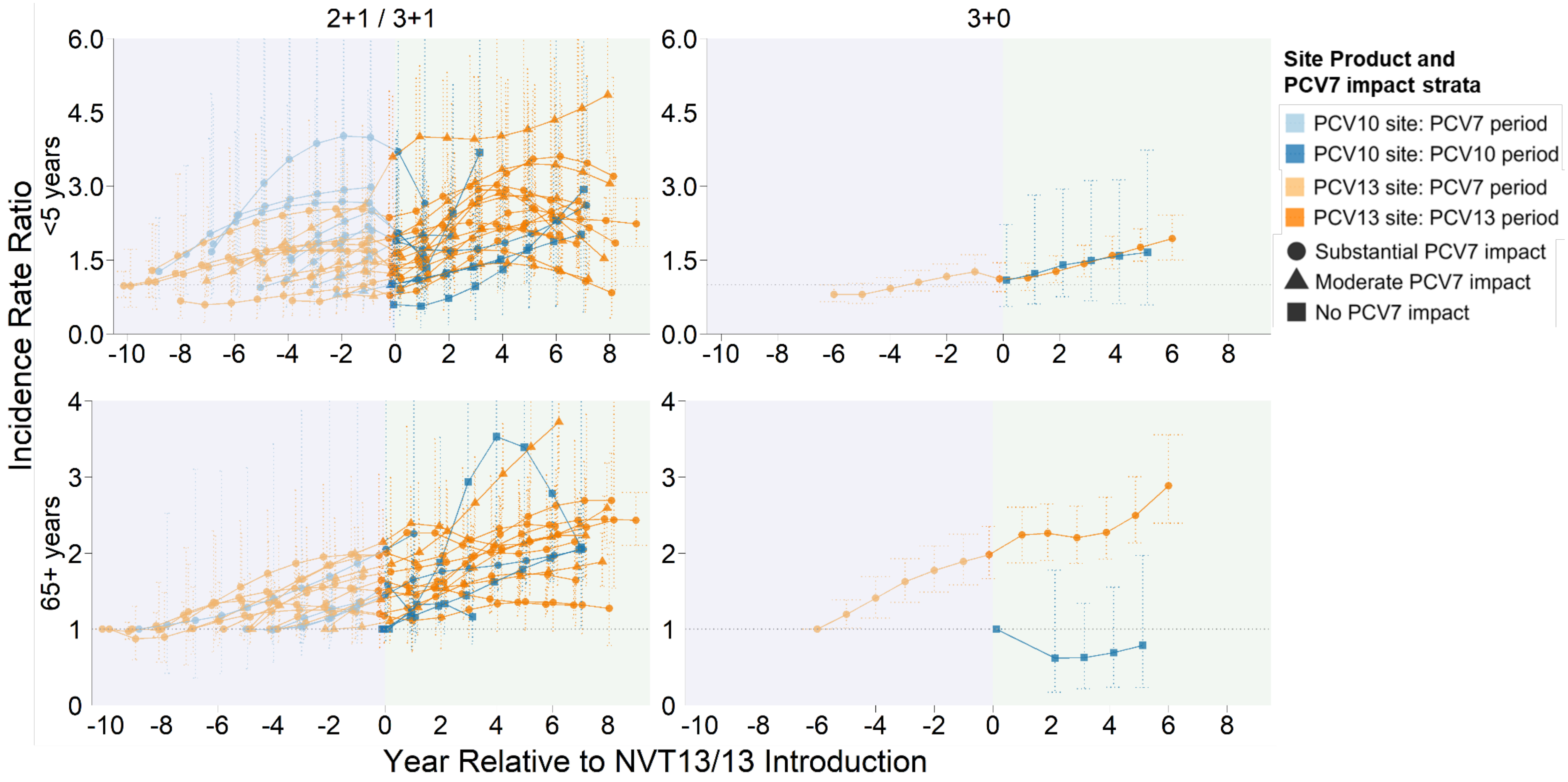

Negative years on x-axis (purple shaded area) show IRRs comparing PCV7 years of use (if any) relative to the pre-PCV IR and positive years (green shaded area) on x-axis show IRRs comparing PCV10/13 years of use relative to the pre-PCV IR

Supplementary Figure 3.15 Site-specific modeled **all ST** IPD IRRs **by PCV10/13 catch-up program** for children aged <5 years and adults aged ≥65 years

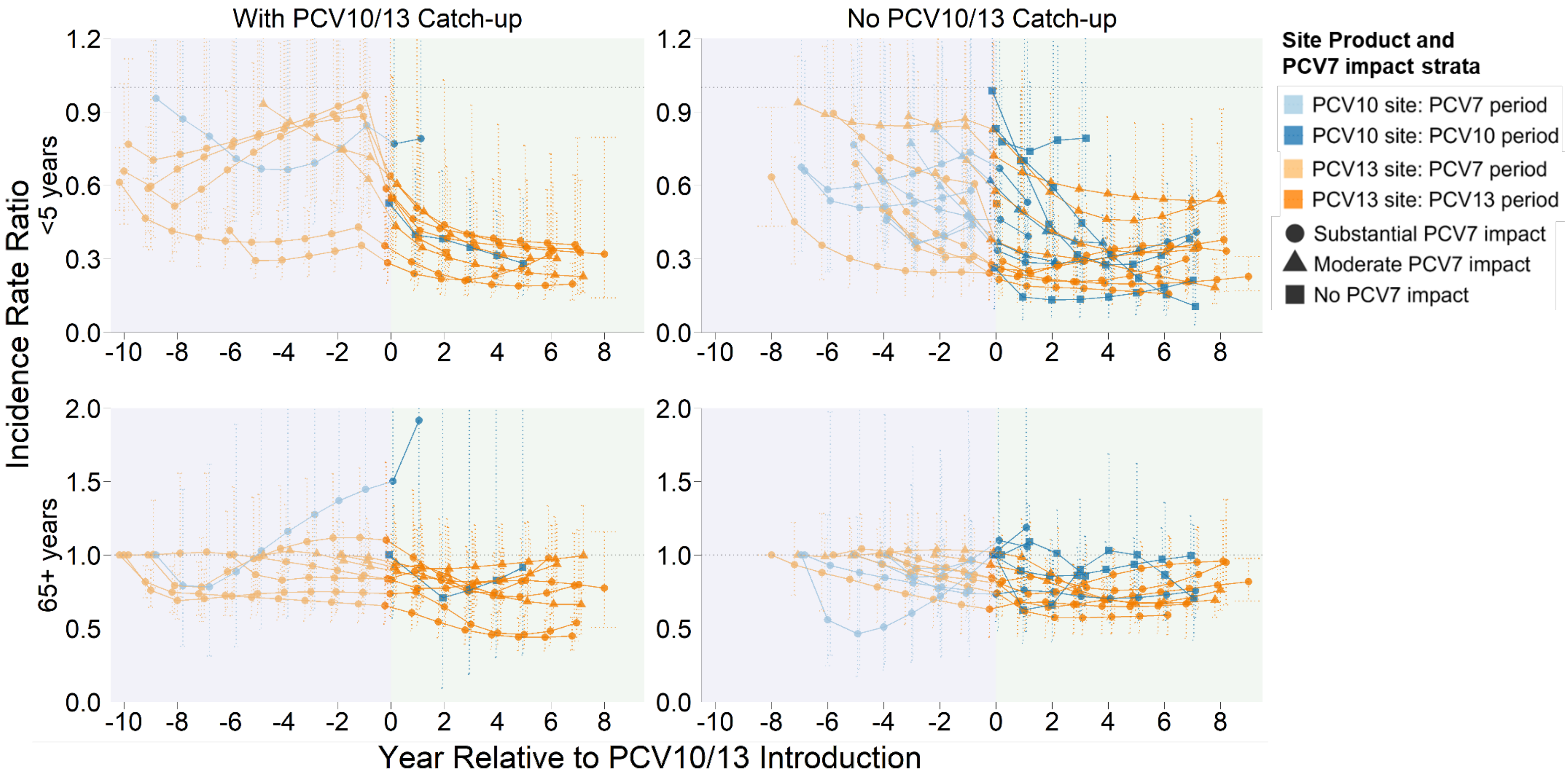

Negative years on x-axis (purple shaded area) show IRRs comparing PCV7 years of use (if any) relative to the pre-PCV IR and positive years (green shaded area) on x-axis show IRRs comparing PCV10/13 years of use relative to the pre-PCV IR

Supplementary Figure 3.16 Site-specific modeled **all ST** IPD IRRs **by region** for children <5 aged years and adults aged ≥65 years

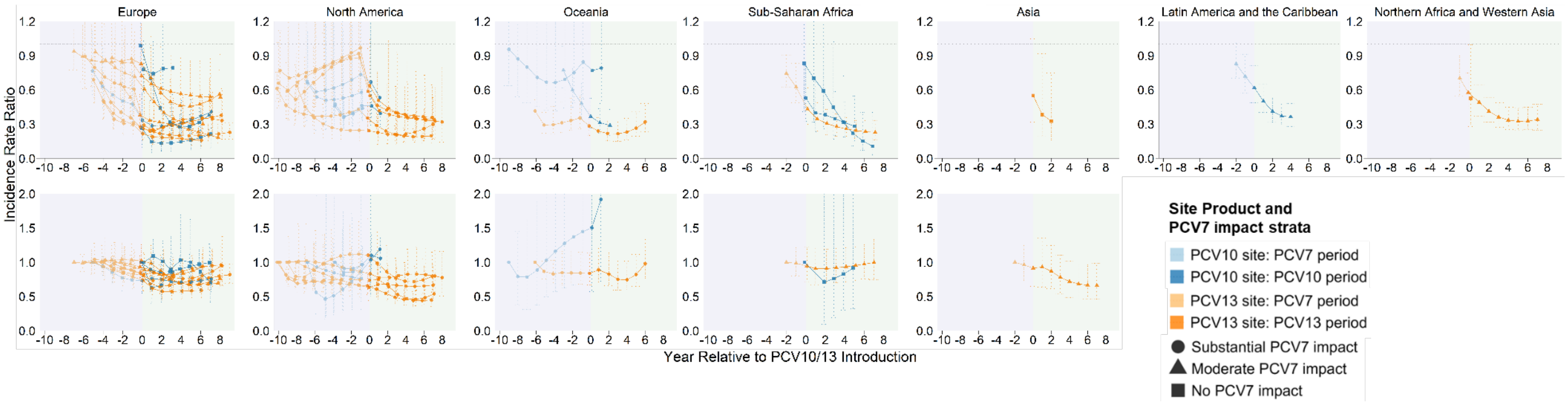

Negative years on x-axis (purple shaded area) show IRRs comparing PCV7 years of use (if any) relative to the pre-PCV IR and positive years (green shaded area) on x-axis show IRRs comparing PCV10/13 years of use relative to the pre-PCV IR

Supplementary Figure 3.17 Site-specific modeled all ST IPD IRRs for adults aged ≥65 years by adult pneumococcal vaccine recommendation. Sites with both a PCV13 and PPV23 recommendation for adults are included in both plots. Recommendations can be for either all adults or for specialized populations only.

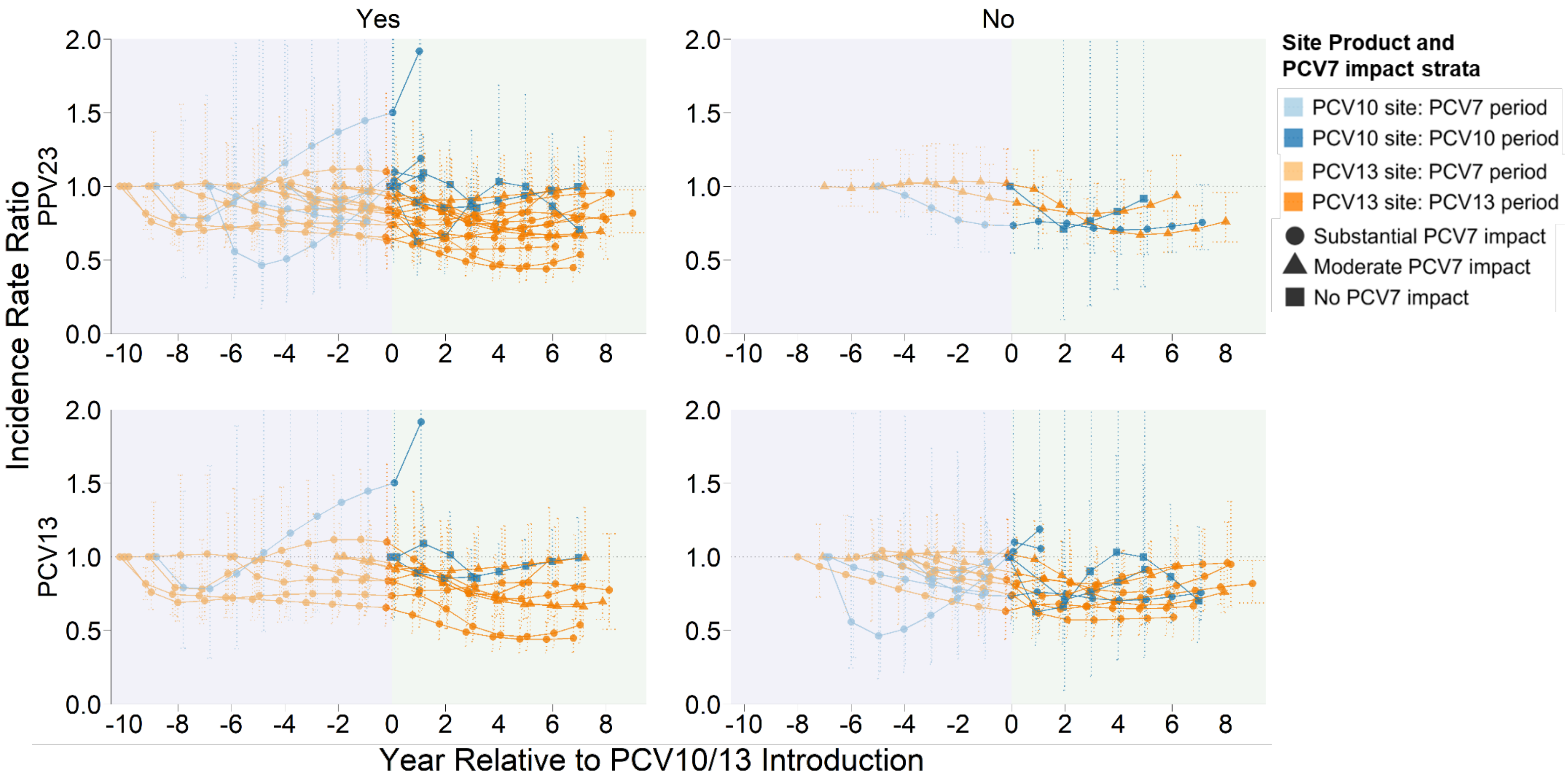

Negative years on x-axis (purple shaded area) show IRRs comparing PCV7 years of use (if any) relative to the pre-PCV IR and positive years (green shaded area) on x-axis show IRRs comparing PCV10/13 years of use relative to the pre-PCV IR.

Supplementary Figure 3.18 Site-specific modeled all ST IPD IRRs for adults ≥65 years by adult pneumococcal vaccine recommendation. Sites with both a PCV13 and PPV23 recommendation for adults are included in both plots. Recommendations can be for either all adults or for specialized populations only.

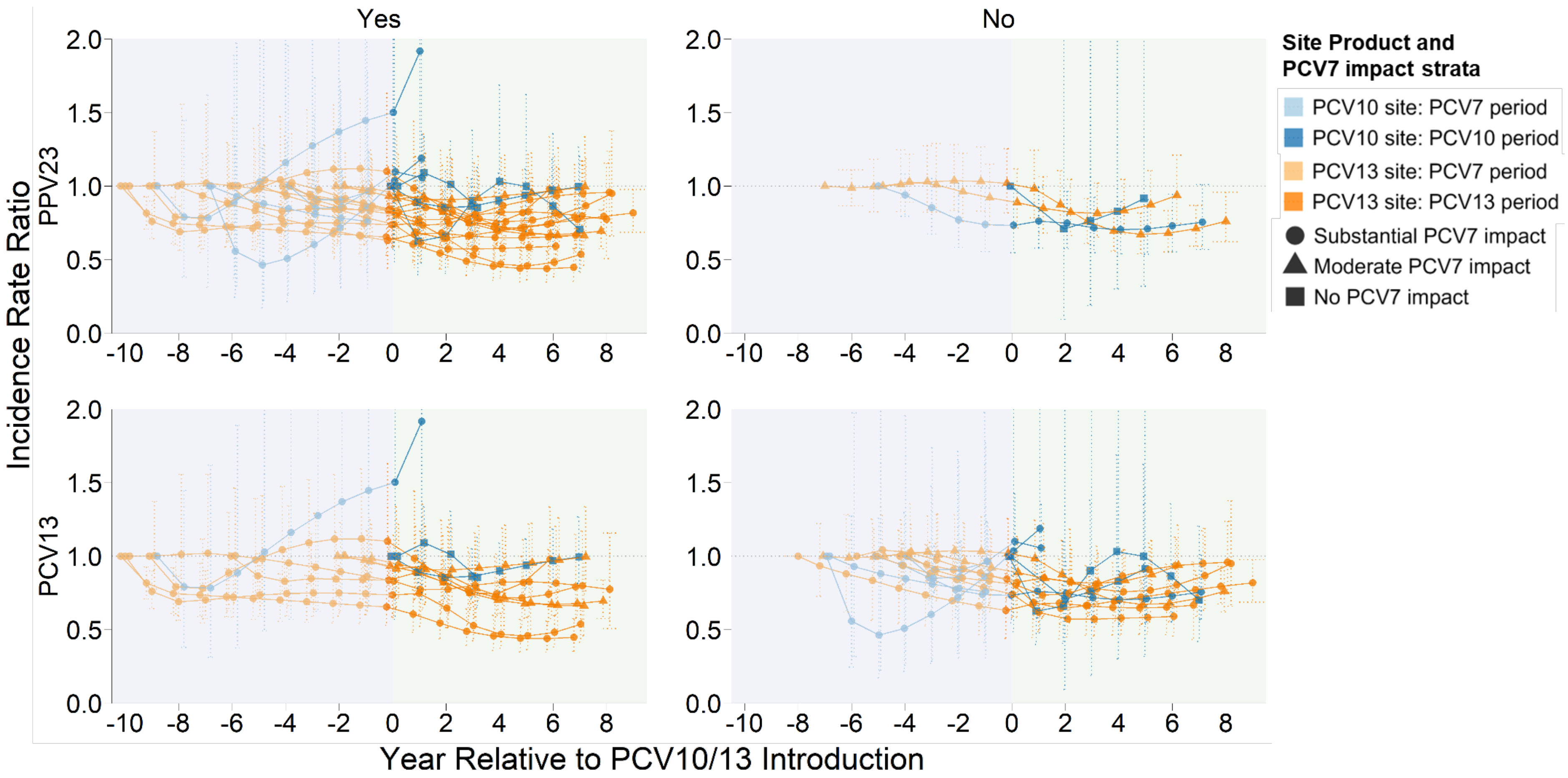

Negative years on x-axis (purple shaded area) show IRRs comparing PCV7 years of use (if any) relative to the pre-PCV IR and positive years (green shaded area) on x-axis show IRRs comparing PCV10/13 years of use relative to the pre-PCV IR.

Supplementary Figure 3.19 All ST IPD all-site weighted average IRRs comparing the annual post-PCV10/13 IR to the average pre-PCV IR for **children aged <5 years**

PCV Product and PCV7 Impact Strata	Number of Sites	Number of Cases	IRR (95%CI
PCV13			
No PCV7 impact			
Year −1	--	--	--
Year 0	2	23	0.54 (0.26, 1.13)
Year 1	1	20	0.38 (0.18, 0.81)
Year 2	1	2	0.32 (0.11, 0.96)
Moderate PCV7 impact			
Year −1	6	2428	0.66 (0.51, 0.86)
Year 0	6	1949	0.58 (0.44, 0.75)
Year 1	6	1614	0.49 (0.38, 0.64)
Year 2	6	1310	0.43 (0.33, 0.55)
Year 3	6	1136	0.38 (0.29, 0.49)
Year 4	6	1063	0.35 (0.27, 0.46)
Year 5	6	916	0.34 (0.26, 0.44)
Year 6	6	971	0.34 (0.26, 0.44)
Year 7	5	960	0.34 (0.26, 0.45)
Year 8	3	963	0.35 (0.27, 0.46)
Substantial PCV7 impact			
Year −1	12	1382	0.42 (0.35, 0.51)
Year 0	12	1475	0.38 (0.31, 0.45)
Year 1	12	1019	0.33 (0.28, 0.4)
Year 2	12	739	0.3 (0.25, 0.36)
Year 3	12	609	0.28 (0.23, 0.33)
Year 4	12	663	0.27 (0.22, 0.32)
Year 5	12	727	0.27 (0.22, 0.32)
Year 6	12	811	0.28 (0.23, 0.33)
Year 7	8	853	0.29 (0.24, 0.35)
Year 8	4	461	0.31 (0.26, 0.37)
Year 9	1	336	0.33 (0.28, 0.4)
PCV10			
No PCV7 impact			
Year −1	--	--	--
Year 0	5	223	0.59 (0.44, 0.79)
Year 1	5	173	0.49 (0.37, 0.66)
Year 2	5	128	0.39 (0.29, 0.52)
Year 3	5	73	0.32 (0.24, 0.43)
Year 4	4	87	0.28 (0.21, 0.38)
Year 5	4	39	0.26 (0.2, 0.35)
Year 6	3	30	0.26 (0.19, 0.34)
Year 7	3	37	0.26 (0.19, 0.35)
Moderate PCV7 impact			
Year −1	2	305	0.58 (0.37, 0.91)
Year 0	2	235	0.47 (0.3, 0.74)
Year 1	2	195	0.4 (0.25, 0.62)
Year 2	2	168	0.34 (0.22, 0.54)
Year 3	1	152	0.31 (0.19, 0.48)
Year 4	1	93	0.28 (0.18, 0.45)
Substantial PCV7 impact			
Year −1	4	148	0.53 (0.38, 0.73)
Year 0	4	163	0.49 (0.35, 0.67)
Year 1	4	167	0.45 (0.33, 0.62)
Year 2	1	131	0.42 (0.3, 0.58)
Year 3	1	7	0.4 (0.29, 0.55)
Year 4	1	16	0.4 (0.29, 0.55)
Year 5	1	15	0.41 (0.29, 0.56)
Year 6	1	11	0.42 (0.3, 0.59)
Year 7	1	16	0.45 (0.32, 0.63)

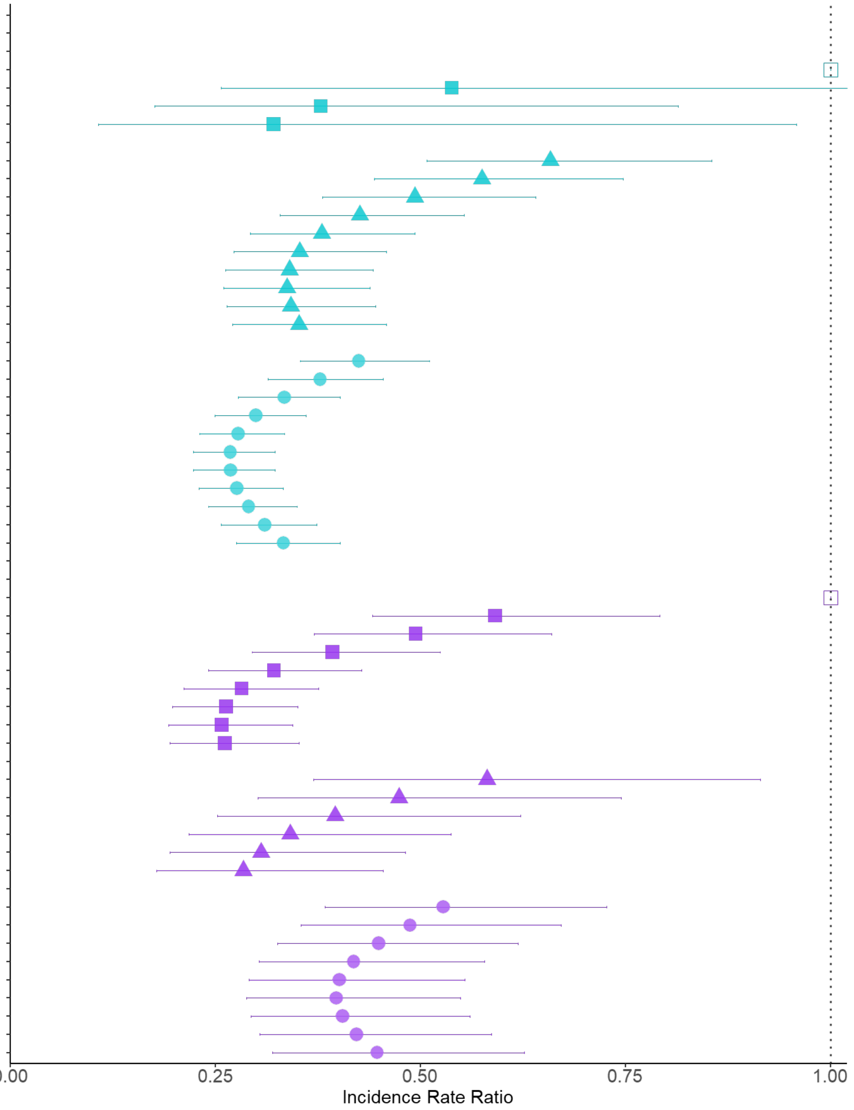

Supplementary Figure 3.20 All ST IPD all-site weighted average IRRs comparing the annual post-PCV10/13 IR to the average pre-PCV IR for **children aged 5–17 years**

PCV Product and PCV7 Impact Strata	Number of Sites	Number of Cases	IRR (95%CI)
PCV13			
Moderate PCV7 impact			
Year −1	6	865	1.02 (0.77, 1.35)
Year 0	7	824	0.92 (0.69, 1.21)
Year 1	7	686	0.79 (0.6, 1.05)
Year 2	7	478	0.68 (0.51, 0.9)
Year 3	7	431	0.59 (0.44, 0.78)
Year 4	7	341	0.52 (0.4, 0.69)
Year 5	7	338	0.48 (0.36, 0.63)
Year 6	7	312	0.44 (0.33, 0.59)
Year 7	6	318	0.42 (0.31, 0.55)
Year 8	3	116	0.4 (0.3, 0.53)
Substantial PCV7 impact			
Year −1	11	493	0.61 (0.48, 0.76)
Year 0	11	535	0.56 (0.45, 0.7)
Year 1	11	413	0.52 (0.41, 0.65)
Year 2	11	343	0.47 (0.38, 0.59)
Year 3	11	297	0.43 (0.35, 0.54)
Year 4	11	283	0.4 (0.32, 0.5)
Year 5	11	299	0.38 (0.3, 0.47)
Year 6	11	327	0.35 (0.28, 0.44)
Year 7	7	199	0.33 (0.26, 0.42)
Year 8	4	99	0.31 (0.25, 0.39)
Year 9	1	79	0.3 (0.24, 0.37)
PCV10			
No PCV7 impact			
Year 0	--	--	--
Year 1	6	49	0.8 (0.57, 1.13)
Year 2	6	42	0.7 (0.51, 0.96)
Year 3	6	36	0.61 (0.44, 0.84)
Year 4	5	25	0.54 (0.39, 0.74)
Year 5	5	24	0.48 (0.35, 0.67)
Year 6	4	16	0.44 (0.32, 0.61)
Year 7	4	21	0.41 (0.28, 0.59)
Moderate PCV7 impact			
Year −1	2	127	0.9 (0.53, 1.53)
Year 0	2	60	0.72 (0.42, 1.22)
Year 1	2	65	0.65 (0.38, 1.09)
Year 2	2	61	0.6 (0.36, 1.03)
Year 3	1	24	0.54 (0.32, 0.93)
Year 4	1	24	0.45 (0.25, 0.81)
Substantial PCV7 impact			
Year −1	4	43	0.74 (0.51, 1.07)
Year 0	4	53	0.74 (0.51, 1.08)
Year 1	4	71	0.74 (0.51, 1.07)
Year 2	1	8	0.72 (0.49, 1.05)
Year 3	1	10	0.7 (0.48, 1.02)
Year 4	1	8	0.66 (0.45, 0.97)
Year 5	1	6	0.62 (0.42, 0.91)
Year 6	1	9	0.58 (0.39, 0.85)
Year 7	1	8	0.53 (0.36, 0.8)

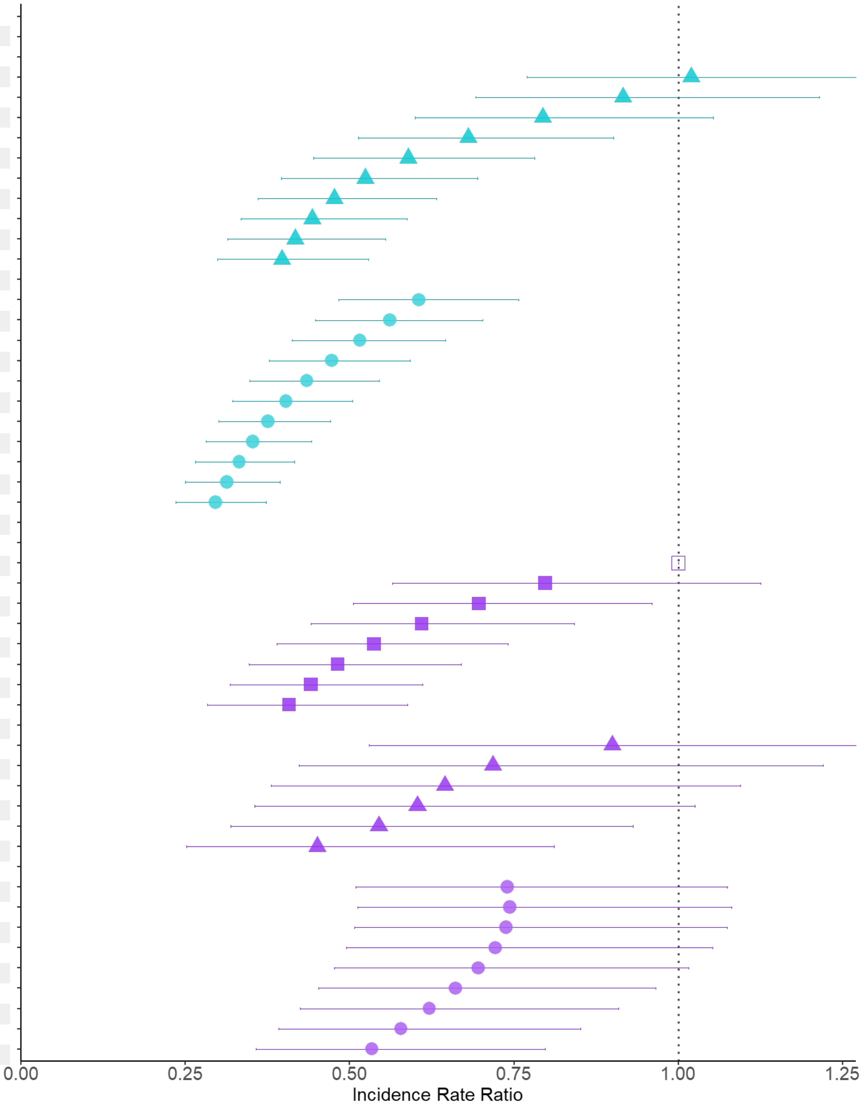

Supplementary Figure 3.21 All ST IPD all-site weighted average IRRs comparing the annual post-PCV10/13 IR to the average pre-PCV IR for **adults aged 18–49 years**

PCV Product and PCV7 Impact Strata	Number of Sites	Number of Cases	IRR (95%CI)
PCV13			
Moderate PCV7 impact			
Year −1	5	3591	1.09 (0.92, 1.29)
Year 0	5	3819	1.04 (0.88, 1.23)
Year 1	5	2997	0.96 (0.81, 1.14)
Year 2	5	3006	0.86 (0.72, 1.01)
Year 3	5	2687	0.76 (0.64, 0.9)
Year 4	5	2524	0.68 (0.57, 0.8)
Year 5	5	2397	0.63 (0.53, 0.75)
Year 6	5	2432	0.6 (0.51, 0.71)
Year 7	4	2209	0.58 (0.49, 0.69)
Year 8	2	1049	0.57 (0.48, 0.68)
Substantial PCV7 impact			
Year −1	12	3639	0.82 (0.7, 0.96)
Year 0	12	3177	0.74 (0.64, 0.87)
Year 1	12	2582	0.67 (0.58, 0.78)
Year 2	12	2187	0.61 (0.53, 0.72)
Year 3	12	2101	0.58 (0.5, 0.68)
Year 4	12	1849	0.56 (0.48, 0.66)
Year 5	12	2149	0.56 (0.48, 0.65)
Year 6	12	2479	0.57 (0.49, 0.66)
Year 7	8	1877	0.59 (0.5, 0.69)
Year 8	4	1186	0.61 (0.52, 0.72)
Year 9	1	915	0.64 (0.55, 0.75)
PCV10			
No PCV7 impact			
Year 0	--	--	--
Year 1	6	235	0.74 (0.52, 1.05)
Year 2	6	177	0.56 (0.4, 0.8)
Year 3	6	200	0.45 (0.32, 0.63)
Year 4	5	145	0.39 (0.27, 0.54)
Year 5	5	157	0.37 (0.26, 0.52)
Year 6	4	153	0.38 (0.27, 0.54)
Year 7	4	150	0.42 (0.29, 0.6)
Substantial PCV7 impact			
Year −1	4	291	0.77 (0.59, 1)
Year 0	4	310	0.78 (0.6, 1.02)
Year 1	4	342	0.79 (0.6, 1.03)
Year 2	1	111	0.78 (0.6, 1.03)
Year 3	1	67	0.75 (0.57, 0.98)
Year 4	1	97	0.7 (0.53, 0.91)
Year 5	1	67	0.63 (0.48, 0.83)
Year 6	1	60	0.56 (0.42, 0.73)
Year 7	1	69	0.48 (0.37, 0.64)

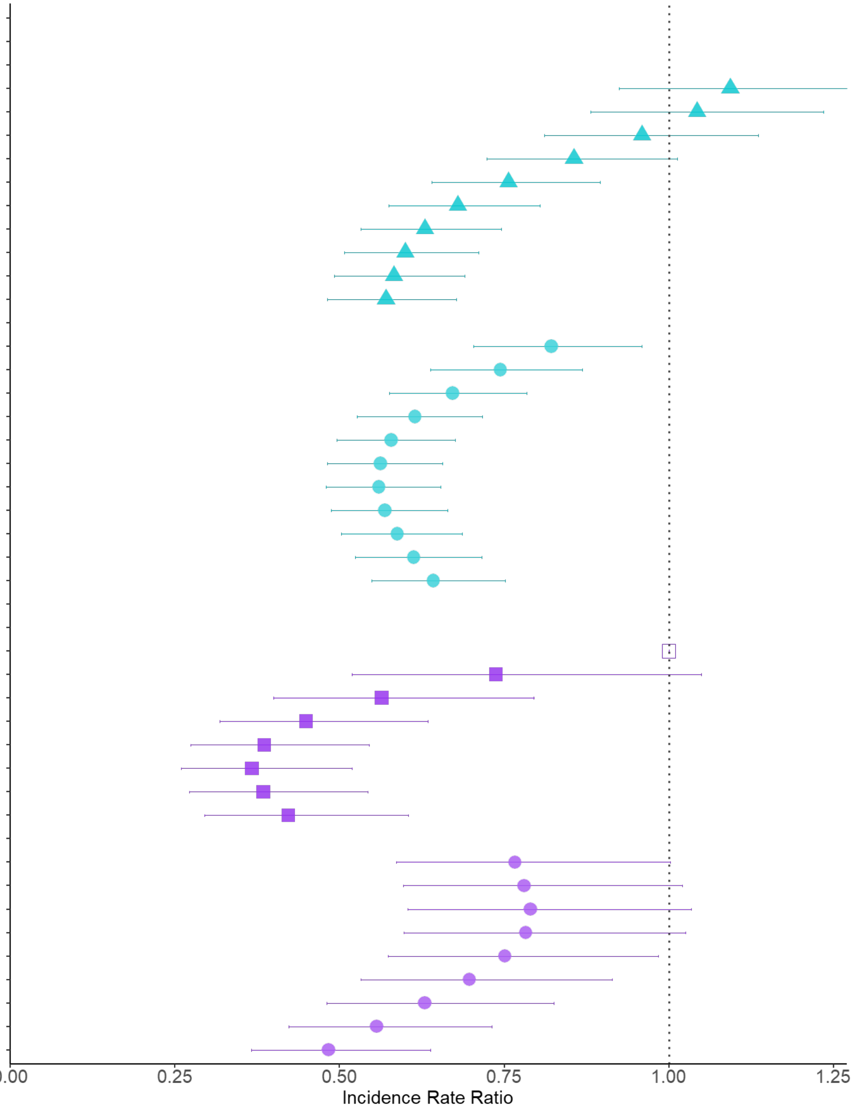

Supplementary Figure 3.22 All ST IPD all-site weighted average IRRs comparing the annual post-PCV10/13 IR to the average pre-PCV IR for **adults aged 50–64 years**

PCV Product and PCV7 Impact Strata	Number of Sites	Number of Cases	IRR (95%CI)
PCV13			
Moderate PCV7 impact			
Year −1	5	1878	1.07 (1.01, 1.14)
Year 0	5	2101	1.05 (0.99, 1.12)
Year 1	5	1944	1 (0.94, 1.06)
Year 2	5	2293	0.93 (0.87, 0.98)
Year 3	5	2143	0.86 (0.81, 0.91)
Year 4	5	2038	0.8 (0.76, 0.85)
Year 5	5	2139	0.78 (0.74, 0.83)
Year 6	5	2342	0.78 (0.74, 0.83)
Year 7	4	2194	0.79 (0.75, 0.84)
Year 8	2	1831	0.81 (0.77, 0.87)
Substantial PCV7 impact			
Year −1	12	3321	0.95 (0.85, 1.05)
Year 0	12	3231	0.9 (0.81, 1)
Year 1	12	2797	0.85 (0.77, 0.95)
Year 2	12	2548	0.82 (0.74, 0.91)
Year 3	12	2630	0.8 (0.72, 0.89)
Year 4	12	2478	0.8 (0.73, 0.89)
Year 5	12	2841	0.82 (0.74, 0.91)
Year 6	12	3321	0.85 (0.77, 0.94)
Year 7	8	2631	0.89 (0.8, 0.99)
Year 8	4	1379	0.94 (0.85, 1.04)
Year 9	1	1105	1 (0.9, 1.11)
PCV10			
No PCV7 impact			
Year 0	--	--	--
Year 1	4	286	0.83 (0.62, 1.12)
Year 2	4	301	0.73 (0.54, 0.99)
Year 3	4	270	0.66 (0.49, 0.9)
Year 4	2	166	0.63 (0.47, 0.86)
Year 5	2	207	0.65 (0.48, 0.88)
Year 6	2	209	0.7 (0.52, 0.95)
Year 7	2	205	0.78 (0.58, 1.06)
Substantial PCV7 impact			
Year −1	4	321	0.95 (0.8, 1.14)
Year 0	4	352	0.97 (0.81, 1.16)
Year 1	4	463	0.98 (0.82, 1.17)
Year 2	1	149	0.98 (0.82, 1.18)
Year 3	1	142	0.97 (0.81, 1.17)
Year 4	1	145	0.96 (0.8, 1.14)
Year 5	1	154	0.93 (0.78, 1.12)
Year 6	1	162	0.9 (0.75, 1.08)
Year 7	1	167	0.87 (0.72, 1.05)

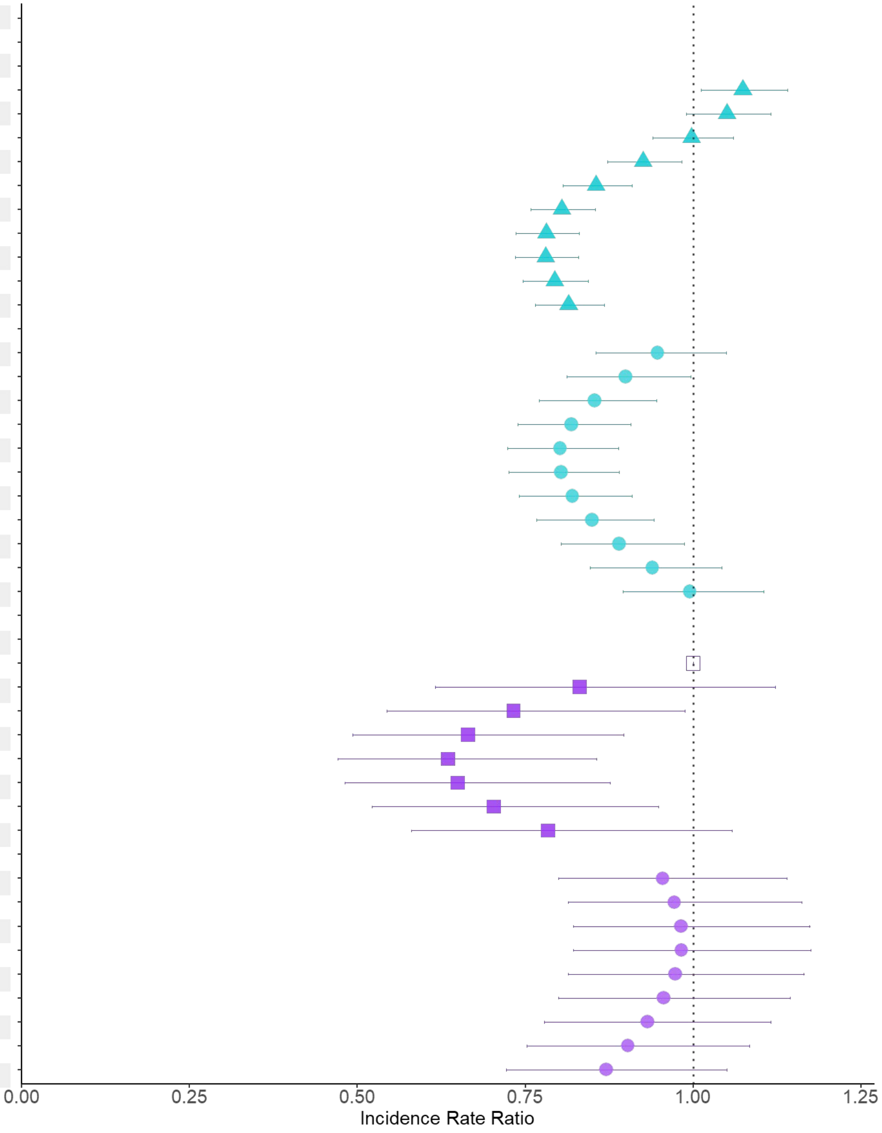

Supplementary Figure 3.23 All ST IPD all-site weighted average IRRs comparing the annual post-PCV10/13 IR to the average pre-PCV IR for **adults aged ≥65 years**

PCV Product and PCV7 Impact Strata	Number of Sites	Number of Cases	IRR (95%CI)
PCV13			
Moderate PCV7 impact			
Year −1	5	3769	0.98 (0.92, 1.05)
Year 0	5	3927	0.95 (0.89, 1.02)
Year 1	5	3844	0.91 (0.85, 0.97)
Year 2	5	4866	0.86 (0.8, 0.92)
Year 3	5	4567	0.81 (0.76, 0.87)
Year 4	5	4291	0.77 (0.72, 0.83)
Year 5	5	4816	0.75 (0.71, 0.81)
Year 6	5	5241	0.75 (0.7, 0.8)
Year 7	4	5004	0.75 (0.7, 0.8)
Year 8	2	4506	0.75 (0.7, 0.81)
Substantial PCV7 impact			
Year −1	12	5439	0.82 (0.74, 0.91)
Year 0	12	5237	0.78 (0.7, 0.87)
Year 1	12	4624	0.74 (0.67, 0.82)
Year 2	12	4362	0.72 (0.64, 0.79)
Year 3	12	4402	0.7 (0.63, 0.78)
Year 4	12	4121	0.7 (0.63, 0.77)
Year 5	12	4914	0.7 (0.63, 0.78)
Year 6	12	5797	0.71 (0.64, 0.79)
Year 7	8	4526	0.73 (0.66, 0.82)
Year 8	4	2983	0.76 (0.68, 0.84)
Year 9	1	2431	0.79 (0.71, 0.87)
PCV10			
No PCV7 impact			
Year 0	--	--	--
Year 1	4	449	0.85 (0.74, 0.97)
Year 2	4	543	0.82 (0.72, 0.94)
Year 3	4	478	0.86 (0.75, 0.98)
Year 4	3	376	0.94 (0.83, 1.08)
Year 5	3	439	0.98 (0.86, 1.12)
Year 6	2	431	0.96 (0.84, 1.09)
Year 7	2	453	0.9 (0.79, 1.04)
Substantial PCV7 impact			
Year −1	4	638	0.97 (0.81, 1.17)
Year 0	4	639	0.97 (0.81, 1.16)
Year 1	4	769	0.95 (0.79, 1.14)
Year 2	1	377	0.92 (0.77, 1.11)
Year 3	1	312	0.9 (0.75, 1.08)
Year 4	1	402	0.88 (0.74, 1.06)
Year 5	1	393	0.87 (0.72, 1.04)
Year 6	1	369	0.86 (0.71, 1.03)
Year 7	1	428	0.85 (0.7, 1.03)

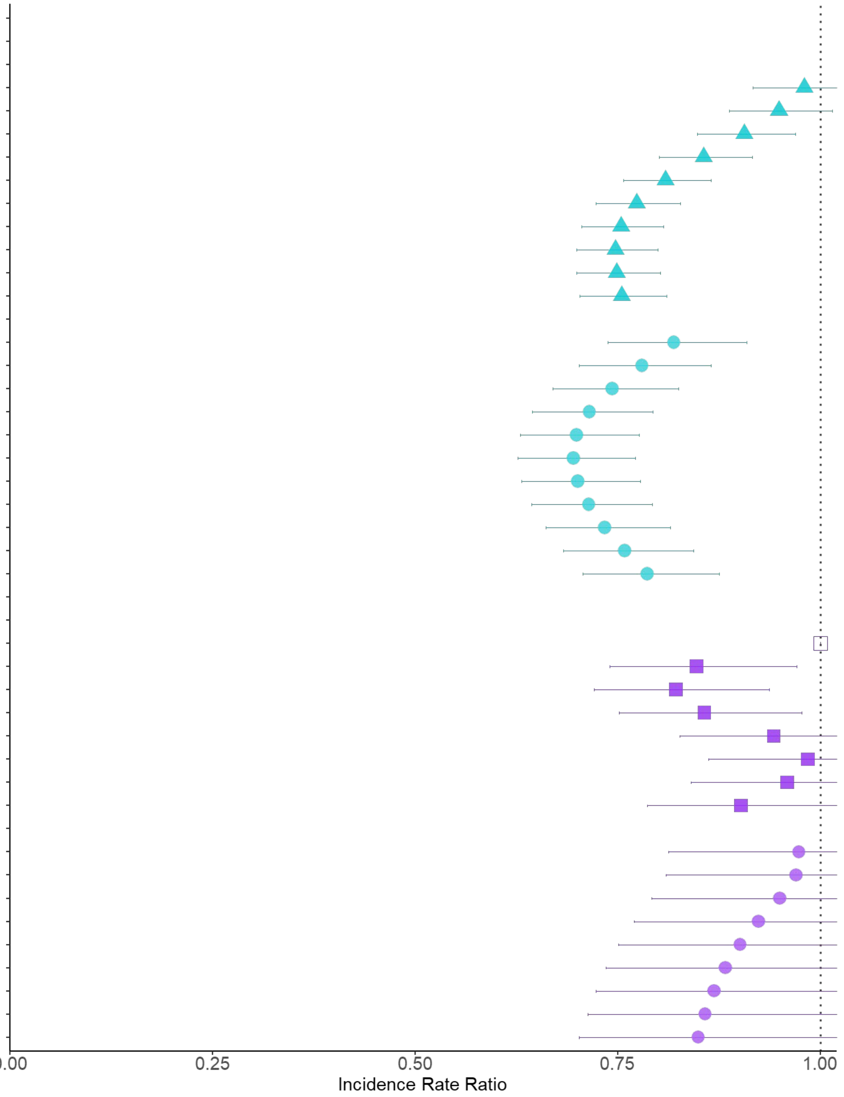

Supplementary Figure 3.24 PCV7 ST IPD all-site weighted average IRRs comparing the annual post-PCV10/13 IR to the average pre-PCV IR for children aged <5 years

PCV Product and PCV7 Impact Strata	Number of Sites	Number of Cases	IRR (95%CI)
PCV13			
Moderate PCV7 impact			
Year −1	6	790	0.17 (0.1, 0.28)
Year 0	6	443	0.1 (0.06, 0.17)
Year 1	6	207	0.07 (0.04, 0.11)
Year 2	6	111	0.04 (0.03, 0.07)
Year 3	6	83	0.03 (0.02, 0.06)
Year 4	6	63	0.03 (0.02, 0.05)
Year 5	6	44	0.03 (0.02, 0.04)
Year 6	6	55	0.03 (0.02, 0.04)
Year 7	5	42	0.03 (0.02, 0.05)
Year 8	3	47	0.03 (0.02, 0.05)
Substantial PCV7 impact			
Year −1	11	107	0.03 (0.02, 0.04)
Year 0	11	52	0.02 (0.01, 0.03)
Year 1	11	35	0.02 (0.01, 0.02)
Year 2	11	26	0.01 (0.01, 0.02)
Year 3	11	21	0.01 (0.01, 0.02)
Year 4	11	32	0.01 (0.01, 0.02)
Year 5	11	36	0.01 (0.01, 0.02)
Year 6	11	38	0.01 (0.01, 0.02)
Year 7	7	41	0.01 (0.01, 0.02)
Year 8	4	14	0.01 (0.01, 0.02)
Year 9	1	11	0.01 (0.01, 0.02)
PCV10			
No PCV7 impact			
Year −1	--	--	--
Year 0	4	137	0.61 (0.29, 1.32)
Year 1	4	121	0.33 (0.15, 0.69)
Year 2	4	73	0.16 (0.08, 0.35)
Year 3	4	33	0.08 (0.04, 0.17)
Year 4	3	20	0.04 (0.02, 0.09)
Year 5	3	3	0.03 (0.01, 0.07)
Year 6	2	4	0.03 (0.01, 0.06)
Year 7	2	4	0.03 (0.01, 0.06)
Moderate PCV7 impact			
Year −1	2	158	0.37 (0.15, 0.89)
Year 0	2	115	0.24 (0.1, 0.57)
Year 1	2	96	0.15 (0.06, 0.37)
Year 2	2	45	0.09 (0.04, 0.22)
Year 3	1	34	0.04 (0.02, 0.1)
Year 4	1	8	0.01 (0, 0.04)
Substantial PCV7 impact			
Year −1	4	3	0.03 (0.02, 0.06)
Year 0	4	0	0.02 (0.01, 0.04)
Year 1	4	4	0.02 (0.01, 0.03)
Year 2	1	3	0.01 (0.01, 0.02)
Year 3	1	1	0.01 (0.01, 0.02)
Year 4	1	1	0.01 (0.01, 0.02)
Year 5	1	1	0.01 (0.01, 0.02)
Year 6	1	2	0.01 (0.01, 0.03)
Year 7	1	1	0.02 (0.01, 0.05)

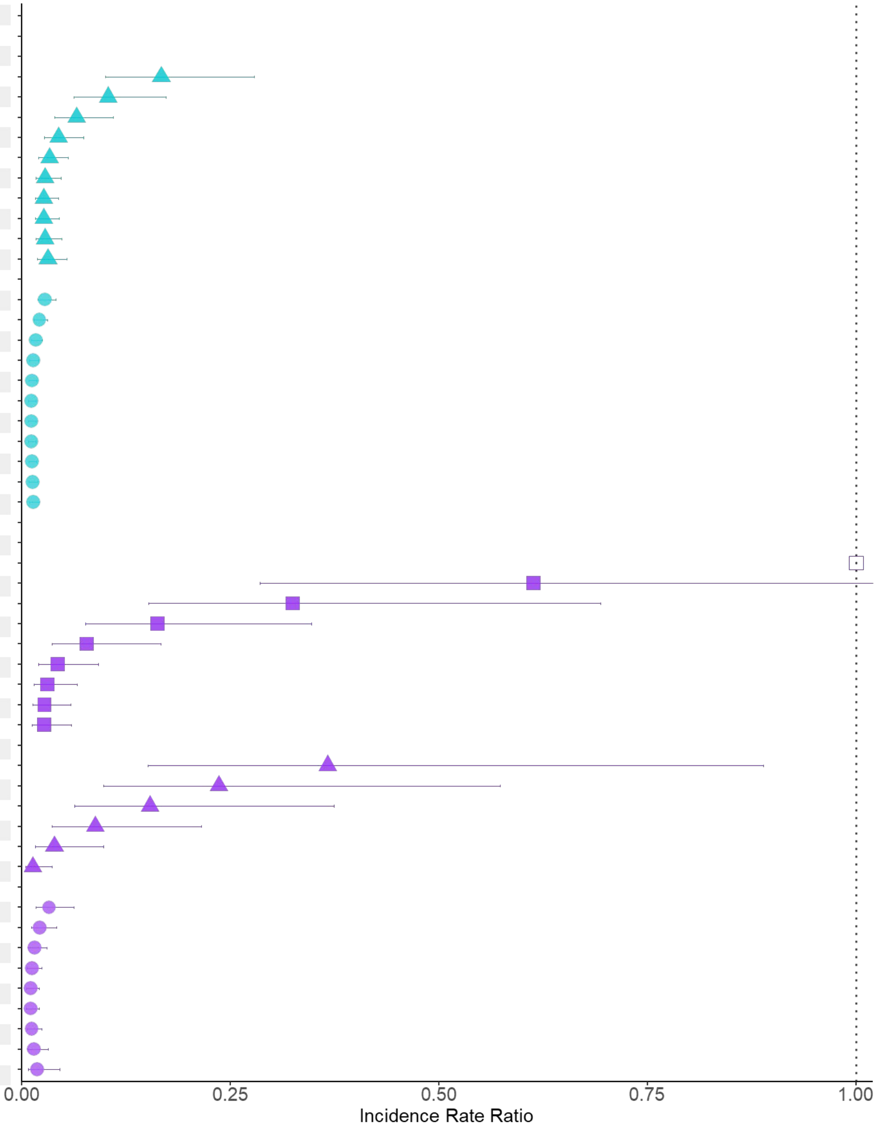

Supplementary Figure 3.25 PCV7 ST IPD all-site weighted average IRRs comparing the annual post-PCV10/13 IR to the average pre-PCV IR for children aged 5–17 years

PCV Product and PCV7 Impact Strata	Number of Sites	Number of Cases	IRR (95%CI)
PCV13			
Moderate PCV7 impact			
Year −1	5	205	0.57 (0.4, 0.82)
Year 0	6	158	0.4 (0.28, 0.58)
Year 1	6	79	0.28 (0.19, 0.4)
Year 2	6	38	0.2 (0.14, 0.28)
Year 3	6	42	0.15 (0.1, 0.21)
Year 4	6	26	0.13 (0.09, 0.18)
Year 5	6	33	0.12 (0.08, 0.17)
Year 6	6	25	0.12 (0.08, 0.17)
Year 7	5	24	0.13 (0.09, 0.18)
Year 8	3	7	0.14 (0.1, 0.2)
Substantial PCV7 impact			
Year −1	10	60	0.13 (0.1, 0.17)
Year 0	10	51	0.1 (0.07, 0.13)
Year 1	10	13	0.08 (0.06, 0.1)
Year 2	10	22	0.06 (0.05, 0.08)
Year 3	10	21	0.05 (0.04, 0.07)
Year 4	10	13	0.05 (0.04, 0.07)
Year 5	10	26	0.05 (0.04, 0.06)
Year 6	10	23	0.05 (0.04, 0.07)
Year 7	7	16	0.05 (0.04, 0.07)
Year 8	4	4	0.06 (0.04, 0.08)
Year 9	1	5	0.07 (0.05, 0.09)
PCV10			
No PCV7 impact			
Year 0	––	––	––
Year 1	5	11	0.41 (0.24, 0.67)
Year 2	5	11	0.38 (0.23, 0.63)
Year 3	5	12	0.33 (0.2, 0.55)
Year 4	4	5	0.25 (0.15, 0.42)
Year 5	4	4	0.16 (0.1, 0.27)
Year 6	3	0	0.09 (0.06, 0.15)
Year 7	3	2	0.05 (0.03, 0.08)
Moderate PCV7 impact			
Year −1	2	31	0.85 (0.45, 1.6)
Year 0	2	11	0.7 (0.37, 1.32)
Year 1	2	23	0.65 (0.35, 1.21)
Year 2	2	15	0.58 (0.31, 1.08)
Year 3	1	7	0.42 (0.22, 0.82)
Year 4	1	3	0.23 (0.1, 0.51)
Substantial PCV7 impact			
Year −1	3	4	0.41 (0.24, 0.68)
Year 0	3	4	0.31 (0.18, 0.51)
Year 1	3	2	0.22 (0.13, 0.37)
Year 2	1	0	0.16 (0.09, 0.26)
Year 3	1	1	0.11 (0.07, 0.19)
Year 4	1	1	0.08 (0.05, 0.14)
Year 5	1	0	0.07 (0.04, 0.11)
Year 6	1	0	0.05 (0.03, 0.1)
Year 7	1	1	0.05 (0.02, 0.08)

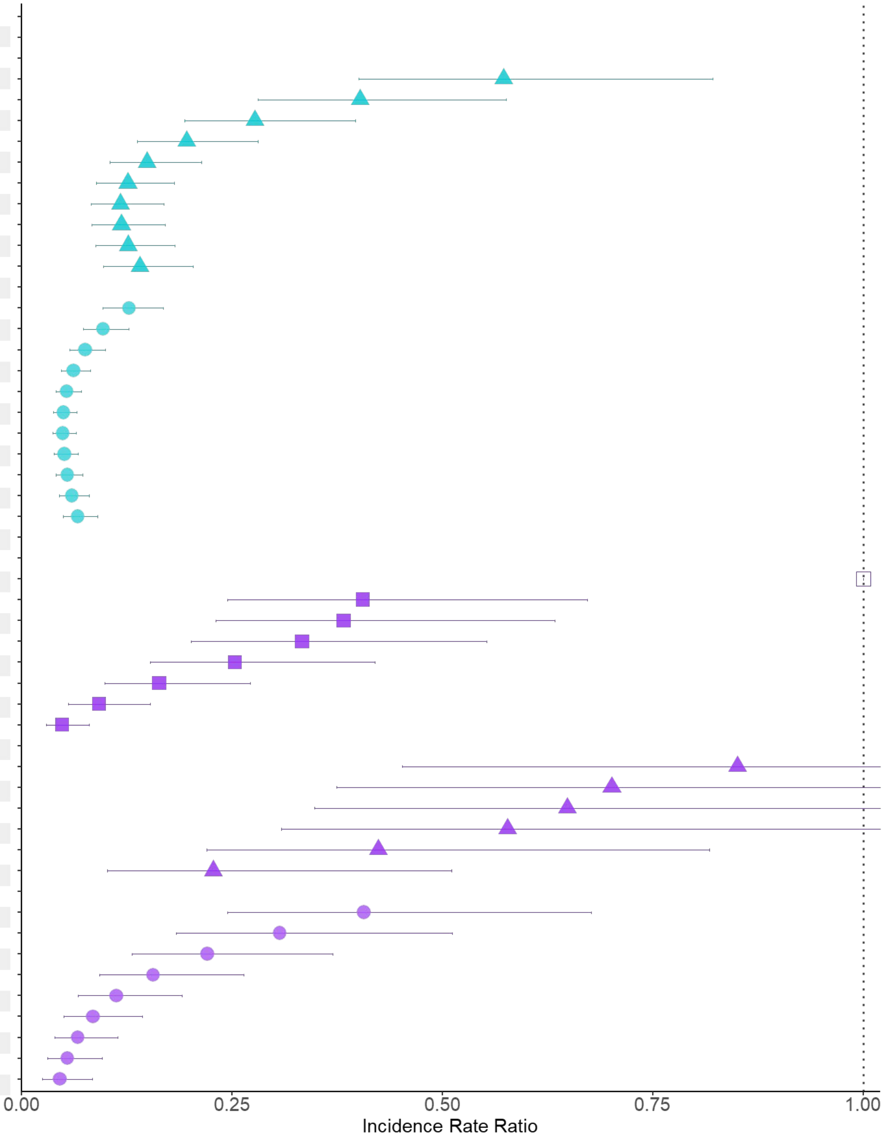

Supplementary Figure 3.26 PCV7 ST IPD all-site weighted average IRRs comparing the annual post-PCV10/13 IR to the average pre-PCV IR for adults aged 18–49 years

PCV Product and PCV7 Impact Strata	Number of Sites	Number of Cases	IRR (95%CI)
PCV13			
Moderate PCV7 impact			
Year −1	4	586	0.48 (0.31, 0.75)
Year 0	4	507	0.36 (0.23, 0.57)
Year 1	4	298	0.27 (0.17, 0.42)
Year 2	4	246	0.2 (0.13, 0.32)
Year 3	4	231	0.16 (0.1, 0.25)
Year 4	4	163	0.13 (0.08, 0.2)
Year 5	4	162	0.12 (0.07, 0.18)
Year 6	4	151	0.11 (0.07, 0.17)
Year 7	3	179	0.11 (0.07, 0.17)
Year 8	2	43	0.11 (0.07, 0.17)
Substantial PCV7 impact			
Year −1	10	411	0.17 (0.12, 0.23)
Year 0	10	239	0.13 (0.09, 0.18)
Year 1	10	112	0.1 (0.07, 0.14)
Year 2	10	108	0.08 (0.06, 0.12)
Year 3	10	94	0.07 (0.05, 0.11)
Year 4	10	87	0.07 (0.05, 0.1)
Year 5	10	103	0.07 (0.05, 0.1)
Year 6	10	135	0.07 (0.05, 0.1)
Year 7	7	87	0.07 (0.05, 0.1)
Year 8	4	33	0.08 (0.05, 0.11)
Year 9	1	28	0.08 (0.06, 0.12)
PCV10			
No PCV7 impact			
Year 0	--	--	--
Year 1	5	102	0.43 (0.23, 0.81)
Year 2	5	61	0.31 (0.17, 0.58)
Year 3	5	74	0.23 (0.12, 0.43)
Year 4	4	42	0.18 (0.09, 0.33)
Year 5	4	30	0.15 (0.08, 0.27)
Year 6	3	26	0.12 (0.07, 0.23)
Year 7	3	24	0.11 (0.06, 0.2)
Substantial PCV7 impact			
Year −1	3	29	0.38 (0.2, 0.72)
Year 0	3	11	0.29 (0.15, 0.54)
Year 1	3	20	0.21 (0.11, 0.4)
Year 2	1	11	0.16 (0.08, 0.29)
Year 3	1	1	0.12 (0.06, 0.22)
Year 4	1	5	0.09 (0.05, 0.18)
Year 5	1	2	0.08 (0.04, 0.14)
Year 6	1	3	0.06 (0.03, 0.12)
Year 7	1	3	0.05 (0.03, 0.11)

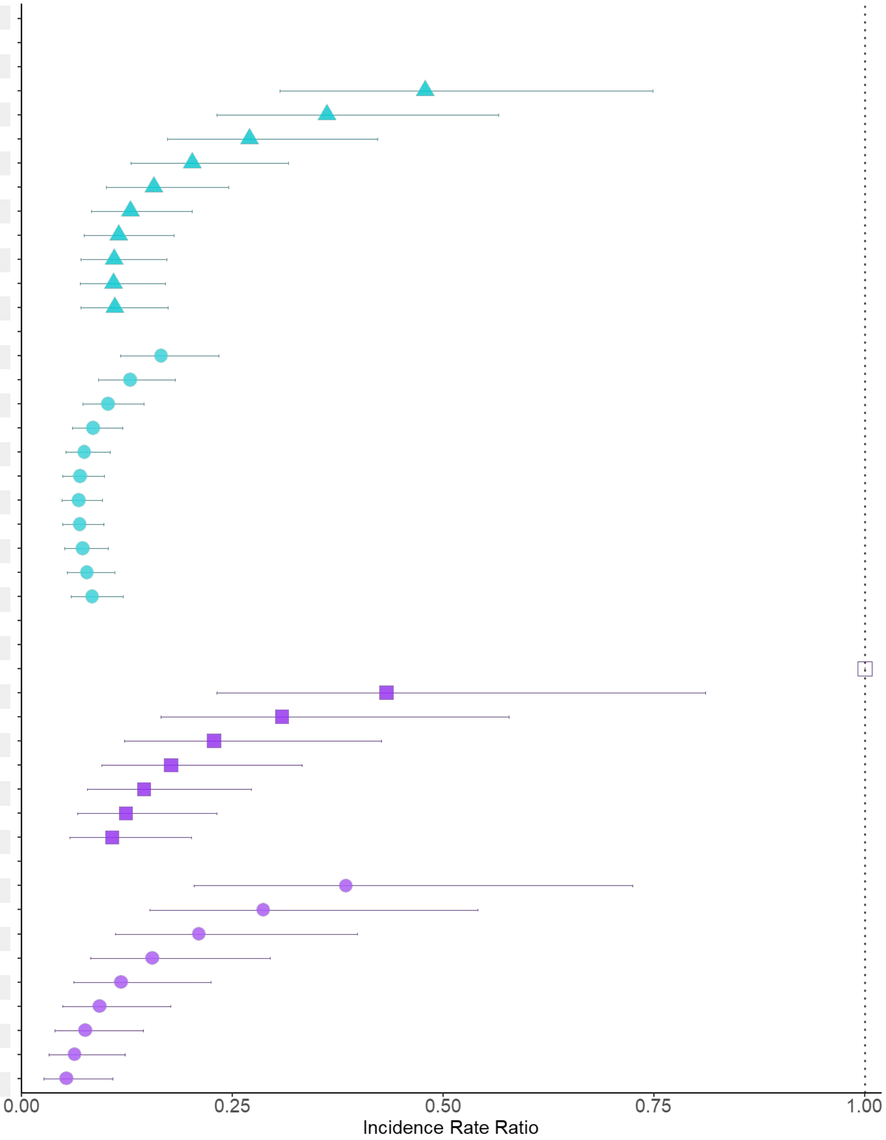

Supplementary Figure 3.27 PCV7 ST IPD all-site weighted average IRRs comparing the annual post-PCV10/13 IR to the average pre-PCV IR for adults aged 50–64 years

PCV Product and PCV7 Impact Strata	Number of Sites	Number of Cases	IRR (95%CI)
PCV13			
Moderate PCV7 impact			
Year −1	4	252	0.51 (0.29, 0.9)
Year 0	4	255	0.39 (0.22, 0.7)
Year 1	4	141	0.3 (0.17, 0.52)
Year 2	4	127	0.22 (0.12, 0.39)
Year 3	4	130	0.17 (0.1, 0.3)
Year 4	4	87	0.14 (0.08, 0.25)
Year 5	4	89	0.13 (0.07, 0.23)
Year 6	4	107	0.13 (0.07, 0.23)
Year 7	3	104	0.14 (0.08, 0.24)
Year 8	2	67	0.15 (0.08, 0.26)
Substantial PCV7 impact			
Year −1	10	424	0.19 (0.14, 0.25)
Year 0	10	270	0.15 (0.11, 0.2)
Year 1	10	130	0.12 (0.09, 0.16)
Year 2	10	99	0.1 (0.07, 0.13)
Year 3	10	120	0.08 (0.06, 0.11)
Year 4	10	110	0.08 (0.06, 0.11)
Year 5	10	124	0.08 (0.06, 0.1)
Year 6	10	157	0.08 (0.06, 0.1)
Year 7	7	103	0.08 (0.06, 0.11)
Year 8	4	36	0.08 (0.06, 0.11)
Year 9	1	31	0.09 (0.07, 0.12)
PCV10			
No PCV7 impact			
Year 0	--	--	--
Year 1	4	125	0.69 (0.5, 0.95)
Year 2	4	120	0.52 (0.38, 0.71)
Year 3	4	84	0.41 (0.3, 0.56)
Year 4	2	46	0.34 (0.25, 0.47)
Year 5	2	39	0.28 (0.21, 0.39)
Year 6	2	25	0.23 (0.17, 0.32)
Year 7	2	30	0.19 (0.14, 0.27)
Substantial PCV7 impact			
Year −1	3	29	0.23 (0.13, 0.4)
Year 0	3	27	0.15 (0.09, 0.26)
Year 1	3	17	0.1 (0.06, 0.17)
Year 2	1	12	0.07 (0.04, 0.12)
Year 3	1	4	0.05 (0.03, 0.08)
Year 4	1	2	0.04 (0.02, 0.06)
Year 5	1	4	0.03 (0.02, 0.05)
Year 6	1	4	0.03 (0.01, 0.05)
Year 7	1	3	0.02 (0.01, 0.04)

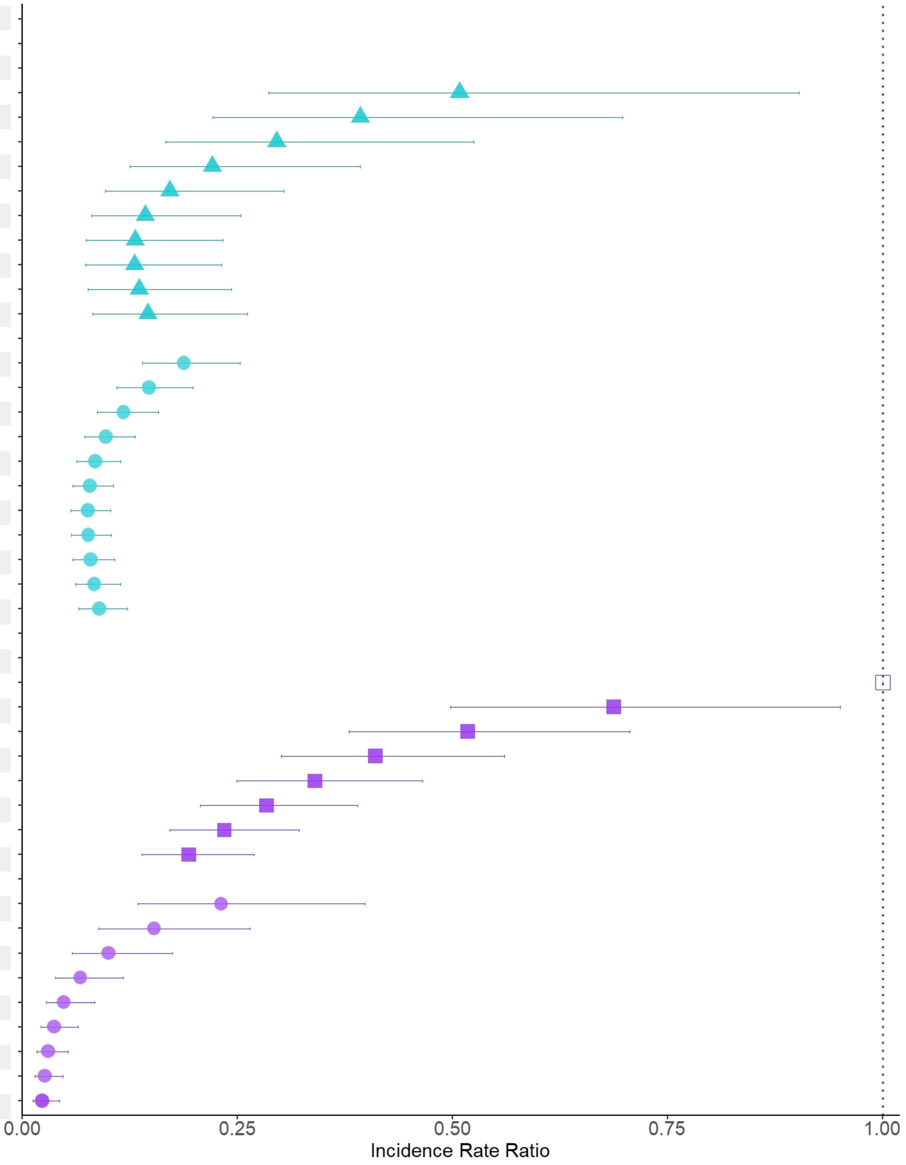

Supplementary Figure 3.28 PCV7 ST IPD all-site weighted average IRRs comparing the annual post-PCV10/13 IR to the average pre-PCV IR for adults aged ≥65 years

PCV Product and PCV7 Impact Strata	Number of Sites	Number of Cases	IRR (95%CI)
PCV13			
Moderate PCV7 impact			
Year −1	4	474	0.5 (0.28, 0.92)
Year 0	4	414	0.4 (0.22, 0.72)
Year 1	4	233	0.31 (0.17, 0.56)
Year 2	4	281	0.24 (0.13, 0.44)
Year 3	4	261	0.19 (0.1, 0.35)
Year 4	4	204	0.16 (0.09, 0.29)
Year 5	4	201	0.14 (0.07, 0.25)
Year 6	4	162	0.12 (0.07, 0.23)
Year 7	3	147	0.12 (0.06, 0.21)
Year 8	2	105	0.11 (0.06, 0.2)
Substantial PCV7 impact			
Year −1	10	878	0.16 (0.12, 0.22)
Year 0	10	517	0.12 (0.09, 0.17)
Year 1	10	247	0.09 (0.07, 0.13)
Year 2	10	182	0.07 (0.05, 0.1)
Year 3	10	161	0.06 (0.05, 0.09)
Year 4	10	150	0.06 (0.04, 0.08)
Year 5	10	166	0.05 (0.04, 0.07)
Year 6	10	199	0.06 (0.04, 0.08)
Year 7	7	138	0.06 (0.04, 0.08)
Year 8	4	85	0.06 (0.04, 0.08)
Year 9	1	69	0.06 (0.05, 0.09)
PCV10			
No PCV7 impact			
Year 0	--	--	--
Year 1	4	186	0.74 (0.54, 1.03)
Year 2	4	204	0.6 (0.44, 0.83)
Year 3	4	146	0.49 (0.36, 0.68)
Year 4	3	87	0.4 (0.29, 0.55)
Year 5	3	70	0.31 (0.22, 0.43)
Year 6	2	54	0.23 (0.16, 0.31)
Year 7	2	45	0.16 (0.12, 0.23)
Substantial PCV7 impact			
Year −1	3	90	0.3 (0.17, 0.54)
Year 0	3	58	0.21 (0.12, 0.38)
Year 1	3	45	0.15 (0.08, 0.26)
Year 2	1	22	0.11 (0.06, 0.19)
Year 3	1	18	0.08 (0.05, 0.14)
Year 4	1	26	0.07 (0.04, 0.12)
Year 5	1	20	0.06 (0.04, 0.11)
Year 6	1	18	0.06 (0.04, 0.11)
Year 7	1	13	0.07 (0.04, 0.12)

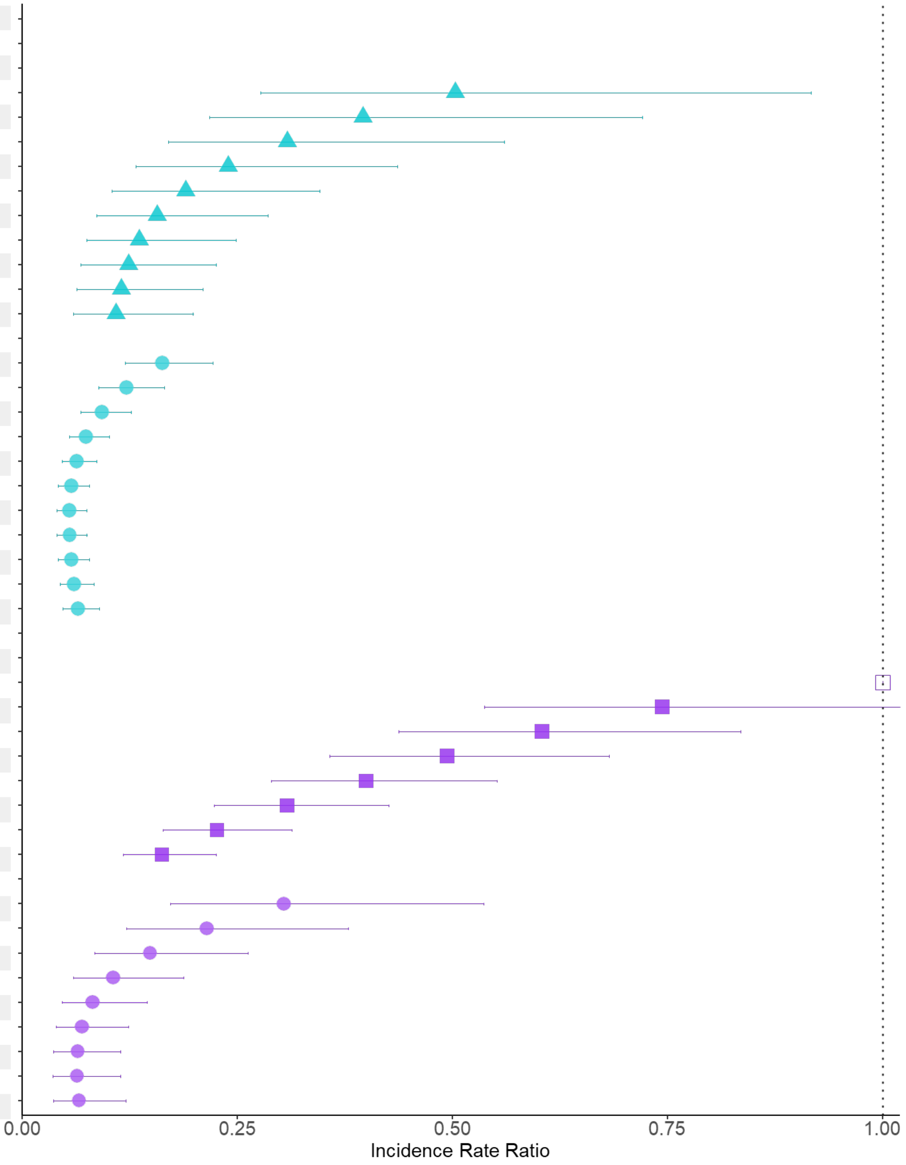

Supplementary Figure 3.28 STs 1, 5, & 7F (PCV10X-type) IPD all-site weighted average IRRs comparing the annual post-PCV10/13 IR to the average pre-PCV IR for **children aged <5 years**

PCV Product and PCV7 Impact Strata	Number of Sites	Number of Cases	IRR (95%CI)
PCV13			
Moderate PCV7 impact			
Year −1	6	285	1.58 (1.1, 2.26)
Year 0	6	375	1.19 (0.83, 1.7)
Year 1	6	333	0.78 (0.54, 1.11)
Year 2	6	211	0.45 (0.32, 0.65)
Year 3	6	85	0.24 (0.17, 0.34)
Year 4	6	42	0.12 (0.08, 0.17)
Year 5	6	13	0.06 (0.04, 0.08)
Year 6	6	18	0.02 (0.02, 0.03)
Year 7	5	4	0.01 (0.01, 0.01)
Year 8	3	5	0 (0, 0.01)
Substantial PCV7 impact			
Year −1	11	221	1.53 (1.17, 2)
Year 0	11	274	1.14 (0.88, 1.49)
Year 1	11	223	0.77 (0.59, 1)
Year 2	11	110	0.46 (0.35, 0.6)
Year 3	11	47	0.25 (0.19, 0.33)
Year 4	11	29	0.13 (0.1, 0.16)
Year 5	11	9	0.06 (0.04, 0.08)
Year 6	11	4	0.03 (0.02, 0.03)
Year 7	7	4	0.01 (0.01, 0.01)
Year 8	4	1	0 (0, 0.01)
Year 9	1	1	0 (0, 0)
PCV10			
No PCV7 impact			
Year −1	--	--	--
Year 0	4	29	0.71 (0.33, 1.5)
Year 1	4	14	0.58 (0.27, 1.22)
Year 2	4	10	0.47 (0.22, 0.99)
Year 3	4	6	0.37 (0.18, 0.79)
Year 4	3	7	0.27 (0.13, 0.58)
Year 5	3	4	0.18 (0.09, 0.39)
Year 6	2	1	0.11 (0.05, 0.24)
Year 7	2	0	0.07 (0.03, 0.14)
Moderate PCV7 impact			
Year −1	2	53	1.89 (1.01, 3.53)
Year 0	2	46	1.32 (0.71, 2.47)
Year 1	2	18	0.82 (0.44, 1.54)
Year 2	2	19	0.54 (0.29, 1.01)
Year 3	1	11	0.44 (0.23, 0.84)
Year 4	1	7	0.47 (0.23, 0.96)
Substantial PCV7 impact			
Year −1	4	5	1.58 (1.01, 2.46)
Year 0	4	9	1.4 (0.9, 2.2)
Year 1	4	19	1.16 (0.74, 1.83)
Year 2	1	15	0.9 (0.57, 1.42)
Year 3	1	1	0.65 (0.41, 1.03)
Year 4	1	3	0.45 (0.28, 0.71)
Year 5	1	0	0.29 (0.18, 0.47)
Year 6	1	1	0.18 (0.11, 0.31)
Year 7	1	0	0.11 (0.06, 0.2)

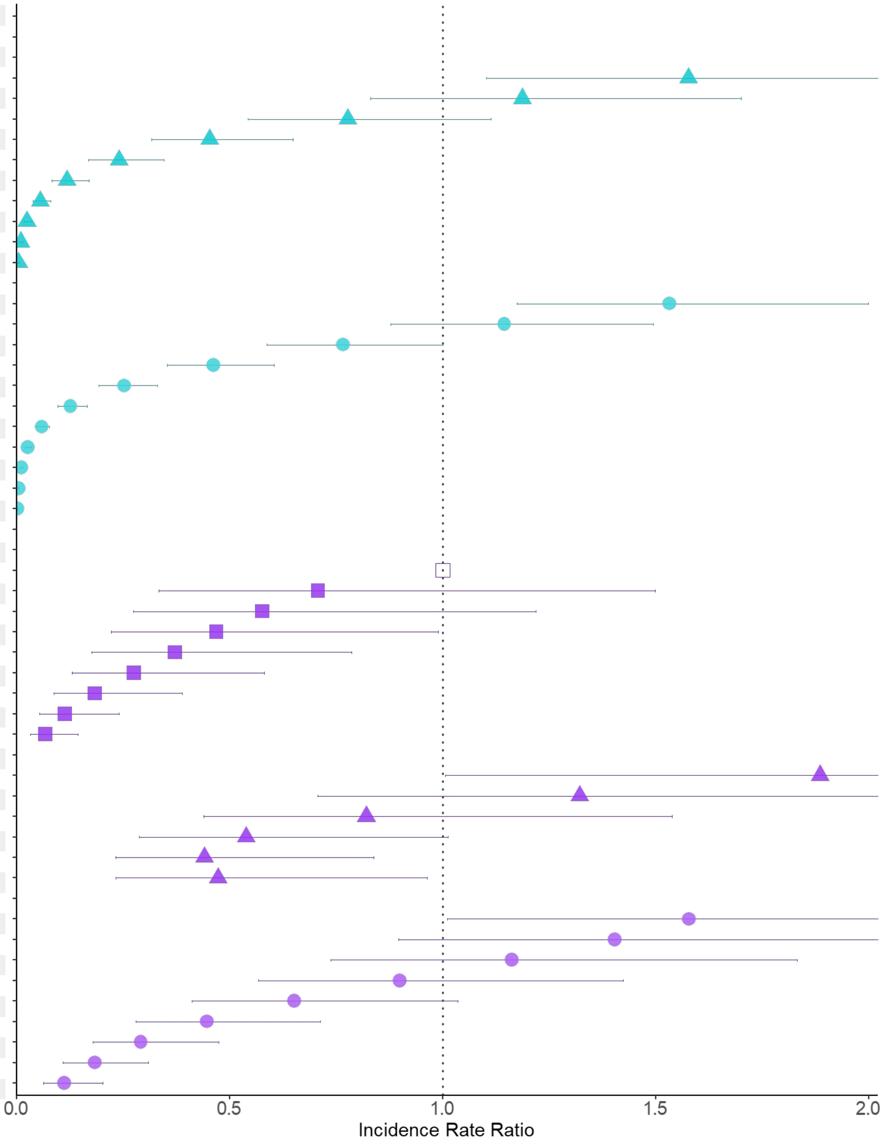

Supplementary Figure 3.29 STs 1, 5, & 7F (PCV10X-type) IPD all-site weighted average IRRs comparing the annual post-PCV10/13 IR to the average pre-PCV IR for **children aged 5–17 years**

PCV Product and PCV7 Impact Strata	Number of Sites	Number of Cases	IRR (95%CI)
PCV13			
Moderate PCV7 impact			
Year −1	5	227	1.16 (0.81, 1.64)
Year 0	6	286	1.01 (0.71, 1.44)
Year 1	6	227	0.81 (0.57, 1.15)
Year 2	6	100	0.59 (0.42, 0.84)
Year 3	6	84	0.39 (0.28, 0.56)
Year 4	6	42	0.24 (0.17, 0.34)
Year 5	6	27	0.13 (0.09, 0.19)
Year 6	6	11	0.07 (0.05, 0.1)
Year 7	5	7	0.03 (0.02, 0.05)
Year 8	3	6	0.02 (0.01, 0.02)
Substantial PCV7 impact			
Year −1	10	186	1.01 (0.77, 1.32)
Year 0	10	191	0.88 (0.67, 1.15)
Year 1	10	155	0.71 (0.54, 0.93)
Year 2	10	88	0.52 (0.4, 0.68)
Year 3	10	60	0.34 (0.26, 0.45)
Year 4	10	45	0.2 (0.15, 0.26)
Year 5	10	25	0.1 (0.08, 0.14)
Year 6	10	12	0.05 (0.04, 0.07)
Year 7	7	4	0.02 (0.02, 0.03)
Year 8	4	0	0.01 (0.01, 0.01)
Year 9	1	0	0 (0, 0.01)
PCV10			
No PCV7 impact			
Year 0	--	--	--
Year 1	5	25	0.77 (0.52, 1.14)
Year 2	5	13	0.55 (0.38, 0.78)
Year 3	5	6	0.39 (0.28, 0.56)
Year 4	4	4	0.28 (0.2, 0.4)
Year 5	4	4	0.18 (0.12, 0.26)
Year 6	3	0	0.1 (0.07, 0.15)
Year 7	3	1	0.06 (0.04, 0.09)
Moderate PCV7 impact			
Year −1	2	83	1.26 (0.68, 2.36)
Year 0	2	35	0.7 (0.38, 1.3)
Year 1	2	16	0.47 (0.26, 0.87)
Year 2	2	16	0.38 (0.21, 0.7)
Year 3	1	8	0.35 (0.19, 0.67)
Year 4	1	10	0.37 (0.17, 0.83)
Substantial PCV7 impact			
Year −1	3	8	1.21 (0.74, 1.99)
Year 0	3	4	1.3 (0.79, 2.14)
Year 1	3	38	1.34 (0.81, 2.21)
Year 2	1	2	1.29 (0.78, 2.13)
Year 3	1	3	1.11 (0.67, 1.85)
Year 4	1	4	0.84 (0.51, 1.4)
Year 5	1	2	0.57 (0.34, 0.95)
Year 6	1	1	0.35 (0.2, 0.6)
Year 7	1	1	0.2 (0.11, 0.36)

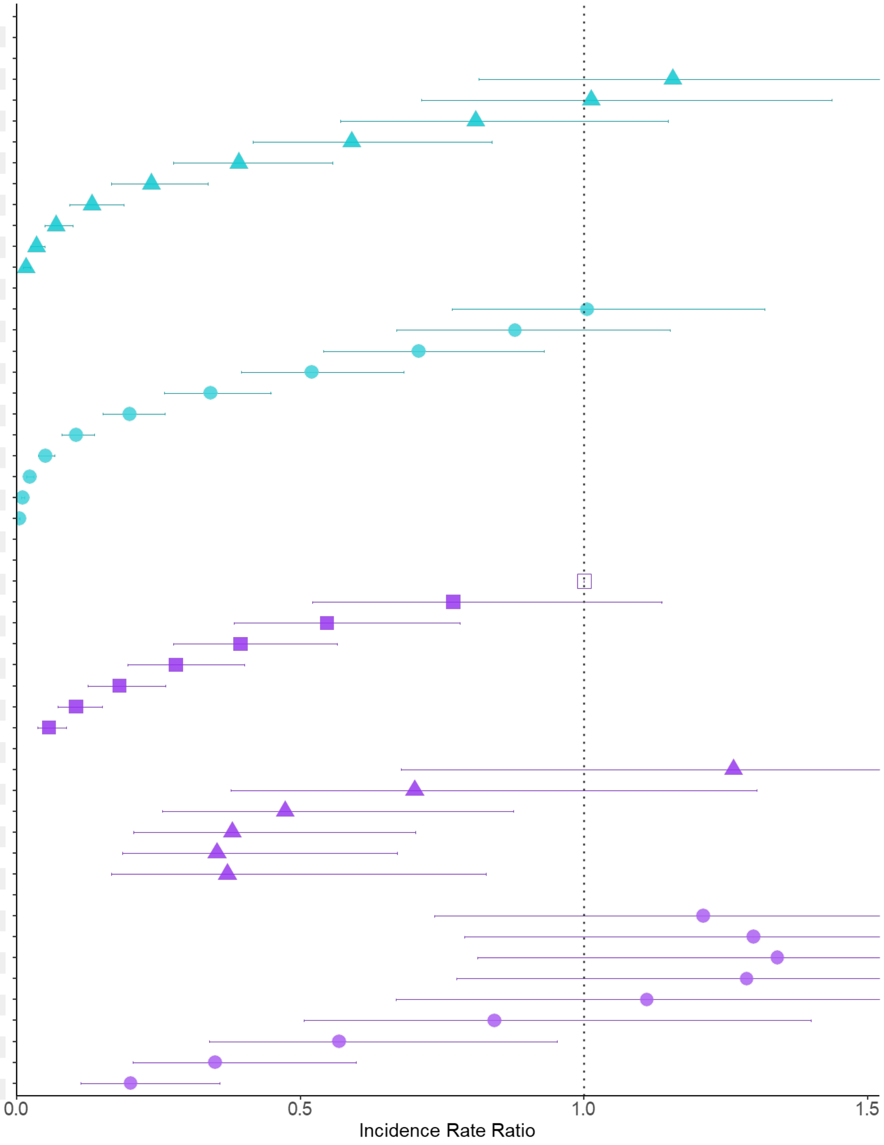

Supplementary Figure 3.30 STs 1, 5, & 7F (PCV10X-type) IPD all-site weighted average IRRs comparing the annual post-PCV10/13 IR to the average pre-PCV IR for **adults aged 18–49 years**

PCV Product and PCV7 Impact Strata	Number of Sites	Number of Cases	IRR (95%CI)
PCV13			
Moderate PCV7 impact			
Year −1	4	438	1.51 (1.06, 2.15)
Year 0	4	521	1.42 (0.99, 2.02)
Year 1	4	403	1.15 (0.81, 1.64)
Year 2	4	347	0.82 (0.58, 1.17)
Year 3	4	257	0.53 (0.37, 0.75)
Year 4	4	140	0.32 (0.23, 0.46)
Year 5	4	110	0.19 (0.13, 0.27)
Year 6	4	50	0.11 (0.08, 0.15)
Year 7	3	35	0.06 (0.04, 0.09)
Year 8	2	12	0.03 (0.02, 0.05)
Substantial PCV7 impact			
Year −1	10	944	1.57 (1.02, 2.42)
Year 0	10	841	1.38 (0.89, 2.12)
Year 1	10	702	1.13 (0.73, 1.74)
Year 2	10	496	0.86 (0.56, 1.33)
Year 3	10	331	0.61 (0.4, 0.95)
Year 4	10	187	0.41 (0.27, 0.64)
Year 5	10	128	0.26 (0.17, 0.41)
Year 6	10	85	0.16 (0.1, 0.25)
Year 7	7	48	0.1 (0.06, 0.15)
Year 8	4	22	0.06 (0.04, 0.09)
Year 9	1	7	0.03 (0.02, 0.05)
PCV10			
No PCV7 impact			
Year 0	--	--	--
Year 1	5	47	0.82 (0.55, 1.22)
Year 2	5	40	0.52 (0.35, 0.77)
Year 3	5	23	0.34 (0.23, 0.5)
Year 4	4	6	0.23 (0.15, 0.33)
Year 5	4	8	0.16 (0.1, 0.23)
Year 6	3	7	0.11 (0.07, 0.16)
Year 7	3	3	0.08 (0.05, 0.12)
Substantial PCV7 impact			
Year −1	3	44	0.91 (0.41, 2.01)
Year 0	3	45	1 (0.45, 2.19)
Year 1	3	77	1.06 (0.48, 2.33)
Year 2	1	41	1.03 (0.47, 2.28)
Year 3	1	27	0.88 (0.4, 1.95)
Year 4	1	31	0.65 (0.3, 1.44)
Year 5	1	14	0.42 (0.19, 0.94)
Year 6	1	8	0.25 (0.11, 0.55)
Year 7	1	7	0.14 (0.06, 0.3)

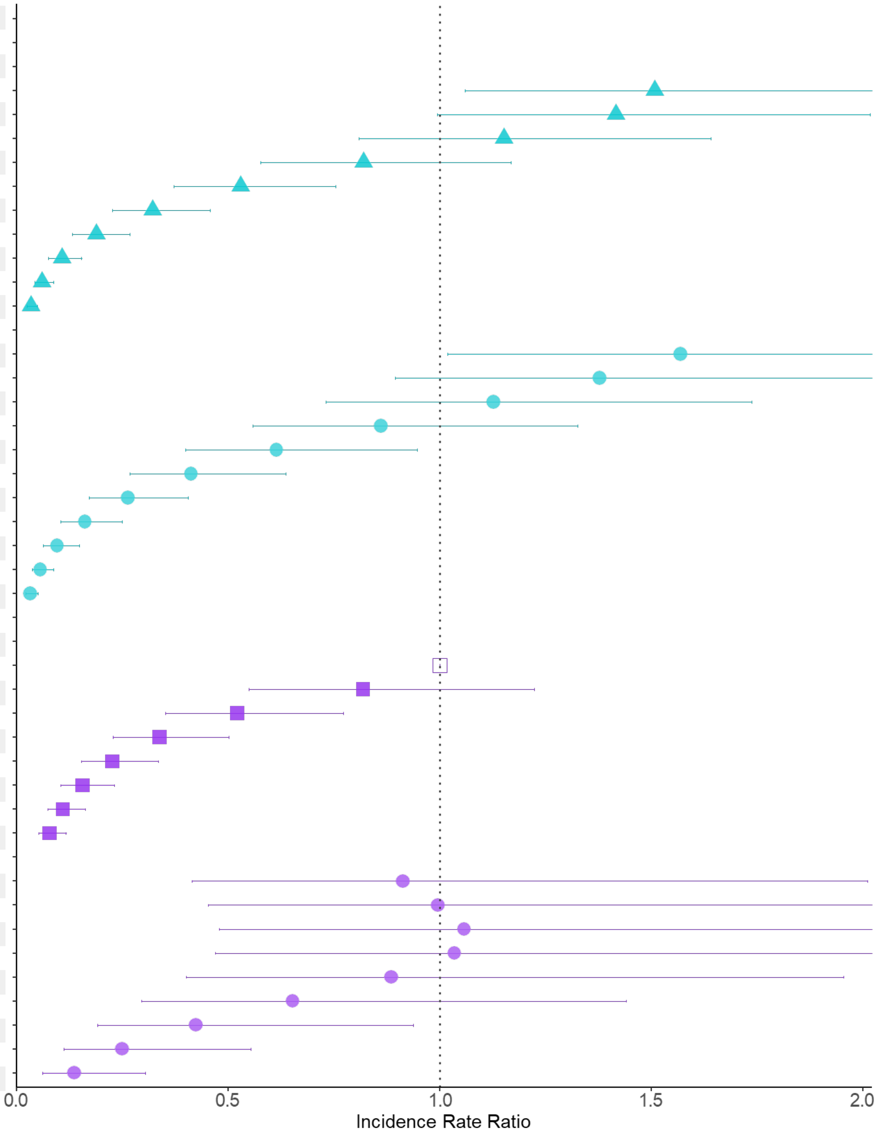

Supplementary Figure 3.31 STs 1, 5, & 7F (PCV10X-type) IPD all-site weighted average IRRs comparing the annual post-PCV10/13 IR to the average pre-PCV IR for **adults aged 50–64 years**

PCV Product and PCV7 Impact Strata	Number of Sites	Number of Cases	IRR (95%CI)
PCV13			
Moderate PCV7 impact			
Year −1	4	150	1.72 (1.11, 2.66)
Year 0	4	224	1.66 (1.07, 2.57)
Year 1	4	175	1.36 (0.88, 2.1)
Year 2	4	227	0.95 (0.62, 1.48)
Year 3	4	158	0.61 (0.39, 0.94)
Year 4	4	76	0.36 (0.24, 0.56)
Year 5	4	67	0.22 (0.14, 0.33)
Year 6	4	26	0.13 (0.08, 0.2)
Year 7	3	29	0.07 (0.05, 0.12)
Year 8	2	10	0.04 (0.03, 0.07)
Substantial PCV7 impact			
Year −1	10	489	1.76 (1.37, 2.28)
Year 0	10	540	1.59 (1.23, 2.05)
Year 1	10	477	1.33 (1.03, 1.72)
Year 2	10	306	1.03 (0.8, 1.33)
Year 3	10	221	0.75 (0.58, 0.97)
Year 4	10	133	0.51 (0.4, 0.66)
Year 5	10	98	0.33 (0.26, 0.43)
Year 6	10	68	0.21 (0.16, 0.27)
Year 7	7	51	0.13 (0.1, 0.16)
Year 8	4	14	0.07 (0.06, 0.1)
Year 9	1	11	0.04 (0.03, 0.06)
PCV10			
No PCV7 impact			
Year 0	--	--	--
Year 1	4	25	0.66 (0.34, 1.26)
Year 2	4	21	0.49 (0.26, 0.92)
Year 3	4	16	0.44 (0.23, 0.83)
Year 4	2	6	0.45 (0.24, 0.86)
Year 5	2	6	0.44 (0.23, 0.85)
Year 6	2	3	0.39 (0.21, 0.75)
Year 7	2	5	0.33 (0.17, 0.64)
Substantial PCV7 impact			
Year −1	3	47	2.24 (1.4, 3.57)
Year 0	3	35	2.34 (1.46, 3.74)
Year 1	3	75	2.24 (1.4, 3.59)
Year 2	1	39	1.96 (1.22, 3.15)
Year 3	1	22	1.56 (0.97, 2.51)
Year 4	1	20	1.14 (0.71, 1.83)
Year 5	1	16	0.77 (0.48, 1.24)
Year 6	1	9	0.49 (0.3, 0.79)
Year 7	1	10	0.3 (0.18, 0.49)

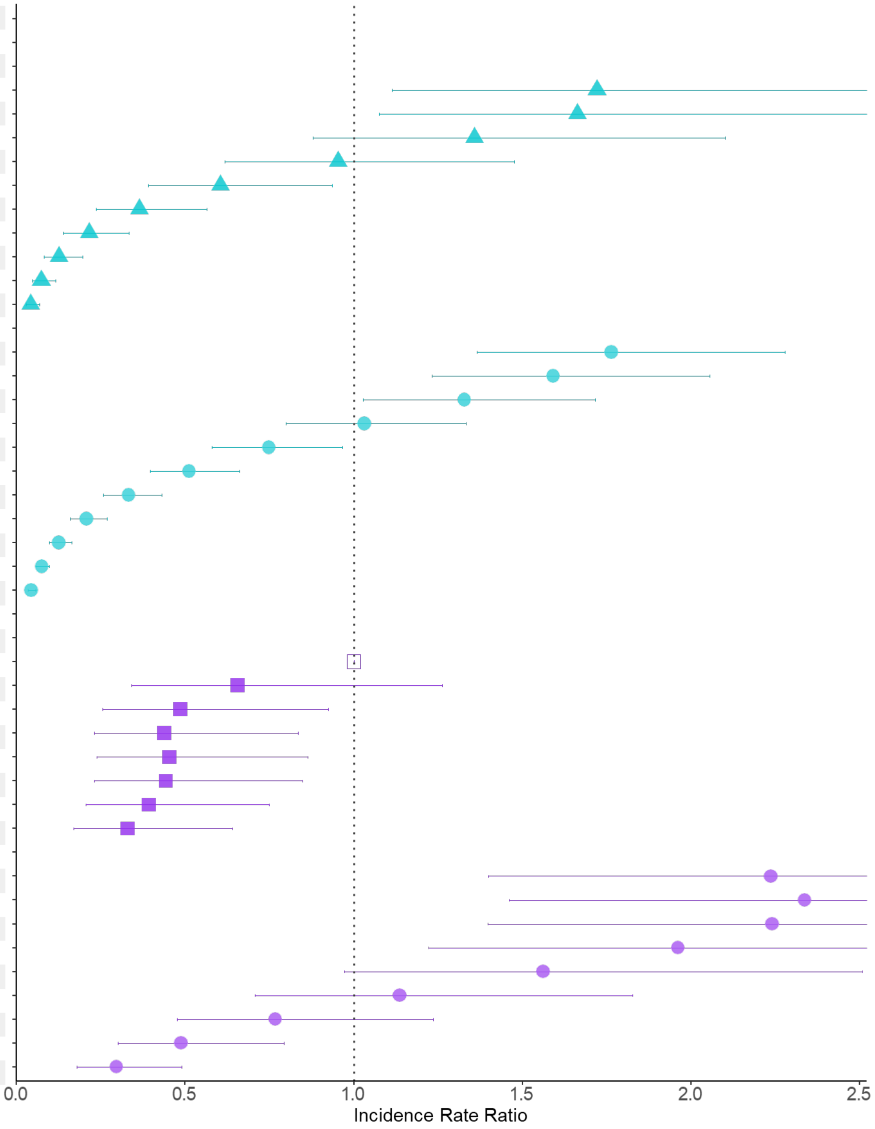

Supplementary Figure 3.32 STs 1, 5, & 7F (PCV10X-type) IPD all-site weighted average IRRs comparing the annual post-PCV10/13 IR to the average pre-PCV IR for **adults aged ≥65 years**

PCV Product and PCV7 Impact Strata	Number of Sites	Number of Cases	IRR (95%CI)
PCV13			
Moderate PCV7 impact			
Year −1	4	218	1.38 (0.81, 2.33)
Year 0	4	266	1.32 (0.78, 2.23)
Year 1	4	256	1.14 (0.67, 1.93)
Year 2	4	340	0.88 (0.52, 1.5)
Year 3	4	242	0.62 (0.36, 1.04)
Year 4	4	169	0.38 (0.23, 0.65)
Year 5	4	99	0.22 (0.13, 0.37)
Year 6	4	56	0.11 (0.07, 0.19)
Year 7	3	25	0.06 (0.03, 0.1)
Year 8	2	19	0.03 (0.02, 0.05)
Substantial PCV7 impact			
Year −1	10	491	1.49 (1.12, 1.98)
Year 0	10	472	1.35 (1.01, 1.8)
Year 1	10	447	1.14 (0.86, 1.52)
Year 2	10	318	0.89 (0.67, 1.18)
Year 3	10	232	0.63 (0.48, 0.85)
Year 4	10	110	0.42 (0.31, 0.56)
Year 5	10	101	0.26 (0.19, 0.34)
Year 6	10	69	0.15 (0.11, 0.2)
Year 7	7	35	0.08 (0.06, 0.11)
Year 8	4	19	0.04 (0.03, 0.06)
Year 9	1	8	0.02 (0.02, 0.03)
PCV10			
No PCV7 impact			
Year 0	--	--	--
Year 1	3	29	0.73 (0.52, 1.01)
Year 2	3	25	0.64 (0.46, 0.89)
Year 3	3	21	0.54 (0.39, 0.75)
Year 4	2	10	0.41 (0.3, 0.57)
Year 5	2	7	0.28 (0.2, 0.39)
Year 6	2	4	0.18 (0.13, 0.25)
Year 7	2	1	0.11 (0.08, 0.15)
Substantial PCV7 impact			
Year −1	3	62	1.11 (0.65, 1.87)
Year 0	3	52	1.12 (0.66, 1.89)
Year 1	3	77	1.08 (0.64, 1.82)
Year 2	1	53	0.97 (0.57, 1.64)
Year 3	1	46	0.8 (0.47, 1.36)
Year 4	1	47	0.61 (0.36, 1.04)
Year 5	1	28	0.44 (0.26, 0.74)
Year 6	1	20	0.29 (0.17, 0.5)
Year 7	1	12	0.19 (0.11, 0.32)

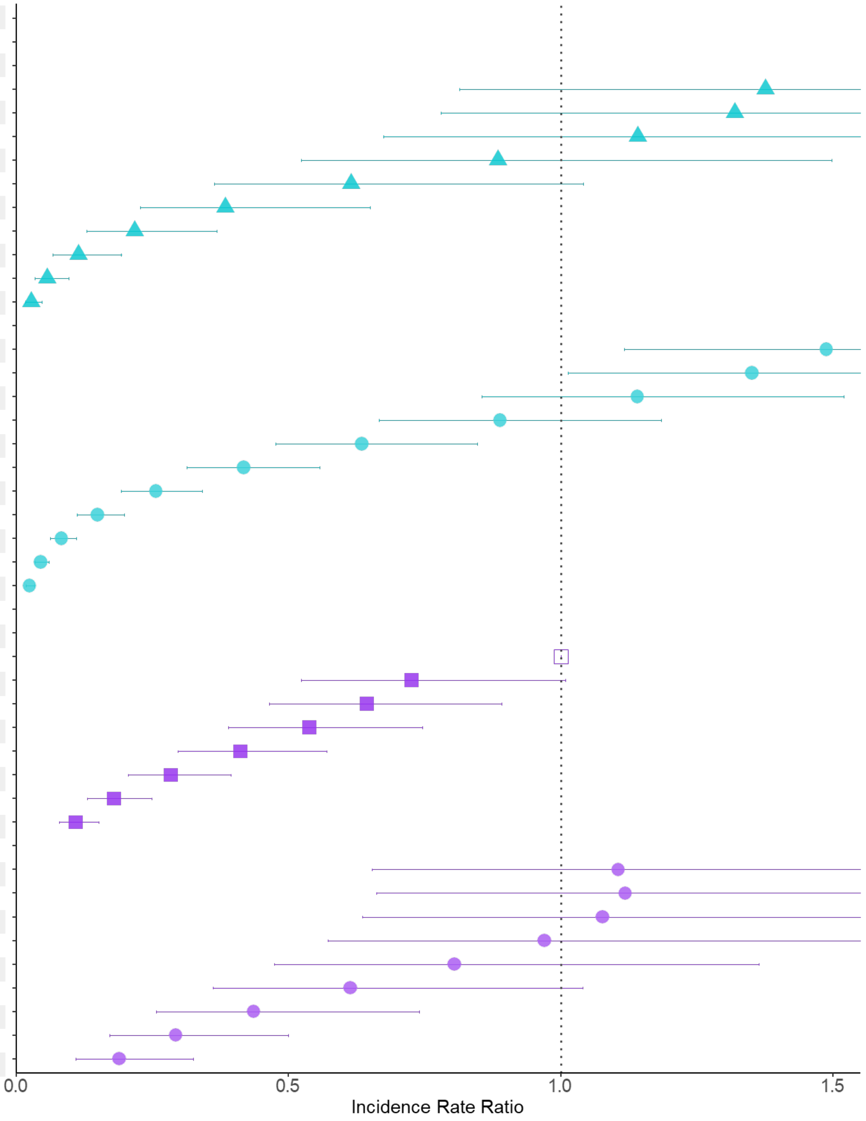

Supplementary Figure 3.33 All PCV10 ST IPD all-site weighted average IRRs comparing the annual post-PCV10/13 IR to the average pre-PCV IR for children aged <5 years

PCV Product and PCV7 Impact Strata	Number of Sites	Number of Cases	IRR (95%CI)
PCV13			
Moderate PCV7 impact			
Year −1	6	1076	0.41 (0.28, 0.6)
Year 0	6	819	0.27 (0.18, 0.39)
Year 1	6	540	0.17 (0.11, 0.24)
Year 2	6	322	0.1 (0.07, 0.15)
Year 3	6	168	0.07 (0.05, 0.1)
Year 4	6	105	0.05 (0.03, 0.07)
Year 5	6	57	0.04 (0.02, 0.05)
Year 6	6	73	0.03 (0.02, 0.04)
Year 7	5	46	0.02 (0.02, 0.03)
Year 8	3	52	0.02 (0.01, 0.03)
Substantial PCV7 impact			
Year −1	10	328	0.1 (0.07, 0.13)
Year 0	10	326	0.07 (0.05, 0.1)
Year 1	10	258	0.05 (0.04, 0.07)
Year 2	10	136	0.04 (0.03, 0.05)
Year 3	10	68	0.03 (0.02, 0.04)
Year 4	10	61	0.02 (0.01, 0.03)
Year 5	10	45	0.01 (0.01, 0.02)
Year 6	10	42	0.01 (0.01, 0.02)
Year 7	6	45	0.01 (0.01, 0.01)
Year 8	4	15	0.01 (0.01, 0.01)
Year 9	1	12	0.01 (0, 0.01)
PCV10			
No PCV7 impact			
Year −1	--	--	--
Year 0	4	166	0.63 (0.33, 1.18)
Year 1	4	135	0.38 (0.2, 0.7)
Year 2	4	83	0.22 (0.12, 0.41)
Year 3	4	39	0.12 (0.07, 0.23)
Year 4	3	27	0.07 (0.04, 0.14)
Year 5	3	7	0.05 (0.03, 0.1)
Year 6	2	5	0.04 (0.02, 0.08)
Year 7	2	4	0.04 (0.02, 0.07)
Moderate PCV7 impact			
Year −1	2	211	0.46 (0.24, 0.9)
Year 0	2	161	0.3 (0.15, 0.58)
Year 1	2	114	0.19 (0.1, 0.37)
Year 2	2	64	0.12 (0.06, 0.23)
Year 3	1	45	0.07 (0.03, 0.13)
Year 4	1	15	0.03 (0.02, 0.07)
Substantial PCV7 impact			
Year −1	3	8	0.13 (0.08, 0.23)
Year 0	3	9	0.11 (0.06, 0.18)
Year 1	3	23	0.08 (0.05, 0.15)
Year 2	1	18	0.07 (0.04, 0.12)
Year 3	1	2	0.06 (0.03, 0.1)
Year 4	1	4	0.05 (0.03, 0.08)
Year 5	1	1	0.04 (0.02, 0.07)
Year 6	1	3	0.04 (0.02, 0.07)
Year 7	1	1	0.03 (0.02, 0.06)

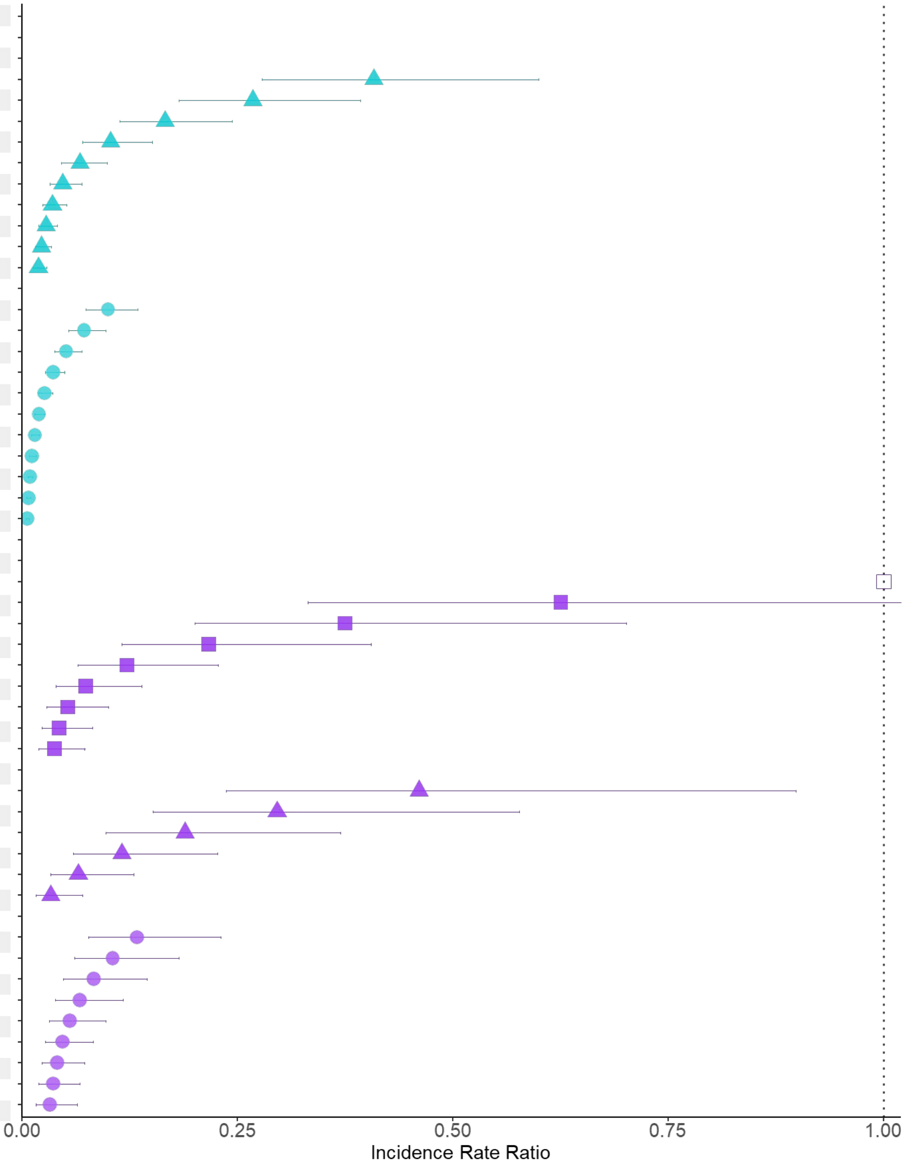

Supplementary Figure 3.34 All PCV10 ST IPD all-site weighted average IRRs comparing the annual post-PCV10/13 IR to the average pre-PCV IR for **children aged 5–17 years**

PCV Product and PCV7 Impact Strata	Number of Sites	Number of Cases	IRR (95%CI)
PCV13			
Moderate PCV7 impact			
Year −1	5	432	0.97 (0.75, 1.24)
Year 0	6	444	0.78 (0.6, 1)
Year 1	6	306	0.58 (0.45, 0.74)
Year 2	6	138	0.41 (0.32, 0.53)
Year 3	6	126	0.29 (0.22, 0.37)
Year 4	6	68	0.2 (0.16, 0.26)
Year 5	6	60	0.14 (0.11, 0.18)
Year 6	6	36	0.1 (0.08, 0.13)
Year 7	5	31	0.07 (0.05, 0.09)
Year 8	3	13	0.05 (0.04, 0.07)
Substantial PCV7 impact			
Year −1	10	246	0.41 (0.34, 0.49)
Year 0	10	242	0.33 (0.27, 0.4)
Year 1	10	168	0.26 (0.22, 0.32)
Year 2	10	110	0.2 (0.17, 0.24)
Year 3	10	81	0.15 (0.12, 0.18)
Year 4	10	58	0.11 (0.09, 0.13)
Year 5	10	51	0.08 (0.06, 0.09)
Year 6	10	35	0.05 (0.04, 0.06)
Year 7	7	20	0.04 (0.03, 0.04)
Year 8	4	4	0.02 (0.02, 0.03)
Year 9	1	5	0.02 (0.01, 0.02)
PCV10			
No PCV7 impact			
Year 0	--	--	--
Year 1	5	36	0.76 (0.57, 1.01)
Year 2	5	24	0.59 (0.46, 0.77)
Year 3	5	18	0.45 (0.35, 0.59)
Year 4	4	9	0.33 (0.25, 0.42)
Year 5	4	8	0.21 (0.16, 0.28)
Year 6	3	0	0.12 (0.09, 0.16)
Year 7	3	3	0.07 (0.05, 0.09)
Moderate PCV7 impact			
Year −1	2	114	0.95 (0.61, 1.49)
Year 0	2	46	0.61 (0.39, 0.96)
Year 1	2	39	0.48 (0.31, 0.75)
Year 2	2	31	0.41 (0.27, 0.64)
Year 3	1	15	0.35 (0.22, 0.56)
Year 4	1	13	0.27 (0.14, 0.51)
Substantial PCV7 impact			
Year −1	3	12	0.74 (0.52, 1.06)
Year 0	3	8	0.74 (0.52, 1.06)
Year 1	3	40	0.71 (0.5, 1.02)
Year 2	1	2	0.65 (0.45, 0.94)
Year 3	1	4	0.55 (0.38, 0.8)
Year 4	1	5	0.43 (0.3, 0.63)
Year 5	1	2	0.31 (0.21, 0.46)
Year 6	1	1	0.21 (0.14, 0.32)
Year 7	1	2	0.14 (0.09, 0.22)

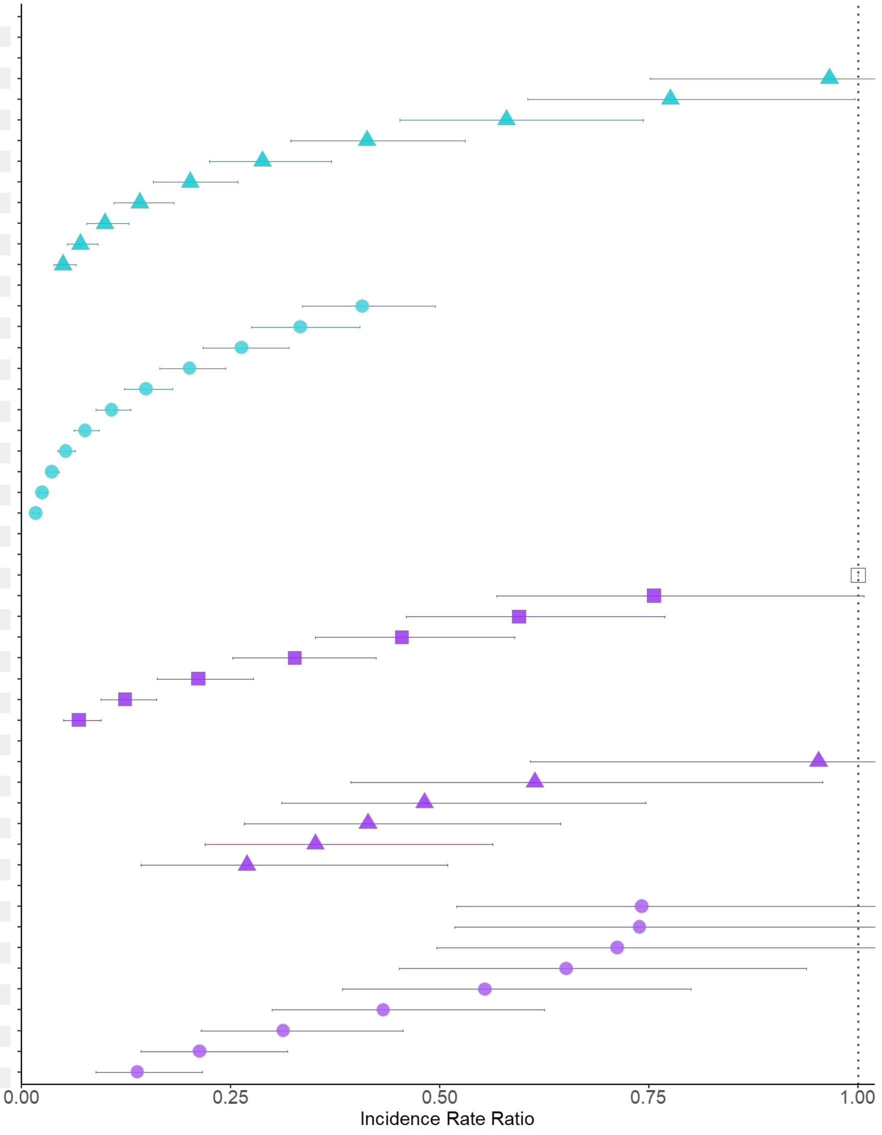

Supplementary Figure 3.35 All PCV10 ST IPD all-site weighted average IRRs comparing the annual post-PCV10/13 IR to the average pre-PCV IR for adults aged 18–49 years

PCV Product and PCV7 Impact Strata	Number of Sites	Number of Cases	IRR (95%CI)
PCV13			
Moderate PCV7 impact			
Year −1	5	1024	0.9 (0.78, 1.05)
Year 0	5	1029	0.77 (0.67, 0.89)
Year 1	5	701	0.62 (0.54, 0.72)
Year 2	5	593	0.47 (0.4, 0.54)
Year 3	5	488	0.34 (0.29, 0.39)
Year 4	5	303	0.24 (0.21, 0.28)
Year 5	5	272	0.17 (0.15, 0.2)
Year 6	5	201	0.12 (0.11, 0.14)
Year 7	4	214	0.09 (0.08, 0.1)
Year 8	2	55	0.06 (0.05, 0.07)
Substantial PCV7 impact			
Year −1	10	1355	0.51 (0.38, 0.69)
Year 0	10	1080	0.43 (0.32, 0.58)
Year 1	10	814	0.35 (0.26, 0.47)
Year 2	10	604	0.27 (0.2, 0.37)
Year 3	10	425	0.21 (0.16, 0.29)
Year 4	10	274	0.17 (0.12, 0.22)
Year 5	10	231	0.13 (0.09, 0.17)
Year 6	10	220	0.1 (0.07, 0.13)
Year 7	7	135	0.07 (0.05, 0.1)
Year 8	4	55	0.06 (0.04, 0.08)
Year 9	1	35	0.04 (0.03, 0.06)
PCV10			
No PCV7 impact			
Year 0	--	--	--
Year 1	5	149	0.62 (0.37, 1.03)
Year 2	5	101	0.39 (0.24, 0.66)
Year 3	5	97	0.26 (0.16, 0.44)
Year 4	4	48	0.19 (0.11, 0.31)
Year 5	4	38	0.15 (0.09, 0.24)
Year 6	3	33	0.12 (0.07, 0.2)
Year 7	3	27	0.11 (0.06, 0.18)
Substantial PCV7 impact			
Year −1	3	73	0.56 (0.32, 0.98)
Year 0	3	56	0.55 (0.31, 0.95)
Year 1	3	97	0.52 (0.3, 0.91)
Year 2	1	52	0.47 (0.27, 0.82)
Year 3	1	28	0.39 (0.22, 0.68)
Year 4	1	36	0.29 (0.17, 0.51)
Year 5	1	16	0.2 (0.12, 0.36)
Year 6	1	11	0.13 (0.08, 0.23)
Year 7	1	10	0.08 (0.05, 0.15)

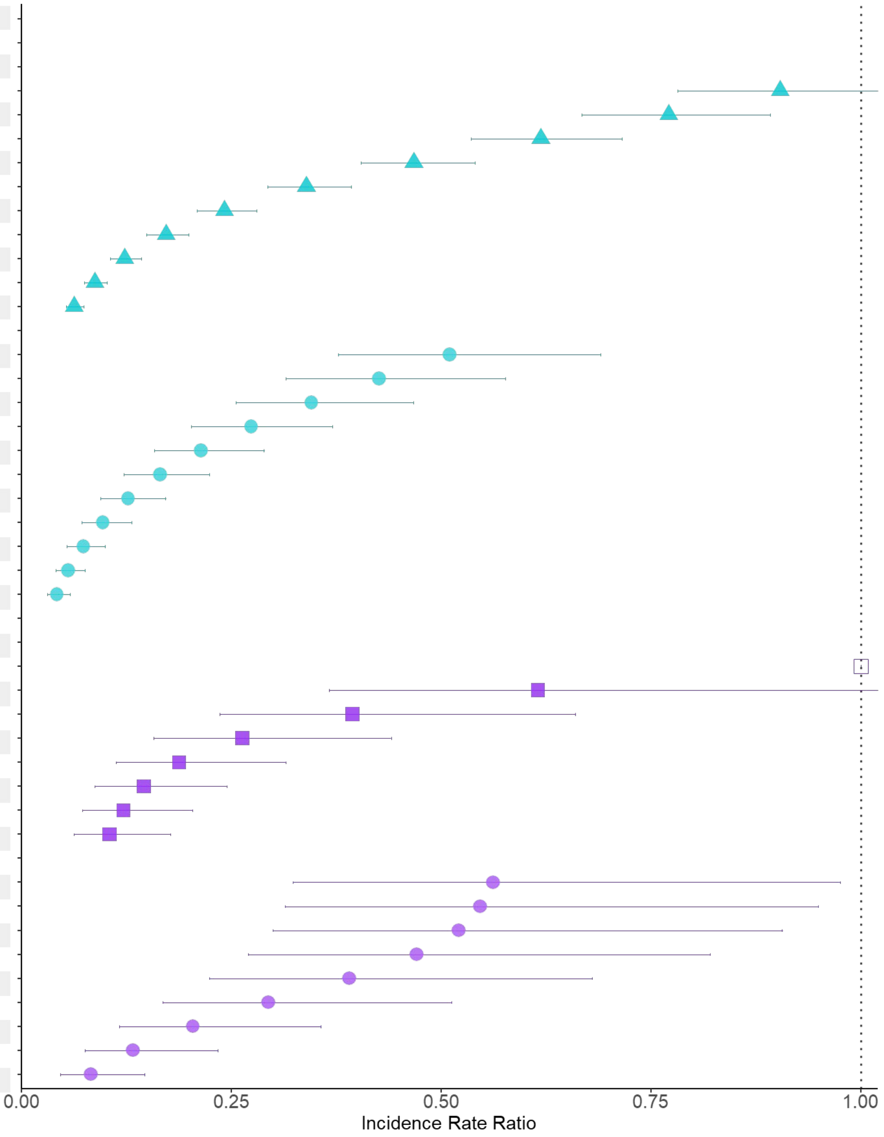

Supplementary Figure 3.36 All PCV10 ST IPD all-site weighted average IRRs comparing the annual post-PCV10/13 IR to the average pre-PCV IR for **adults aged 50–64 years**

PCV Product and PCV7 Impact Strata	Number of Sites	Number of Cases	IRR (95%CI)
PCV13			
Moderate PCV7 impact			
Year −1	5	402	0.81 (0.7, 0.94)
Year 0	5	479	0.69 (0.59, 0.8)
Year 1	5	316	0.55 (0.47, 0.64)
Year 2	5	354	0.42 (0.36, 0.48)
Year 3	5	288	0.31 (0.26, 0.36)
Year 4	5	163	0.23 (0.19, 0.26)
Year 5	5	156	0.17 (0.15, 0.2)
Year 6	5	133	0.13 (0.12, 0.16)
Year 7	4	133	0.11 (0.09, 0.13)
Year 8	2	77	0.09 (0.07, 0.1)
Substantial PCV7 impact			
Year −1	10	913	0.46 (0.38, 0.55)
Year 0	10	810	0.39 (0.32, 0.47)
Year 1	10	607	0.32 (0.27, 0.39)
Year 2	10	405	0.26 (0.22, 0.31)
Year 3	10	341	0.21 (0.17, 0.25)
Year 4	10	243	0.16 (0.14, 0.2)
Year 5	10	222	0.13 (0.11, 0.16)
Year 6	10	225	0.1 (0.08, 0.12)
Year 7	7	154	0.08 (0.07, 0.1)
Year 8	4	50	0.06 (0.05, 0.08)
Year 9	1	42	0.05 (0.04, 0.06)
PCV10			
No PCV7 impact			
Year 0	--	--	--
Year 1	4	150	0.69 (0.45, 1.06)
Year 2	4	141	0.5 (0.33, 0.77)
Year 3	4	100	0.39 (0.25, 0.59)
Year 4	2	52	0.32 (0.21, 0.48)
Year 5	2	45	0.27 (0.17, 0.41)
Year 6	2	28	0.23 (0.15, 0.35)
Year 7	2	35	0.2 (0.13, 0.3)
Substantial PCV7 impact			
Year −1	3	76	0.59 (0.42, 0.83)
Year 0	3	62	0.52 (0.37, 0.73)
Year 1	3	92	0.43 (0.31, 0.61)
Year 2	1	51	0.34 (0.24, 0.49)
Year 3	1	26	0.27 (0.19, 0.38)
Year 4	1	22	0.2 (0.14, 0.28)
Year 5	1	20	0.15 (0.1, 0.21)
Year 6	1	13	0.1 (0.07, 0.15)
Year 7	1	13	0.07 (0.05, 0.11)

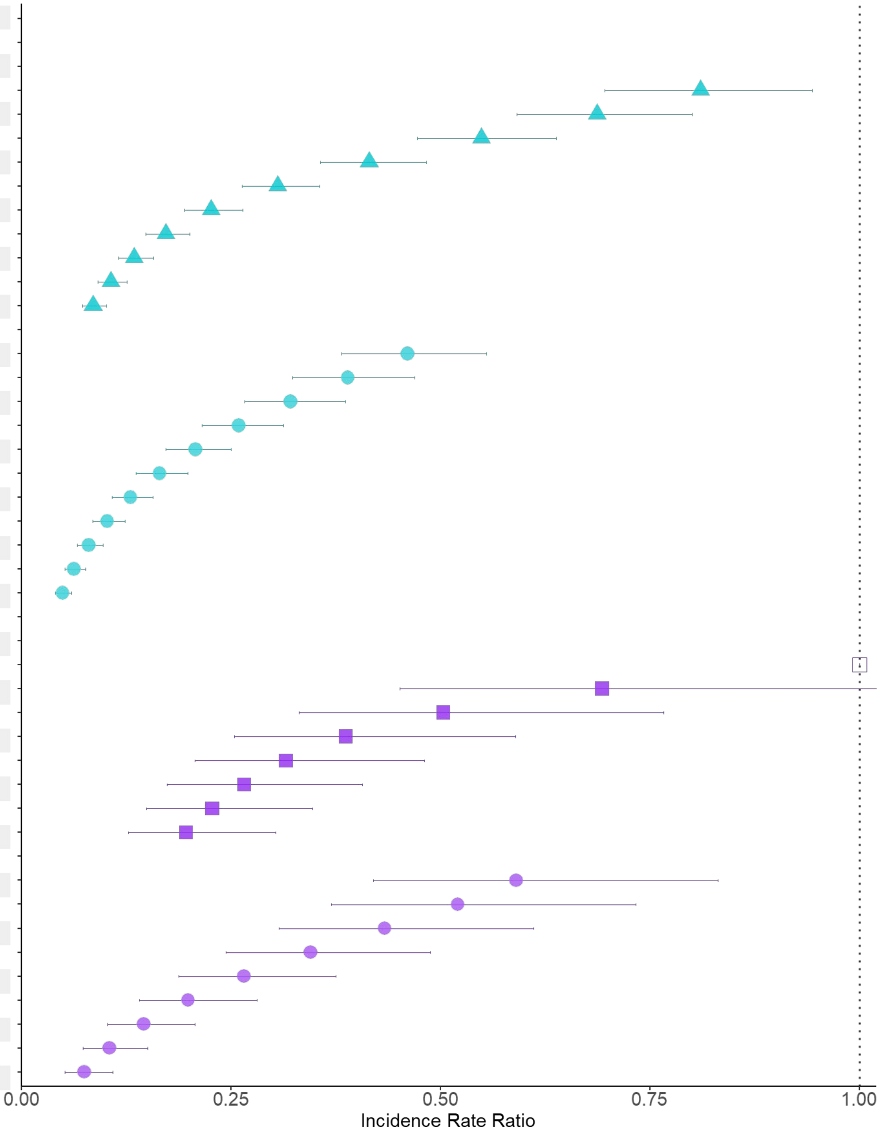

Supplementary Figure 3.37 All PCV10 ST IPD all-site weighted average IRRs comparing the annual post-PCV10/13 IR to the average pre-PCV IR for **adults aged ≥65 years**

PCV Product and PCV7 Impact Strata	Number of Sites	Number of Cases	IRR (95%CI)
PCV13			
Moderate PCV7 impact			
Year −1	5	692	0.71 (0.56, 0.9)
Year 0	5	680	0.59 (0.47, 0.75)
Year 1	5	489	0.48 (0.38, 0.61)
Year 2	5	621	0.37 (0.29, 0.47)
Year 3	5	503	0.28 (0.22, 0.36)
Year 4	5	373	0.21 (0.17, 0.27)
Year 5	5	300	0.16 (0.13, 0.2)
Year 6	5	218	0.12 (0.1, 0.15)
Year 7	4	172	0.09 (0.07, 0.12)
Year 8	2	124	0.07 (0.05, 0.09)
Substantial PCV7 impact			
Year −1	10	1369	0.3 (0.25, 0.37)
Year 0	10	989	0.24 (0.2, 0.29)
Year 1	10	694	0.19 (0.16, 0.23)
Year 2	10	500	0.15 (0.13, 0.19)
Year 3	10	393	0.12 (0.1, 0.15)
Year 4	10	260	0.1 (0.08, 0.12)
Year 5	10	267	0.08 (0.07, 0.1)
Year 6	10	268	0.07 (0.05, 0.08)
Year 7	7	173	0.05 (0.04, 0.07)
Year 8	4	104	0.05 (0.04, 0.06)
Year 9	1	77	0.04 (0.03, 0.05)
PCV10			
No PCV7 impact			
Year 0	--	--	--
Year 1	4	215	0.76 (0.55, 1.07)
Year 2	4	229	0.63 (0.45, 0.88)
Year 3	4	167	0.51 (0.37, 0.72)
Year 4	3	97	0.41 (0.29, 0.57)
Year 5	3	77	0.3 (0.22, 0.42)
Year 6	2	58	0.22 (0.16, 0.3)
Year 7	2	46	0.15 (0.11, 0.21)
Substantial PCV7 impact			
Year −1	3	152	0.44 (0.31, 0.64)
Year 0	3	110	0.38 (0.26, 0.54)
Year 1	3	122	0.32 (0.22, 0.45)
Year 2	1	75	0.26 (0.18, 0.38)
Year 3	1	64	0.21 (0.15, 0.31)
Year 4	1	73	0.17 (0.12, 0.24)
Year 5	1	48	0.13 (0.09, 0.19)
Year 6	1	38	0.1 (0.07, 0.15)
Year 7	1	25	0.08 (0.06, 0.12)

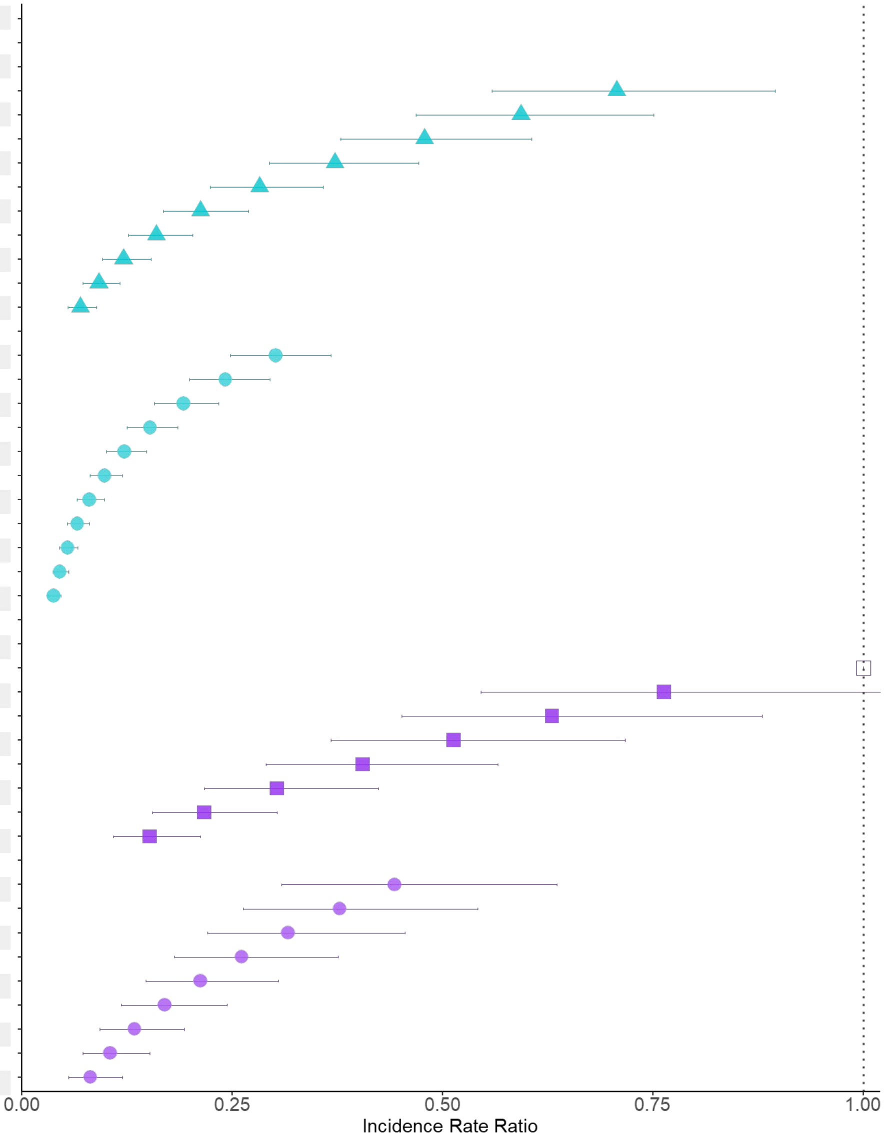

Supplementary Figure 3.38 ST6A IPD all-site weighted average IRRs comparing the annual post-PCV10/13 IR to the average pre-PCV IR for **children aged <5 years**

PCV Product and PCV7 Impact Strata	Number of Sites	Number of Cases	IRR (95%CI)
PCV13			
Moderate PCV7 impact			
Year −1	6	150	0.5 (0.28, 0.9)
Year 0	6	111	0.29 (0.16, 0.53)
Year 1	6	59	0.17 (0.09, 0.3)
Year 2	6	27	0.09 (0.05, 0.17)
Year 3	6	20	0.06 (0.03, 0.1)
Year 4	6	8	0.03 (0.02, 0.06)
Year 5	6	3	0.02 (0.01, 0.04)
Year 6	6	5	0.01 (0.01, 0.02)
Year 7	5	2	0.01 (0.01, 0.02)
Year 8	3	2	0.01 (0, 0.01)
Substantial PCV7 impact			
Year −1	11	21	0.09 (0.06, 0.14)
Year 0	11	14	0.06 (0.04, 0.09)
Year 1	11	6	0.04 (0.02, 0.06)
Year 2	11	4	0.02 (0.02, 0.04)
Year 3	11	3	0.02 (0.01, 0.03)
Year 4	11	1	0.01 (0.01, 0.02)
Year 5	11	0	0.01 (0, 0.01)
Year 6	11	2	0 (0, 0.01)
Year 7	7	1	0 (0, 0.01)
Year 8	4	0	0 (0, 0)
Year 9	1	0	0 (0, 0)
PCV10			
No PCV7 impact			
Year −1	--	--	--
Year 0	4	9	0.48 (0.31, 0.75)
Year 1	4	6	0.37 (0.24, 0.57)
Year 2	4	6	0.28 (0.18, 0.43)
Year 3	4	4	0.21 (0.14, 0.32)
Year 4	3	4	0.17 (0.11, 0.26)
Year 5	3	3	0.16 (0.1, 0.24)
Year 6	2	0	0.17 (0.11, 0.26)
Year 7	2	0	0.18 (0.12, 0.29)
Moderate PCV7 impact			
Year −1	2	15	0.43 (0.16, 1.21)
Year 0	2	8	0.39 (0.14, 1.09)
Year 1	2	16	0.3 (0.11, 0.83)
Year 2	2	8	0.19 (0.07, 0.52)
Year 3	1	5	0.1 (0.03, 0.27)
Year 4	1	2	0.04 (0.01, 0.11)
Substantial PCV7 impact			
Year −1	4	0	0.22 (0.11, 0.45)
Year 0	4	2	0.17 (0.08, 0.36)
Year 1	4	2	0.13 (0.06, 0.26)
Year 2	1	0	0.08 (0.04, 0.18)
Year 3	1	0	0.05 (0.02, 0.11)
Year 4	1	0	0.03 (0.01, 0.06)
Year 5	1	0	0.01 (0.01, 0.03)
Year 6	1	0	0.01 (0, 0.02)
Year 7	1	0	0 (0, 0.01)

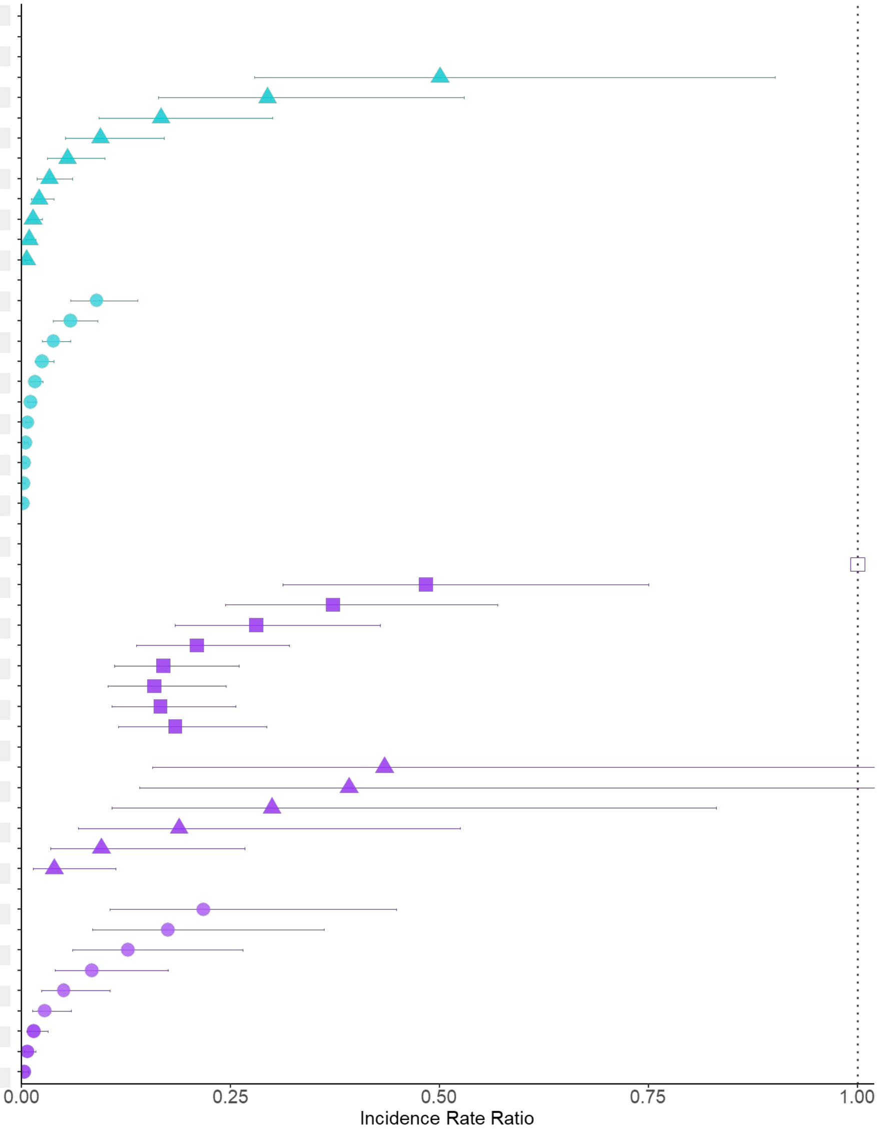

Supplementary Figure 3.39 ST6A IPD all-site weighted average IRRs comparing the annual post-PCV10/13 IR to the average pre-PCV IR for children aged 5–17 years

PCV Product and PCV7 Impact Strata	Number of Sites	Number of Cases	IRR (95%CI)
PCV13			
Moderate PCV7 impact			
Year −1	5	25	0.66 (0.42, 1.02)
Year 0	6	26	0.46 (0.3, 0.72)
Year 1	6	11	0.33 (0.21, 0.51)
Year 2	6	3	0.23 (0.15, 0.36)
Year 3	6	10	0.16 (0.1, 0.25)
Year 4	6	6	0.12 (0.07, 0.18)
Year 5	6	3	0.08 (0.05, 0.13)
Year 6	6	1	0.06 (0.04, 0.09)
Year 7	5	3	0.04 (0.03, 0.07)
Year 8	3	0	0.03 (0.02, 0.05)
Substantial PCV7 impact			
Year −1	9	7	0.24 (0.17, 0.35)
Year 0	9	8	0.18 (0.12, 0.25)
Year 1	9	3	0.12 (0.09, 0.18)
Year 2	9	3	0.09 (0.06, 0.12)
Year 3	9	0	0.06 (0.04, 0.08)
Year 4	9	0	0.04 (0.03, 0.05)
Year 5	9	0	0.02 (0.02, 0.03)
Year 6	9	0	0.01 (0.01, 0.02)
Year 7	6	0	0.01 (0.01, 0.01)
Year 8	3	0	0 (0, 0.01)
Year 9	1	0	0 (0, 0)
PCV10			
No PCV7 impact			
Year 0	--	--	--
Year 1	4	1	1.11 (0.25, 4.9)
Year 2	4	1	1.25 (0.29, 5.5)
Year 3	4	3	1.25 (0.29, 5.5)
Year 4	3	1	1.11 (0.25, 4.88)
Year 5	3	0	0.97 (0.22, 4.28)
Year 6	2	0	0.87 (0.2, 3.82)
Year 7	2	2	0.79 (0.18, 3.47)
Moderate PCV7 impact			
Year −1	2	1	1.6 (0.69, 3.69)
Year 0	2	1	0.88 (0.39, 1.99)
Year 1	2	2	0.64 (0.29, 1.42)
Year 2	2	0	0.53 (0.24, 1.16)
Year 3	1	1	0.4 (0.17, 0.97)
Year 4	1	0	0.24 (0.07, 0.86)
Substantial PCV7 impact			
Year −1	1	0	1.62 (0.16, 15.87)
Year 0	1	0	1.14 (0.1, 13.61)
Year 1	1	0	0.48 (0, 111.17)

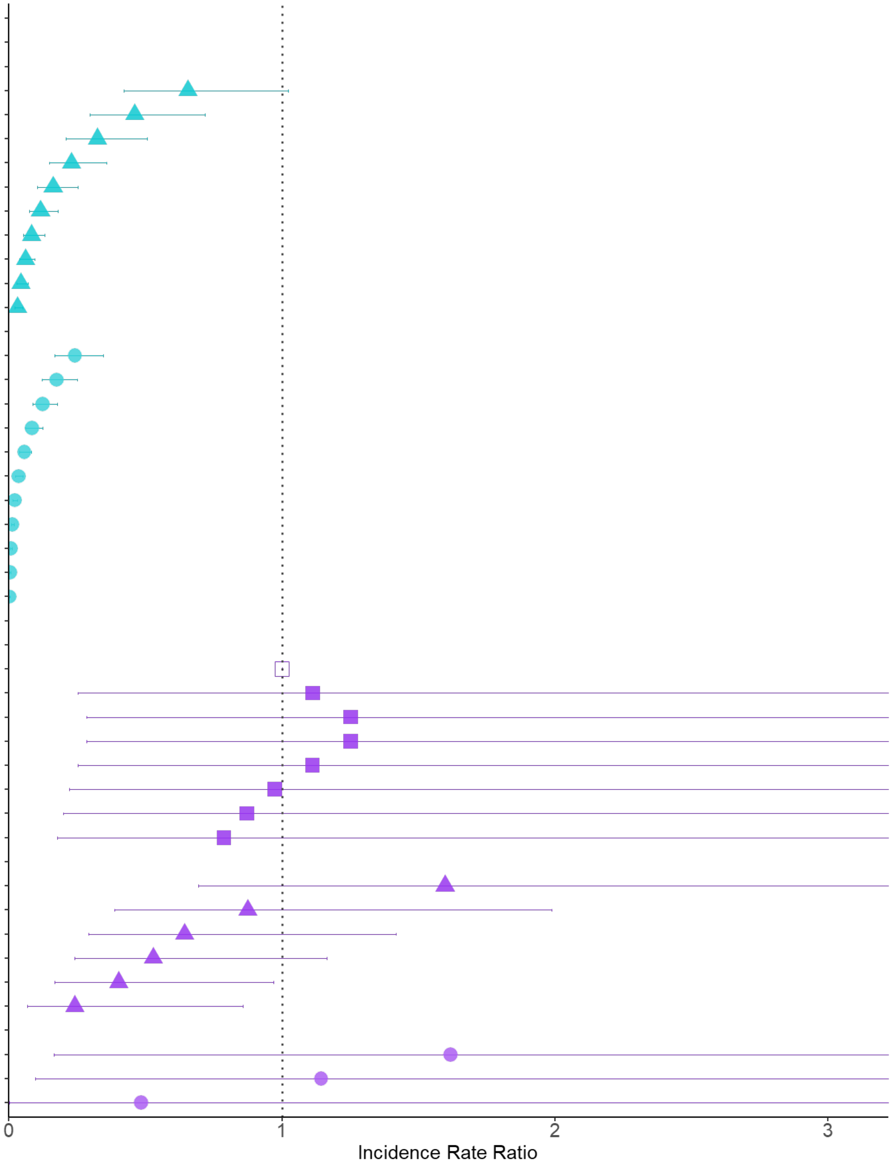

Supplementary Figure 3.40 ST6A IPD all-site weighted average IRRs comparing the annual post-PCV10/13 IR to the average pre-PCV IR for **adults aged 18–49 years**

PCV Product and PCV7 Impact Strata	Number of Sites	Number of Cases	IRR (95%CI)
PCV13			
Moderate PCV7 impact			
Year −1	4	103	0.96 (0.54, 1.71)
Year 0	4	107	0.75 (0.42, 1.33)
Year 1	4	68	0.53 (0.3, 0.93)
Year 2	4	38	0.34 (0.19, 0.61)
Year 3	4	31	0.23 (0.13, 0.4)
Year 4	4	19	0.16 (0.09, 0.29)
Year 5	4	19	0.13 (0.07, 0.23)
Year 6	4	20	0.12 (0.07, 0.21)
Year 7	3	12	0.12 (0.07, 0.21)
Year 8	2	4	0.12 (0.07, 0.21)
Substantial PCV7 impact			
Year −1	10	49	0.38 (0.25, 0.57)
Year 0	10	39	0.23 (0.15, 0.35)
Year 1	10	10	0.14 (0.09, 0.21)
Year 2	10	5	0.09 (0.06, 0.13)
Year 3	10	1	0.06 (0.04, 0.08)
Year 4	10	1	0.04 (0.03, 0.06)
Year 5	10	3	0.03 (0.02, 0.04)
Year 6	10	2	0.02 (0.01, 0.03)
Year 7	7	1	0.02 (0.01, 0.03)
Year 8	4	1	0.01 (0.01, 0.02)
Year 9	1	0	0.01 (0.01, 0.02)
PCV10			
No PCV7 impact			
Year 0	--	--	--
Year 1	3	6	1.33 (0.57, 3.08)
Year 2	3	4	0.99 (0.43, 2.3)
Year 3	3	3	0.69 (0.3, 1.6)
Year 4	2	0	0.48 (0.21, 1.12)
Year 5	2	2	0.42 (0.18, 0.98)
Year 6	2	1	0.51 (0.22, 1.19)
Year 7	2	3	0.73 (0.32, 1.71)
Substantial PCV7 impact			
Year −1	3	2	0.43 (0.2, 0.89)
Year 0	3	0	0.32 (0.15, 0.67)
Year 1	3	0	0.24 (0.11, 0.51)
Year 2	1	1	0.19 (0.09, 0.41)
Year 3	1	0	0.18 (0.08, 0.37)
Year 4	1	0	0.18 (0.09, 0.39)
Year 5	1	0	0.22 (0.1, 0.46)
Year 6	1	1	0.28 (0.13, 0.61)
Year 7	1	0	0.39 (0.17, 0.88)

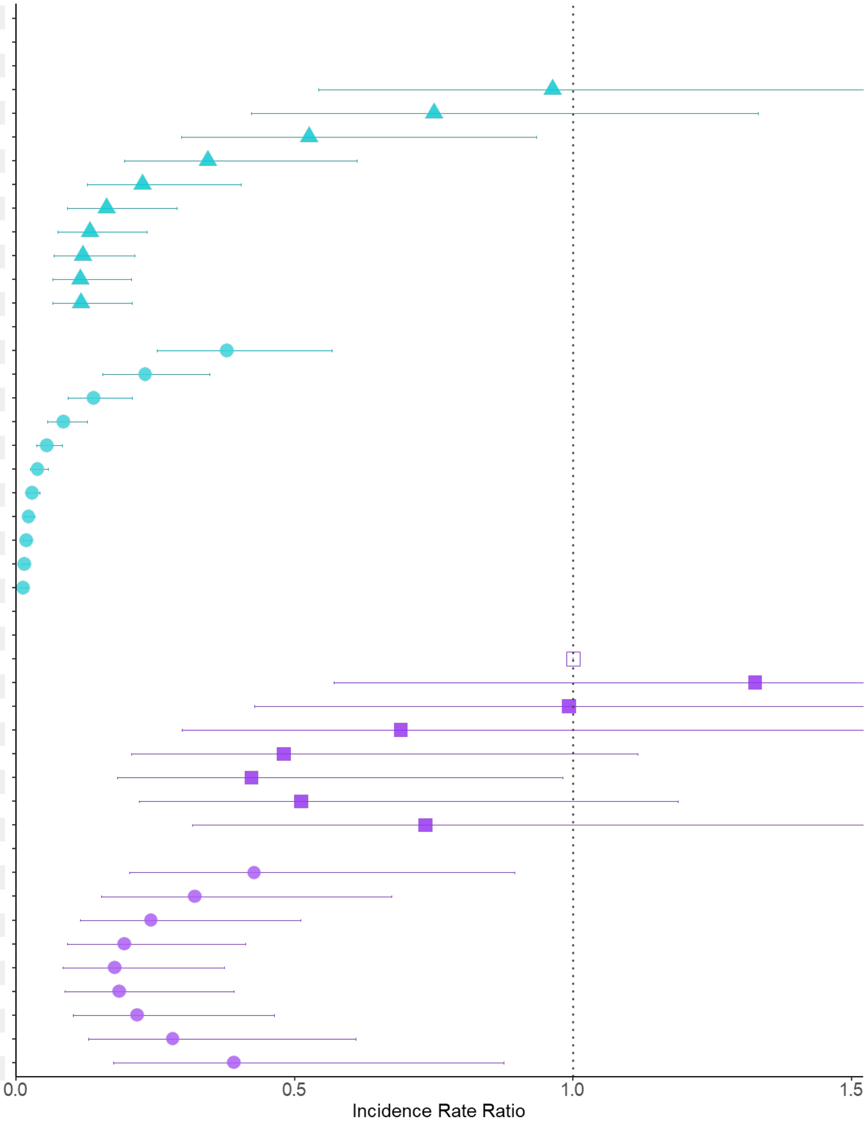

Supplementary Figure 3.41 ST6A IPD all-site weighted average IRRs comparing the annual post-PCV10/13 IR to the average pre-PCV IR for **adults aged 50–64 years**

PCV Product and PCV7 Impact Strata	Number of Sites	Number of Cases	IRR (95%CI)
PCV13			
Moderate PCV7 impact			
Year −1	4	37	1.02 (0.46, 2.27)
Year 0	4	45	0.86 (0.39, 1.9)
Year 1	4	26	0.65 (0.3, 1.45)
Year 2	4	21	0.47 (0.21, 1.03)
Year 3	4	16	0.33 (0.15, 0.73)
Year 4	4	13	0.25 (0.11, 0.54)
Year 5	4	13	0.2 (0.09, 0.44)
Year 6	4	11	0.17 (0.08, 0.38)
Year 7	3	7	0.16 (0.07, 0.35)
Year 8	2	4	0.15 (0.07, 0.33)
Substantial PCV7 impact			
Year −1	10	61	0.36 (0.23, 0.55)
Year 0	10	61	0.26 (0.17, 0.39)
Year 1	10	13	0.18 (0.12, 0.28)
Year 2	10	13	0.13 (0.09, 0.2)
Year 3	10	10	0.1 (0.06, 0.15)
Year 4	10	5	0.07 (0.05, 0.11)
Year 5	10	5	0.06 (0.04, 0.09)
Year 6	10	8	0.05 (0.03, 0.07)
Year 7	7	4	0.04 (0.03, 0.06)
Year 8	4	1	0.03 (0.02, 0.05)
Year 9	1	1	0.03 (0.02, 0.04)
PCV10			
No PCV7 impact			
Year 0	--	--	--
Year 1	4	6	0.52 (0.32, 0.85)
Year 2	4	6	0.52 (0.32, 0.84)
Year 3	4	7	0.49 (0.3, 0.8)
Year 4	2	5	0.45 (0.28, 0.73)
Year 5	2	3	0.4 (0.25, 0.65)
Year 6	2	2	0.36 (0.22, 0.59)
Year 7	2	4	0.32 (0.2, 0.52)
Substantial PCV7 impact			
Year −1	3	1	0.42 (0.19, 0.91)
Year 0	3	3	0.25 (0.11, 0.54)
Year 1	3	2	0.15 (0.07, 0.32)
Year 2	1	0	0.1 (0.04, 0.22)
Year 3	1	0	0.08 (0.04, 0.18)
Year 4	1	0	0.09 (0.04, 0.19)
Year 5	1	0	0.11 (0.05, 0.25)
Year 6	1	1	0.18 (0.08, 0.4)
Year 7	1	1	0.32 (0.14, 0.73)

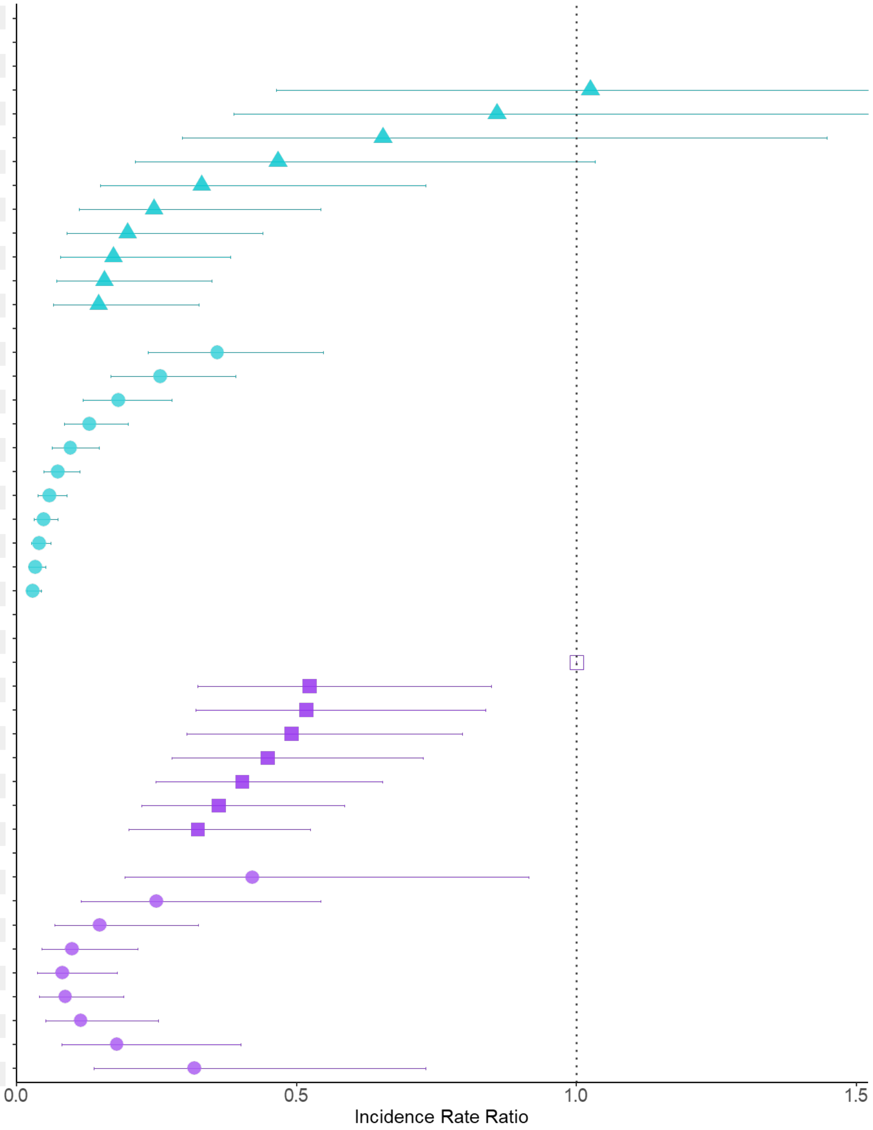

Supplementary Figure 3.42 ST6A IPD all-site weighted average IRRs comparing the annual post-PCV10/13 IR to the average pre-PCV IR for adults aged ≥65 years

PCV Product and PCV7 Impact Strata	Number of Sites	Number of Cases	IRR (95%CI)
PCV13			
Moderate PCV7 impact			
Year −1	4	48	0.69 (0.31, 1.53)
Year 0	4	52	0.54 (0.24, 1.21)
Year 1	4	38	0.4 (0.18, 0.89)
Year 2	4	39	0.29 (0.13, 0.64)
Year 3	4	41	0.2 (0.09, 0.46)
Year 4	4	32	0.15 (0.07, 0.34)
Year 5	4	28	0.12 (0.05, 0.27)
Year 6	4	21	0.1 (0.04, 0.22)
Year 7	3	23	0.09 (0.04, 0.19)
Year 8	2	9	0.08 (0.03, 0.17)
Substantial PCV7 impact			
Year −1	10	222	0.41 (0.29, 0.59)
Year 0	10	155	0.28 (0.2, 0.4)
Year 1	10	65	0.19 (0.13, 0.27)
Year 2	10	36	0.13 (0.09, 0.18)
Year 3	10	19	0.09 (0.06, 0.13)
Year 4	10	18	0.06 (0.05, 0.09)
Year 5	10	14	0.05 (0.03, 0.07)
Year 6	10	7	0.04 (0.03, 0.05)
Year 7	7	10	0.03 (0.02, 0.04)
Year 8	4	5	0.02 (0.02, 0.03)
Year 9	1	5	0.02 (0.01, 0.03)
PCV10			
No PCV7 impact			
Year 0	--	--	--
Year 1	4	18	0.8 (0.46, 1.4)
Year 2	4	12	0.69 (0.4, 1.19)
Year 3	4	17	0.63 (0.37, 1.09)
Year 4	3	19	0.6 (0.35, 1.03)
Year 5	3	12	0.54 (0.31, 0.93)
Year 6	2	10	0.46 (0.27, 0.79)
Year 7	2	10	0.38 (0.22, 0.66)
Substantial PCV7 impact			
Year −1	3	9	0.45 (0.24, 0.86)
Year 0	3	7	0.3 (0.16, 0.58)
Year 1	3	9	0.21 (0.11, 0.4)
Year 2	1	2	0.15 (0.08, 0.28)
Year 3	1	0	0.11 (0.06, 0.21)
Year 4	1	2	0.09 (0.05, 0.17)
Year 5	1	0	0.08 (0.04, 0.15)
Year 6	1	2	0.07 (0.03, 0.13)
Year 7	1	3	0.06 (0.03, 0.13)

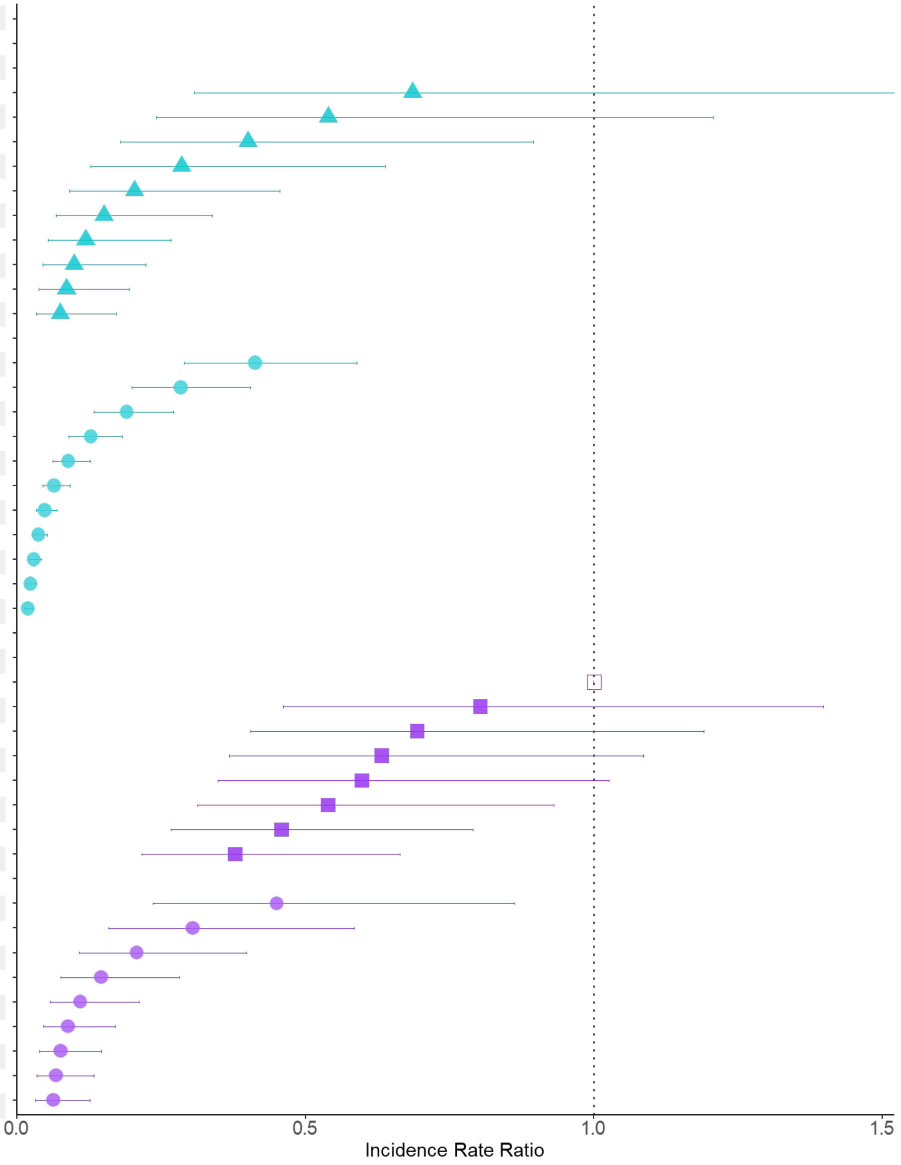

Supplementary Figure 3.43 ST19A IPD all-site weighted average IRRs comparing the annual post-PCV10/13 IR to the average pre-PCV IR for children aged <5 years

PCV Product and PCV7 Impact Strata	Number of Sites	Number of Cases	IRR (95%CI)
PCV13			
Moderate PCV7 impact			
Year −1	6	228	1.56 (0.99, 2.46)
Year 0	6	243	1.16 (0.74, 1.83)
Year 1	6	212	0.81 (0.51, 1.27)
Year 2	6	138	0.55 (0.35, 0.87)
Year 3	6	54	0.4 (0.25, 0.63)
Year 4	6	57	0.3 (0.19, 0.48)
Year 5	6	29	0.25 (0.16, 0.39)
Year 6	6	35	0.21 (0.13, 0.33)
Year 7	5	30	0.19 (0.12, 0.29)
Year 8	3	26	0.17 (0.1, 0.26)
Substantial PCV7 impact			
Year −1	11	347	2.49 (1.78, 3.49)
Year 0	11	422	1.83 (1.31, 2.57)
Year 1	11	318	1.27 (0.91, 1.79)
Year 2	11	118	0.89 (0.63, 1.24)
Year 3	11	76	0.66 (0.47, 0.92)
Year 4	11	57	0.52 (0.37, 0.73)
Year 5	11	54	0.44 (0.32, 0.62)
Year 6	11	44	0.39 (0.28, 0.55)
Year 7	7	47	0.36 (0.26, 0.51)
Year 8	4	23	0.35 (0.24, 0.49)
Year 9	1	10	0.33 (0.23, 0.47)
PCV10			
No PCV7 impact			
Year −1	--	--	--
Year 0	4	17	0.44 (0.33, 0.57)
Year 1	4	7	0.6 (0.46, 0.78)
Year 2	4	13	0.8 (0.62, 1.05)
Year 3	4	5	1.02 (0.78, 1.33)
Year 4	3	21	1.23 (0.94, 1.6)
Year 5	3	8	1.41 (1.08, 1.83)
Year 6	2	15	1.55 (1.19, 2.03)
Year 7	2	15	1.69 (1.28, 2.21)
Moderate PCV7 impact			
Year −1	2	29	1.16 (0.53, 2.57)
Year 0	2	22	1.26 (0.57, 2.77)
Year 1	2	24	1.35 (0.61, 2.98)
Year 2	2	27	1.57 (0.71, 3.48)
Year 3	1	26	2.14 (0.96, 4.76)
Year 4	1	34	3.45 (1.47, 8.08)
Substantial PCV7 impact			
Year −1	4	2	1.58 (0.9, 2.77)
Year 0	4	10	1.67 (0.95, 2.94)
Year 1	4	38	1.76 (0.99, 3.11)
Year 2	1	35	1.85 (1.04, 3.29)
Year 3	1	0	1.95 (1.09, 3.48)
Year 4	1	1	2.06 (1.15, 3.68)
Year 5	1	3	2.18 (1.21, 3.94)
Year 6	1	2	2.31 (1.25, 4.29)
Year 7	1	4	2.46 (1.26, 4.79)

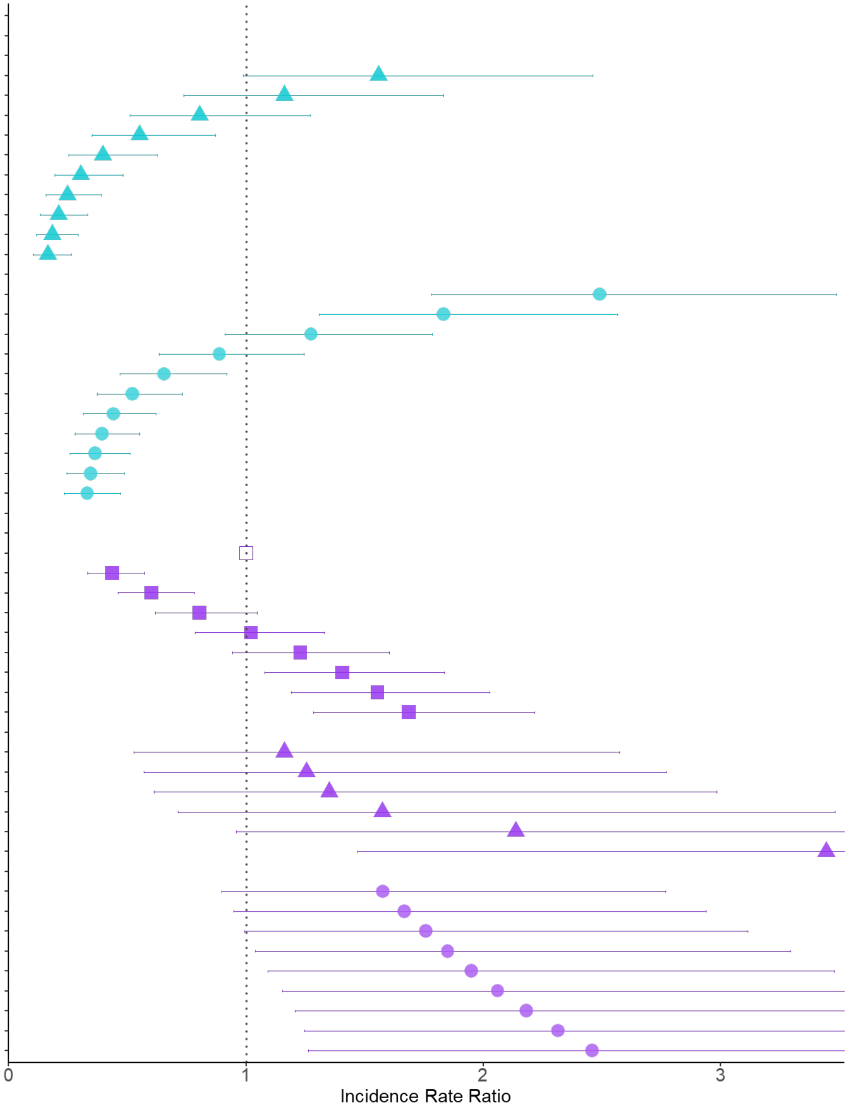

Supplementary Figure 3.44 ST19A IPD all-site weighted average IRRs comparing the annual post-PCV10/13 IR to the average pre-PCV IR for children aged 5–17 years

PCV Product and PCV7 Impact Strata	Number of Sites	Number of Cases	IRR (95%CI)
PCV13			
Moderate PCV7 impact			
Year −1	5	33	1.91 (1.32, 2.75)
Year 0	6	22	1.71 (1.18, 2.46)
Year 1	6	30	1.4 (0.97, 2.02)
Year 2	6	25	1.11 (0.77, 1.6)
Year 3	6	9	0.89 (0.62, 1.29)
Year 4	6	10	0.76 (0.53, 1.09)
Year 5	6	6	0.68 (0.47, 0.98)
Year 6	6	8	0.63 (0.44, 0.91)
Year 7	5	9	0.61 (0.42, 0.88)
Year 8	3	4	0.6 (0.41, 0.88)
Substantial PCV7 impact			
Year −1	10	39	3.29 (2.48, 4.37)
Year 0	10	53	2.86 (2.16, 3.79)
Year 1	10	46	2.31 (1.74, 3.06)
Year 2	10	24	1.81 (1.36, 2.4)
Year 3	10	14	1.44 (1.09, 1.92)
Year 4	10	13	1.22 (0.92, 1.62)
Year 5	10	18	1.09 (0.82, 1.45)
Year 6	10	16	1.02 (0.77, 1.36)
Year 7	7	15	0.99 (0.74, 1.32)
Year 8	4	6	0.98 (0.73, 1.32)
Year 9	1	4	0.98 (0.72, 1.33)
PCV10			
No PCV7 impact			
Year 0	--	--	--
Year 1	4	3	0.27 (0.12, 0.6)
Year 2	4	1	0.44 (0.2, 0.97)
Year 3	4	3	0.9 (0.41, 1.97)
Year 4	3	4	2.06 (0.94, 4.5)
Year 5	3	4	3.34 (1.52, 7.32)
Year 6	2	6	3.67 (1.68, 8.05)
Year 7	2	3	3.32 (1.51, 7.31)
Moderate PCV7 impact			
Year −1	2	2	0.54 (0.28, 1.03)
Year 0	2	2	0.68 (0.35, 1.3)
Year 1	2	3	0.75 (0.4, 1.43)
Year 2	2	4	0.95 (0.5, 1.8)
Year 3	1	1	1.76 (0.9, 3.44)
Year 4	1	6	5.78 (2.45, 13.65)
Substantial PCV7 impact			
Year −1	3	0	2.07 (1.23, 3.49)
Year 0	3	1	2 (1.19, 3.37)
Year 1	3	2	1.87 (1.1, 3.17)
Year 2	1	0	1.79 (1.05, 3.08)
Year 3	1	0	1.89 (1.1, 3.24)
Year 4	1	0	2.27 (1.32, 3.9)
Year 5	1	0	3.09 (1.78, 5.35)
Year 6	1	1	4.65 (2.61, 8.28)
Year 7	1	1	7.53 (4, 14.17)

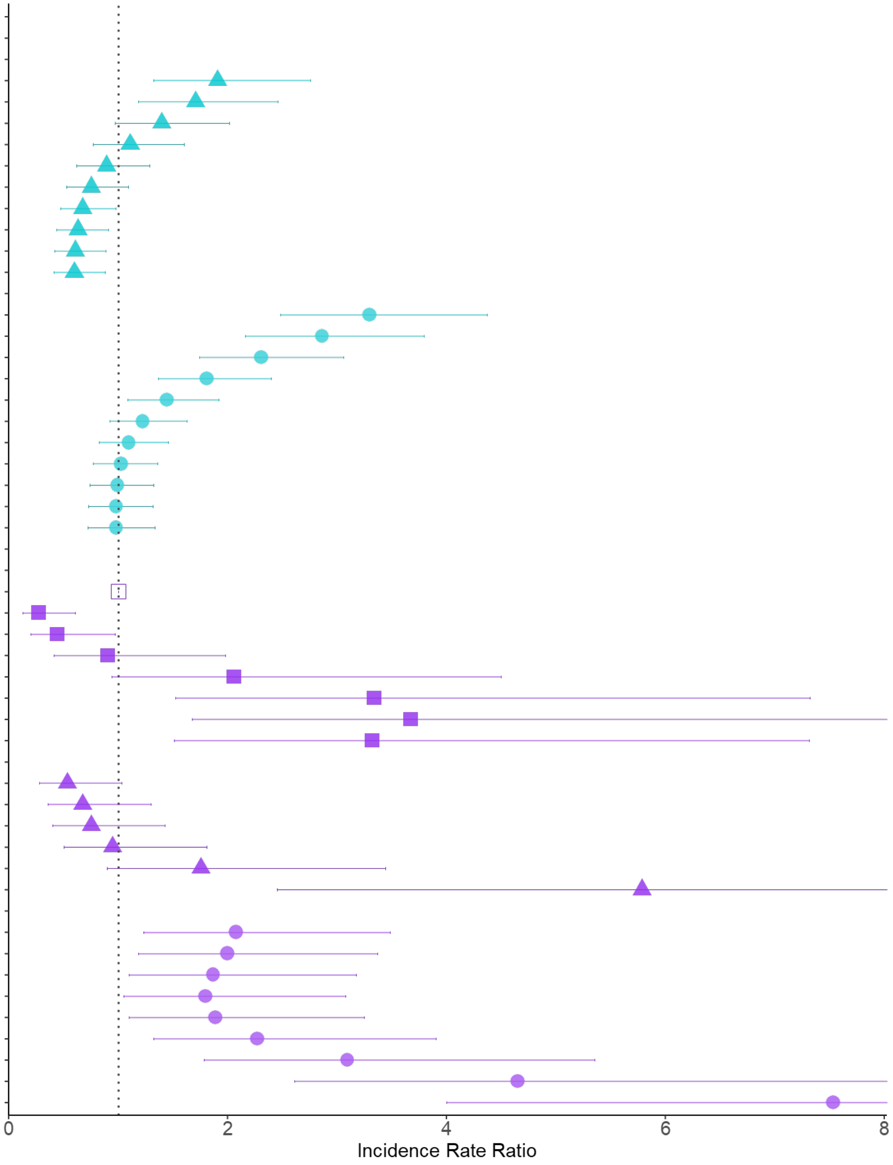

Supplementary Figure 3.45 ST19A IPD all-site weighted average IRRs comparing the annual post-PCV10/13 IR to the average pre-PCV IR for adults aged 18–49 years

PCV Product and PCV7 Impact Strata	Number of Sites	Number of Cases	IRR (95%CI)
PCV13			
Moderate PCV7 impact			
Year −1	4	200	1.83 (1.14, 2.95)
Year 0	4	208	1.76 (1.09, 2.83)
Year 1	4	149	1.54 (0.96, 2.48)
Year 2	4	173	1.26 (0.78, 2.02)
Year 3	4	123	1.01 (0.63, 1.63)
Year 4	4	122	0.85 (0.53, 1.37)
Year 5	4	103	0.77 (0.48, 1.24)
Year 6	4	119	0.75 (0.46, 1.2)
Year 7	3	91	0.76 (0.47, 1.22)
Year 8	2	25	0.78 (0.48, 1.26)
Substantial PCV7 impact			
Year −1	10	314	3.51 (2.44, 5.04)
Year 0	10	335	2.97 (2.07, 4.26)
Year 1	10	300	2.41 (1.68, 3.47)
Year 2	10	149	1.94 (1.35, 2.79)
Year 3	10	140	1.58 (1.1, 2.27)
Year 4	10	94	1.33 (0.92, 1.9)
Year 5	10	122	1.14 (0.79, 1.63)
Year 6	10	103	0.99 (0.69, 1.43)
Year 7	7	68	0.88 (0.61, 1.27)
Year 8	4	51	0.78 (0.54, 1.14)
Year 9	1	30	0.7 (0.48, 1.02)
PCV10			
No PCV7 impact			
Year 0	--	--	--
Year 1	5	5	1.04 (0.62, 1.73)
Year 2	5	10	1.54 (0.92, 2.57)
Year 3	5	12	2.22 (1.33, 3.71)
Year 4	4	25	3.01 (1.81, 5.02)
Year 5	4	30	3.62 (2.17, 6.05)
Year 6	3	23	3.89 (2.33, 6.49)
Year 7	3	39	3.95 (2.35, 6.62)
Substantial PCV7 impact			
Year −1	3	6	3.71 (1.91, 7.21)
Year 0	3	5	3.96 (2.03, 7.7)
Year 1	3	22	4.19 (2.14, 8.2)
Year 2	1	6	4.38 (2.23, 8.61)
Year 3	1	2	4.47 (2.27, 8.79)
Year 4	1	7	4.43 (2.25, 8.73)
Year 5	1	5	4.3 (2.16, 8.53)
Year 6	1	3	4.09 (2.02, 8.28)
Year 7	1	8	3.84 (1.82, 8.08)

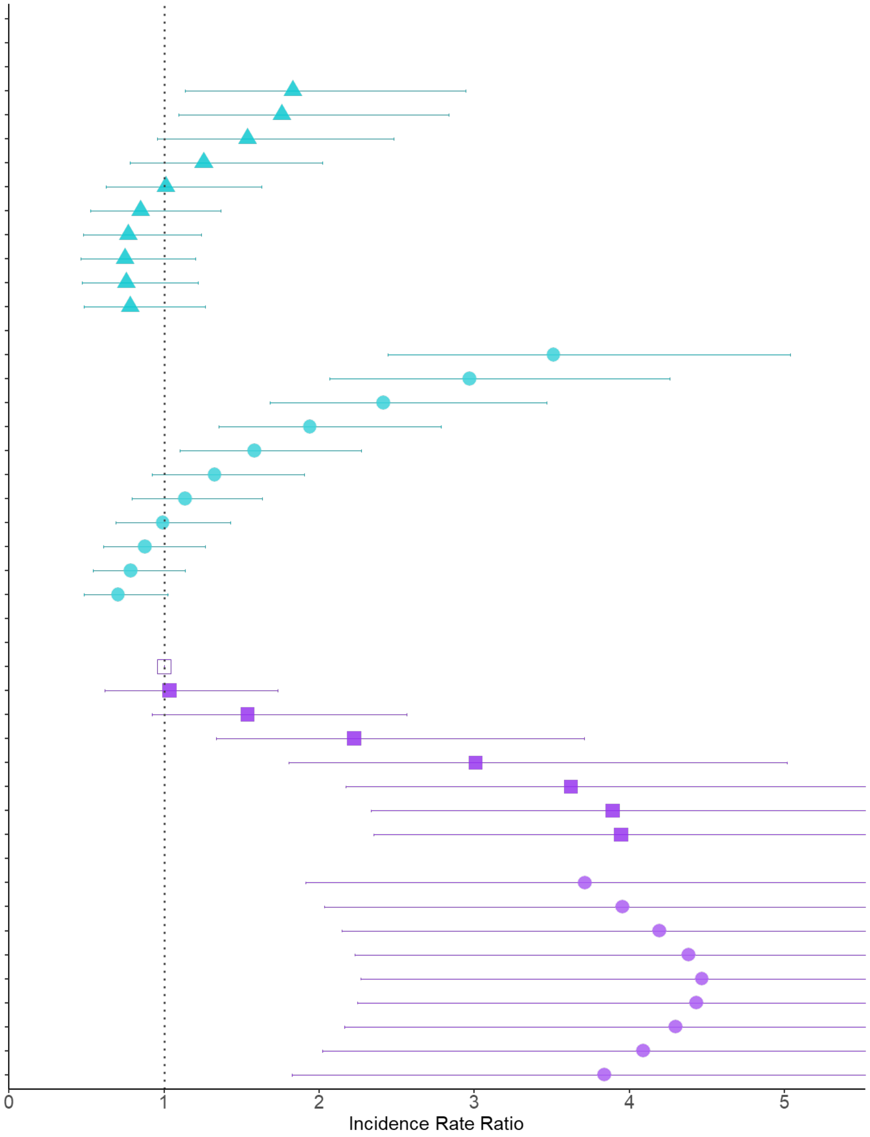

Supplementary Figure 3.46 ST19A IPD all-site weighted average IRRs comparing the annual post-PCV10/13 IR to the average pre-PCV IR for adults aged 50–64 years

PCV Product and PCV7 Impact Strata	Number of Sites	Number of Cases	IRR (95%CI)
PCV13			
Moderate PCV7 impact			
Year −1	4	78	2.06 (1.38, 3.07)
Year 0	4	90	2.03 (1.36, 3.02)
Year 1	4	93	1.83 (1.23, 2.72)
Year 2	4	107	1.53 (1.03, 2.28)
Year 3	4	100	1.24 (0.83, 1.84)
Year 4	4	98	0.99 (0.67, 1.48)
Year 5	4	78	0.82 (0.55, 1.22)
Year 6	4	74	0.69 (0.46, 1.02)
Year 7	3	70	0.58 (0.39, 0.87)
Year 8	2	39	0.5 (0.33, 0.75)
Substantial PCV7 impact			
Year −1	10	352	3.73 (2.58, 5.39)
Year 0	10	357	3.35 (2.32, 4.85)
Year 1	10	342	2.89 (2, 4.18)
Year 2	10	212	2.45 (1.7, 3.55)
Year 3	10	200	2.1 (1.45, 3.04)
Year 4	10	142	1.83 (1.27, 2.65)
Year 5	10	143	1.63 (1.12, 2.35)
Year 6	10	146	1.46 (1.01, 2.12)
Year 7	7	109	1.33 (0.92, 1.92)
Year 8	4	64	1.21 (0.83, 1.76)
Year 9	1	51	1.11 (0.76, 1.62)
PCV10			
No PCV7 impact			
Year 0	--	--	--
Year 1	4	6	0.27 (0.15, 0.48)
Year 2	4	11	0.46 (0.26, 0.82)
Year 3	4	21	0.88 (0.5, 1.58)
Year 4	2	21	1.66 (0.93, 2.97)
Year 5	2	36	2.36 (1.32, 4.22)
Year 6	2	39	2.52 (1.41, 4.5)
Year 7	2	37	2.33 (1.3, 4.17)
Substantial PCV7 impact			
Year −1	3	5	4.46 (2.27, 8.78)
Year 0	3	15	5.41 (2.75, 10.65)
Year 1	3	38	6.24 (3.16, 12.29)
Year 2	1	16	6.93 (3.51, 13.68)
Year 3	1	16	7.53 (3.81, 14.86)
Year 4	1	22	8.06 (4.08, 15.91)
Year 5	1	22	8.52 (4.31, 16.84)
Year 6	1	28	8.92 (4.49, 17.74)
Year 7	1	33	9.27 (4.61, 18.65)

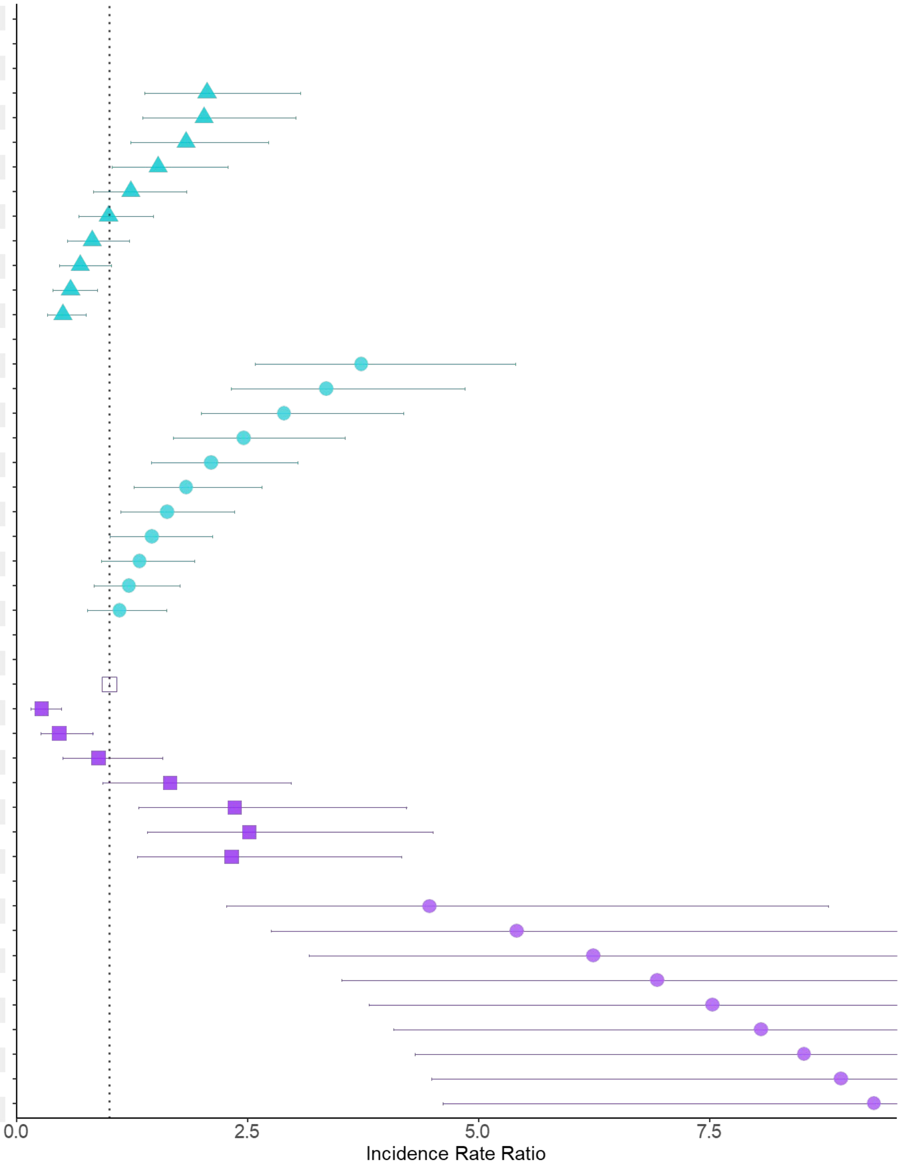

Supplementary Figure 3.47 ST19A IPD all-site weighted average IRRs comparing the annual post-PCV10/13 IR to the average pre-PCV IR for **adults aged ≥65 years**

PCV Product and PCV7 Impact Strata	Number of Sites	Number of Cases	IRR (95%CI)
PCV13			
Moderate PCV7 impact			
Year −1	4	105	2.23 (1.73, 2.89)
Year 0	4	179	2.27 (1.75, 2.93)
Year 1	4	204	2.05 (1.59, 2.65)
Year 2	4	285	1.7 (1.31, 2.19)
Year 3	4	239	1.34 (1.04, 1.73)
Year 4	4	202	1.06 (0.82, 1.37)
Year 5	4	180	0.87 (0.67, 1.13)
Year 6	4	192	0.74 (0.57, 0.95)
Year 7	3	166	0.64 (0.49, 0.83)
Year 8	2	128	0.56 (0.42, 0.73)
Substantial PCV7 impact			
Year −1	10	472	3.09 (2.53, 3.77)
Year 0	10	579	2.63 (2.15, 3.21)
Year 1	10	521	2.16 (1.77, 2.64)
Year 2	10	396	1.76 (1.44, 2.15)
Year 3	10	253	1.45 (1.19, 1.77)
Year 4	10	228	1.23 (1, 1.5)
Year 5	10	233	1.06 (0.87, 1.3)
Year 6	10	253	0.93 (0.76, 1.14)
Year 7	7	197	0.83 (0.68, 1.02)
Year 8	4	151	0.74 (0.6, 0.92)
Year 9	1	103	0.67 (0.54, 0.84)
PCV10			
No PCV7 impact			
Year 0	--	--	--
Year 1	3	29	1.25 (0.71, 2.17)
Year 2	3	37	1.48 (0.85, 2.57)
Year 3	3	48	2.01 (1.16, 3.48)
Year 4	2	43	3.04 (1.75, 5.28)
Year 5	2	63	4.22 (2.43, 7.34)
Year 6	2	85	4.9 (2.82, 8.5)
Year 7	2	100	5.15 (2.95, 8.99)
Substantial PCV7 impact			
Year −1	3	19	2.52 (1.74, 3.64)
Year 0	3	36	2.79 (1.93, 4.05)
Year 1	3	80	3.09 (2.12, 4.49)
Year 2	1	43	3.35 (2.29, 4.9)
Year 3	1	28	3.54 (2.42, 5.19)
Year 4	1	52	3.64 (2.48, 5.32)
Year 5	1	51	3.63 (2.47, 5.34)
Year 6	1	49	3.56 (2.39, 5.3)
Year 7	1	57	3.43 (2.23, 5.26)

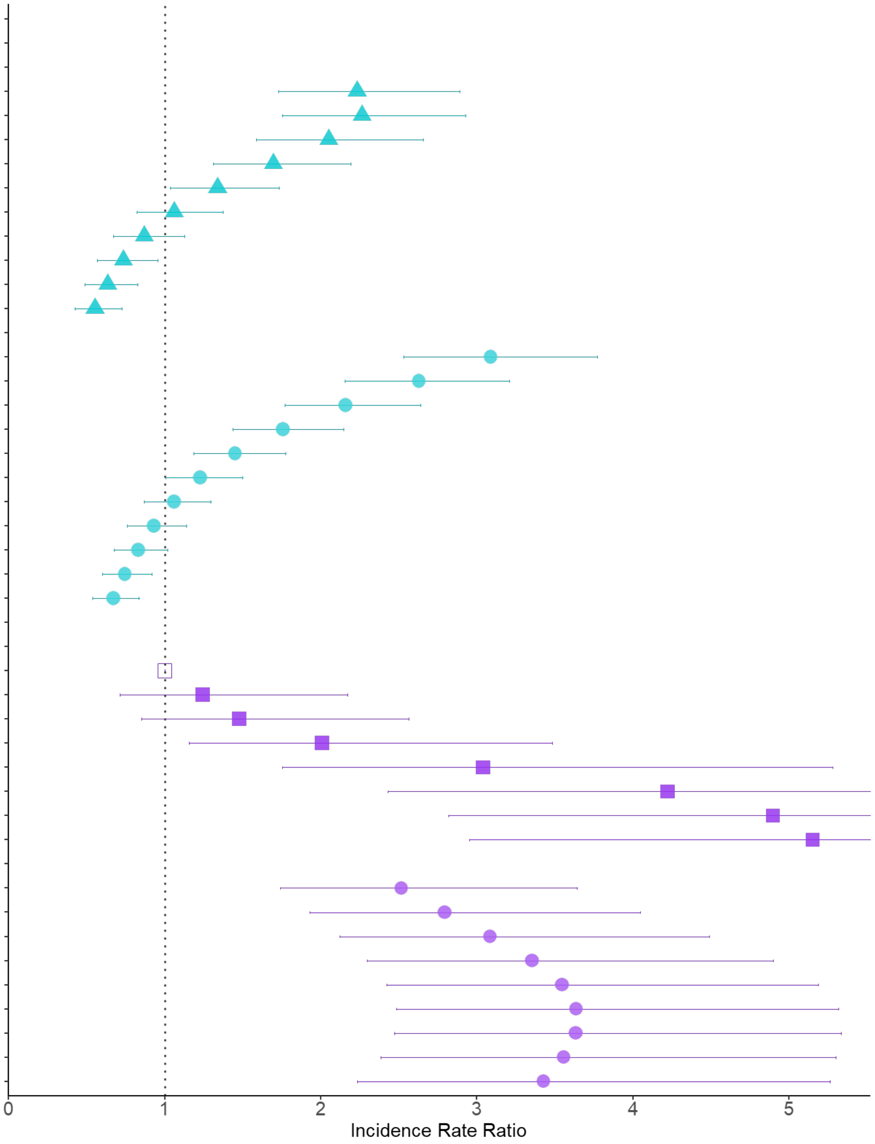

Supplementary Figure 3.48 ST3 IPD all-site weighted average IRRs comparing the annual post-PCV10/13 IR to the average pre-PCV IR for children aged <5 years

PCV Product and PCV7 Impact Strata	Number of Sites	Number of Cases	IRR (95%CI)
PCV13			
Moderate PCV7 impact			
Year −1	6	41	1.35 (0.97, 1.89)
Year 0	6	56	1.19 (0.85, 1.66)
Year 1	6	52	1.03 (0.74, 1.44)
Year 2	6	32	0.91 (0.65, 1.27)
Year 3	6	40	0.85 (0.61, 1.19)
Year 4	6	25	0.86 (0.61, 1.2)
Year 5	6	32	0.92 (0.66, 1.28)
Year 6	6	23	1.04 (0.74, 1.45)
Year 7	5	39	1.22 (0.87, 1.7)
Year 8	3	45	1.46 (1.04, 2.05)
Substantial PCV7 impact			
Year −1	11	55	1.32 (1.03, 1.69)
Year 0	11	54	1.26 (0.98, 1.61)
Year 1	11	54	1.19 (0.93, 1.53)
Year 2	11	54	1.14 (0.89, 1.46)
Year 3	11	32	1.12 (0.88, 1.44)
Year 4	11	54	1.14 (0.89, 1.46)
Year 5	11	38	1.19 (0.93, 1.52)
Year 6	11	63	1.26 (0.98, 1.62)
Year 7	7	83	1.36 (1.06, 1.75)
Year 8	4	36	1.49 (1.16, 1.92)
Year 9	1	23	1.64 (1.27, 2.11)
PCV10			
No PCV7 impact			
Year −1	--	--	--
Year 0	4	2	0.91 (0.49, 1.72)
Year 1	4	2	1.29 (0.69, 2.43)
Year 2	4	4	1.68 (0.89, 3.14)
Year 3	4	5	1.89 (1.01, 3.54)
Year 4	3	5	1.92 (1.02, 3.6)
Year 5	3	6	1.84 (0.98, 3.44)
Year 6	2	1	1.69 (0.9, 3.17)
Year 7	2	4	1.52 (0.81, 2.87)
Moderate PCV7 impact			
Year −1	2	8	1.55 (0.86, 2.79)
Year 0	2	4	1.77 (0.99, 3.18)
Year 1	2	1	2.19 (1.22, 3.93)
Year 2	2	6	2.76 (1.53, 4.96)
Year 3	1	9	3.37 (1.86, 6.09)
Year 4	1	7	3.86 (2.01, 7.4)
Substantial PCV7 impact			
Year −1	2	0	1.96 (1.09, 3.54)
Year 0	2	1	2.1 (1.17, 3.77)
Year 1	2	7	2.21 (1.22, 3.99)
Year 2	1	3	2.25 (1.23, 4.1)
Year 3	1	1	2.16 (1.17, 3.97)
Year 4	1	1	1.94 (1.05, 3.57)
Year 5	1	2	1.65 (0.88, 3.07)
Year 6	1	1	1.34 (0.69, 2.6)
Year 7	1	1	1.05 (0.49, 2.22)

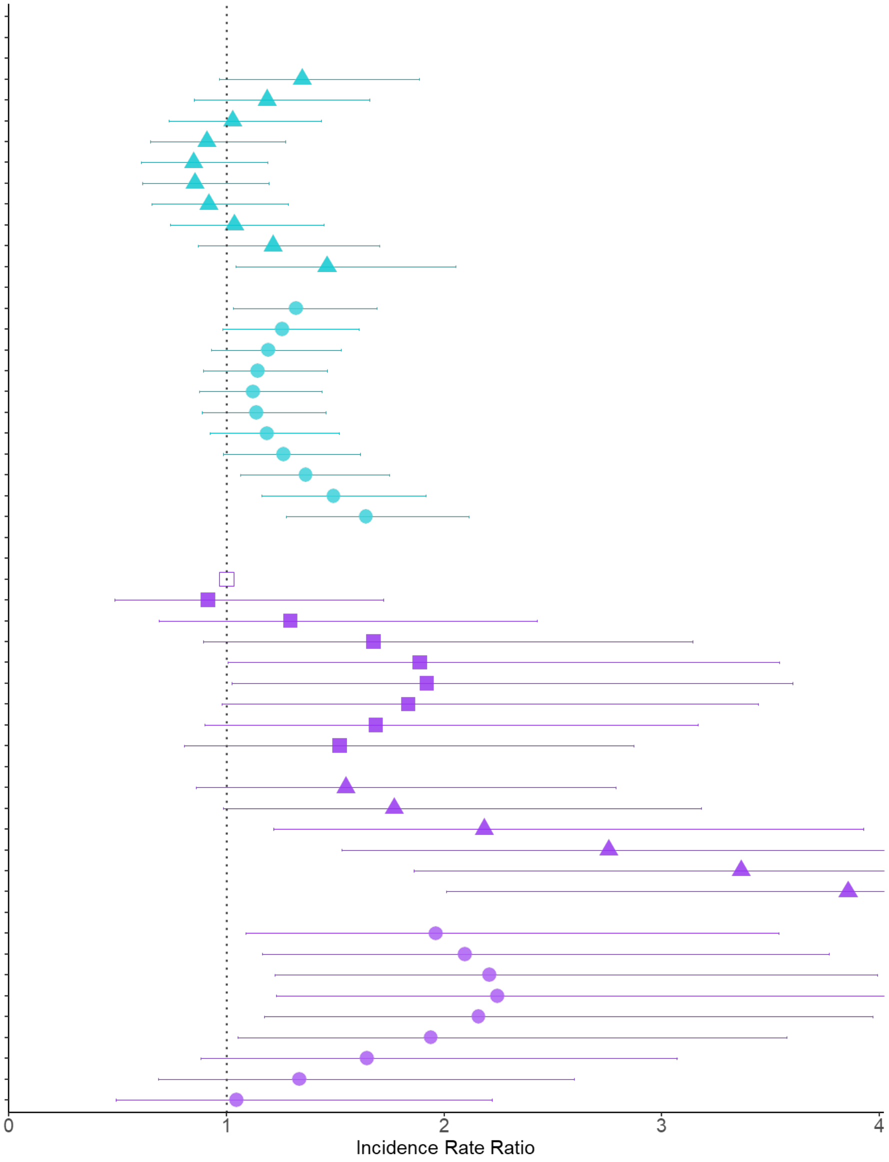

Supplementary Figure 3.49 ST3 IPD all-site weighted average IRRs comparing the annual post-PCV10/13 IR to the average pre-PCV IR for children aged 5–17 years

PCV Product and PCV7 Impact Strata	Number of Sites	Number of Cases	IRR (95%CI)
PCV13			
Moderate PCV7 impact			
Year −1	5	12	1.01 (0.71, 1.44)
Year 0	6	10	1.02 (0.72, 1.45)
Year 1	6	18	1.04 (0.73, 1.47)
Year 2	6	20	1.05 (0.74, 1.49)
Year 3	6	12	1.05 (0.74, 1.49)
Year 4	6	13	1.03 (0.73, 1.46)
Year 5	6	12	0.99 (0.7, 1.41)
Year 6	6	13	0.94 (0.66, 1.34)
Year 7	5	10	0.89 (0.62, 1.26)
Year 8	3	6	0.83 (0.58, 1.19)
Substantial PCV7 impact			
Year −1	10	13	1.23 (0.94, 1.62)
Year 0	10	18	1.31 (1, 1.72)
Year 1	10	22	1.41 (1.07, 1.85)
Year 2	10	20	1.5 (1.14, 1.97)
Year 3	10	12	1.57 (1.19, 2.05)
Year 4	10	16	1.59 (1.21, 2.09)
Year 5	10	22	1.58 (1.2, 2.07)
Year 6	10	36	1.53 (1.16, 2.01)
Year 7	7	14	1.46 (1.11, 1.92)
Year 8	4	3	1.38 (1.04, 1.82)
Year 9	1	4	1.3 (0.98, 1.72)
PCV10			
No PCV7 impact			
Year 0	--	--	--
Year 1	3	3	1 (0.69, 1.43)
Year 2	3	3	1.09 (0.84, 1.43)
Year 3	3	1	1.27 (0.97, 1.67)
Year 4	3	5	1.65 (1.27, 2.13)
Year 5	3	3	2.38 (1.8, 3.14)
Year 6	2	5	3.78 (2.89, 4.96)
Year 7	2	5	6.31 (4.33, 9.19)
Moderate PCV7 impact			
Year −1	2	0	1.32 (0.71, 2.46)
Year 0	2	2	1.51 (0.81, 2.79)
Year 1	2	1	1.4 (0.76, 2.58)
Year 2	2	2	1.23 (0.67, 2.28)
Year 3	1	0	1.21 (0.64, 2.29)
Year 4	1	1	1.49 (0.67, 3.28)
Substantial PCV7 impact			
Year −1	3	1	3.19 (1.94, 5.24)
Year 0	3	1	2.71 (1.65, 4.46)
Year 1	3	1	2.19 (1.33, 3.61)
Year 2	1	1	1.76 (1.06, 2.92)
Year 3	1	0	1.48 (0.89, 2.46)
Year 4	1	0	1.36 (0.82, 2.25)
Year 5	1	0	1.35 (0.81, 2.25)
Year 6	1	1	1.43 (0.85, 2.4)
Year 7	1	0	1.58 (0.92, 2.74)

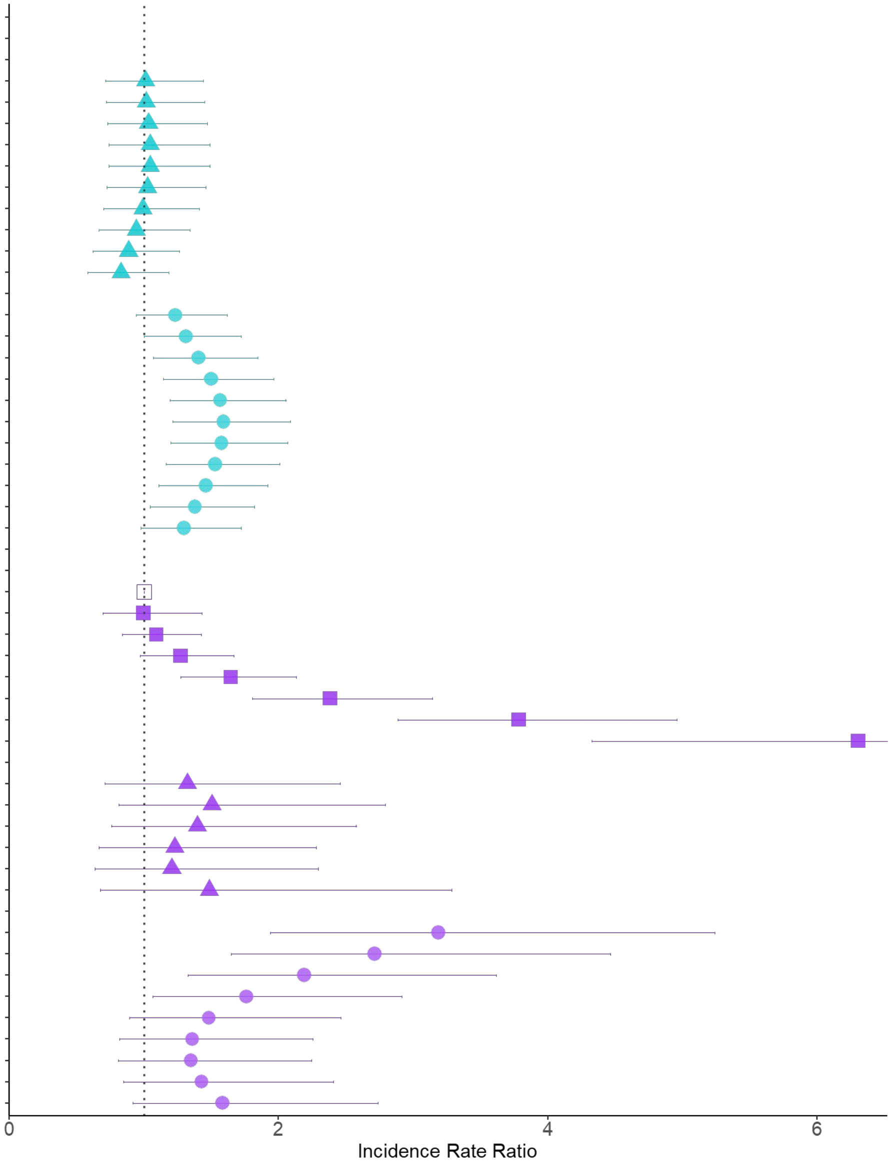

Supplementary Figure 3.50 ST3 IPD all-site weighted average IRRs comparing the annual post-PCV10/13 IR to the average pre-PCV IR for adults aged 18–49 years

PCV Product and PCV7 Impact Strata	Number of Sites	Number of Cases	IRR (95%CI)
PCV13			
Moderate PCV7 impact			
Year −1	4	99	1.21 (1, 1.45)
Year 0	4	89	1.15 (0.96, 1.38)
Year 1	4	97	1.07 (0.89, 1.28)
Year 2	4	129	0.98 (0.81, 1.17)
Year 3	4	116	0.9 (0.75, 1.08)
Year 4	4	115	0.87 (0.72, 1.04)
Year 5	4	126	0.87 (0.73, 1.05)
Year 6	4	140	0.92 (0.76, 1.1)
Year 7	3	132	0.99 (0.82, 1.19)
Year 8	2	75	1.09 (0.9, 1.31)
Substantial PCV7 impact			
Year −1	10	187	1.22 (1.07, 1.39)
Year 0	10	167	1.14 (1, 1.3)
Year 1	10	160	1.06 (0.93, 1.2)
Year 2	10	141	1 (0.87, 1.13)
Year 3	10	135	0.97 (0.85, 1.1)
Year 4	10	113	0.98 (0.86, 1.11)
Year 5	10	149	1.02 (0.89, 1.16)
Year 6	10	196	1.09 (0.96, 1.24)
Year 7	7	143	1.19 (1.04, 1.35)
Year 8	4	73	1.31 (1.15, 1.5)
Year 9	1	70	1.46 (1.28, 1.67)
PCV10			
No PCV7 impact			
Year 0	--	--	--
Year 1	5	19	0.44 (0.24, 0.81)
Year 2	5	16	0.45 (0.25, 0.82)
Year 3	5	20	0.51 (0.28, 0.94)
Year 4	4	27	0.66 (0.36, 1.19)
Year 5	4	30	0.83 (0.45, 1.51)
Year 6	3	38	0.99 (0.54, 1.8)
Year 7	3	40	1.14 (0.62, 2.09)
Substantial PCV7 impact			
Year −1	3	6	0.74 (0.58, 0.94)
Year 0	3	7	0.73 (0.58, 0.93)
Year 1	3	8	0.73 (0.58, 0.93)
Year 2	1	11	0.74 (0.58, 0.94)
Year 3	1	3	0.75 (0.59, 0.96)
Year 4	1	4	0.78 (0.61, 0.99)
Year 5	1	6	0.81 (0.64, 1.04)
Year 6	1	4	0.86 (0.67, 1.1)
Year 7	1	7	0.91 (0.71, 1.18)

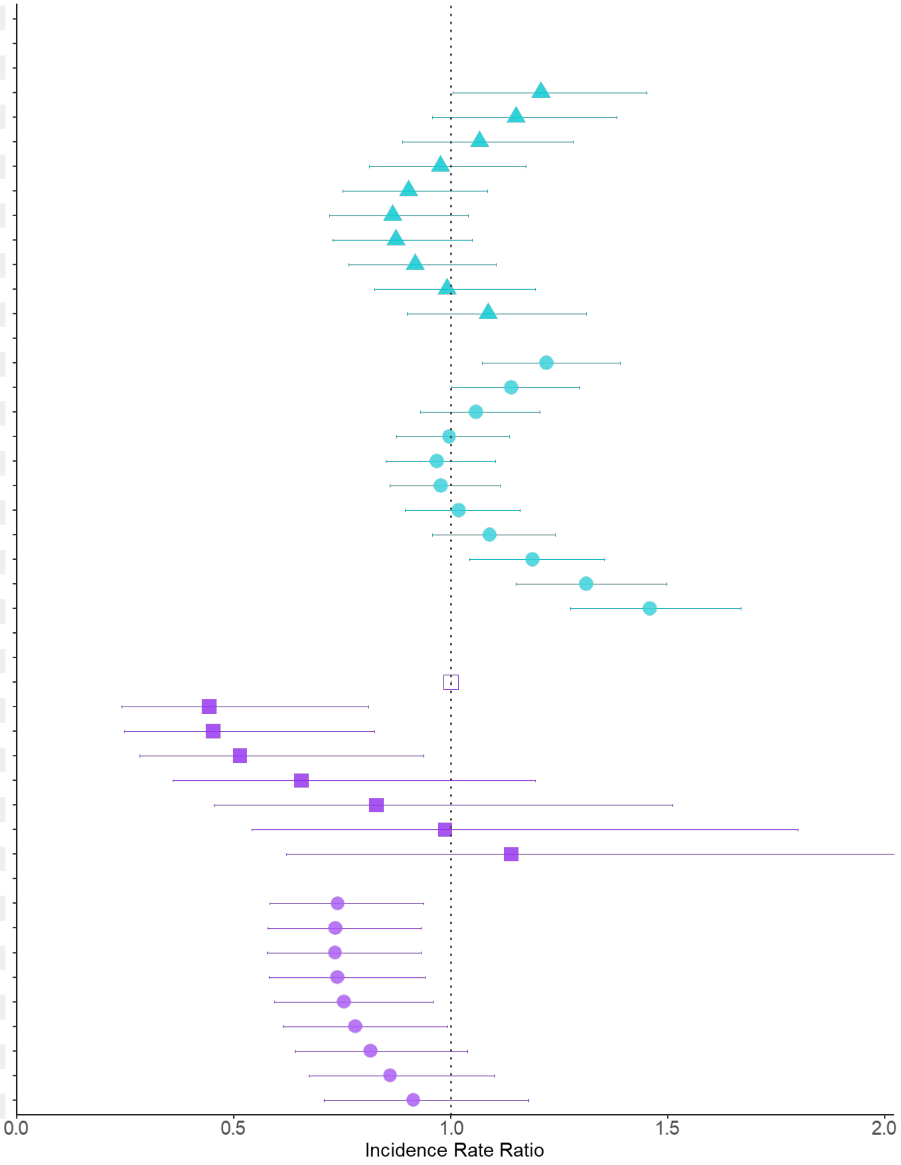

Supplementary Figure 3.51 ST3 IPD all-site weighted average IRRs comparing the annual post-PCV10/13 IR to the average pre-PCV IR for adults aged 50–64 years

PCV Product and PCV7 Impact Strata	Number of Sites	Number of Cases	IRR (95%CI)
PCV13			
Moderate PCV7 impact			
Year −1	4	68	1.24 (1.09, 1.42)
Year 0	4	99	1.22 (1.07, 1.39)
Year 1	4	92	1.17 (1.03, 1.34)
Year 2	4	142	1.12 (0.98, 1.27)
Year 3	4	163	1.07 (0.93, 1.22)
Year 4	4	159	1.04 (0.91, 1.19)
Year 5	4	205	1.05 (0.92, 1.2)
Year 6	4	186	1.1 (0.96, 1.25)
Year 7	3	200	1.16 (1.01, 1.32)
Year 8	2	208	1.24 (1.08, 1.41)
Substantial PCV7 impact			
Year −1	10	235	1.56 (1.26, 1.95)
Year 0	10	257	1.52 (1.22, 1.88)
Year 1	10	232	1.46 (1.18, 1.82)
Year 2	10	233	1.42 (1.15, 1.77)
Year 3	10	219	1.42 (1.14, 1.77)
Year 4	10	193	1.46 (1.18, 1.82)
Year 5	10	242	1.54 (1.24, 1.91)
Year 6	10	318	1.65 (1.33, 2.05)
Year 7	7	300	1.8 (1.45, 2.24)
Year 8	4	134	1.98 (1.59, 2.46)
Year 9	1	102	2.19 (1.75, 2.73)
PCV10			
No PCV7 impact			
Year 0	--	--	--
Year 1	4	28	0.54 (0.34, 0.84)
Year 2	4	37	0.56 (0.36, 0.87)
Year 3	4	43	0.7 (0.45, 1.1)
Year 4	2	23	0.99 (0.64, 1.55)
Year 5	2	37	1.25 (0.8, 1.96)
Year 6	2	46	1.36 (0.87, 2.12)
Year 7	2	38	1.37 (0.88, 2.15)
Substantial PCV7 impact			
Year −1	3	12	1.15 (0.78, 1.72)
Year 0	3	10	1.21 (0.81, 1.8)
Year 1	3	32	1.26 (0.84, 1.87)
Year 2	1	6	1.31 (0.88, 1.95)
Year 3	1	10	1.36 (0.91, 2.04)
Year 4	1	8	1.43 (0.96, 2.14)
Year 5	1	11	1.51 (1.01, 2.26)
Year 6	1	17	1.61 (1.07, 2.41)
Year 7	1	16	1.71 (1.14, 2.58)

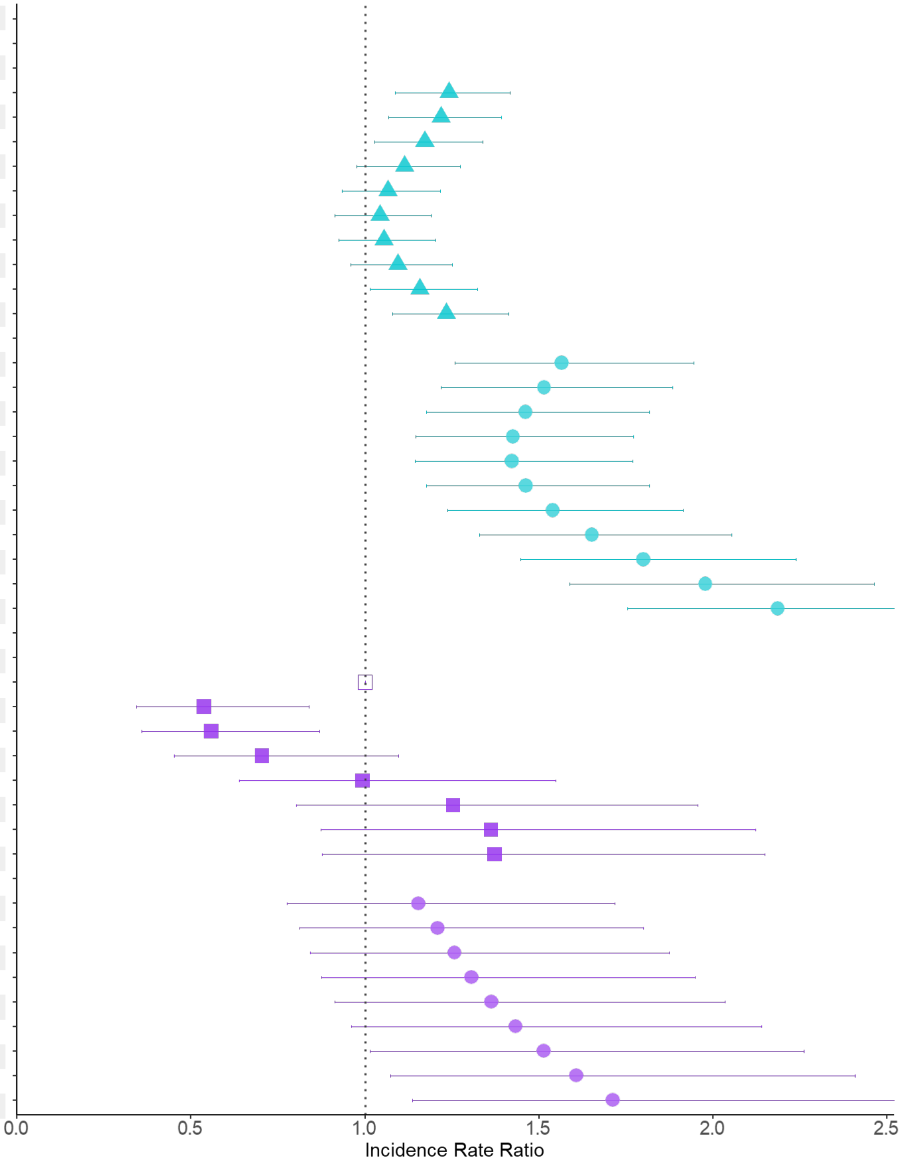

Supplementary Figure 3.52 ST3 IPD all-site weighted average IRRs comparing the annual post-PCV10/13 IR to the average pre-PCV IR for adults aged ≥65 years

PCV Product and PCV7 Impact Strata	Number of Sites	Number of Cases	IRR (95%CI)
PCV13			
Moderate PCV7 impact			
Year −1	4	173	1.11 (1.04, 1.18)
Year 0	4	191	1.09 (1.03, 1.16)
Year 1	4	231	1.06 (0.99, 1.13)
Year 2	4	360	1.02 (0.95, 1.08)
Year 3	4	390	0.98 (0.92, 1.04)
Year 4	4	365	0.95 (0.89, 1.01)
Year 5	4	554	0.95 (0.89, 1.01)
Year 6	4	544	0.96 (0.9, 1.02)
Year 7	3	559	0.98 (0.92, 1.05)
Year 8	2	492	1.01 (0.95, 1.09)
Substantial PCV7 impact			
Year −1	10	445	0.91 (0.78, 1.05)
Year 0	10	477	0.86 (0.74, 1)
Year 1	10	464	0.82 (0.71, 0.95)
Year 2	10	397	0.8 (0.69, 0.93)
Year 3	10	402	0.8 (0.69, 0.93)
Year 4	10	345	0.84 (0.72, 0.97)
Year 5	10	500	0.9 (0.78, 1.04)
Year 6	10	664	0.99 (0.85, 1.15)
Year 7	7	559	1.11 (0.95, 1.28)
Year 8	4	383	1.26 (1.08, 1.46)
Year 9	1	305	1.43 (1.23, 1.66)
PCV10			
No PCV7 impact			
Year 0	--	--	--
Year 1	3	61	0.62 (0.21, 1.86)
Year 2	3	72	0.63 (0.21, 1.89)
Year 3	3	70	0.7 (0.23, 2.07)
Year 4	2	62	0.82 (0.28, 2.44)
Year 5	2	78	0.96 (0.32, 2.87)
Year 6	2	75	1.08 (0.36, 3.21)
Year 7	2	77	1.18 (0.39, 3.5)
Substantial PCV7 impact			
Year −1	3	27	0.94 (0.71, 1.23)
Year 0	3	21	0.9 (0.69, 1.18)
Year 1	3	41	0.85 (0.65, 1.12)
Year 2	1	28	0.82 (0.62, 1.07)
Year 3	1	20	0.81 (0.62, 1.07)
Year 4	1	25	0.85 (0.65, 1.12)
Year 5	1	34	0.93 (0.71, 1.22)
Year 6	1	33	1.05 (0.8, 1.38)
Year 7	1	52	1.21 (0.92, 1.61)

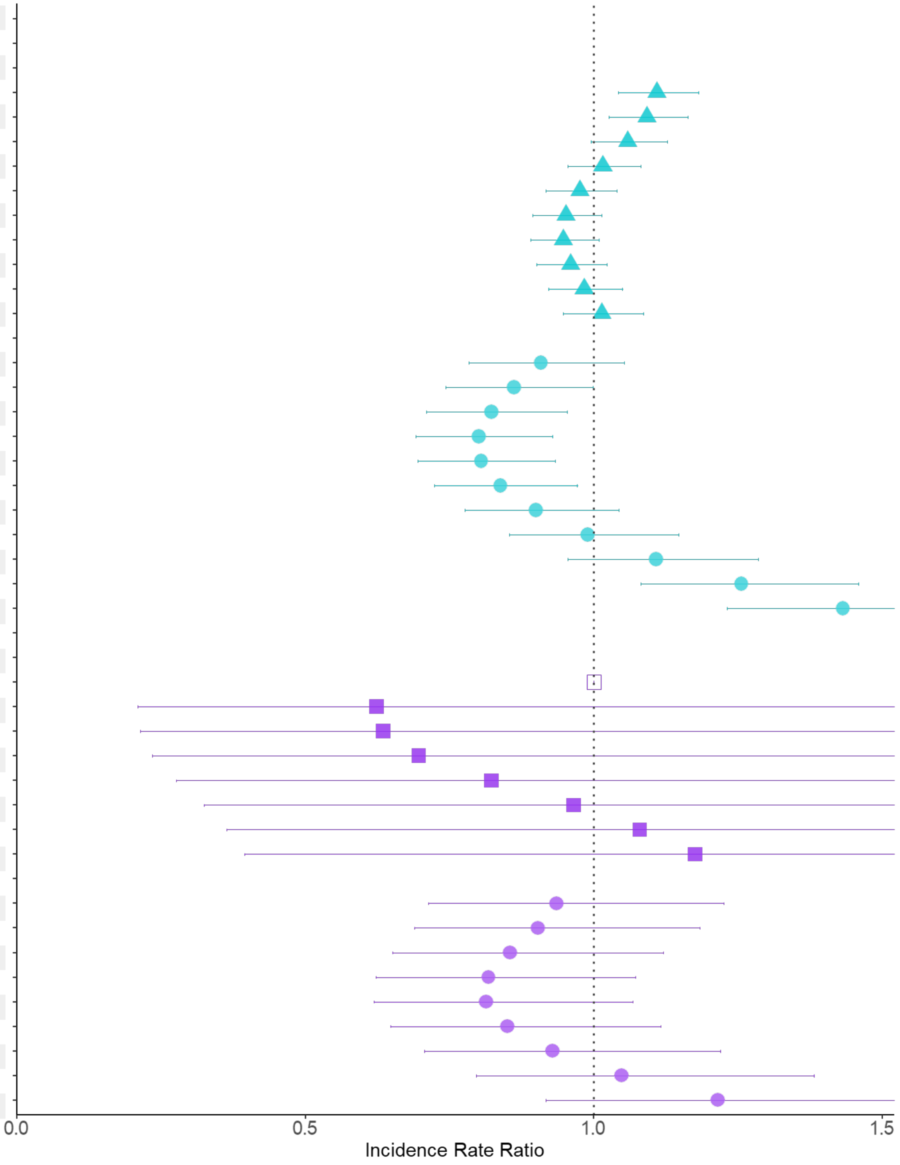

Supplementary Figure 3.53 All PCV13 ST IPD all-site weighted average IRRs comparing the annual post-PCV10/13 IR to the average pre-PCV IR for children aged <5 years

PCV Product and PCV7 Impact Strata	Number of Sites	Number of Cases	IRR (95%CI)
PCV13			
Moderate PCV7 impact			
Year −1	6	1494	0.52 (0.41, 0.65)
Year 0	6	1228	0.36 (0.29, 0.45)
Year 1	6	863	0.24 (0.19, 0.29)
Year 2	6	519	0.16 (0.12, 0.19)
Year 3	6	282	0.11 (0.09, 0.14)
Year 4	6	195	0.08 (0.07, 0.11)
Year 5	6	121	0.07 (0.06, 0.09)
Year 6	6	136	0.06 (0.05, 0.08)
Year 7	5	117	0.06 (0.05, 0.07)
Year 8	3	125	0.06 (0.04, 0.07)
Substantial PCV7 impact			
Year −1	10	750	0.23 (0.19, 0.27)
Year 0	10	816	0.17 (0.14, 0.21)
Year 1	10	636	0.13 (0.11, 0.15)
Year 2	10	310	0.09 (0.08, 0.11)
Year 3	10	178	0.07 (0.06, 0.09)
Year 4	10	173	0.06 (0.05, 0.07)
Year 5	10	135	0.05 (0.04, 0.06)
Year 6	10	151	0.05 (0.04, 0.06)
Year 7	6	176	0.04 (0.04, 0.05)
Year 8	4	73	0.04 (0.04, 0.05)
Year 9	1	45	0.04 (0.03, 0.05)
PCV10			
No PCV7 impact			
Year −1	--	--	--
Year 0	4	194	0.57 (0.32, 1.03)
Year 1	4	150	0.41 (0.23, 0.74)
Year 2	4	106	0.3 (0.17, 0.53)
Year 3	4	53	0.22 (0.12, 0.4)
Year 4	3	57	0.18 (0.1, 0.33)
Year 5	3	24	0.18 (0.1, 0.32)
Year 6	2	21	0.2 (0.11, 0.36)
Year 7	2	23	0.24 (0.13, 0.42)
Moderate PCV7 impact			
Year −1	2	263	0.54 (0.37, 0.8)
Year 0	2	195	0.4 (0.27, 0.59)
Year 1	2	155	0.3 (0.2, 0.44)
Year 2	2	105	0.23 (0.16, 0.34)
Year 3	1	85	0.18 (0.12, 0.27)
Year 4	1	58	0.15 (0.09, 0.23)
Substantial PCV7 impact			
Year −1	3	10	0.27 (0.2, 0.37)
Year 0	3	22	0.25 (0.18, 0.34)
Year 1	3	70	0.22 (0.16, 0.31)
Year 2	1	56	0.2 (0.15, 0.28)
Year 3	1	3	0.18 (0.13, 0.25)
Year 4	1	6	0.16 (0.12, 0.22)
Year 5	1	6	0.14 (0.1, 0.2)
Year 6	1	6	0.12 (0.08, 0.18)
Year 7	1	6	0.1 (0.07, 0.16)

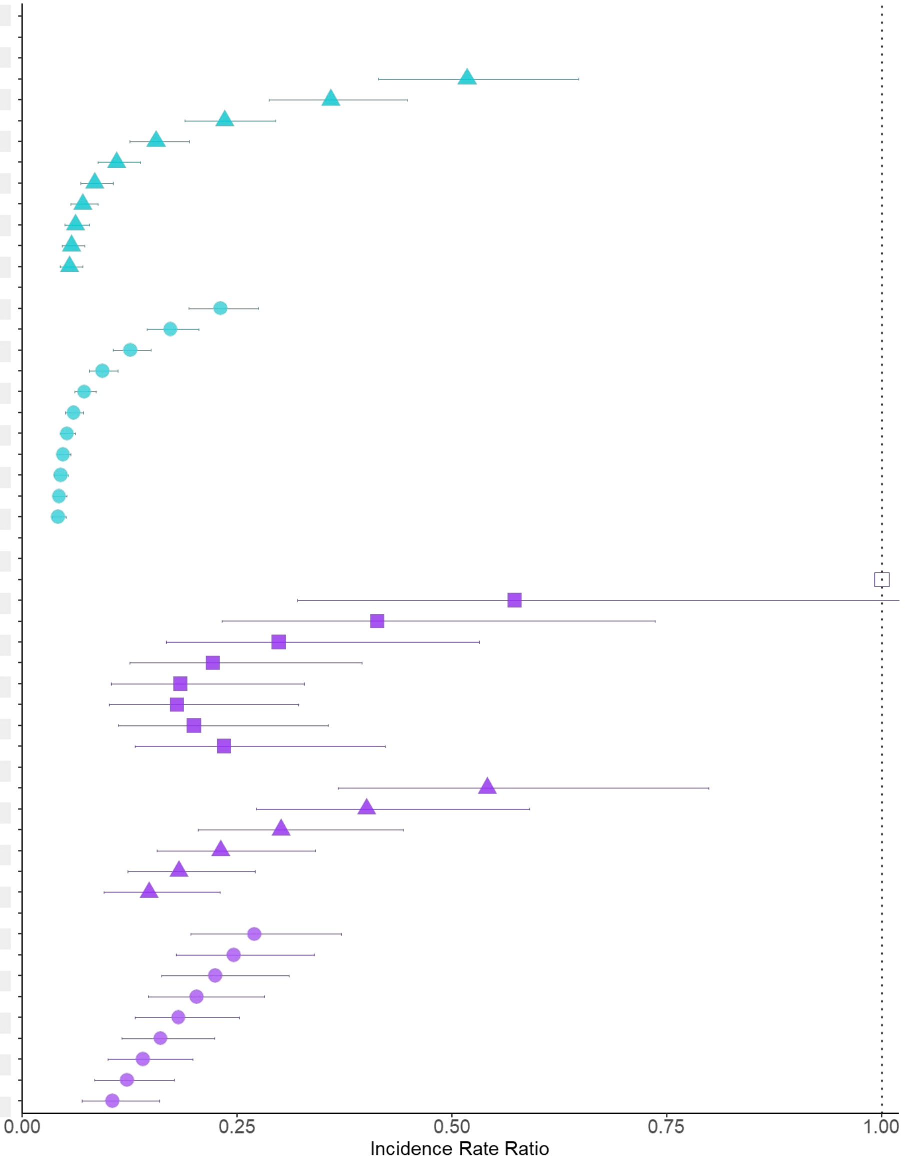

Supplementary Figure 3.54 All PCV13 ST IPD all-site weighted average IRRs comparing the annual post-PCV10/13 IR to the average pre-PCV IR for children aged 5–17 years

PCV Product and PCV7 Impact Strata	Number of Sites	Number of Cases	IRR (95%CI)
PCV13			
Moderate PCV7 impact			
Year −1	5	502	0.98 (0.77, 1.23)
Year 0	6	502	0.81 (0.64, 1.01)
Year 1	6	365	0.62 (0.49, 0.78)
Year 2	6	186	0.46 (0.37, 0.58)
Year 3	6	157	0.34 (0.27, 0.43)
Year 4	6	97	0.25 (0.2, 0.32)
Year 5	6	81	0.19 (0.15, 0.24)
Year 6	6	58	0.14 (0.11, 0.18)
Year 7	5	53	0.11 (0.09, 0.14)
Year 8	3	23	0.08 (0.07, 0.11)
Substantial PCV7 impact			
Year −1	10	305	0.52 (0.44, 0.63)
Year 0	10	321	0.44 (0.37, 0.53)
Year 1	10	239	0.36 (0.3, 0.43)
Year 2	10	157	0.29 (0.24, 0.34)
Year 3	10	107	0.23 (0.19, 0.27)
Year 4	10	87	0.18 (0.15, 0.21)
Year 5	10	91	0.14 (0.12, 0.17)
Year 6	10	87	0.11 (0.09, 0.13)
Year 7	7	49	0.09 (0.07, 0.1)
Year 8	4	13	0.07 (0.06, 0.08)
Year 9	1	13	0.05 (0.04, 0.07)
PCV10			
No PCV7 impact			
Year 0	--	--	--
Year 1	5	43	0.74 (0.53, 1.04)
Year 2	5	29	0.6 (0.45, 0.8)
Year 3	5	25	0.52 (0.39, 0.69)
Year 4	4	19	0.47 (0.35, 0.62)
Year 5	4	15	0.42 (0.31, 0.57)
Year 6	3	11	0.38 (0.28, 0.5)
Year 7	3	13	0.33 (0.23, 0.47)
Moderate PCV7 impact			
Year −1	2	117	0.92 (0.6, 1.4)
Year 0	2	51	0.64 (0.42, 0.96)
Year 1	2	45	0.52 (0.35, 0.78)
Year 2	2	37	0.47 (0.31, 0.7)
Year 3	1	17	0.43 (0.27, 0.66)
Year 4	1	20	0.38 (0.2, 0.69)
Substantial PCV7 impact			
Year −1	3	13	0.87 (0.63, 1.21)
Year 0	3	10	0.84 (0.61, 1.17)
Year 1	3	43	0.79 (0.56, 1.1)
Year 2	1	3	0.71 (0.51, 1)
Year 3	1	4	0.62 (0.44, 0.87)
Year 4	1	5	0.52 (0.37, 0.74)
Year 5	1	2	0.43 (0.3, 0.61)
Year 6	1	3	0.34 (0.23, 0.49)
Year 7	1	3	0.27 (0.18, 0.41)

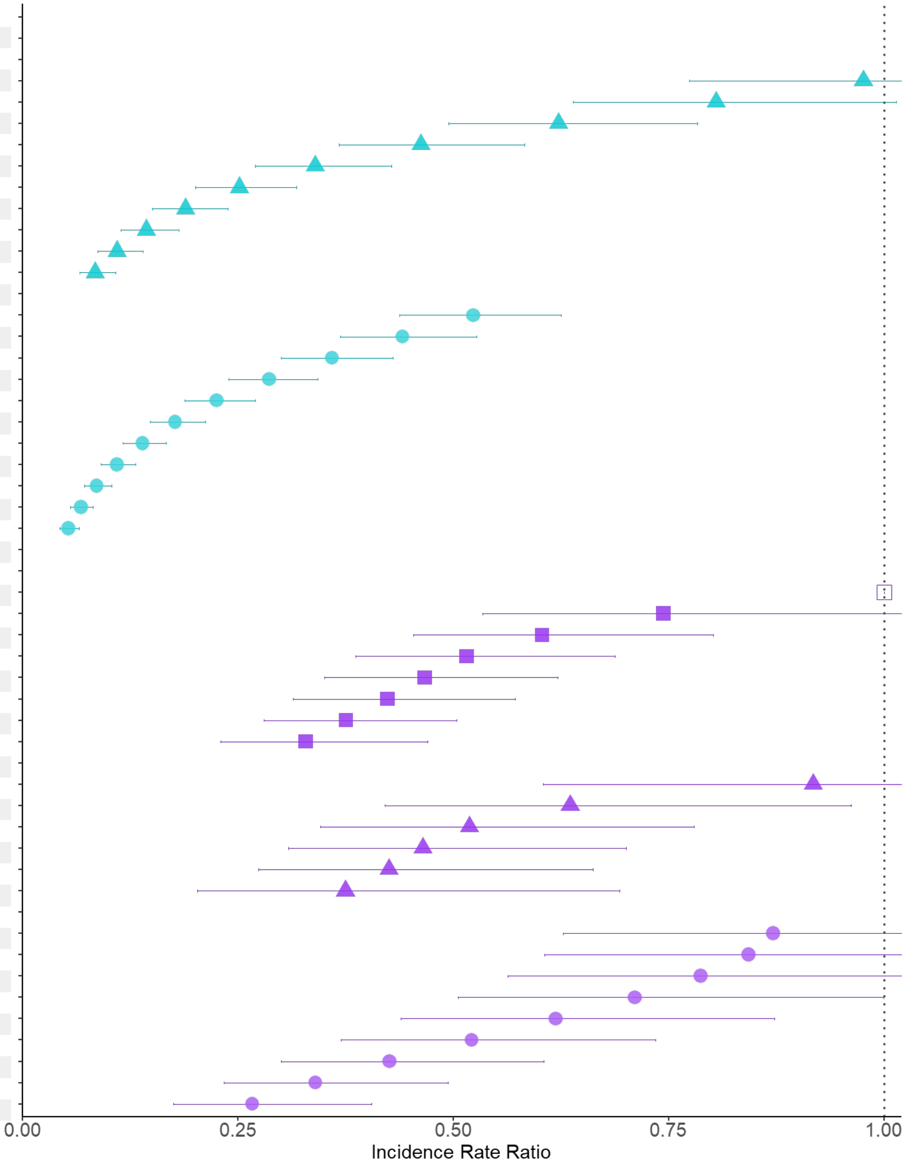

Supplementary Figure 3.55 All PCV13 ST IPD all-site weighted average IRRs comparing the annual post-PCV10/13 IR to the average pre-PCV IR for adults aged 18–49 years

PCV Product and PCV7 Impact Strata	Number of Sites	Number of Cases	IRR (95%CI)
PCV13			
Moderate PCV7 impact			
Year −1	5	1426	0.96 (0.91, 1.02)
Year 0	5	1433	0.85 (0.8, 0.91)
Year 1	5	1015	0.71 (0.67, 0.75)
Year 2	5	933	0.56 (0.53, 0.59)
Year 3	5	758	0.43 (0.41, 0.46)
Year 4	5	559	0.34 (0.32, 0.36)
Year 5	5	520	0.27 (0.25, 0.29)
Year 6	5	480	0.22 (0.21, 0.24)
Year 7	4	449	0.19 (0.18, 0.2)
Year 8	2	159	0.16 (0.15, 0.18)
Substantial PCV7 impact			
Year −1	10	1905	0.65 (0.52, 0.82)
Year 0	10	1621	0.54 (0.43, 0.68)
Year 1	10	1284	0.44 (0.35, 0.56)
Year 2	10	899	0.36 (0.29, 0.45)
Year 3	10	701	0.3 (0.23, 0.37)
Year 4	10	482	0.25 (0.2, 0.31)
Year 5	10	505	0.21 (0.17, 0.27)
Year 6	10	521	0.18 (0.15, 0.23)
Year 7	7	347	0.16 (0.13, 0.2)
Year 8	4	180	0.14 (0.11, 0.18)
Year 9	1	135	0.13 (0.1, 0.16)
PCV10			
No PCV7 impact			
Year 0	--	--	--
Year 1	5	179	0.58 (0.33, 1.03)
Year 2	5	131	0.42 (0.24, 0.74)
Year 3	5	132	0.33 (0.19, 0.58)
Year 4	4	100	0.29 (0.16, 0.5)
Year 5	4	100	0.28 (0.16, 0.49)
Year 6	3	95	0.3 (0.17, 0.52)
Year 7	3	109	0.33 (0.18, 0.58)
Substantial PCV7 impact			
Year −1	3	87	0.69 (0.45, 1.05)
Year 0	3	68	0.66 (0.43, 1)
Year 1	3	127	0.62 (0.41, 0.95)
Year 2	1	70	0.57 (0.38, 0.88)
Year 3	1	33	0.5 (0.33, 0.77)
Year 4	1	47	0.42 (0.27, 0.64)
Year 5	1	27	0.33 (0.22, 0.51)
Year 6	1	19	0.25 (0.17, 0.39)
Year 7	1	25	0.19 (0.12, 0.29)

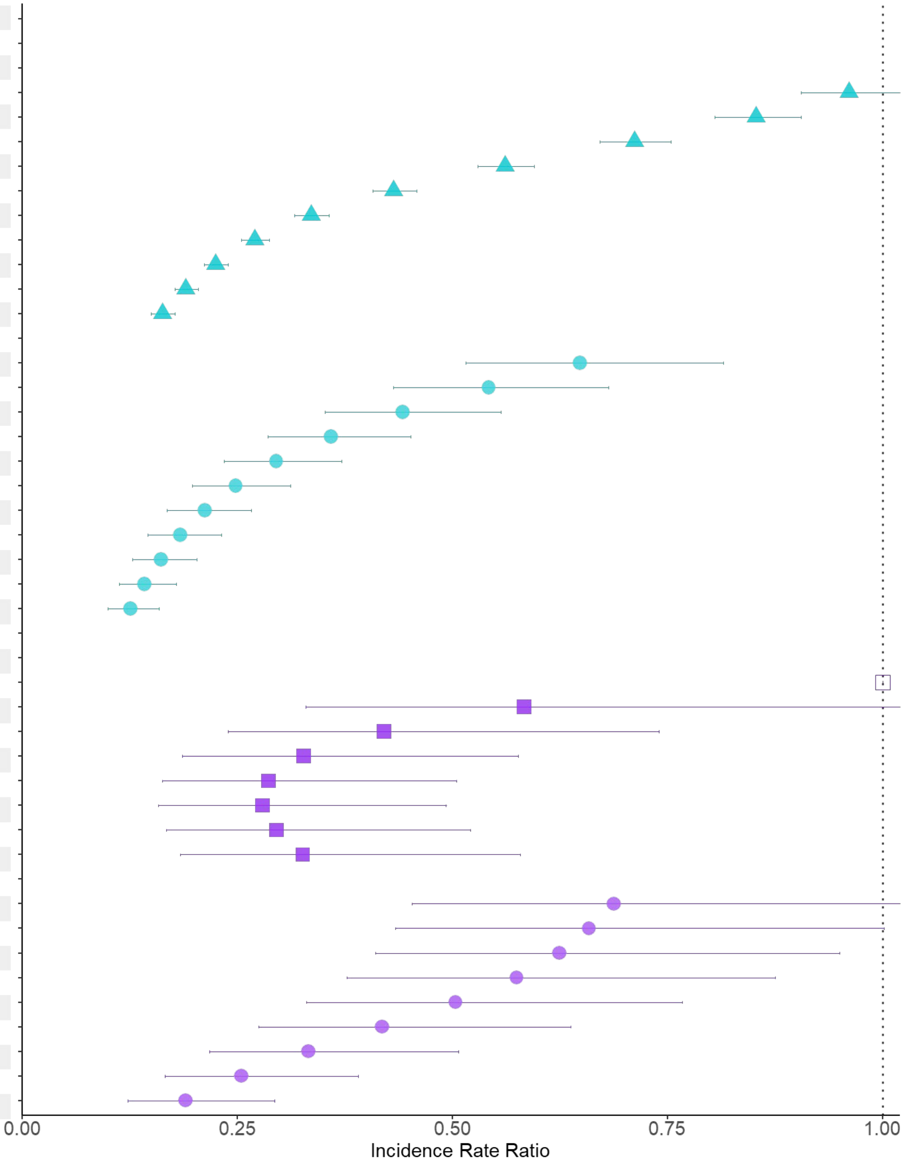

Supplementary Figure 3.56 All PCV13 ST IPD all-site weighted average IRRs comparing the annual post-PCV10/13 IR to the average pre-PCV IR for adults aged 50–64 years

PCV Product and PCV7 Impact Strata	Number of Sites	Number of Cases	IRR (95%CI)
PCV13			
Moderate PCV7 impact			
Year −1	5	585	0.95 (0.83, 1.08)
Year 0	5	713	0.85 (0.75, 0.98)
Year 1	5	527	0.73 (0.64, 0.83)
Year 2	5	624	0.59 (0.52, 0.68)
Year 3	5	567	0.47 (0.42, 0.54)
Year 4	5	433	0.39 (0.34, 0.44)
Year 5	5	452	0.33 (0.29, 0.37)
Year 6	5	404	0.29 (0.25, 0.33)
Year 7	4	410	0.26 (0.22, 0.29)
Year 8	2	328	0.23 (0.2, 0.27)
Substantial PCV7 impact			
Year −1	10	1561	1.34 (0.88, 2.04)
Year 0	10	1485	0.96 (0.63, 1.47)
Year 1	10	1194	0.69 (0.45, 1.06)
Year 2	10	863	0.5 (0.33, 0.77)
Year 3	10	770	0.39 (0.25, 0.59)
Year 4	10	583	0.32 (0.21, 0.48)
Year 5	10	612	0.27 (0.17, 0.41)
Year 6	10	697	0.23 (0.15, 0.35)
Year 7	7	567	0.2 (0.13, 0.3)
Year 8	4	249	0.17 (0.11, 0.26)
Year 9	1	196	0.15 (0.09, 0.23)
PCV10			
No PCV7 impact			
Year 0	--	--	--
Year 1	4	190	0.53 (0.29, 0.96)
Year 2	4	195	0.45 (0.24, 0.81)
Year 3	4	171	0.43 (0.23, 0.78)
Year 4	2	101	0.45 (0.25, 0.82)
Year 5	2	121	0.47 (0.25, 0.85)
Year 6	2	115	0.46 (0.25, 0.85)
Year 7	2	114	0.45 (0.24, 0.83)
Substantial PCV7 impact			
Year −1	3	94	1.34 (0.88, 2.04)
Year 0	3	90	0.96 (0.63, 1.47)
Year 1	3	164	0.69 (0.45, 1.06)
Year 2	1	73	0.5 (0.33, 0.77)
Year 3	1	52	0.39 (0.25, 0.59)
Year 4	1	52	0.32 (0.21, 0.48)
Year 5	1	53	0.27 (0.17, 0.41)
Year 6	1	59	0.23 (0.15, 0.35)
Year 7	1	63	0.2 (0.13, 0.3)

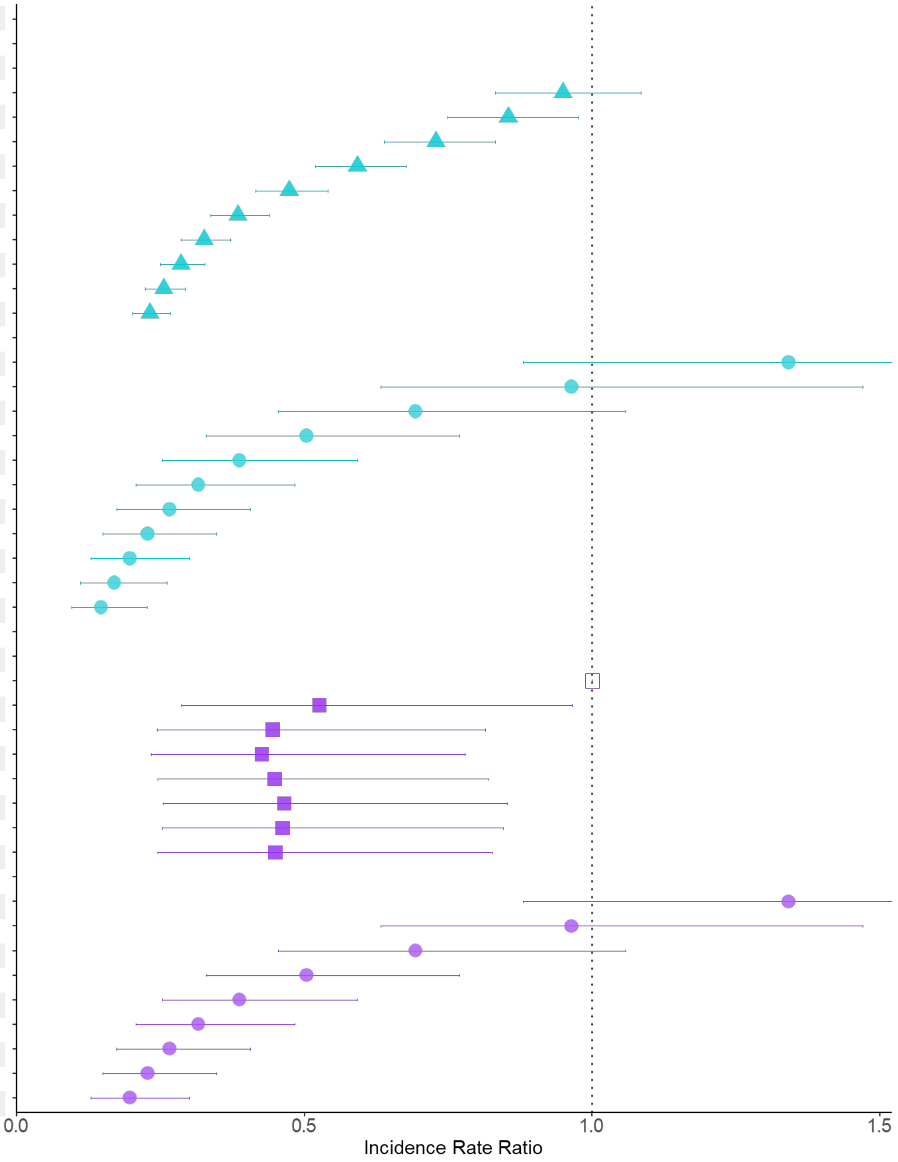

Supplementary Figure 3.57 All PCV13 ST IPD all-site weighted average IRRs comparing the annual post-PCV10/13 IR to the average pre-PCV IR for adults aged ≥65 years

PCV Product and PCV7 Impact Strata	Number of Sites	Number of Cases	IRR (95%CI)
PCV13			
Moderate PCV7 impact			
Year −1	5	1018	0.84 (0.7, 1.01)
Year 0	5	1102	0.75 (0.63, 0.9)
Year 1	5	962	0.65 (0.54, 0.78)
Year 2	5	1305	0.54 (0.45, 0.65)
Year 3	5	1173	0.45 (0.37, 0.54)
Year 4	5	972	0.37 (0.31, 0.45)
Year 5	5	1062	0.32 (0.27, 0.39)
Year 6	5	975	0.29 (0.24, 0.35)
Year 7	4	920	0.26 (0.22, 0.31)
Year 8	2	753	0.24 (0.2, 0.29)
Substantial PCV7 impact			
Year −1	10	2508	0.53 (0.46, 0.6)
Year 0	10	2200	0.45 (0.39, 0.51)
Year 1	10	1744	0.37 (0.33, 0.43)
Year 2	10	1329	0.32 (0.28, 0.36)
Year 3	10	1067	0.28 (0.24, 0.32)
Year 4	10	851	0.25 (0.22, 0.29)
Year 5	10	1014	0.24 (0.21, 0.27)
Year 6	10	1192	0.23 (0.2, 0.26)
Year 7	7	939	0.23 (0.2, 0.26)
Year 8	4	643	0.22 (0.2, 0.26)
Year 9	1	490	0.22 (0.19, 0.26)
PCV10			
No PCV7 impact			
Year 0	--	--	--
Year 1	4	323	0.86 (0.52, 1.42)
Year 1	4	323	0.86 (0.52, 1.42)
Year 2	4	350	0.77 (0.47, 1.28)
Year 2	4	350	0.77 (0.47, 1.28)
Year 3	4	302	0.74 (0.45, 1.22)
Year 3	4	302	0.74 (0.45, 1.22)
Year 4	3	221	0.75 (0.45, 1.24)
Year 4	3	221	0.75 (0.45, 1.24)
Year 5	3	230	0.75 (0.45, 1.24)
Year 5	3	230	0.75 (0.45, 1.24)
Year 6	2	228	0.72 (0.44, 1.19)
Year 6	2	228	0.72 (0.44, 1.19)
Year 7	2	233	0.69 (0.41, 1.14)
Year 7	2	233	0.69 (0.41, 1.14)
Substantial PCV7 impact			
Year −1	3	207	0.62 (0.49, 0.8)
Year 0	3	174	0.56 (0.43, 0.71)
Year 1	3	252	0.49 (0.38, 0.63)
Year 2	1	148	0.44 (0.34, 0.57)
Year 3	1	112	0.4 (0.31, 0.51)
Year 4	1	152	0.37 (0.29, 0.48)
Year 5	1	133	0.35 (0.27, 0.45)
Year 6	1	122	0.34 (0.26, 0.43)
Year 7	1	137	0.32 (0.25, 0.42)

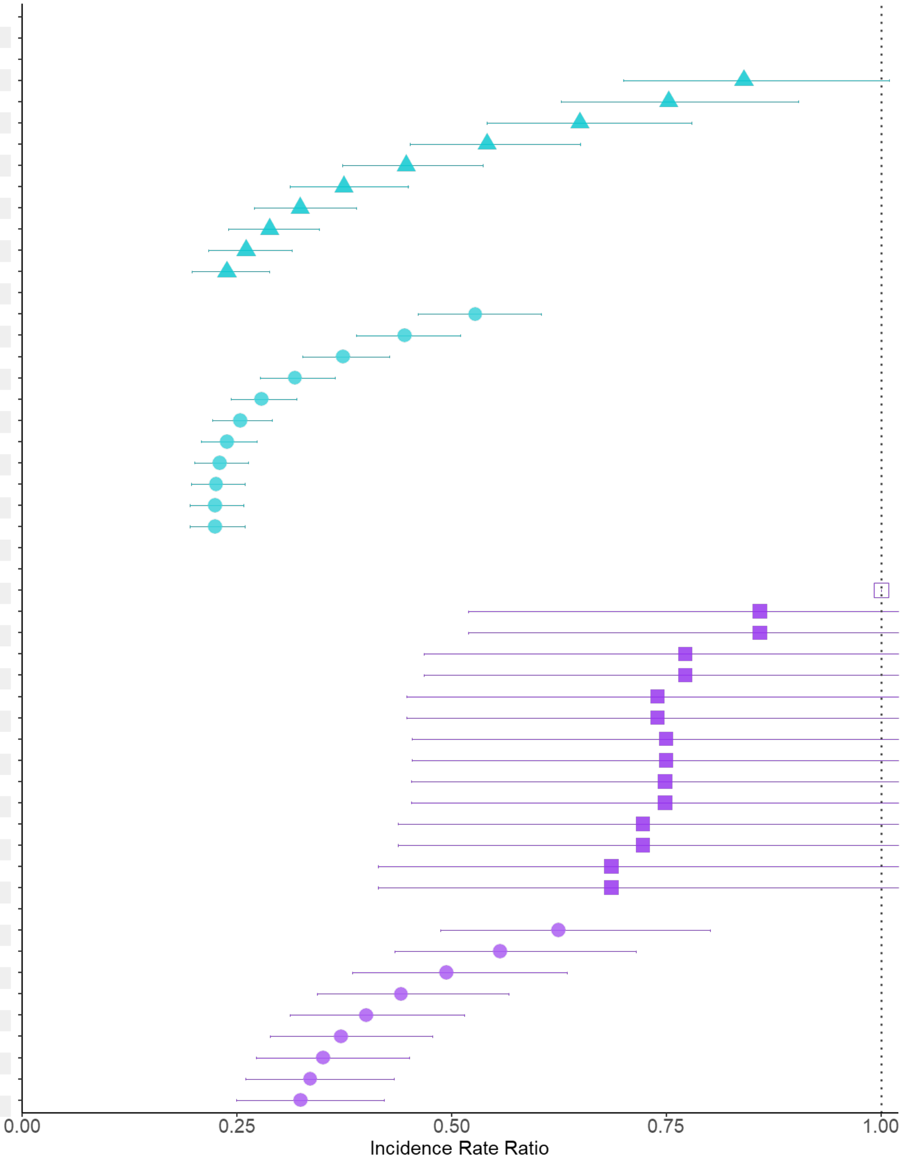

Supplementary Figure 3.58 All PCV13 ST minus ST3 IPD all-site weighted average IRRs comparing the annual post-PCV10/13 IR to the average pre-PCV IR for children aged <5 years

PCV Product and PCV7 Impact Strata	Number of Sites	Number of Cases	IRR (95%CI)
PCV13			
Moderate PCV7 impact			
Year −1	6	1453	0.5 (0.4, 0.64)
Year 0	6	1172	0.34 (0.27, 0.43)
Year 1	6	811	0.22 (0.17, 0.27)
Year 2	6	487	0.14 (0.11, 0.17)
Year 3	6	242	0.09 (0.07, 0.12)
Year 4	6	170	0.07 (0.05, 0.08)
Year 5	6	89	0.05 (0.04, 0.06)
Year 6	6	113	0.04 (0.03, 0.05)
Year 7	5	78	0.03 (0.03, 0.04)
Year 8	3	80	0.03 (0.02, 0.04)
Substantial PCV7 impact			
Year −1	11	695	0.22 (0.19, 0.26)
Year 0	11	762	0.16 (0.14, 0.19)
Year 1	11	582	0.11 (0.1, 0.14)
Year 2	11	256	0.08 (0.07, 0.1)
Year 3	11	146	0.06 (0.05, 0.07)
Year 4	11	119	0.04 (0.04, 0.05)
Year 5	11	97	0.04 (0.03, 0.04)
Year 6	11	88	0.03 (0.02, 0.03)
Year 7	7	93	0.02 (0.02, 0.03)
Year 8	4	37	0.02 (0.02, 0.02)
Year 9	1	22	0.02 (0.01, 0.02)
PCV10			
No PCV7 impact			
Year −1	--	--	--
Year 0	4	192	0.61 (0.36, 1.03)
Year 1	4	148	0.41 (0.24, 0.68)
Year 2	4	102	0.27 (0.16, 0.46)
Year 3	4	48	0.19 (0.11, 0.32)
Year 4	3	52	0.16 (0.09, 0.26)
Year 5	3	18	0.15 (0.09, 0.26)
Year 6	2	20	0.17 (0.1, 0.29)
Year 7	2	19	0.21 (0.12, 0.35)
Moderate PCV7 impact			
Year −1	2	255	0.53 (0.35, 0.79)
Year 0	2	191	0.38 (0.25, 0.57)
Year 1	2	154	0.28 (0.18, 0.42)
Year 2	2	99	0.2 (0.14, 0.31)
Year 3	1	76	0.16 (0.1, 0.24)
Year 4	1	51	0.12 (0.08, 0.19)
Substantial PCV7 impact			
Year −1	4	10	0.24 (0.18, 0.33)
Year 0	4	21	0.22 (0.16, 0.3)
Year 1	4	63	0.2 (0.15, 0.27)
Year 2	1	53	0.18 (0.13, 0.24)
Year 3	1	2	0.16 (0.12, 0.21)
Year 4	1	5	0.13 (0.1, 0.18)
Year 5	1	4	0.11 (0.08, 0.15)
Year 6	1	5	0.09 (0.06, 0.13)
Year 7	1	5	0.07 (0.05, 0.11)

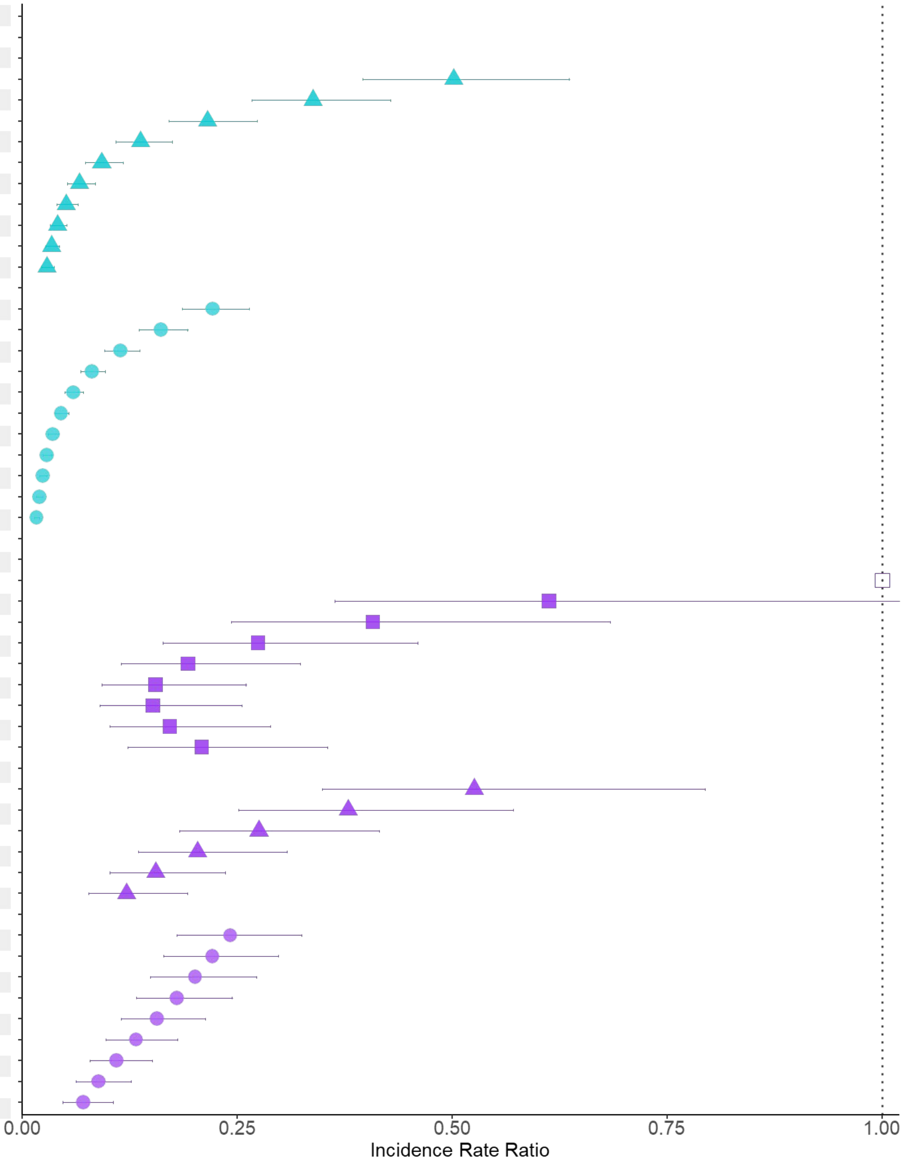

Supplementary Figure 3.59 All PCV13 ST minus ST3 IPD all-site weighted average IRRs comparing the annual post-PCV10/13 IR to the average pre-PCV IR for **children aged 5–17 years**

PCV Product and PCV7 Impact Strata	Number of Sites	Number of Cases	IRR (95%CI)
PCV13			
Moderate PCV7 impact			
Year −1	5	490	1 (0.81, 1.24)
Year 0	6	492	0.81 (0.65, 1)
Year 1	6	694	0.6 (0.49, 0.75)
Year 2	6	332	0.43 (0.35, 0.54)
Year 3	6	290	0.31 (0.25, 0.38)
Year 4	6	168	0.22 (0.18, 0.27)
Year 5	6	138	0.16 (0.13, 0.2)
Year 6	6	90	0.12 (0.09, 0.14)
Year 7	5	86	0.09 (0.07, 0.11)
Year 8	3	34	0.06 (0.05, 0.08)
Substantial PCV7 impact			
Year −1	10	292	0.5 (0.42, 0.58)
Year 0	10	303	0.41 (0.35, 0.48)
Year 1	10	434	0.32 (0.27, 0.38)
Year 2	10	274	0.25 (0.21, 0.29)
Year 3	10	190	0.19 (0.16, 0.22)
Year 4	10	142	0.14 (0.12, 0.16)
Year 5	10	138	0.1 (0.09, 0.12)
Year 6	10	102	0.08 (0.06, 0.09)
Year 7	7	70	0.06 (0.05, 0.07)
Year 8	4	20	0.04 (0.04, 0.05)
Year 9	1	18	0.03 (0.03, 0.04)
PCV10			
No PCV7 impact			
Year 0	--	--	--
Year 1	5	80	0.73 (0.52, 1.02)
Year 2	5	52	0.58 (0.43, 0.79)
Year 3	5	48	0.48 (0.36, 0.66)
Year 4	4	28	0.41 (0.3, 0.56)
Year 5	4	24	0.34 (0.25, 0.47)
Year 6	3	12	0.26 (0.19, 0.36)
Year 7	3	16	0.2 (0.14, 0.29)
Moderate PCV7 impact			
Year −1	2	117	0.93 (0.63, 1.37)
Year 0	2	49	0.63 (0.43, 0.93)
Year 1	2	88	0.51 (0.35, 0.75)
Year 2	2	70	0.46 (0.32, 0.67)
Year 3	1	34	0.42 (0.28, 0.63)
Year 4	1	38	0.37 (0.21, 0.65)
Substantial PCV7 impact			
Year −1	3	12	0.84 (0.62, 1.13)
Year 0	3	9	0.81 (0.6, 1.1)
Year 1	3	84	0.76 (0.56, 1.04)
Year 2	1	4	0.69 (0.5, 0.94)
Year 3	1	8	0.6 (0.44, 0.82)
Year 4	1	10	0.5 (0.36, 0.69)
Year 5	1	4	0.41 (0.29, 0.56)
Year 6	1	4	0.32 (0.22, 0.45)
Year 7	1	6	0.25 (0.17, 0.37)

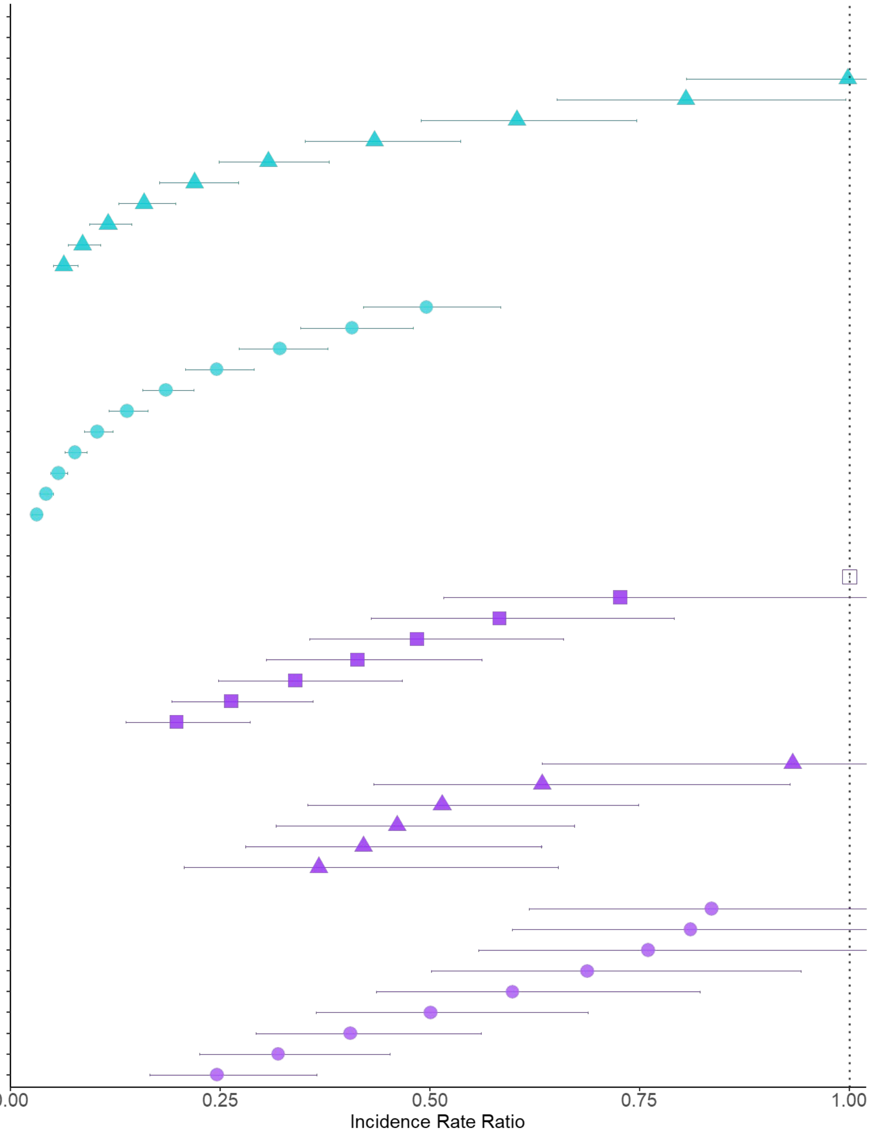

Supplementary Figure 3.60 All PCV13 ST minus ST3 IPD all-site weighted average IRRs comparing the annual post-PCV10/13 IR to the average pre-PCV IR for adults aged 18–49 years

PCV Product and PCV7 Impact Strata	Number of Sites	Number of Cases	IRR (95%CI)
PCV13			
Moderate PCV7 impact			
Year −1	4	1327	0.97 (0.86, 1.08)
Year 0	4	1344	0.84 (0.75, 0.94)
Year 1	4	1836	0.67 (0.6, 0.75)
Year 2	4	1608	0.51 (0.45, 0.57)
Year 3	4	1284	0.37 (0.33, 0.41)
Year 4	4	888	0.27 (0.24, 0.3)
Year 5	4	788	0.2 (0.18, 0.23)
Year 6	4	680	0.15 (0.14, 0.17)
Year 7	3	634	0.12 (0.1, 0.13)
Year 8	2	168	0.09 (0.08, 0.11)
Substantial PCV7 impact			
Year −1	10	1718	0.61 (0.47, 0.79)
Year 0	10	1454	0.51 (0.39, 0.66)
Year 1	10	2248	0.41 (0.31, 0.53)
Year 2	10	1516	0.32 (0.25, 0.41)
Year 3	10	1132	0.25 (0.19, 0.33)
Year 4	10	738	0.2 (0.15, 0.26)
Year 5	10	712	0.16 (0.12, 0.2)
Year 6	10	650	0.12 (0.1, 0.16)
Year 7	7	408	0.1 (0.08, 0.13)
Year 8	4	214	0.08 (0.06, 0.1)
Year 9	1	130	0.06 (0.05, 0.08)
PCV10			
No PCV7 impact			
Year 0	--	--	--
Year 1	5	320	0.6 (0.32, 1.1)
Year 2	5	230	0.41 (0.23, 0.76)
Year 3	5	224	0.31 (0.17, 0.56)
Year 4	4	146	0.25 (0.14, 0.46)
Year 5	4	140	0.23 (0.13, 0.42)
Year 6	3	114	0.22 (0.12, 0.41)
Year 7	3	138	0.22 (0.12, 0.41)
Substantial PCV7 impact			
Year −1	3	81	0.65 (0.41, 1.04)
Year 0	3	61	0.63 (0.39, 1.01)
Year 1	3	238	0.6 (0.37, 0.96)
Year 2	1	118	0.55 (0.34, 0.88)
Year 3	1	60	0.47 (0.29, 0.76)
Year 4	1	86	0.38 (0.23, 0.61)
Year 5	1	42	0.28 (0.18, 0.46)
Year 6	1	30	0.2 (0.13, 0.33)
Year 7	1	36	0.14 (0.09, 0.23)

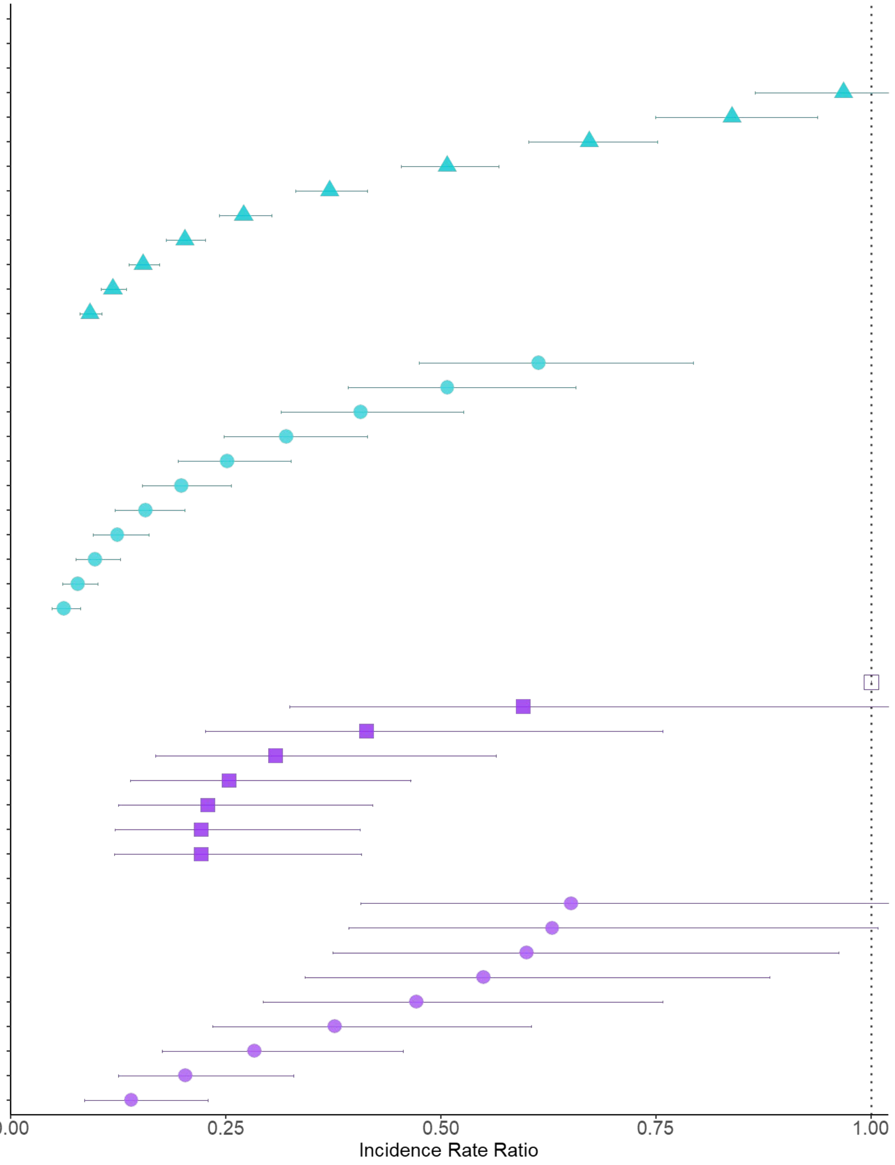

Supplementary Figure 3.61 All PCV13 ST minus ST3 IPD all-site weighted average IRRs comparing the annual post-PCV10/13 IR to the average pre-PCV IR for adults aged 50–64 years

PCV Product and PCV7 Impact Strata	Number of Sites	Number of Cases	IRR (95%CI)
PCV13			
Moderate PCV7 impact			
Year −1	4	517	0.93 (0.77, 1.11)
Year 0	4	614	0.81 (0.67, 0.97)
Year 1	4	435	0.65 (0.55, 0.78)
Year 2	4	482	0.5 (0.42, 0.6)
Year 3	4	404	0.37 (0.31, 0.45)
Year 4	4	274	0.28 (0.23, 0.34)
Year 5	4	247	0.22 (0.18, 0.26)
Year 6	4	218	0.17 (0.14, 0.21)
Year 7	3	210	0.14 (0.12, 0.17)
Year 8	2	120	0.11 (0.09, 0.14)
Substantial PCV7 impact			
Year −1	10	1326	0.61 (0.53, 0.71)
Year 0	10	1228	0.52 (0.45, 0.6)
Year 1	10	962	0.43 (0.37, 0.5)
Year 2	10	630	0.35 (0.3, 0.4)
Year 3	10	551	0.29 (0.25, 0.33)
Year 4	10	390	0.24 (0.2, 0.27)
Year 5	10	370	0.2 (0.17, 0.23)
Year 6	10	379	0.17 (0.14, 0.19)
Year 7	7	267	0.14 (0.12, 0.16)
Year 8	4	115	0.12 (0.1, 0.14)
Year 9	1	94	0.1 (0.09, 0.12)
PCV10			
No PCV7 impact			
Year 0	--	--	--
Year 1	4	162	0.57 (0.33, 1)
Year 2	4	158	0.45 (0.26, 0.79)
Year 3	4	128	0.4 (0.23, 0.7)
Year 4	2	78	0.38 (0.22, 0.67)
Year 5	2	84	0.37 (0.21, 0.64)
Year 6	2	69	0.35 (0.2, 0.61)
Year 7	2	76	0.33 (0.19, 0.58)
Substantial PCV7 impact			
Year −1	3	82	0.76 (0.58, 0.99)
Year 0	3	80	0.69 (0.53, 0.91)
Year 1	3	132	0.61 (0.46, 0.8)
Year 2	1	67	0.53 (0.4, 0.7)
Year 3	1	42	0.46 (0.35, 0.61)
Year 4	1	44	0.41 (0.31, 0.53)
Year 5	1	42	0.36 (0.27, 0.48)
Year 6	1	42	0.32 (0.25, 0.43)
Year 7	1	47	0.29 (0.22, 0.39)

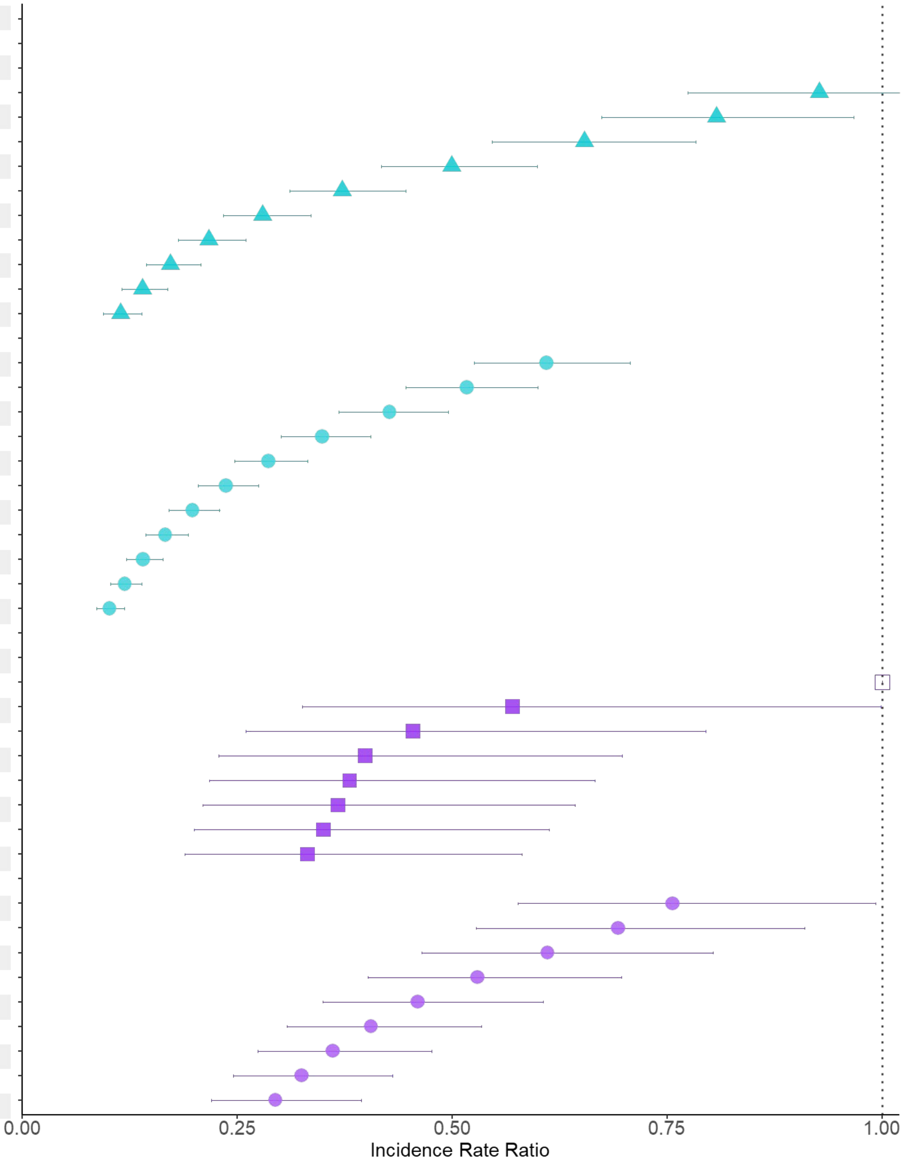

Supplementary Figure 3.62 All PCV13 ST minus ST3 IPD all-site weighted average IRRs comparing the annual post-PCV10/13 IR to the average pre-PCV IR for adults aged ≥65 years

PCV Product and PCV7 Impact Strata	Number of Sites	Number of Cases	IRR (95%CI)
PCV13			
Moderate PCV7 impact			
Year −1	4	845	0.81 (0.59, 1.1)
Year 0	4	911	0.7 (0.52, 0.96)
Year 1	4	731	0.58 (0.43, 0.79)
Year 2	4	945	0.46 (0.34, 0.63)
Year 3	4	783	0.36 (0.26, 0.49)
Year 4	4	607	0.28 (0.2, 0.38)
Year 5	4	508	0.22 (0.16, 0.29)
Year 6	4	431	0.17 (0.12, 0.23)
Year 7	3	361	0.13 (0.1, 0.18)
Year 8	2	261	0.11 (0.08, 0.15)
Substantial PCV7 impact			
Year −1	10	2063	0.48 (0.4, 0.56)
Year 0	10	1723	0.38 (0.32, 0.45)
Year 1	10	1280	0.3 (0.25, 0.36)
Year 2	10	932	0.24 (0.2, 0.28)
Year 3	10	665	0.19 (0.16, 0.23)
Year 4	10	506	0.16 (0.13, 0.19)
Year 5	10	514	0.13 (0.11, 0.16)
Year 6	10	528	0.11 (0.09, 0.13)
Year 7	7	380	0.1 (0.08, 0.11)
Year 8	4	260	0.08 (0.07, 0.1)
Year 9	1	185	0.07 (0.06, 0.09)
PCV10			
No PCV7 impact			
Year 0	--	--	--
Year 1	4	262	0.88 (0.57, 1.36)
Year 2	4	278	0.77 (0.5, 1.18)
Year 3	4	232	0.71 (0.46, 1.09)
Year 4	3	159	0.69 (0.45, 1.06)
Year 5	3	152	0.66 (0.43, 1.01)
Year 6	2	153	0.6 (0.39, 0.93)
Year 7	2	156	0.54 (0.35, 0.84)
Substantial PCV7 impact			
Year −1	3	180	0.62 (0.45, 0.85)
Year 0	3	153	0.55 (0.4, 0.75)
Year 1	3	211	0.48 (0.35, 0.66)
Year 2	1	120	0.42 (0.31, 0.58)
Year 3	1	92	0.38 (0.27, 0.52)
Year 4	1	127	0.33 (0.24, 0.46)
Year 5	1	99	0.3 (0.22, 0.41)
Year 6	1	89	0.27 (0.2, 0.38)
Year 7	1	85	0.25 (0.18, 0.34)

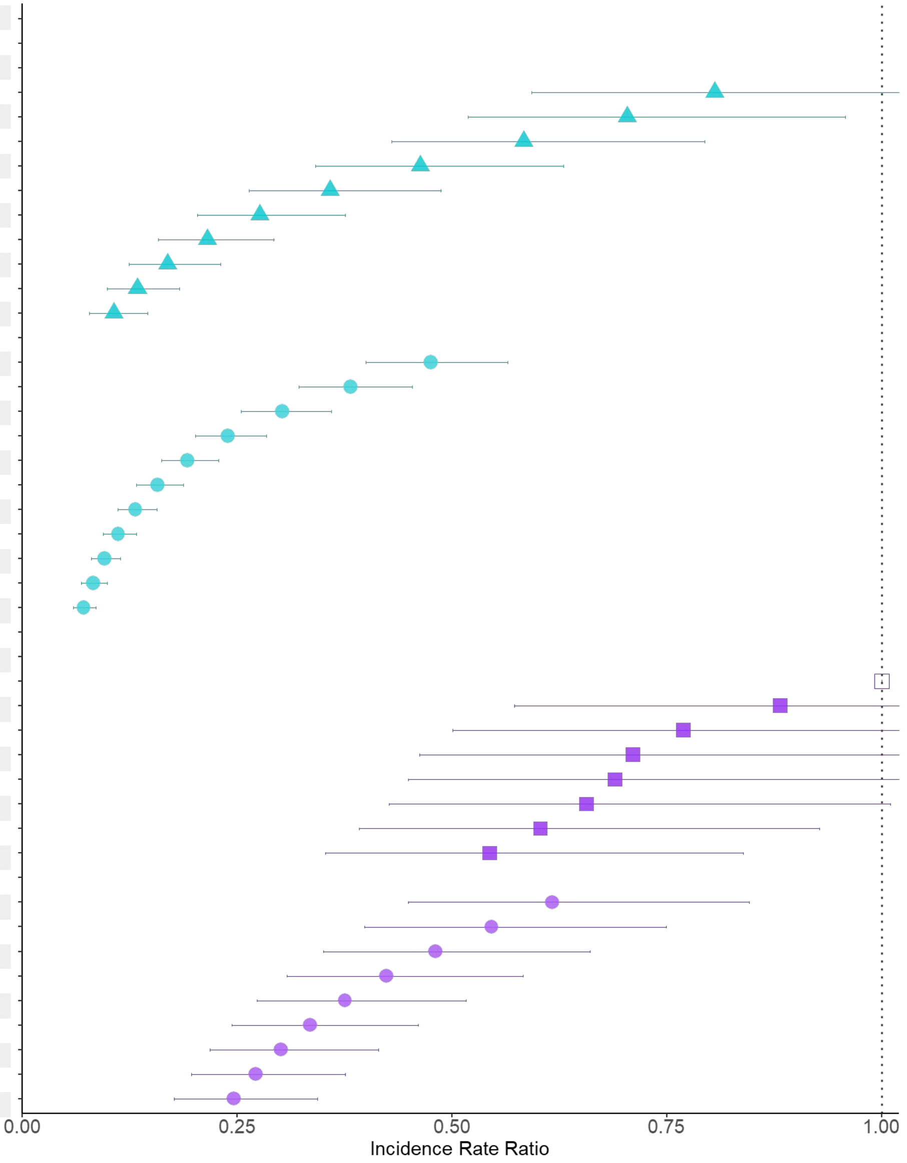

Supplementary Figure 3.63 All non-PCV10 ST IPD all-site weighted average IRRs comparing the annual post-PCV10/13 IR to the average pre-PCV IR for children aged <5 years

PCV Product and PCV7 Impact Strata	Number of Sites	Number of Cases	IRR (95%CI)
PCV10			
No PCV7 impact			
Year −1	--	--	--
Year 0	4	40	0.58 (0.42, 0.81)
Year 1	4	26	0.75 (0.54, 1.03)
Year 2	4	39	0.91 (0.65, 1.25)
Year 3	4	29	1.02 (0.74, 1.42)
Year 4	3	56	1.14 (0.82, 1.58)
Year 5	3	30	1.31 (0.95, 1.82)
Year 6	2	25	1.55 (1.12, 2.15)
Year 7	2	32	1.85 (1.33, 2.58)
Moderate PCV7 impact			
Year −1	2	26	1.06 (0.63, 1.79)
Year 0	2	28	1.17 (0.69, 1.96)
Year 1	2	79	1.24 (0.74, 2.09)
Year 2	2	93	1.33 (0.79, 2.23)
Year 3	1	102	1.46 (0.86, 2.47)
Year 4	1	78	1.67 (0.95, 2.93)
Substantial PCV7 impact			
Year −1	4	11	2.1 (1.45, 3.05)
Year 0	4	14	2.12 (1.46, 3.07)
Year 1	4	94	2.12 (1.46, 3.09)
Year 2	1	62	2.14 (1.47, 3.13)
Year 3	1	5	2.22 (1.52, 3.24)
Year 4	1	12	2.35 (1.6, 3.43)
Year 5	1	14	2.54 (1.72, 3.73)
Year 6	1	8	2.79 (1.87, 4.16)
Year 7	1	15	3.11 (2.03, 4.75)

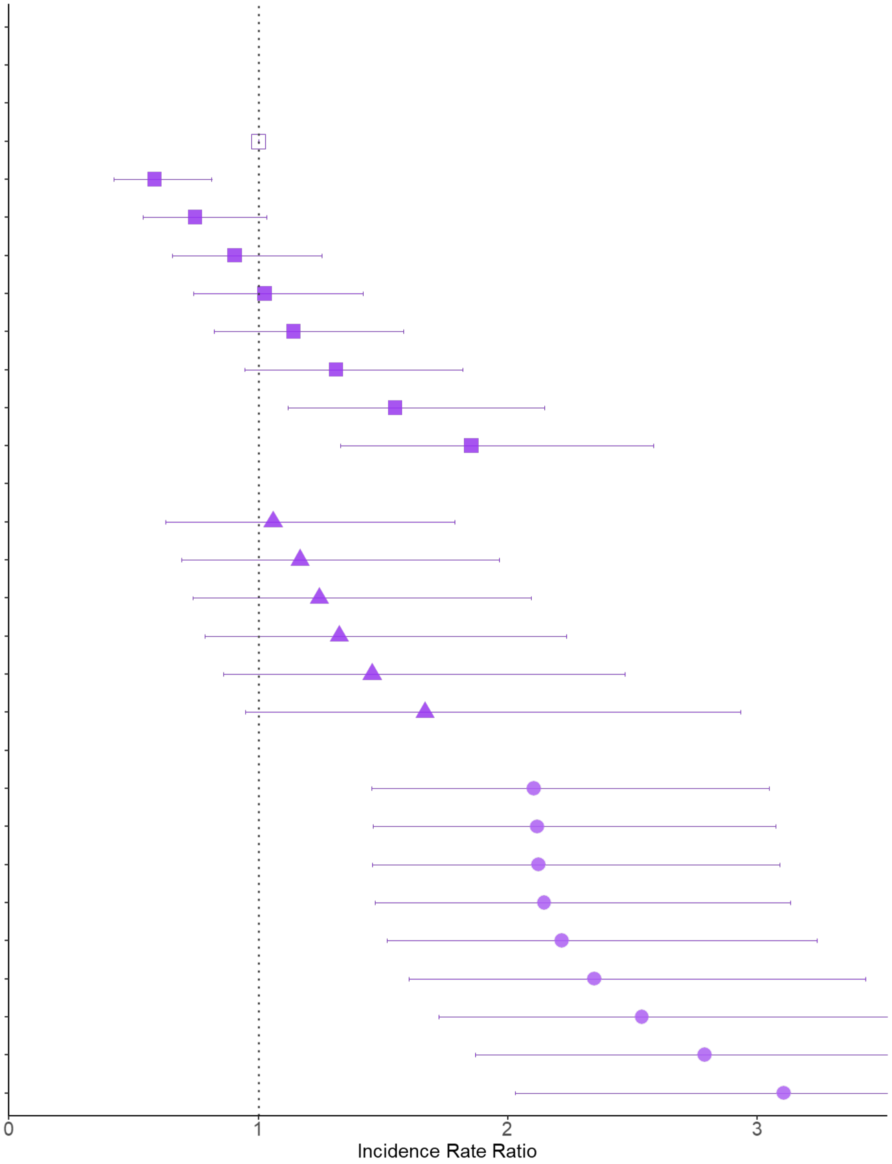

Supplementary Figure 3.64 All non-PCV10 ST IPD all-site weighted average IRRs comparing the annual post-PCV10/13 IR to the average pre-PCV IR for children aged 5–17 years

PCV Product and PCV7 Impact Strata	Number of Sites	Number of Cases	IRR (95%CI)
PCV10			
No PCV7 impact			
Year 0	--	--	--
Year 1	5	11	1.01 (0.45, 2.26)
Year 2	5	14	1.22 (0.55, 2.72)
Year 3	5	13	1.46 (0.66, 3.25)
Year 4	4	14	1.71 (0.77, 3.8)
Year 5	4	13	1.93 (0.87, 4.3)
Year 6	3	16	2.11 (0.95, 4.71)
Year 7	3	15	2.27 (1.02, 5.09)
Moderate PCV7 impact			
Year −1	2	5	0.85 (0.54, 1.32)
Year 0	2	6	1.13 (0.72, 1.76)
Year 1	2	22	1.43 (0.92, 2.23)
Year 2	2	28	1.67 (1.07, 2.6)
Year 3	1	8	1.71 (1.09, 2.7)
Year 4	1	11	1.5 (0.89, 2.53)
Substantial PCV7 impact			
Year −1	3	3	1.65 (1.15, 2.36)
Year 0	3	4	1.68 (1.17, 2.41)
Year 1	3	14	1.69 (1.18, 2.43)
Year 2	1	6	1.71 (1.19, 2.46)
Year 3	1	6	1.78 (1.24, 2.57)
Year 4	1	3	1.94 (1.35, 2.79)
Year 5	1	4	2.2 (1.52, 3.17)
Year 6	1	8	2.57 (1.77, 3.73)
Year 7	1	6	3.08 (2.1, 4.54)

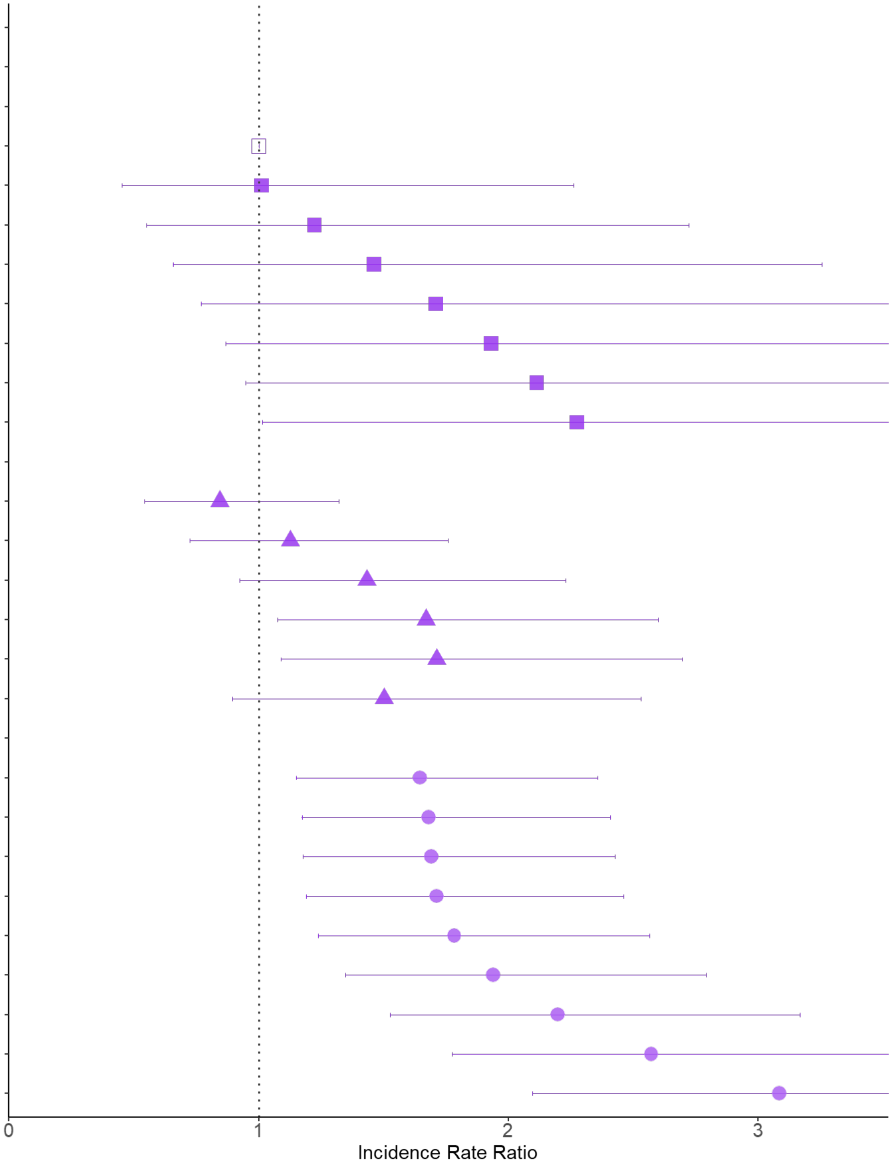

Supplementary Figure 3.65 All non-PCV10 ST IPD all-site weighted average IRRs comparing the annual post-PCV10/13 IR to the average pre-PCV IR for adults aged 18–49 years

PCV Product and PCV7 Impact Strata	Number of Sites	Number of Cases	IRR (95%CI)
PCV10			
No PCV7 impact			
Year 0	--	--	--
Year 1	5	80	0.9 (0.53, 1.52)
Year 2	5	73	0.91 (0.54, 1.52)
Year 3	5	96	0.92 (0.55, 1.55)
Year 4	4	96	0.95 (0.57, 1.59)
Year 5	4	115	1.01 (0.6, 1.69)
Year 6	3	120	1.1 (0.65, 1.84)
Year 7	3	121	1.21 (0.71, 2.05)
Substantial PCV7 impact			
Year −1	3	39	1.24 (1.02, 1.51)
Year 0	3	32	1.31 (1.07, 1.59)
Year 1	3	99	1.39 (1.15, 1.7)
Year 2	1	59	1.49 (1.23, 1.82)
Year 3	1	39	1.59 (1.31, 1.94)
Year 4	1	61	1.69 (1.38, 2.06)
Year 5	1	51	1.78 (1.46, 2.17)
Year 6	1	49	1.86 (1.52, 2.28)
Year 7	1	59	1.94 (1.58, 2.4)

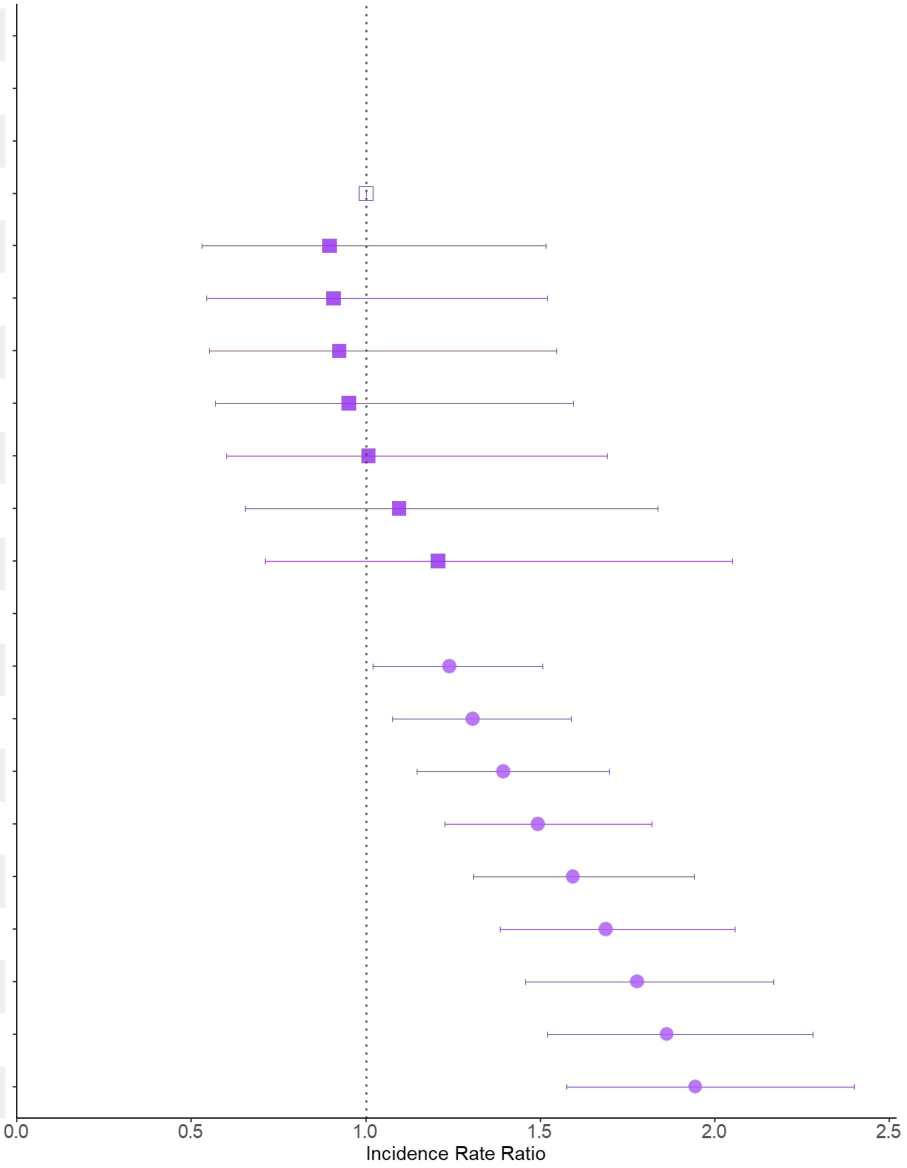

Supplementary Figure 3.66 All non-PCV10 ST IPD all-site weighted average IRRs comparing the annual post-PCV10/13 IR to the average pre-PCV IR for adults aged 50–64 years

PCV Product and PCV7 Impact Strata	Number of Sites	Number of Cases	IRR (95%CI)
PCV10			
No PCV7 impact			
Year 0	--	--	--
Year 1	4	132	0.88 (0.71, 1.08)
Year 2	4	158	0.97 (0.79, 1.19)
Year 3	4	168	1.04 (0.85, 1.27)
Year 4	2	112	1.09 (0.89, 1.34)
Year 5	2	162	1.19 (0.97, 1.45)
Year 6	2	178	1.33 (1.09, 1.63)
Year 7	2	170	1.52 (1.24, 1.87)
Substantial PCV7 impact			
Year −1	3	72	1.46 (1.18, 1.81)
Year 0	3	94	1.63 (1.32, 2.02)
Year 1	3	204	1.85 (1.49, 2.29)
Year 2	1	98	2.07 (1.67, 2.57)
Year 3	1	116	2.26 (1.82, 2.81)
Year 4	1	123	2.38 (1.92, 2.96)
Year 5	1	134	2.44 (1.96, 3.04)
Year 6	1	149	2.44 (1.96, 3.05)
Year 7	1	154	2.4 (1.91, 3.01)

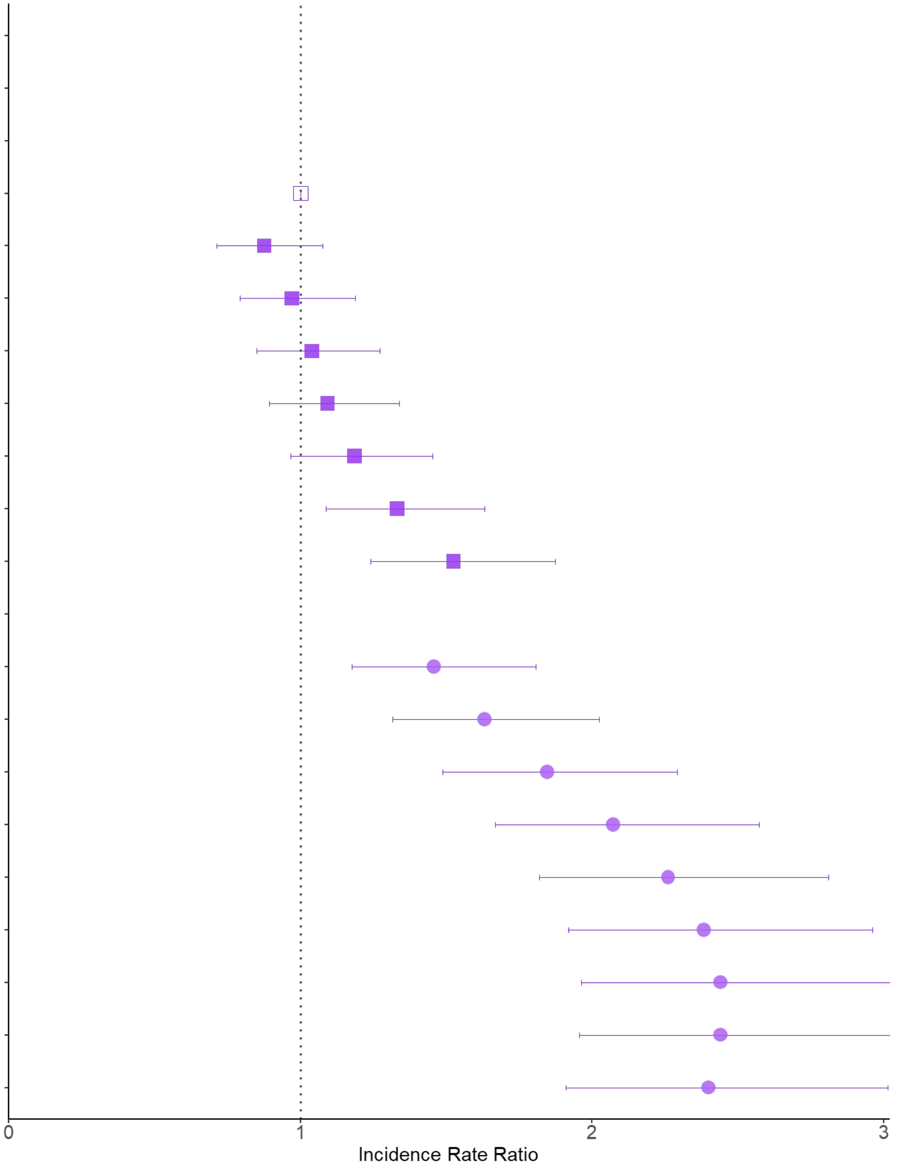

Supplementary Figure 3.67 All non-PCV10 ST IPD all-site weighted average IRRs comparing the annual post-PCV10/13 IR to the average pre-PCV IR for adults aged ≥65 years

PCV Product and PCV7 Impact Strata	Number of Sites	Number of Cases	IRR (95%CI)
PCV10			
No PCV7 impact			
Year 0	--	--	--
Year 1	4	230	1.02 (0.82, 1.26)
Year 2	4	307	1.1 (0.9, 1.36)
Year 3	4	311	1.28 (1.04, 1.58)
Year 4	3	274	1.54 (1.25, 1.9)
Year 5	3	359	1.74 (1.41, 2.14)
Year 6	2	372	1.8 (1.46, 2.21)
Year 7	2	401	1.78 (1.44, 2.2)
Substantial PCV7 impact			
Year −1	3	180	1.52 (1.23, 1.86)
Year 0	3	193	1.63 (1.33, 2.01)
Year 1	3	379	1.76 (1.43, 2.16)
Year 2	1	301	1.87 (1.52, 2.3)
Year 3	1	248	1.97 (1.6, 2.42)
Year 4	1	329	2.05 (1.66, 2.52)
Year 5	1	345	2.1 (1.71, 2.58)
Year 6	1	330	2.13 (1.73, 2.63)
Year 7	1	402	2.16 (1.74, 2.67)

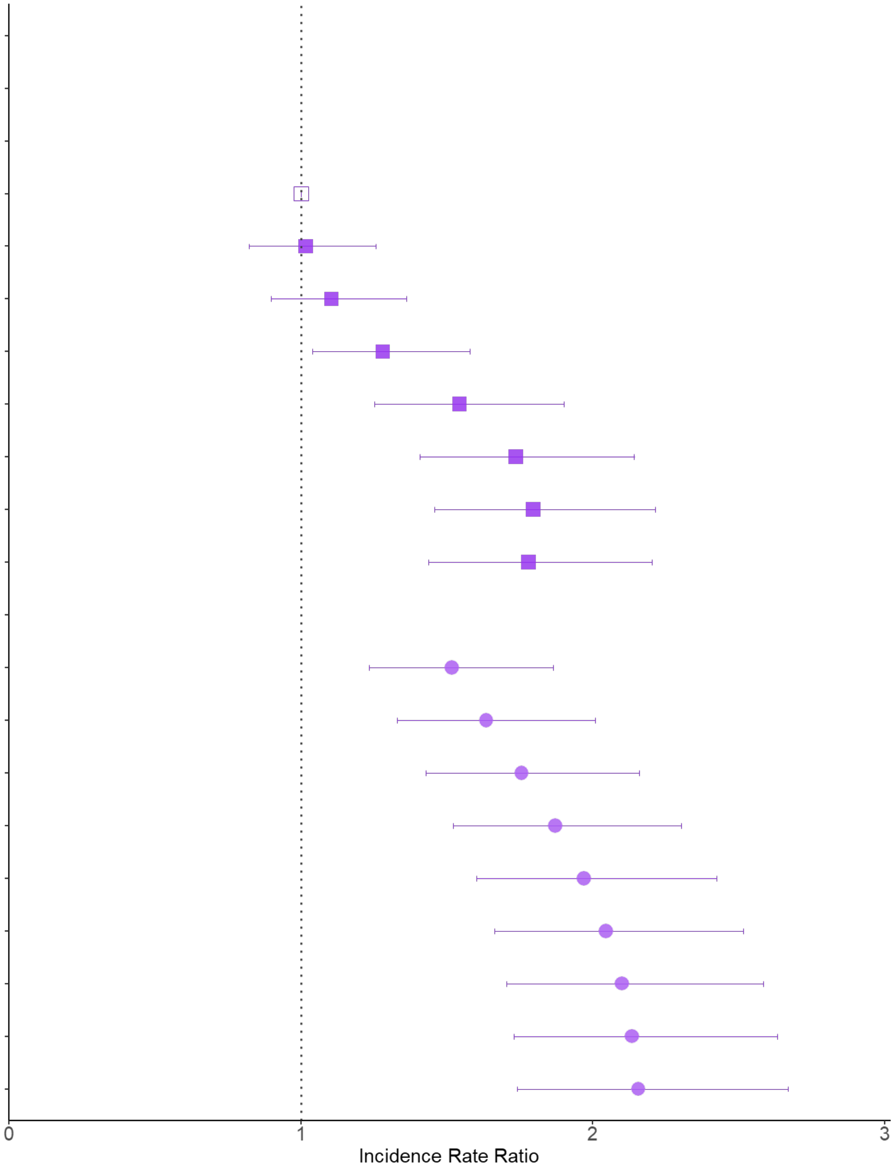

Supplementary Figure 3.68 Non-PCV10 ST minus ST6A IPD all-site weighted average IRRs comparing the annual post-PCV10/13 IR to the average pre-PCV IR for children aged <5 years

PCV Product and PCV7 Impact Strata	Number of Sites	Number of Cases	IRR (95%CI)
PCV10			
No PCV7 impact			
Year −1	--	--	--
Year 0	4	31	0.62 (0.43, 0.91)
Year 1	4	20	0.86 (0.59, 1.25)
Year 2	4	33	1.11 (0.76, 1.61)
Year 3	4	25	1.3 (0.9, 1.89)
Year 4	3	52	1.48 (1.02, 2.14)
Year 5	3	27	1.69 (1.16, 2.46)
Year 6	2	25	1.97 (1.36, 2.87)
Year 7	2	32	2.31 (1.58, 3.39)
Moderate PCV7 impact			
Year −1	2	23	1.18 (0.69, 2.01)
Year 0	2	28	1.32 (0.78, 2.25)
Year 1	2	63	1.45 (0.85, 2.47)
Year 2	2	85	1.63 (0.96, 2.78)
Year 3	1	97	1.95 (1.14, 3.33)
Year 4	1	76	2.5 (1.41, 4.42)
Substantial PCV7 impact			
Year −1	4	11	2.4 (1.64, 3.5)
Year 0	4	14	2.45 (1.67, 3.58)
Year 1	4	92	2.49 (1.7, 3.65)
Year 2	1	62	2.55 (1.73, 3.74)
Year 3	1	5	2.65 (1.8, 3.89)
Year 4	1	12	2.81 (1.91, 4.13)
Year 5	1	14	3.02 (2.04, 4.47)
Year 6	1	8	3.3 (2.2, 4.93)
Year 7	1	15	3.63 (2.37, 5.55)

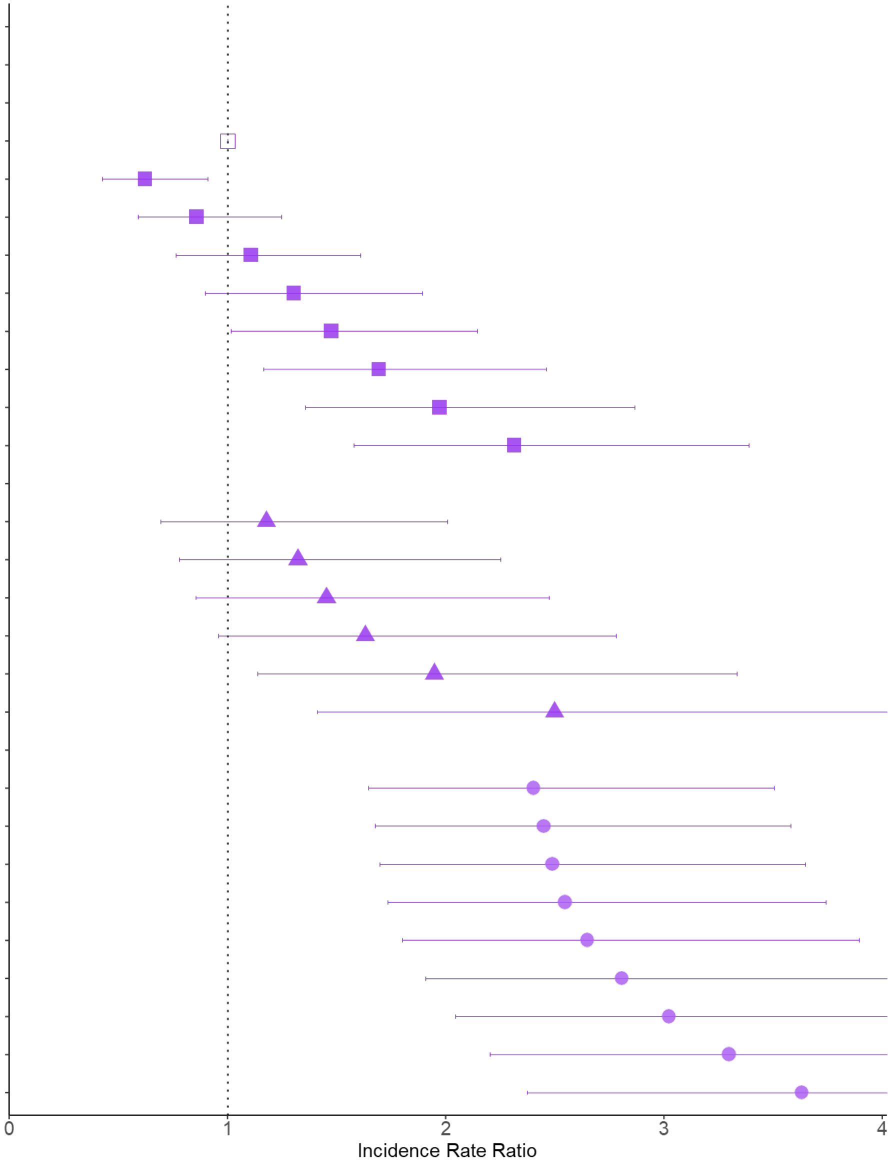

Supplementary Figure 3.69 Non-PCV10 ST minus ST6A IPD all-site weighted average IRRs comparing the annual post-PCV10/13 IR to the average pre-PCV IR for children aged 5–17 years

PCV Product and PCV7 Impact Strata	Number of Sites	Number of Cases	IRR (95%CI)
PCV10			
No PCV7 impact			
Year 0	--	--	--
Year 1	5	10	1.05 (0.55, 2)
Year 2	5	13	1.35 (0.72, 2.56)
Year 3	5	10	1.71 (0.9, 3.24)
Year 4	4	13	2.07 (1.09, 3.92)
Year 5	4	13	2.37 (1.25, 4.49)
Year 6	3	16	2.57 (1.36, 4.88)
Year 7	3	13	2.73 (1.42, 5.22)
Moderate PCV7 impact			
Year −1	2	4	0.77 (0.5, 1.17)
Year 0	2	5	1.03 (0.68, 1.58)
Year 1	2	20	1.29 (0.84, 1.96)
Year 2	2	28	1.47 (0.97, 2.25)
Year 3	1	7	1.54 (0.99, 2.37)
Year 4	1	11	1.46 (0.88, 2.41)
Substantial PCV7 impact			
Year −1	3	3	1.71 (1.22, 2.42)
Year 0	3	4	1.75 (1.24, 2.47)
Year 1	3	14	1.75 (1.24, 2.47)
Year 2	1	6	1.75 (1.24, 2.48)
Year 3	1	6	1.79 (1.26, 2.54)
Year 4	1	3	1.91 (1.35, 2.71)
Year 5	1	4	2.12 (1.49, 3.01)
Year 6	1	8	2.42 (1.69, 3.46)
Year 7	1	6	2.82 (1.94, 4.09)

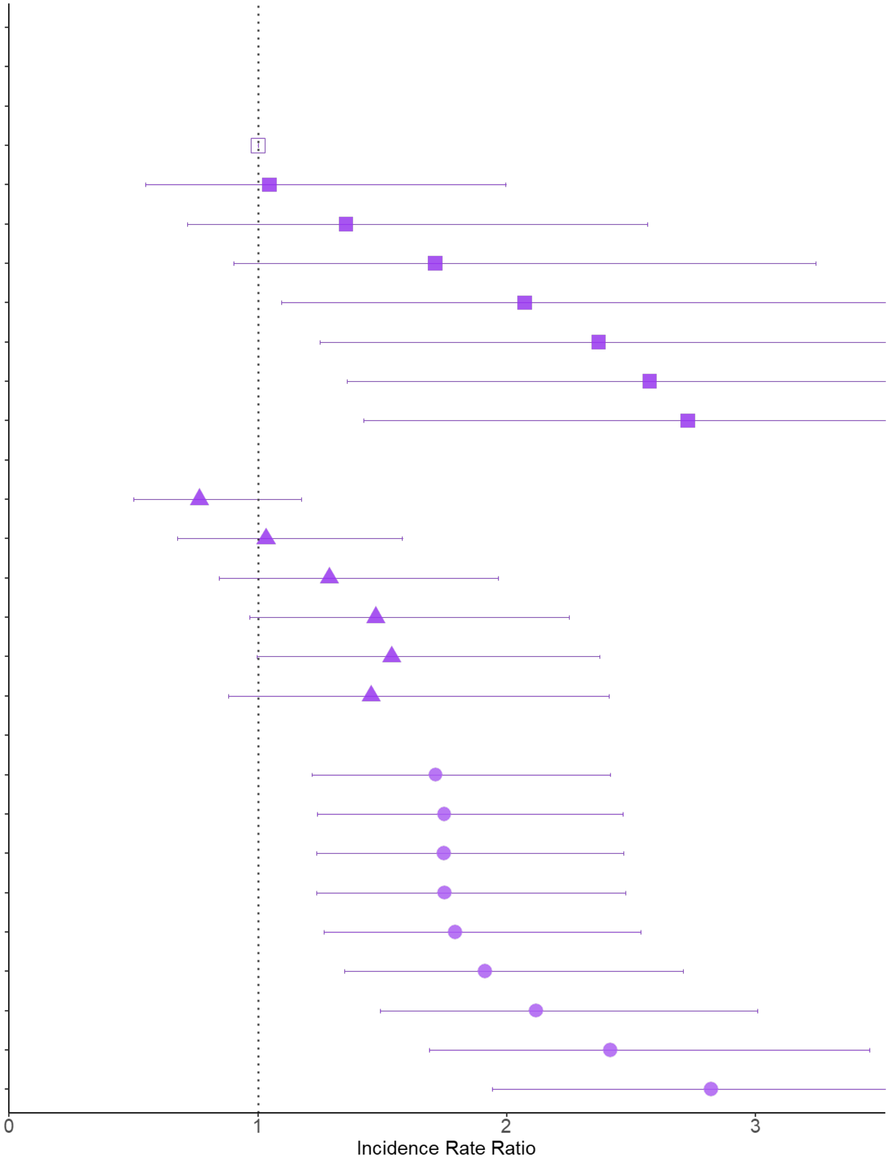

Supplementary Figure 3.70 Non-PCV10 ST minus ST6A IPD all-site weighted average IRRs comparing the annual post-PCV10/13 IR to the average pre-PCV IR for adults aged 18–49 years

PCV Product and PCV7 Impact Strata	Number of Site	Number of Cases	IRR (95%CI)
PCV10			
No PCV7 impact			
Year 0	--	--	--
Year 1	5	74	0.91 (0.55, 1.51)
Year 2	5	69	0.91 (0.56, 1.49)
Year 3	5	93	0.92 (0.56, 1.51)
Year 4	4	96	0.95 (0.58, 1.56)
Year 5	4	113	1.01 (0.61, 1.66)
Year 6	3	119	1.09 (0.67, 1.8)
Year 7	3	118	1.2 (0.72, 2)
Substantial PCV7 impact			
Year −1	3	37	1.24 (1.02, 1.51)
Year 0	3	32	1.31 (1.08, 1.6)
Year 1	3	99	1.41 (1.16, 1.71)
Year 2	1	58	1.51 (1.24, 1.85)
Year 3	1	39	1.62 (1.33, 1.98)
Year 4	1	61	1.73 (1.41, 2.1)
Year 5	1	51	1.82 (1.49, 2.22)
Year 6	1	48	1.91 (1.56, 2.34)
Year 7	1	59	2 (1.62, 2.47)

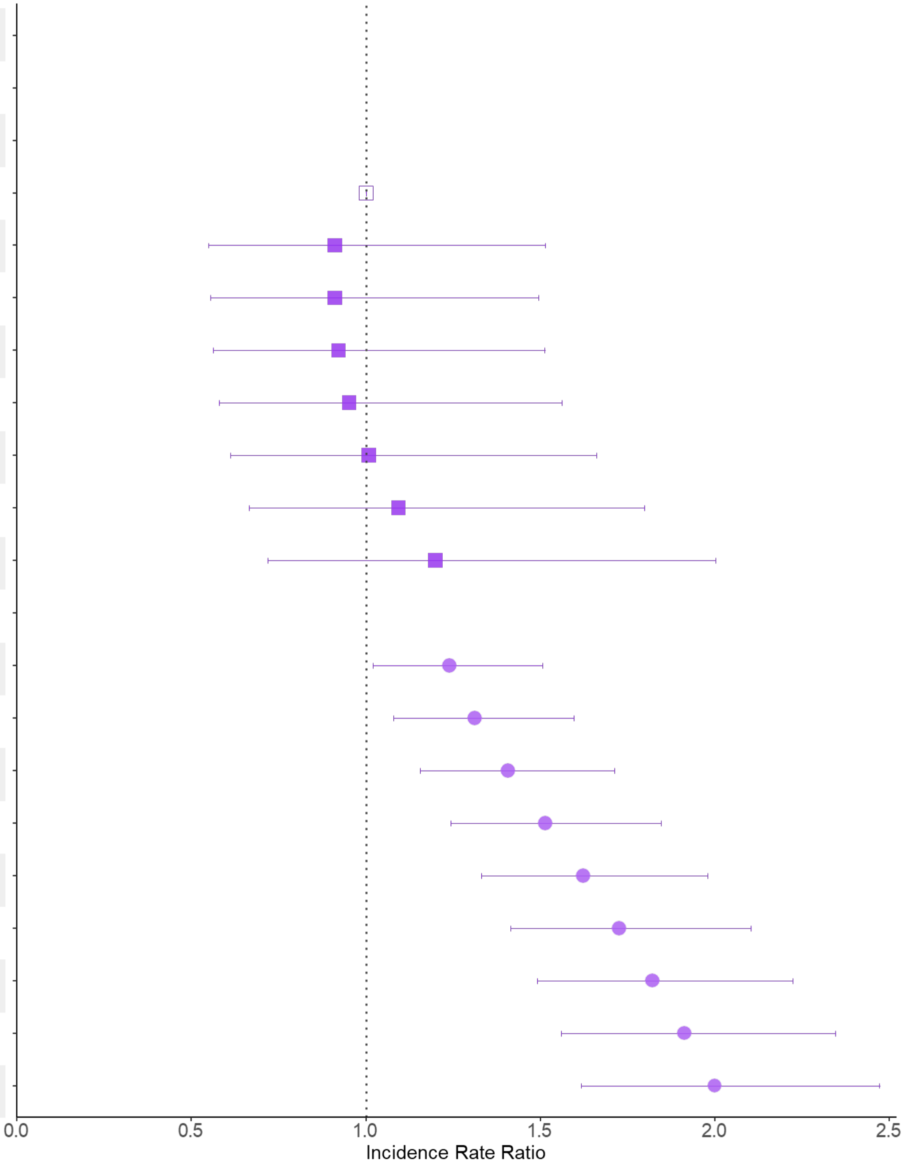

Supplementary Figure 3.71 Non-PCV10 ST minus ST6A IPD all-site weighted average IRRs comparing the annual post-PCV10/13 IR to the average pre-PCV IR for adults aged 50–64 years

PCV Product and PCV7 Impact Strata	Number of Sites	Number of Cases	IRR (95%CI)
PCV10			
No PCV7 impact			
Year 0	--	--	--
Year 1	4	126	0.89 (0.72, 1.11)
Year 2	4	152	1 (0.81, 1.24)
Year 3	4	161	1.1 (0.89, 1.36)
Year 4	2	107	1.19 (0.96, 1.47)
Year 5	2	159	1.31 (1.06, 1.62)
Year 6	2	176	1.47 (1.19, 1.82)
Year 7	2	166	1.67 (1.35, 2.06)
Substantial PCV7 impact			
Year −1	3	71	1.52 (1.2, 1.92)
Year 0	3	91	1.71 (1.35, 2.17)
Year 1	3	202	1.95 (1.54, 2.47)
Year 2	1	98	2.2 (1.73, 2.79)
Year 3	1	116	2.41 (1.9, 3.06)
Year 4	1	123	2.55 (2, 3.23)
Year 5	1	134	2.6 (2.05, 3.31)
Year 6	1	148	2.6 (2.04, 3.32)
Year 7	1	153	2.55 (1.98, 3.27)

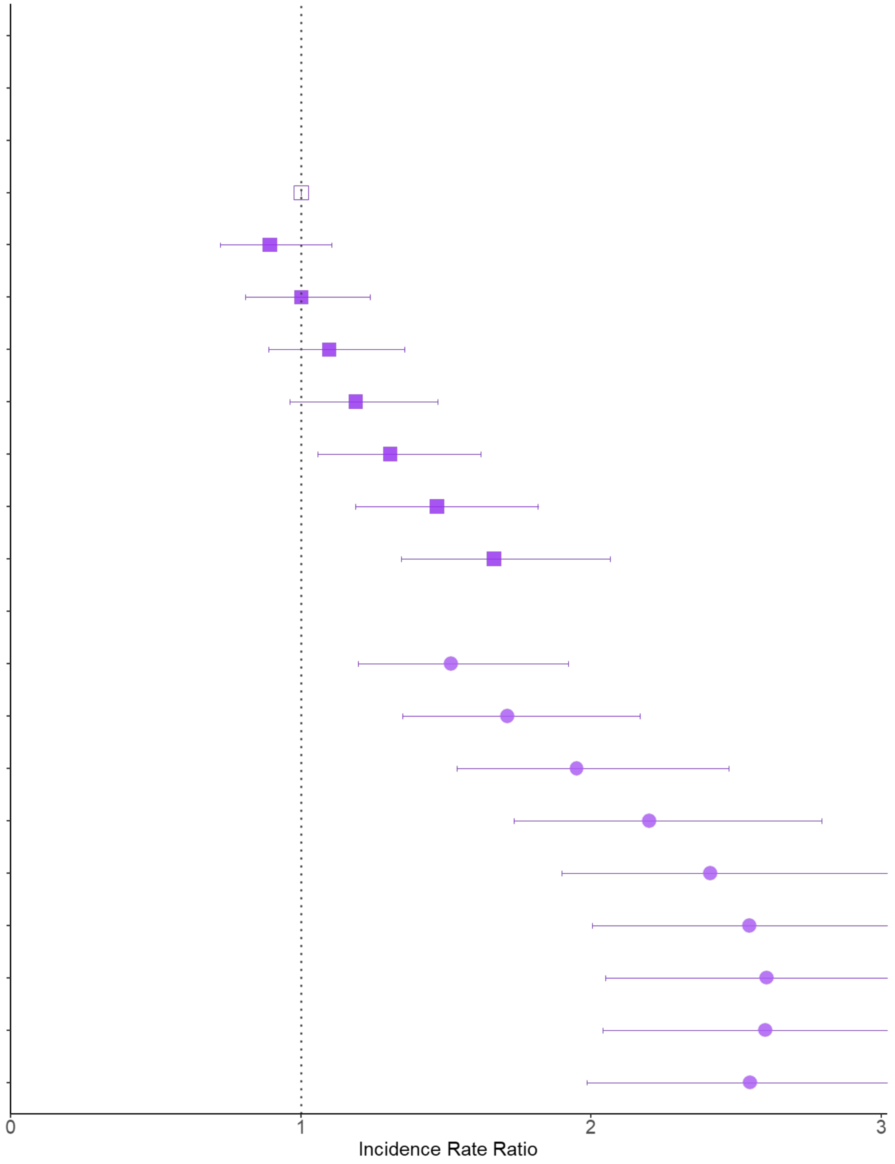

Supplementary Figure 3.72 Non-PCV10 ST minus ST6A IPD all-site weighted average IRRs comparing the annual post-PCV10/13 IR to the average pre-PCV IR for adults aged ≥65 years

PCV Product and PCV7 Impact Strata	Number of Sites	Number of Cases	IRR (95%CI)
PCV10			
No PCV7 impact			
Year 0	--	--	--
Year 1	4	212	1.06 (0.83, 1.35)
Year 2	4	295	1.17 (0.92, 1.48)
Year 3	4	294	1.36 (1.07, 1.73)
Year 4	3	255	1.66 (1.3, 2.1)
Year 5	3	347	1.89 (1.48, 2.4)
Year 6	2	362	1.99 (1.57, 2.53)
Year 7	2	391	2.01 (1.58, 2.57)
Substantial PCV7 impact			
Year −1	3	171	1.54 (1.24, 1.9)
Year 0	3	186	1.67 (1.35, 2.07)
Year 1	3	370	1.8 (1.45, 2.23)
Year 2	1	299	1.92 (1.55, 2.38)
Year 3	1	248	2.02 (1.63, 2.51)
Year 4	1	327	2.1 (1.69, 2.61)
Year 5	1	345	2.16 (1.74, 2.69)
Year 6	1	328	2.21 (1.78, 2.75)
Year 7	1	399	2.24 (1.8, 2.81)

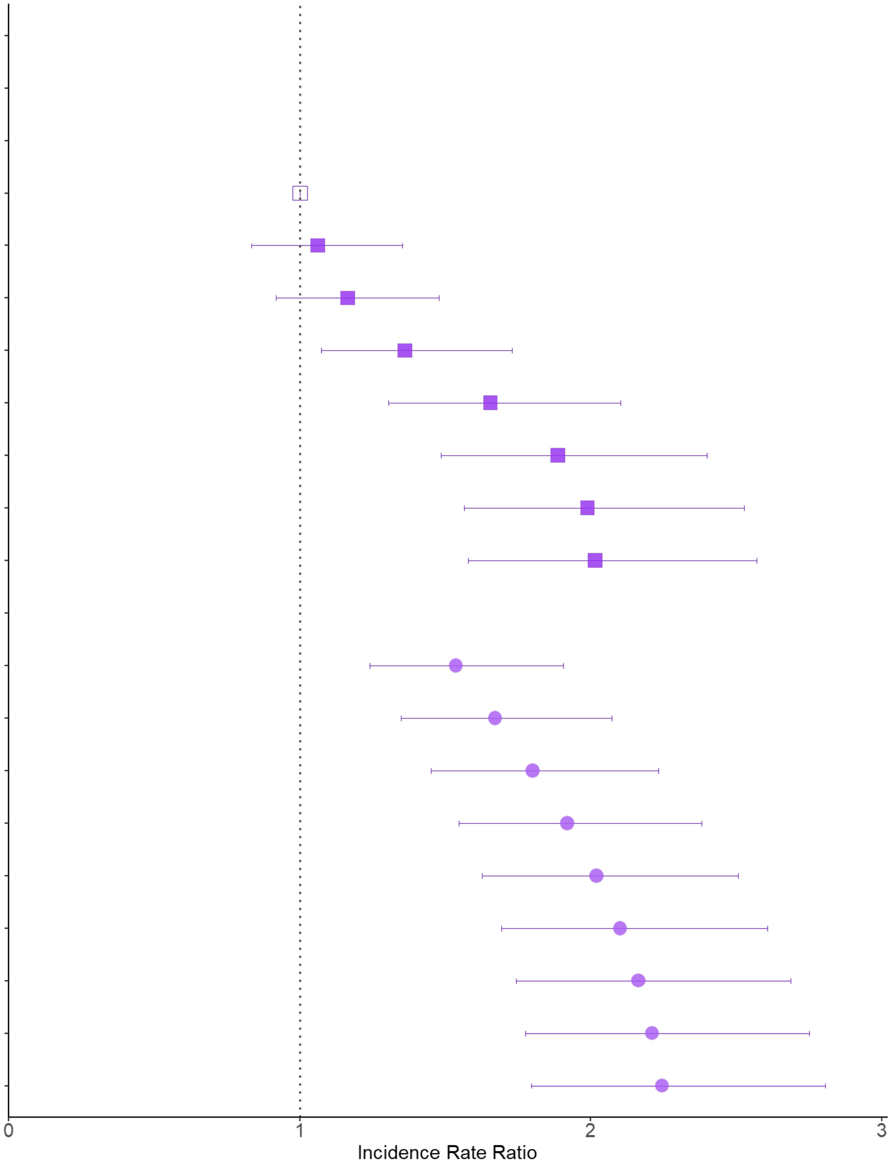

Supplementary Figure 3.73 All non-PCV13 ST IPD all-site weighted average IRRs comparing the annual post-PCV10/13 IR to the average pre-PCV IR for children aged <5 years

PCV Product and PCV7 Impact Strata	Number of Sites	Number of Cases	IRR (95%CI)
PCV13			
Moderate PCV7 impact			
Year −1	6	338	1.51 (1.15, 1.99)
Year 0	6	368	1.76 (1.34, 2.33)
Year 1	6	368	2.04 (1.55, 2.69)
Year 2	6	533	2.3 (1.74, 3.03)
Year 3	6	548	2.49 (1.89, 3.29)
Year 4	6	628	2.6 (1.97, 3.43)
Year 5	6	556	2.62 (1.99, 3.46)
Year 6	6	583	2.58 (1.96, 3.4)
Year 7	5	602	2.48 (1.88, 3.28)
Year 8	3	583	2.36 (1.79, 3.12)
Substantial PCV7 impact			
Year −1	11	369	1.7 (1.39, 2.09)
Year 0	11	413	1.8 (1.47, 2.21)
Year 1	11	311	1.9 (1.55, 2.34)
Year 2	11	359	2.01 (1.64, 2.47)
Year 3	11	378	2.13 (1.74, 2.61)
Year 4	11	421	2.25 (1.83, 2.76)
Year 5	11	473	2.38 (1.94, 2.91)
Year 6	11	524	2.51 (2.04, 3.07)
Year 7	7	501	2.64 (2.15, 3.24)
Year 8	4	326	2.78 (2.27, 3.42)
Year 9	1	246	2.93 (2.38, 3.61)
PCV10			
No PCV7 impact			
Year −1	--	--	--
Year 0	4	12	0.84 (0.61, 1.17)
Year 1	4	11	1.1 (0.8, 1.51)
Year 2	4	16	1.36 (0.99, 1.86)
Year 3	4	15	1.55 (1.13, 2.12)
Year 4	3	26	1.72 (1.25, 2.36)
Year 5	3	13	1.97 (1.44, 2.71)
Year 6	2	9	2.33 (1.69, 3.2)
Year 7	2	13	2.78 (2, 3.86)
Moderate PCV7 impact			
Year −1	2	36	1.16 (0.72, 1.88)
Year 0	2	37	1.34 (0.83, 2.17)
Year 1	2	38	1.48 (0.92, 2.39)
Year 2	2	52	1.61 (1, 2.61)
Year 3	1	62	1.77 (1.09, 2.86)
Year 4	1	35	1.97 (1.19, 3.26)
Substantial PCV7 impact			
Year −1	4	24	2.44 (1.73, 3.43)
Year 0	4	20	2.4 (1.7, 3.37)
Year 1	4	47	2.35 (1.66, 3.31)
Year 2	1	24	2.34 (1.66, 3.31)
Year 3	1	4	2.42 (1.71, 3.42)
Year 4	1	10	2.61 (1.84, 3.68)
Year 5	1	9	2.9 (2.04, 4.11)
Year 6	1	5	3.31 (2.32, 4.74)
Year 7	1	10	3.87 (2.66, 5.61)

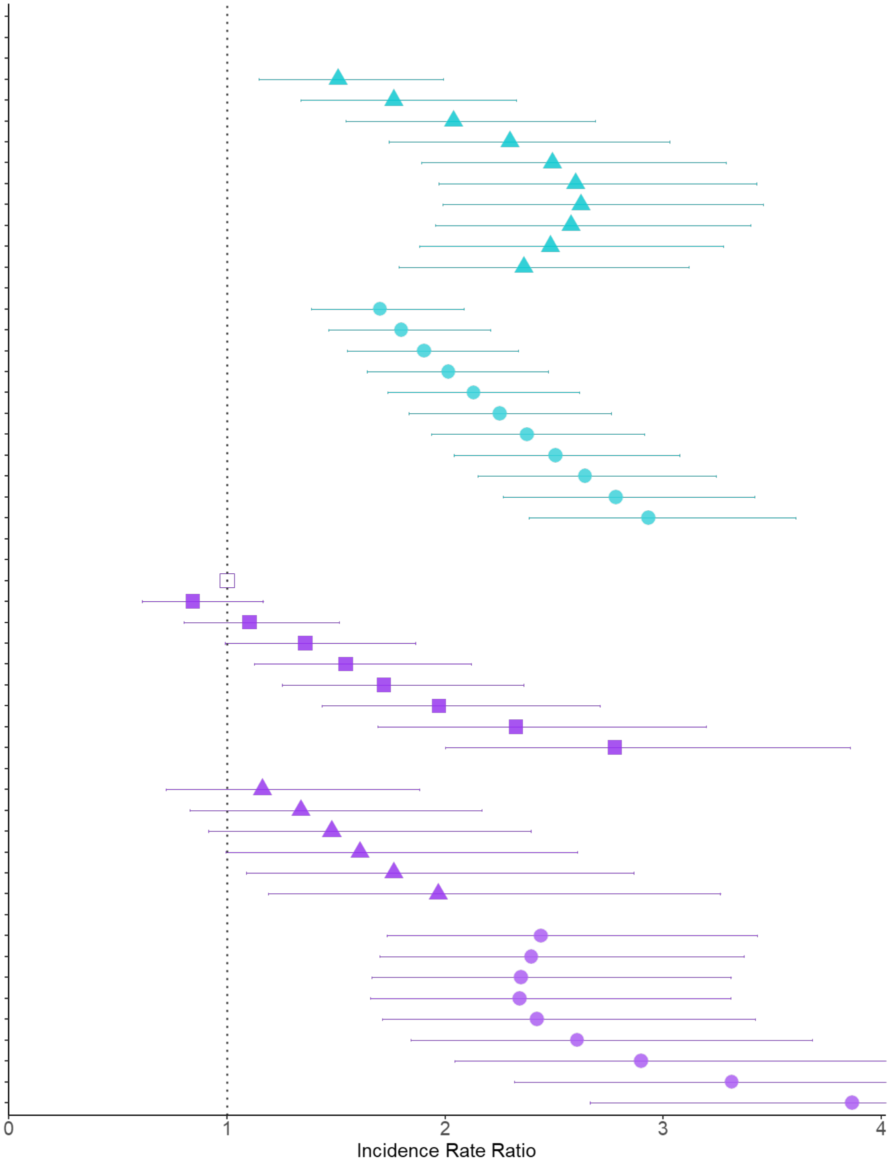

Supplementary Figure 3.74 All non-PCV13 ST IPD all-site weighted average IRRs comparing the annual post-PCV10/13 IR to the average pre-PCV IR for children aged 5–17 years

PCV Product and PCV7 Impact Strata	Number of Sites	Number of Cases	IRR (95%CI)
PCV13			
Moderate PCV7 impact			
Year −1	5	103	1.11 (0.87, 1.41)
Year 0	6	121	1.19 (0.93, 1.51)
Year 1	6	148	1.26 (0.99, 1.6)
Year 2	6	124	1.33 (1.04, 1.69)
Year 3	6	145	1.39 (1.1, 1.77)
Year 4	6	141	1.46 (1.15, 1.86)
Year 5	6	143	1.54 (1.21, 1.96)
Year 6	6	151	1.62 (1.28, 2.06)
Year 7	5	162	1.71 (1.34, 2.18)
Year 8	3	79	1.8 (1.41, 2.3)
Substantial PCV7 impact			
Year −1	10	119	1.31 (1.09, 1.58)
Year 0	10	141	1.36 (1.13, 1.63)
Year 1	10	137	1.41 (1.17, 1.7)
Year 2	10	155	1.47 (1.22, 1.77)
Year 3	10	160	1.53 (1.27, 1.84)
Year 4	10	171	1.58 (1.31, 1.9)
Year 5	10	177	1.62 (1.35, 1.95)
Year 6	10	175	1.66 (1.38, 2)
Year 7	7	130	1.69 (1.4, 2.04)
Year 8	4	74	1.72 (1.43, 2.08)
Year 9	1	56	1.75 (1.45, 2.12)
PCV10			
No PCV7 impact			
Year 0	--	--	--
Year 1	5	4	1.2 (0.55, 2.64)
Year 2	5	9	1.41 (0.64, 3.07)
Year 3	5	6	1.57 (0.72, 3.44)
Year 4	4	4	1.68 (0.77, 3.68)
Year 5	4	6	1.82 (0.83, 3.99)
Year 6	3	5	2.03 (0.93, 4.44)
Year 7	3	5	2.3 (1.04, 5.07)
Moderate PCV7 impact			
Year −1	2	8	0.87 (0.57, 1.33)
Year 0	2	6	1.2 (0.79, 1.83)
Year 1	2	16	1.56 (1.03, 2.37)
Year 2	2	22	1.73 (1.14, 2.63)
Year 3	1	6	1.47 (0.95, 2.26)
Year 4	1	4	0.89 (0.54, 1.46)
Substantial PCV7 impact			
Year −1	3	8	1.4 (1, 1.97)
Year 0	3	11	1.49 (1.06, 2.1)
Year 1	3	11	1.58 (1.12, 2.22)
Year 2	1	5	1.67 (1.18, 2.36)
Year 3	1	6	1.78 (1.26, 2.51)
Year 4	1	3	1.92 (1.36, 2.71)
Year 5	1	4	2.1 (1.48, 2.97)
Year 6	1	6	2.31 (1.63, 3.29)
Year 7	1	5	2.57 (1.78, 3.7)

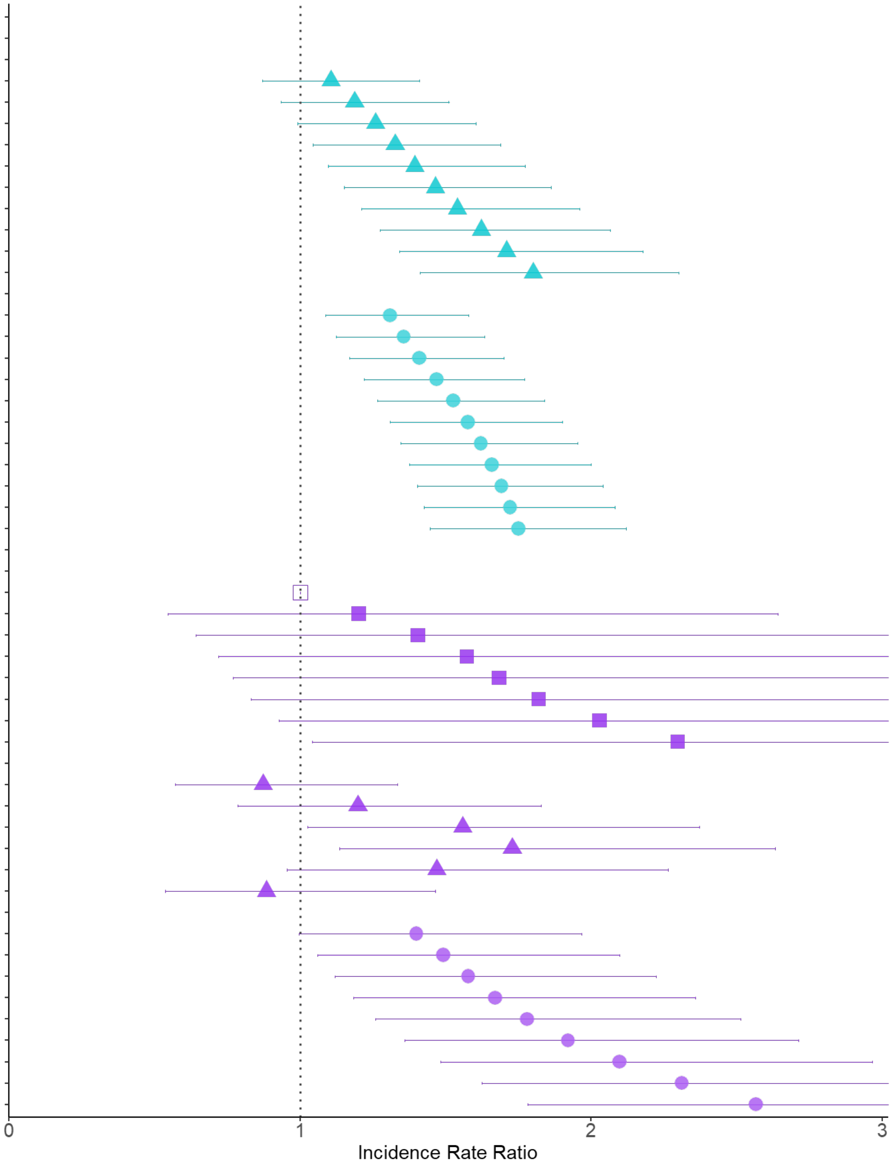

Supplementary Figure 3.75 All non-PCV13 ST IPD all-site weighted average IRRs comparing the annual post-PCV10/13 IR to the average pre-PCV IR for adults aged 18–49 years

PCV Product and PCV7 Impact Strata	Number of Sites	Number of Cases	IRR (95%CI)
PCV13			
Moderate PCV7 impact			
Year −1	4	592	1.27 (1.07, 1.51)
Year 0	4	723	1.33 (1.12, 1.58)
Year 1	4	747	1.37 (1.16, 1.63)
Year 2	4	1006	1.4 (1.18, 1.67)
Year 3	4	920	1.42 (1.19, 1.68)
Year 4	4	1064	1.42 (1.2, 1.69)
Year 5	4	1104	1.43 (1.2, 1.7)
Year 6	4	1106	1.43 (1.2, 1.7)
Year 7	3	931	1.43 (1.2, 1.7)
Year 8	2	412	1.43 (1.2, 1.7)
Substantial PCV7 impact			
Year −1	10	1147	1.27 (1.14, 1.41)
Year 0	10	1094	1.26 (1.13, 1.4)
Year 1	10	1095	1.25 (1.12, 1.39)
Year 2	10	1119	1.26 (1.13, 1.41)
Year 3	10	1246	1.3 (1.17, 1.45)
Year 4	10	1206	1.37 (1.23, 1.53)
Year 5	10	1454	1.47 (1.32, 1.64)
Year 6	10	1708	1.61 (1.44, 1.79)
Year 7	7	1361	1.77 (1.59, 1.97)
Year 8	4	920	1.97 (1.76, 2.2)
Year 9	1	720	2.19 (1.96, 2.45)
PCV10			
No PCV7 impact			
Year 0	--	--	--
Year 1	5	50	1.01 (0.65, 1.58)
Year 2	5	43	0.98 (0.64, 1.52)
Year 3	5	61	0.95 (0.61, 1.46)
Year 4	4	44	0.91 (0.59, 1.4)
Year 5	4	53	0.87 (0.56, 1.35)
Year 6	3	58	0.85 (0.55, 1.31)
Year 7	3	39	0.83 (0.53, 1.3)
Substantial PCV7 impact			
Year −1	3	36	1.16 (0.95, 1.41)
Year 0	3	54	1.24 (1.02, 1.51)
Year 1	3	69	1.35 (1.11, 1.65)
Year 2	1	41	1.48 (1.21, 1.8)
Year 3	1	34	1.61 (1.32, 1.97)
Year 4	1	50	1.75 (1.43, 2.13)
Year 5	1	40	1.88 (1.54, 2.3)
Year 6	1	41	2.01 (1.64, 2.47)
Year 7	1	44	2.15 (1.73, 2.66)

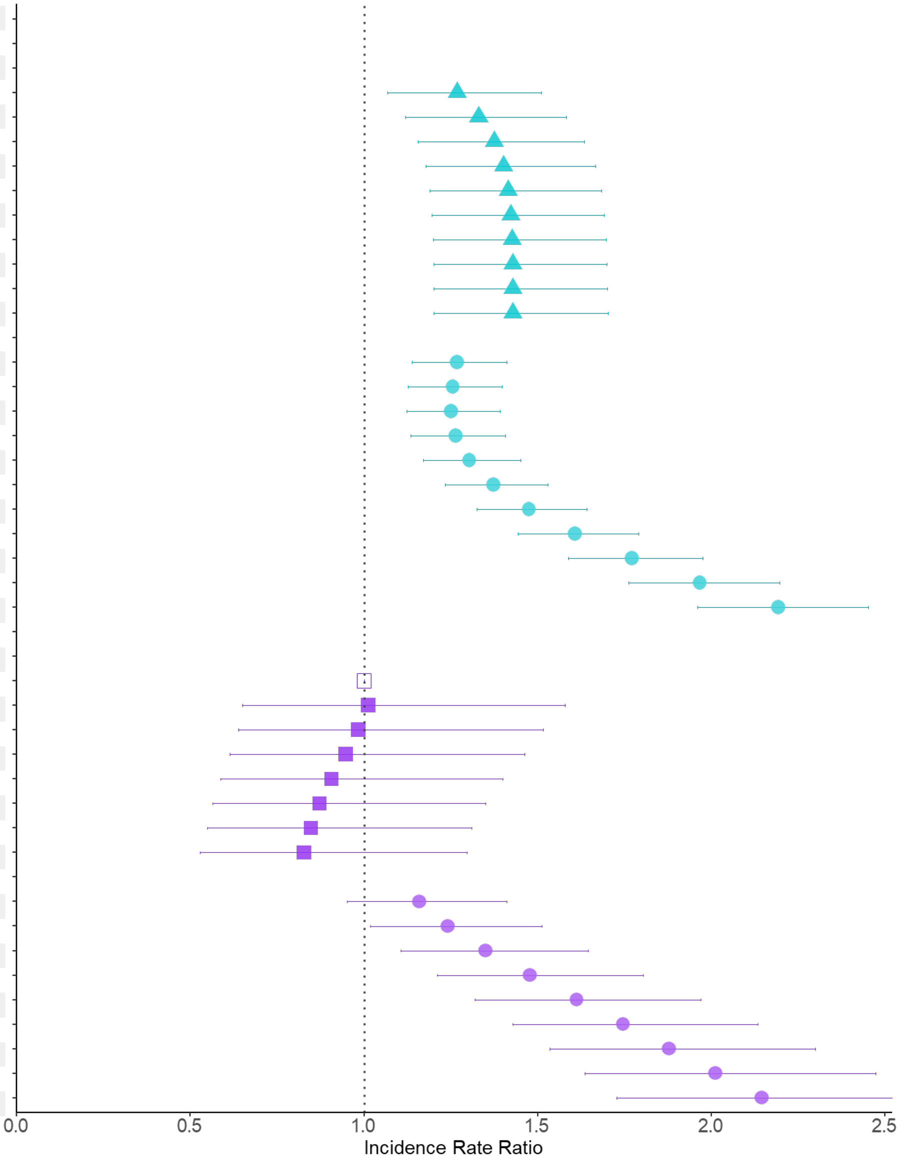

Supplementary Figure 3.76 All non-PCV13 ST IPD all-site weighted average IRRs comparing the annual post-PCV10/13 IR to the average pre-PCV IR for adults aged 50–64 years

PCV Product and PCV7 Impact Strata	Number of Sites	Number of Cases	IRR (95%CI)
PCV13			
Moderate PCV7 impact			
Year −1	4	343	1.43 (1.11, 1.83)
Year 0	4	369	1.55 (1.21, 1.99)
Year 1	4	508	1.67 (1.3, 2.14)
Year 2	4	742	1.77 (1.38, 2.27)
Year 3	4	842	1.85 (1.44, 2.37)
Year 4	4	942	1.92 (1.49, 2.46)
Year 5	4	1068	1.98 (1.54, 2.54)
Year 6	4	1185	2.03 (1.58, 2.61)
Year 7	3	1024	2.08 (1.62, 2.67)
Year 8	2	822	2.12 (1.65, 2.73)
Substantial PCV7 impact			
Year −1	10	1237	1.49 (1.3, 1.71)
Year 0	10	1251	1.52 (1.32, 1.74)
Year 1	10	1375	1.55 (1.35, 1.78)
Year 2	10	1519	1.6 (1.39, 1.84)
Year 3	10	1662	1.68 (1.46, 1.92)
Year 4	10	1707	1.79 (1.56, 2.05)
Year 5	10	1979	1.93 (1.68, 2.21)
Year 6	10	2259	2.11 (1.84, 2.42)
Year 7	7	1839	2.33 (2.03, 2.67)
Year 8	4	1027	2.58 (2.25, 2.97)
Year 9	1	844	2.87 (2.49, 3.31)
PCV10			
No PCV7 impact			
Year 0	--	--	--
Year 1	4	92	1.24 (0.89, 1.74)
Year 2	4	104	1.35 (0.96, 1.88)
Year 3	4	97	1.33 (0.95, 1.86)
Year 4	2	63	1.27 (0.91, 1.77)
Year 5	2	86	1.3 (0.93, 1.82)
Year 6	2	91	1.46 (1.05, 2.04)
Year 7	2	91	1.72 (1.23, 2.41)
Substantial PCV7 impact			
Year −1	3	56	1.48 (1.15, 1.91)
Year 0	3	72	1.66 (1.29, 2.13)
Year 1	3	132	1.87 (1.45, 2.41)
Year 2	1	76	2.09 (1.63, 2.7)
Year 3	1	90	2.28 (1.77, 2.94)
Year 4	1	93	2.4 (1.86, 3.09)
Year 5	1	101	2.44 (1.89, 3.15)
Year 6	1	103	2.43 (1.88, 3.14)
Year 7	1	104	2.37 (1.82, 3.09)

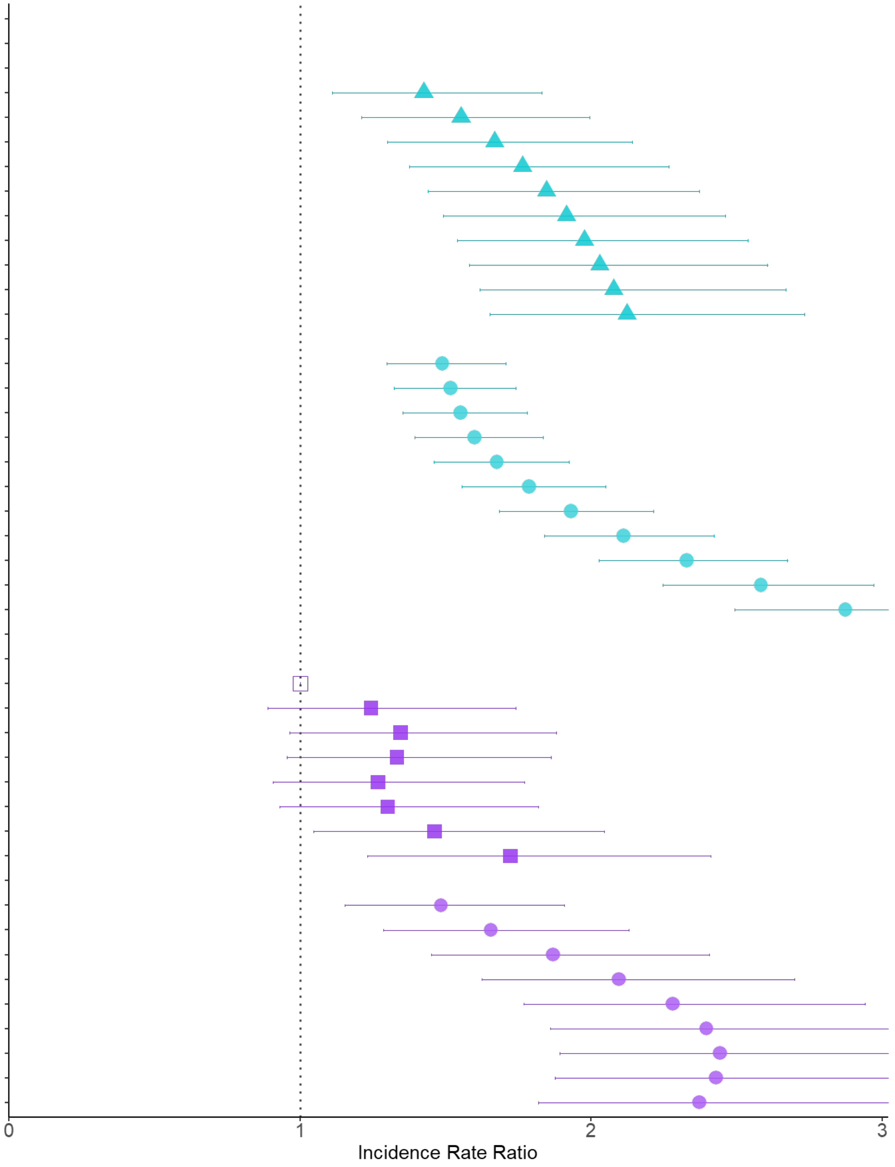

Supplementary Figure 3.77 All non-PCV13 ST IPD all-site weighted average IRRs comparing the annual post-PCV10/13 IR to the average pre-PCV IR for adults aged ≥65 years

PCV Product and PCV7 Impact Strata	Number of Sites	Number of Cases	IRR (95%CI)
PCV13			
Moderate PCV7 impact			
Year −1	4	498	1.51 (1.22, 1.88)
Year 0	4	696	1.64 (1.32, 2.04)
Year 1	4	837	1.77 (1.43, 2.2)
Year 2	4	1534	1.91 (1.54, 2.37)
Year 3	4	1718	2.04 (1.64, 2.54)
Year 4	4	1856	2.15 (1.73, 2.67)
Year 5	4	2327	2.22 (1.78, 2.75)
Year 6	4	2395	2.26 (1.81, 2.8)
Year 7	3	2297	2.27 (1.83, 2.83)
Year 8	2	2023	2.28 (1.83, 2.84)
Substantial PCV7 impact			
Year −1	10	2094	1.55 (1.4, 1.72)
Year 0	10	2237	1.6 (1.44, 1.77)
Year 1	10	2549	1.65 (1.49, 1.83)
Year 2	10	2712	1.71 (1.54, 1.9)
Year 3	10	3011	1.78 (1.6, 1.97)
Year 4	10	2946	1.86 (1.67, 2.06)
Year 5	10	3517	1.95 (1.76, 2.16)
Year 6	10	3938	2.05 (1.85, 2.27)
Year 7	7	3218	2.16 (1.95, 2.4)
Year 8	4	2171	2.28 (2.05, 2.54)
Year 9	1	1794	2.41 (2.16, 2.69)
PCV10			
No PCV7 impact			
Year 0	--	--	--
Year 1	4	122	1.08 (0.63, 1.85)
Year 2	4	186	1.18 (0.69, 2.02)
Year 3	4	176	1.35 (0.79, 2.31)
Year 4	3	150	1.58 (0.92, 2.69)
Year 5	3	206	1.72 (1.01, 2.94)
Year 6	2	202	1.74 (1.02, 2.97)
Year 7	2	214	1.69 (0.99, 2.89)
Substantial PCV7 impact			
Year −1	3	126	1.46 (1.2, 1.76)
Year 0	3	133	1.61 (1.33, 1.95)
Year 1	3	249	1.77 (1.46, 2.14)
Year 2	1	228	1.92 (1.58, 2.32)
Year 3	1	200	2.04 (1.68, 2.47)
Year 4	1	250	2.13 (1.76, 2.58)
Year 5	1	260	2.18 (1.8, 2.65)
Year 6	1	246	2.21 (1.82, 2.68)
Year 7	1	290	2.21 (1.81, 2.7)

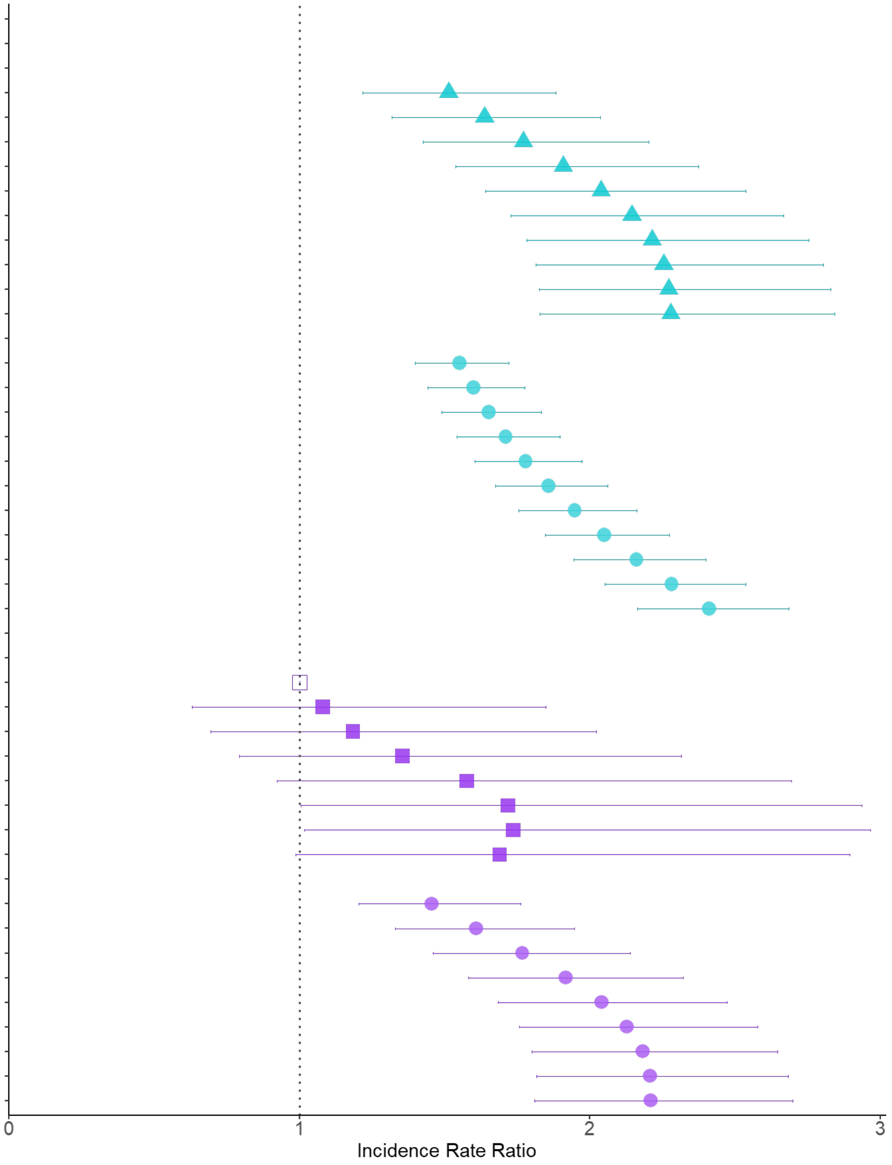

4. Sensitivity Analyses

Supplementary Figure 4.1 Sensitivity analysis excluding sites without pre-PCV data compared to results from the primary analysis: All-site weighted average IRRs for all serotype IPD for children aged <5 years, comparing the annual post-PCV10/13 IR to the average pre-PCV IR. The sensitivity analysis excluded 2 of 14 PCV10 sites and 10 of 31 PCV13 sites without pre-PCV years of data.

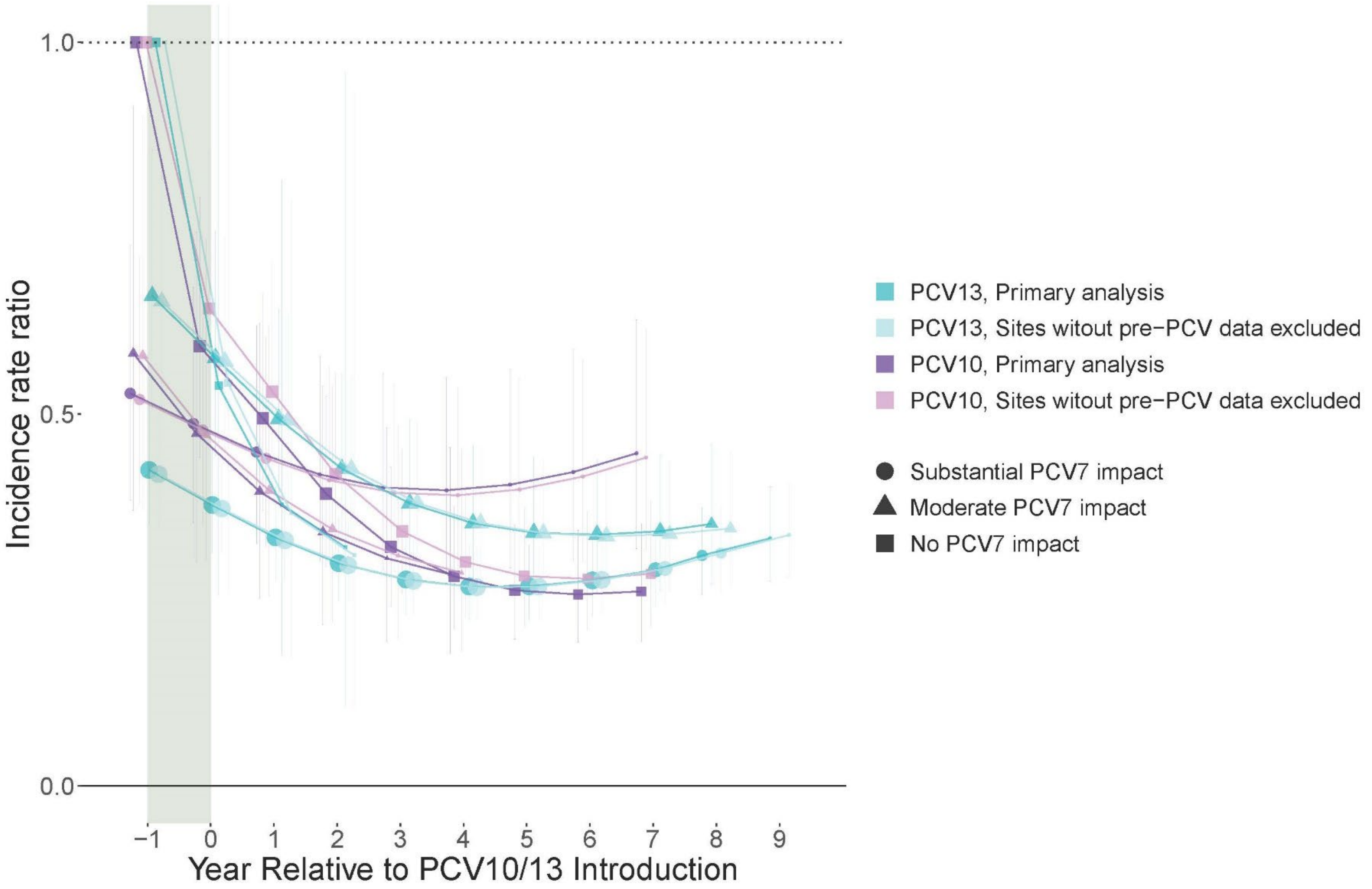

Supplementary Figure 4.2 Sensitivity analysis comparing all-site weighted average IRRs when estimated using (1) a single linear mixed effects regression including data from all sites with a three-way interaction between year since PCV10/13 introduction, prior PCV7 impact, and product (PCV10 or PCV13) (i.e., the primary analysis) versus (2) separate linear mixed effects regressions for each PCV product and PCV7 impact strata (i.e., six separate models). Shown below for all serotype IPD for children <5 years.

For the primary analysis, all-site weighted average IRRs were estimated for all PCV7 use-product strata in a single model (instead of stratum-specific models). Random intercepts by site to account for approximately exchangeable correlation among multiple years for a given site allow for each stratum to deviate from the mean, i.e., the curves of all six strata are not forced by the model to be the same. For both the combined and separate model, time was modeled with two cubic spline knots at the 33rd and 67th percentiles of the distribution of observed time to allow for flexibility in the curve.

The all-site weighted average IRRs from the single stratum models provided estimates similar to those in the combined model. Among strata-years with >1 site, IRR point estimates were within ≤0.03 of each other and confidence bound widths within ≤0.1 of each other. Larger differences in point estimates and confidence bound widths occurred in strata-years with only one site. Differences between the two models arose for two reasons. The first is how the two methods estimate the variance for the random intercept: in the combined model it is pooled across sites while in the separate models it is estimated for each stratum which can produce unreliable variance estimates for strata with small samples sizes. Second, the cubic spline knots were at different locations for each model because spline knots were placed at the 33rd and 67th percentile of the distribution of observed time and this varied across strata. The different knot locations results in flexibility in the curves at different times across each model. For example, in a stratum for which the majority of the data was concentrated at the beginning of the time period, the knots would be concentrated at earlier years. Conversely, in the combined model, which had a more even distribution of data across time, the knots were at locations that better fit the data, thus producing curves that better fit the data.

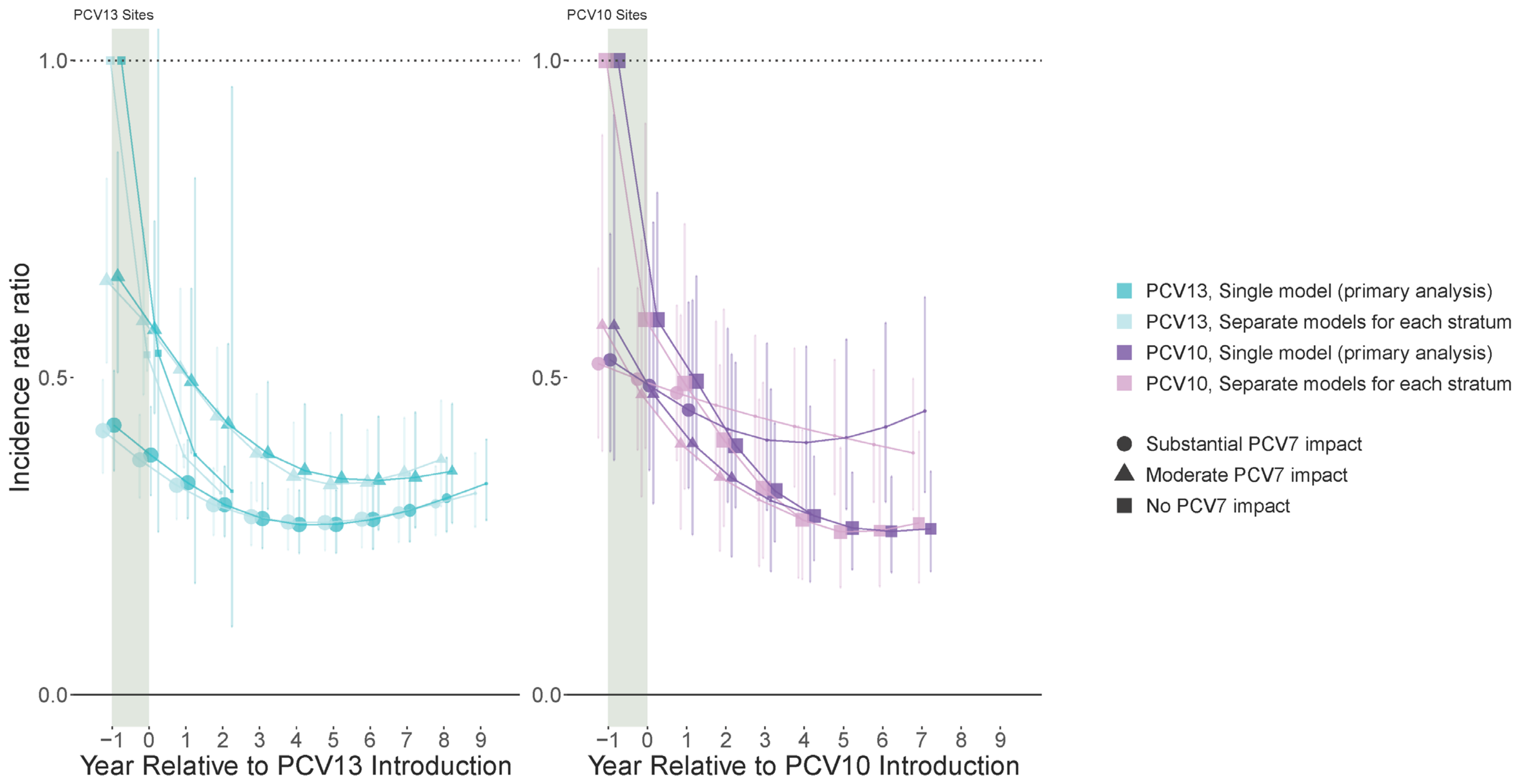

PCV13 Sites
PCV10 Sites
Incidence rate ratio
1.0
0.5
0.0
−1 0 1 2 3 4 5 6 7 8 9
Year Relative to PCV13 Introduction
Year Relative to PCV10 Introduction
PCV13, Single model (primary analysis)
PCV13, Separate models for each stratum
PCV10, Single model (primary analysis)
PCV10, Separate models for each stratum
Substantial PCV7 impact
Moderate PCV7 impact
No PCV7 impact
